# An accessible infrastructure for artificial intelligence using a Docker-based JupyterLab in Galaxy

**DOI:** 10.1093/gigascience/giad028

**Published:** 2023-04-26

**Authors:** Anup Kumar, Gianmauro Cuccuru, Björn Grüning, Rolf Backofen

**Affiliations:** Bioinformatics Group, Department of Computer Science, University of Freiburg, Georges-Koehler-Allee 106, 79110 Freiburg, Germany; Bioinformatics Group, Department of Computer Science, University of Freiburg, Georges-Koehler-Allee 106, 79110 Freiburg, Germany; Bioinformatics Group, Department of Computer Science, University of Freiburg, Georges-Koehler-Allee 106, 79110 Freiburg, Germany; Bioinformatics Group, Department of Computer Science, University of Freiburg, Georges-Koehler-Allee 106, 79110 Freiburg, Germany; Signalling Research Centres BIOSS and CIBSS, University of Freiburg, Schaenzlestr. 18, 79104 Freiburg, Germany

**Keywords:** JupyterLab, Galaxy Europe, artificial intelligence, remote model training, ONNX, Elyra AI, GPU, CUDA

## Abstract

**Background:**

Artificial intelligence (AI) programs that train on large datasets require powerful compute infrastructure consisting of several CPU cores and GPUs. JupyterLab provides an excellent framework for developing AI programs, but it needs to be hosted on such an infrastructure to enable faster training of AI programs using parallel computing.

**Findings:**

An open-source, docker-based, and GPU-enabled JupyterLab infrastructure is developed that runs on the public compute infrastructure of Galaxy Europe consisting of thousands of CPU cores, many GPUs, and several petabytes of storage to rapidly prototype and develop end-to-end AI projects. Using a JupyterLab notebook, long-running AI model training programs can also be executed remotely to create trained models, represented in open neural network exchange (ONNX) format, and other output datasets in Galaxy. Other features include Git integration for version control, the option of creating and executing pipelines of notebooks, and multiple dashboards and packages for monitoring compute resources and visualization, respectively.

**Conclusions:**

These features make JupyterLab in Galaxy Europe highly suitable for creating and managing AI projects. A recent scientific publication that predicts infected regions in COVID-19 computed tomography scan images is reproduced using various features of JupyterLab on Galaxy Europe. In addition, ColabFold, a faster implementation of AlphaFold2, is accessed in JupyterLab to predict the 3-dimensional structure of protein sequences. JupyterLab is accessible in 2 ways—one as an interactive Galaxy tool and the other by running the underlying Docker container. In both ways, long-running training can be executed on Galaxy’s compute infrastructure. Scripts to create the Docker container are available under MIT license at https://github.com/usegalaxy-eu/gpu-jupyterlab-docker.

## Findings

### Background

Bioinformatics comprises many subfields, such as single cell, medical imaging, sequencing, proteomics, and many more, that produce a huge amount of biological data in myriad formats. For example, the single-cell field creates gene expression patterns for each cell that are represented as matrices of real numbers. The medical imaging field generates images of cells and tissues, radiography images such as chest x-rays, and computed tomography (CT) scans. Next-generation sequencing generates DNA sequences that are stored as FASTA and FASTQ [1] files. Machine learning (ML) approaches are being increasingly used with these datasets [2] for predictive tasks such as medical diagnosis, imputing missing features, augmenting datasets with artificially generated ones, estimating gene expression patterns, and many more. To be able to use ML algorithms on such datasets, a robust and efficient compute infrastructure is needed that can serve multiple purposes. They include preprocessing raw datasets to transform them into suitable formats that are compatible with ML algorithms, creating and executing their complex architectures on preprocessed datasets, and making trained models and predicted datasets readily available for further analyses. To facilitate such tasks, a complete infrastructure is developed that combines JupyterLab [3], augmented with many useful features, running on the public compute infrastructure of Galaxy [4] Europe to perform end-to-end AI analyses on scientific datasets. The infrastructure consists of 3 major components: first, a Docker container [5] that encapsulates JupyterLab together with multiple packages and plugins used for developing AI programs, data manipulation, and visualization (section S2 in the supplementary file lists all such packages and plugins with their respective versions); second, a Galaxy interactive tool [6, 7] that downloads this Docker container to serve JupyterLab on Galaxy Europe; and third, the compute infrastructure [8] of Galaxy Europe and the de.NBI cloud [9].

### Docker container

Docker [10] containers are popular for shipping packaged software as complete ecosystems, enabling them to be reproducible in a platform-independent manner. Software executing inside a Docker container is abstracted from the host operating system (OS) as most of the requirements necessary for them to run successfully are already configured inside its container. A container runs in an isolated environment having minimal interactions with the host OS. Therefore, running software in a container is more secure. Using a Docker container leverages the security benefits necessary for online program editing software executing arbitrary code. Arbitrary code may contain some malicious script, posing security risks. Using Docker containers can minimize their consequences. Further, in our Docker container, a nonroot user is created that can execute and manage projects inside the JupyterLab environment, which further minimizes security risks. In addition to minimizing security risks, Docker containers provide performance benefits compared to running programs on a virtual machine [11]. Motivated by such benefits, a Docker container is used in this project to encapsulate JupyterLab along with many useful packages such as Git [12], Elyra AI [13], TensorFlow-GPU [14], Scikit-learn [15], ONNX [16], and many others. The Docker container inherits many packages such as CUDA [17], NumPy [18], SciPy [19], and a few more from its base container, nvidia/cuda [20], and augments them with many other packages suitable for ML, data manipulation, and visualization. The Docker container is decoupled from Galaxy and can independently be executed for serving JupyterLab with the same set of packages on a different compute infrastructure or any personal computer or laptop having approximately 25 GB of disk space. Moreover, the Docker container is easily extended by adding the names of packages to the dockerfile [21]. Adding new packages requires the container to be rebuilt and added to Docker hub [22]. The approach to extending the Docker container is discussed in the Methods section.

### JupyterLab

JupyterLab is a web-based, robust editor used for varied purposes such as data science, scientific computing, and ML. It is a program editor that supports more than 40 programming languages, including Python, R, Julia, and Scala. Python is one of the most popular languages used by researchers for performing numerous scientific and predictive analyses. Therefore, it is used as the programming language in Galaxy’s JupyterLab because many popular packages such as Scikit-learn and TensorFlow for ML, data manipulation packages such as Pandas, and visualization packages such as Seaborn [23], Matplotlib [24], Bokeh [25], and many others are readily available as Python packages. Moreover, the extensible architecture of JupyterLab makes it possible to add many external packages as its plugins such as Git, Elyra AI, dashboards, and many others that have a user interface as necessary components. Such editors, integrated with several useful packages, provide a favorable platform for rapid prototyping and end-to-end development and management of AI projects. To harness the benefits of JupyterLab, it is used as the editor for the interactive tool in Galaxy.

### Features of JupyterLab infrastructure

**Figure 1:**
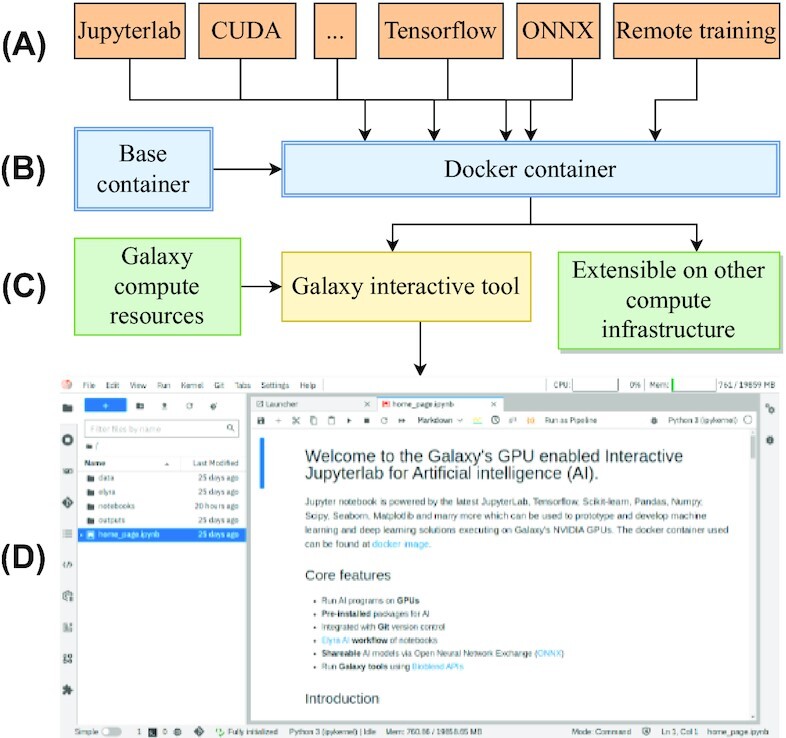
Architecture of Galaxy’s JupyterLab. (A) Packages and features wrapped inside a Docker container. (B) A base Docker container [41] from which the customized container [5] is derived. (C) Galaxy’s interactive tool downloads the customized container. The customized Docker container can also be hosted on a different compute infrastructure. (D) Galaxy’s JupyterLab.

Many features such as easy accessibility, support of a wide variety of programming languages on JupyterLab, and extensibility to install useful plugins make it a desirable editor for researchers for creating project prototypes rapidly. Many such features have been integrated into our JupyterLab infrastructure that is served online on Galaxy Europe, enabling researchers to create prototypes and end-to-end artificial intelligence (AI) projects (Fig. 1). A few important features are discussed here. To allow GPU computation from JupyterLab, TensorFlow-GPU interacts with Nvidia GPU hardware using another software, CUDA, when the compute resource has GPU(s) for accelerating ML programs. Faster execution of ML programs is one of the significant features of JupyterLab hosted on Galaxy Europe. However, if the hosted machine on which a Docker container runs does not have GPUs, then the program in JupyterLab relies on CPU cores. Other useful features include ONNX for transforming trained TensorFlow and Scikit-learn models to ONNX models; Open-CV [26] and Scikit-image [27] for processing images; Nibabel [28] for reading image files stored as “.nii”; Bioblend [29] for accessing Galaxy’s datasets, histories, and workflows in a JupyterLab notebook; visualization packages such as Bqplot [30] and Bokeh for plotting interactive charts; Voilà [31] for displaying output cells of a JupyterLab notebook in a different tab; and dashboard such as NVDashboard [32] for monitoring GPU usage and performance. Support for file extension such as H5 [33], efficient for storing matrices, enables ML researchers to save model weights and input datasets for AI algorithms. Other packages such as ColabFold [34] together with JAX [35] are used for predicting 3-dimensional (3D) structures of proteins, which are discussed in the Results section. In addition, it is possible to create a long-running training job that runs remotely and stores trained models and output datasets permanently in a newly created Galaxy history. The trained model is saved as an ONNX file, and tabular datasets are in an H5 file. It is discussed in the Methods section.

### Related infrastructure

There are a few other infrastructures available, free and commercial, that offer JupyterLab or similar environments for developing data science and AI projects. A few popular ones are Google Colab [36], Kaggle Kernel [37], and Amazon Sagemaker [38]. Google Colab is partially free and offers an online editor similar to JupyterLab. The free version of Google Colab offers dynamic compute resources. The disk space is around 70 GB and the memory (RAM) is around 12 GB. AI projects that deal with high-dimensional scientific data [39, 40] may require more resources. In addition, these resources offered by Google Colab are variable and depend on a user’s past usage. More compute resources are assigned to those users that have used less in the past for a more equitable sharing of resources. Moreover, there is a limitation of only 12 hours of running time, which may be inadequate for training AI models on large datasets needing longer running time. However, Google Colab pro and pro+ offer better compute resources, but they come at a price; EUR 9.25 and EUR 42.25 per month, respectively. In contrast, Kaggle Kernel is free of charge, but its computing resources are comparable to Colab. The total disk space is approximately 73 GB and RAM is 16 GB for a CPU-based kernel. For the GPU-based kernel, the disk space is of the same size as that of the CPU-based kernel, but the RAM of the CPU decreases to 13 GB. An additional RAM of 15 GB is added through a GPU, and computation time is limited to 30 hours a week. It also supports tensor processing unit (TPU), but the computation time is further limited to only 20 hours a week. Amazon Sagemaker is also a commercial software for developing AI algorithms that is free of charge but only for 2 months. Overall, these notebook infrastructures do not offer unrestricted compute resources free of charge. In addition, compute resources offered free of charge can be insufficient for training AI models on high-dimensional scientific datasets. To address the drawbacks of these notebook infrastructures and provide researchers and users with large compute resources more reliably, Galaxy JupyterLab infrastructure [8] offers an unlimited computation time on GPU and many CPU cores for each session as shown in Table 1. The offered resources for JupyterLab running in Galaxy stay constant and are independent of the user’s past usage. To make it more useful, JupyterLab opens a tab for each notebook that allows researchers to develop and execute several notebooks inside the same session of the allotted compute resource rather than having them connect to a different session for each notebook as in Google Colab and Kaggle Kernel.

**Table 1.** Comparison of Galaxy JupyterLab with other notebook infrastructures

Indicators/infrastructures	Google Colab [36]	Kaggle Kernel [37]	Galaxy JupyterLab
Memory/disk space (GB)	12/70	16/73	**20**/**250**
GPU/TPU	Yes/**Yes**	Yes/**Yes**	**Yes**/No
Max usage time (hours)	12	12, 30 hours of GPU/week, 20	**No time restriction** on GPU
		hours of TPU/week	and CPU cores usage,
			notebook sessions, and job execution
Dynamic compute resources	Yes	Yes	**Fixed and guaranteed**
Remote model training	No	No	**Yes**
Run multiple notebooks (as tabs) in 1 session	No	No	**Yes**

## Implementation

JupyterLab infrastructure is developed in 2 stages. First, a Docker container is created containing all the necessary packages such as JupyterLab itself, CUDA from the base Docker image [41], TensorFlow, Scikit-learn, ONNX, and many more. The Docker container is inherited from a base container that has all the necessary CUDA packages installed for working with NVIDIA GPUs. Many packages are added to the Docker container with their compatible versions. Compatible packages for CUDA, CUDA DNN, and TensorFlow are necessary so that they together interact with the GPU on the host machine for accelerating ML programs. The versions of all packages installed in the Docker container are listed in Supplementary Section S5. Second, the container can be downloaded to any powerful compute infrastructure, and JupyterLab can be served in an internet browser via the URL that it generates. In addition, to run this container in Galaxy, an interactive tool is created that downloads this container on a remote compute infrastructure and generates a URL used to run JupyterLab in an internet browser. The architecture of JupyterLab infrastructure in Galaxy is shown in Fig. 1. The running instance of JupyterLab in Galaxy contains a default IPython notebook that summarizes several of its features. Further, there are other notebooks available, each describing a feature of JupyterLab with code examples such as how to create ONNX models for Scikit-learn and TensorFlow classifiers, how to connect to Galaxy using Bioblend, how to create interactive plots using Bqplots, and how to create a pipeline of notebooks using Elyra AI. In addition, the notebooks explaining the use-cases are also available in the Docker container. To access JupyterLab in Galaxy Europe, a ready-to-use hands-on Galaxy training network (GTN) [42] tutorial [43] has been developed that shows all the steps such as opening the notebook, using Git to clone a code repository from GitHub, sending long-running training jobs to a remote Galaxy cluster, and how this notebook can be used as a tool in a Galaxy workflow. The approach of remote model training is explained in the Methods section. The 2 use-cases are also discussed in the tutorial along with their respective notebooks. The steps to access this resource on Galaxy Europe are elaborated in Supplementary Section S1.

## Results

JupyterLab infrastructure in Galaxy Europe is used to reproduce the results of 2 recent scientific publications. They demonstrate its robustness and usefulness to develop ML models using COVID-19 CT scan images [44] and predict the 3D structure of proteins using ColabFold, a faster implementation of AlphaFold2 [45].

### COVID-19 CT scan image segmentation

**Figure 2:**
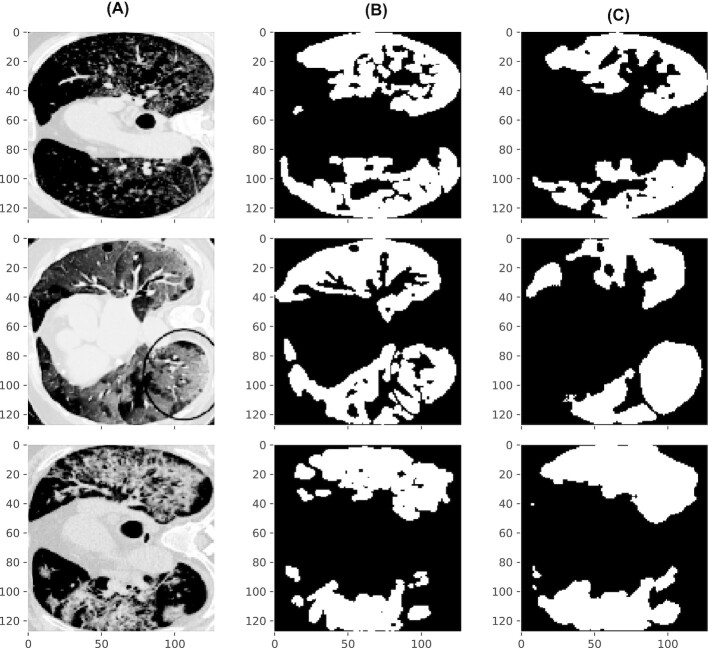
Original CT scan images (A), corresponding ground-truth masks of original CT scan images (B), and the predicted masks (C). Masks are COVID-19 infected regions in the corresponding CT scan images. The ground-truth and predicted masks show high similarity [44].

In [44], COVID-19 CT scan images have been used to develop and train an ML model architecture that predicts COVID-19 infected regions in those images with high accuracy. An open-source implementation of the work is available that trains a Unet deep learning architecture [46] distinguishing between normal and infected regions in CT scan images. Scripts of this implementation are adapted and executed on Galaxy’s JupyterLab infrastructure. Adaption only involves the transformation of all CT scan images, used in [44], into an H5 file so that they can directly be used as an input to the Unet architecture defined in a notebook available in [47]. All the notebooks available in [47] are also available in the Docker container in the “usecases” directory. A composite H5 file [48] is created using a script [49] that contains multiple datasets inside, and each dataset is a real-valued matrix corresponding to the training, test, and validation sets as used in [44]. The entire analysis of [44] can be reproduced using multiple notebooks in [47]. They achieve similar precision and recall (approximately 0.98) metrics, as mentioned in [44]. In [47], the first notebook (1_fetch_datasets.ipynb) downloads the input dataset as an H5 file. Additionally, it also downloads the trained ONNX model. The second notebook (2_create_model_and_train.ipynb) creates and trains a Unet model on the training dataset extracted from the H5 file. Training, accelerated by GPU, for 10 iterations over the entire training dataset finishes in a few minutes. The third notebook (3_predict_masks.ipynb) extracts the test dataset and predicts infected regions of the CT scan images in the test dataset using the trained model created by the second notebook. Fig. 2 shows the comparison of ground-truth infected regions in the second column and the predicted infected regions in the third column. A few original CT scan images from the test dataset are shown in the first column of Fig. 2.

#### Predict 3D structure of proteins using ColabFold

**Figure 3:**
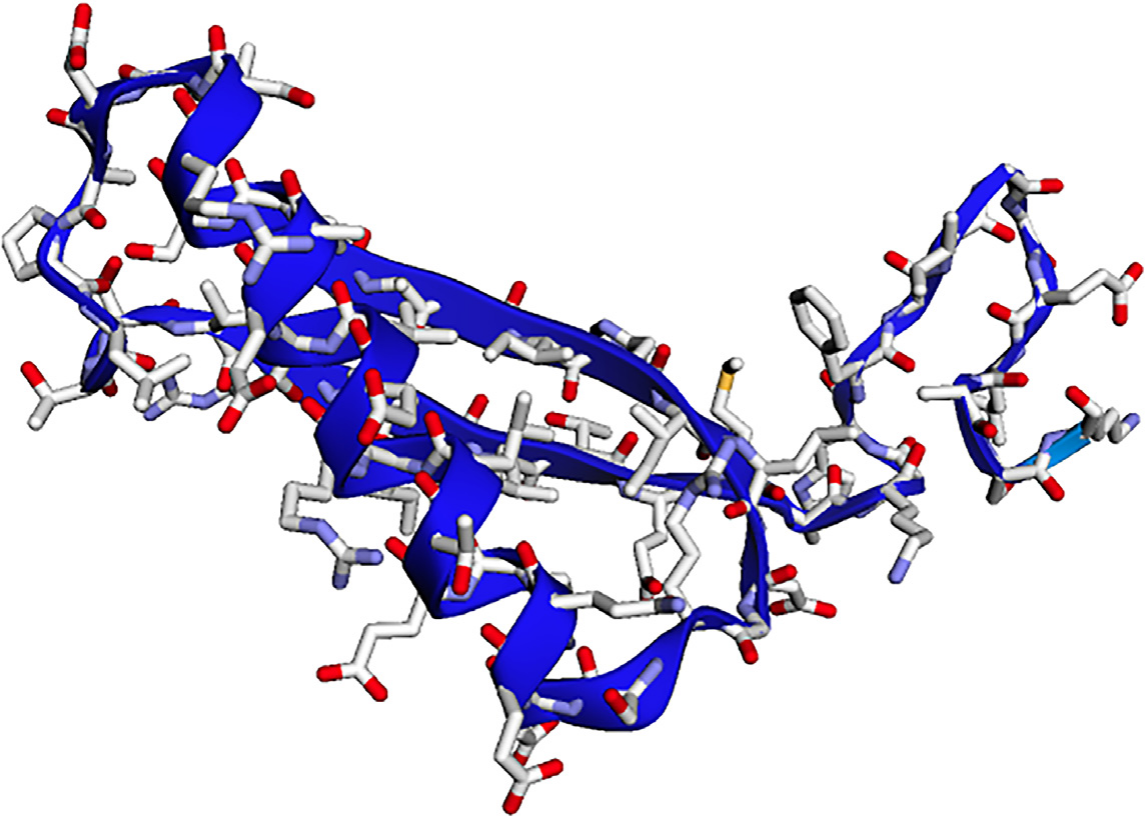
Figures shows a 3D structure of 4-oxalocrotonate tautomerase enzyme (protein) [51] predicted by ColabFold.

AlphaFold2 has made a breakthrough in predicting the 3D structures of proteins with outstanding accuracy. However, due to their large database size (a few TBs), it is not easily accessible to researchers. Therefore, a few approaches have been developed to replace the time-consuming steps of AlphaFold2 with slightly different steps. They predict 3D structures of proteins with similar accuracy while consuming less memory and time. One such approach is ColabFold, which replaces the large database search in AlphaFold2 for finding homologous sequences by a significantly faster (40–60 times) MMseqs2 API [50] call to generate input features based on the query protein sequence. ColabFold’s prediction of 3D structures in batches is approximately 90 times faster. It is integrated into the Docker container [5] by adding 2 packages: ColabFold and GPU-enabled JAX, which is a just-in-time compiler for making mathematical transformations. Notebook “7_ColabFold_MMseq2.ipynb” in [47] predicts the 3D structure of a protein sequence using ColabFold by making use of the pretrained weights of AlphaFold2. Fig. 3 shows the 3D structure of 4-oxalocrotonate tautomerase [51], a protein sequence of length 62, along with its side chains. This 3D structure is extremely similar to the structure predicted by the JupyterLab notebook [52] from ColabFold [34].

## Methods

### Remote model training

For large datasets, ML model training may need several hours or even days. In such cases, it would be cumbersome to keep JupyterLab open in a browser’s tab until the training finishes. Therefore, another Galaxy tool [53] is developed to enable researchers to send long-running training jobs to a remote Galaxy cluster. The tool can be executed from JupyterLab using a custom Python function [54], part of each JupyterLab notebook, that takes input datasets and a training script as input parameters. The input datasets to be used for training, testing, and validation must be provided in H5 format. It allows the standardization of input data format for AI models that train on matrices in JupyterLab. Input data to an AI model can be in multiple formats such as images, genomic sequences, or gene expression patterns. H5 files can be created using any of these data formats and fed to the AI model in JupyterLab. Long-running training happens in a remote Galaxy cluster as a regular Galaxy job. Upon completion of the job, the resulting datasets and the trained model become available in a newly created Galaxy history [55]. The trained model and other resulting datasets can either be downloaded to a local machine or imported from the Galaxy history for further analysis using “get” (for fetching datasets directly into a JupyterLab notebook from Galaxy history) and “put” (for saving datasets directly into a Galaxy history from JupyterLab notebook) methods into a JupyterLab notebook [56]. In [47], a few notebooks are available that showcase the approach of remote model training. Notebook “4_create_model_and_train_remote.ipynb” contains code for developing and training a Unet architecture. Notebook “5_run_remote_training.ipynb” executes the previous notebook on a Galaxy cluster remotely after creating a Galaxy history and then uploading the script extracted from “4_create_model_and_train_remote.ipynb” notebook and input datasets. Custom Python function, “run_script_job,” creates a Galaxy history using Bioblend and then uploads the datasets to the same history. After the upload is finished, the Python script from the specified notebook is executed dynamically. It trains an ML architecture on the uploaded datasets to create a model and saves it as an ONNX file in the Galaxy history. Using “6_predict_masks_remote_model.ipynb” notebook from [47], the trained model can be downloaded from the Galaxy history and used for predicting infected regions of the CT scan images from the test dataset. A significant advantage of training ML models remotely is that researchers do not have to keep the JupyterLab session running as long as the model is being trained as the model training becomes decoupled from JupyterLab. Using such a feature, ML models that take several hours or even days to train can be conveniently trained.

### Extend Docker container

The customized Docker container developed as shown in Fig. 1 can be easily extended to have more or different packages. To update the container, a package or a list of new packages should be added to the dockerfile and then the new container should be built and pushed to Docker hub [5]. After pushing the new container, when Galaxy’s JupyterLab interactive tool is accessed on Galaxy Europe, it downloads the new container, and all the newly added packages are available in JupyterLab. Similarly, versions of existing packages can be updated or existing packages can be removed if no longer needed. The simplified extension procedure of the entire infrastructure incurs low maintenance costs as any change to this entire infrastructure is reflected only in the container without updating Galaxy’s codebase. In addition, packages can also be added or updated using “pip” in any JupyterLab notebook, but such changes remain as long as the JupyterLab session runs as they do not update the underlying Docker container.

### Collaborative notebooks

Notebooks created in Galaxy’s JupyterLab infrastructure can instantly be shared with other researchers and collaborators only by sharing the public URL of a notebook. Researchers and users who share a notebook can collaborate on the same notebook without having to store it anywhere as it is directly served by Galaxy Europe.

### Workflow of notebooks

Resembling many tools in Galaxy, JupyterLab can also be used in any Galaxy workflow where it can accept datasets from different tools and then executes an IPython notebook to process the input datasets. It outputs a collection of datasets, which can further be used by other Galaxy tools [43]. In addition, using the Elyra AI package, a workflow of notebooks can be created using existing notebooks in a JupyterLab session and executed as one unit of software similarly as Galaxy workflows are created using several tools. It is possible to execute such workflows of notebooks on the same compute resource on which the JupyterLab session runs. In addition, a few other services such as Kubeflow [57] or Apache Airflow [58] can also be used to deploy, run, and manage such workflows on a cloud but are not explored in our work.

## Summary

JupyterLab is integrated as an interactive tool in Galaxy Europe running on a public and powerful compute infrastructure comprising several CPU cores and GPUs having large memory and disk space. A Docker container is created that wraps JupyterLab along with packages such as TensorFlow-GPU, Scikit-learn, Pandas, and many others to provide a robust architecture for the development and management of projects in ML and data science. Remote model training makes it convenient to run multiple analyses in parallel in different Galaxy jobs by executing the same Galaxy tool. The resulting datasets of each job become available in different Galaxy histories. Features such as Git integration are useful for managing entire code repositories on GitHub and Elyra AI for creating pipelines of notebooks working as one software unit. All notebooks created by a user run on the same session of JupyterLab in different tabs. The entire infrastructure of JupyterLab is readily accessible through Galaxy Europe. In contrast to commercial infrastructures that host editors similar to JupyterLab and offer powerful and reliable compute only through paid subscriptions, this infrastructure provides large compute resources free of cost that are invariant to usage and has an unlimited usage time while ensuring a constant amount of compute resources across successive usages. Sustaining and improving such an openly accessible infrastructure would highly benefit ML practitioners and researchers from various fields of science.

## Availability of Supporting Source Code and Requirements

Project name: GPU-enabled Docker container with JupyterLab for artificial intelligence

Project home page: https://github.com/usegalaxy-eu/gpu-jupyterlab-docker

Galaxy interactive tool: https://github.com/usegalaxy-eu/galaxy/blob/release_22.05_europe/tools/interactive/interactivetool_ml_jupyter_notebook.xml

Operating system: Linux

Programming languages: Python, XML, Docker, Bash

License: MIT License

RRID: SCR_022695

Biotools ID: gpu-enabled_docker_container_with_jupyterlab_for_ai

## Supplementary Material

giad028_GIGA-D-22-00220_Original_Submission

giad028_GIGA-D-22-00220_Revision_1

giad028_GIGA-D-22-00220_Revision_2

giad028_Response_to_Reviewer_Comments_Original_Submission

giad028_Response_to_Reviewer_Comments_Revision_1

giad028_Reviewer_1_Report_Original_Submission Philippe Boileau -- 9/8/2022 Reviewed

giad028_Reviewer_1_Report_Revision_1 Philippe Boileau -- 2/2/2023 Reviewed

giad028_Reviewer_2_Report_Original_Submission Milot Mirdita -- 10/26/2022 Reviewed

giad028_Reviewer_2_Report_Revision_1 Milot Mirdita -- 2/16/2023 Reviewed

giad028_Supplemental_File

## Data Availability

All supporting data and materials are available in the *GigaScience* GigaDB database [59].

## Additional Files

Supplementary Material: An accessible infrastructure for artificial intelligence using a Docker-based JupyterLab in Galaxy.

## Abbreviations

AI: artificial intelligence; CT: computed tomography; CUDA: Compute Unified Device Architecture; EUR: Euro; GPU: graphical processing unit; GB: gigabyte; GTN: Galaxy training network; JAX: accelerated linear algebra; ML: machine learning; ONNX: Open Neural Network Exchange; OS: operating system; RAM: random-access memory; PB: petabyte; TB: terabyte; URL: uniform resource locator; 3D: 3-dimensional.

## References

[1] Pearson W, Crusoe M, et al. The FASTA package—protein and DNA sequence similarity searching and alignment programs. GitHub. 2016. https://github.com/wrpearson/fasta36. [Accessed June 30, 2022].

[2] Kumar I, Singh SP, Shivam. Machine learning in bioinformatics. Bioinformatics, Dev BS and Pathak RK, Academic Press; Dehradun 2022:443–56.. https://www.sciencedirect.com/science/article/pii/B9780323897754000201.

[3] Kluyver T, Ragan-Kelley B, Pérez F, Granger B, Bussonnier M, et al. Jupyter Notebooks—A Publishing Format for Reproducible Computational Workflows. IOS Press; Amsterdam. 2016:87.

[4] The Galaxy Community . The Galaxy platform for accessible, reproducible and collaborative biomedical analyses: 2022 update. Nucleic Acids Res. 2022;50(W1):W345–51. 35446428 10.1093/nar/gkac247 PMC9252830

[5] Kumar A. Container for machine learning and deep learning in Jupyter notebook. Docker. 2021. https://hub.docker.com/r/anupkumar/docker-ml-jupyterlab. [Accessed June 29, 2022]

[6] Galaxy Europe . Live instance of the European Galaxy server. Galaxy Europe. 2019. https://live.usegalaxy.eu/. [Accessed June 30, 2022]

[7] Kumar A, Grüning B. GPU enabled interactive Jupyter notebook for machine learning. GitHub 2021. https://github.com/usegalaxy-eu/galaxy/blob/release_22.05_europe/tools/interactive/interactivetool_ml_jupyter_notebook.xml. [Accessed April 24, 2023]

[8] Compute resources in Galaxy Europe . GitHub. 2023. https://galaxyproject.org/news/2023-01-24-gpu-jupyterlab-galaxy/#current-resources-will-be-updated-regularly.[Accessed April 24, 2023]

[9] German Network for Bioinformatics Infrastructure . de.NBI. 2015. https://www.denbi.de/cloud. [Accessed April 24, 2023]

[10] Merkel D. Docker: lightweight linux containers for consistent development and deployment. Linux J. 2014;2014(239):2.

[11] Baset S, Berger S, Bottomley J, et al. Docker and container security white paper. 2016. https://dominoweb.draco.res.ibm.com/reports/rc25625.pdf.[Accessed April 24, 2023]

[12] Collonval F, Rheines A, Zhang J, et al. A JupyterLab extension for version control using Git. https://github.com/jupyterlab/jupyterlab-git. [Accessed June 29, 2022]

[13] Resende L, Chin A, Titzler P, et al. Elyra is a set of AI-centric extensions to JupyterLab notebooks. 2018.https://github.com/elyra-ai/elyra. [Accessed June 29, 2022].

[14] Abadi M, Agarwal A, Barham P, et al. TensorFlow: large-scale machine learning on heterogeneous systems. 2015. https://www.tensorflow.org/.[Accessed 24 April 2023]

[15] Pedregosa F, Varoquaux G, Gramfort A, et al. Scikit-learn: machine learning in Python. J Machine Learn Res. 2011;12:2825–30.

[16] Bai J, Lu F, Zhang K, et al. ONNX: Open Neural Network Exchange. GitHub. 2019. https://github.com/onnx/onnx.[Accessed June 29, 2022].

[17] NVIDIA , Vingelmann P, Fitzek FHP. CUDA, release: 10.2.89. 2020. https://developer.nvidia.com/cuda-toolkit. [Accessed June 29, 2022]

[18] Harris CR, Millman KJ, van der Walt SJ, et al. Array programming with NumPy. Nature. 2020;585(7825):357–62. 32939066 10.1038/s41586-020-2649-2 PMC7759461

[19] Virtanen P. SciPy 1 0 Contributors. SciPy 1.0: fundamental algorithms for scientific computing in Python. Nat Methods. 2020;17:261–72. 32015543 10.1038/s41592-019-0686-2 PMC7056644

[20] NVIDIA Corporation . CUDA and cuDNN images from gitlab.com/nvidia/cuda. Docker. 2014. https://hub.docker.com/r/nvidia/cuda. [Accessed June 29, 2022].

[21] Kumar A. Jupyter container used for Data Science and Tensorflow. GitHub. 2021. https://github.com/anuprulez/ml-jupyter-notebook/blob/master/Dockerfile.[ Accessed June 29, 2022].

[22] Docker Hub . Docker. 2013. https://hub.docker.com/

[23] Waskom ML. Seaborn: statistical data visualization. J Open Source Softw. 2021;6(60):3021

[24] Hunter JD. Matplotlib: a 2D graphics environment. IEEE Comput Soc. 2007.9(3):90–95.

[25] Bokeh Development Team . Bokeh: Python library for interactive visualization. GitHub. 2018. https://bokeh.pydata.org/en/latest/. [Accessed April 24, 2023]

[26] Alekhin A, Lavrenov I, Shabunin M, et al. OpenCV: Open Source Computer Vision Library. GitHub 2012.; https://github.com/opencv/opencv. [Accessed April 24, 2023]

[27] Van der Walt S, Schönberger JL, Nunez-Iglesias J, et al. scikit-image: image processing in Python. PeerJ. 2014;2:e453. 25024921 10.7717/peerj.453 PMC4081273

[28] Brett M, Markiewicz CJ, Hanke M, et al. nipy/nibabel: 3.2.2. *Zenodo*. 2022. 10.5281/zenodo.6617121.

[29] Sloggett C, Goonasekera N, Afgan E. BioBlend: automating pipeline analyses within Galaxy and CloudMan. Bioinformatics. 2013;29(13):1685–6. 23630176 10.1093/bioinformatics/btt199 PMC4288140

[30] Corlay S, Cherukuri C, Renou M, et al. 2-D plotting library for Project Jupyter. GitHub. 2015. https://github.com/bqplot/bqplot. [Accessed June 29, 2022]

[31] Tuloup J, Breddels M, Corlay S, et al. Rendering of live Jupyter notebooks with interactive widgets. GitHub. 2018. https://github.com/voila-dashboards/voila..[Accessed 29 June 2022]

[32] Tomlinson J, Schmidt AJ, Zamora R, et al. A JupyterLab extension for displaying GPU usage dashboards. GitHub. 2021. https://github.com/rapidsai/jupyterlab-nvdashboard. [Accessed June 29, 2022].

[33] The HDF Group . Hierarchical Data Format, version 5; 1997–2022. https://www.hdfgroup.org/HDF5/. [Accessed June 29, 2022]

[34] Mirdita M, Schütze K, Moriwaki Y et al. ColabFold: making protein folding accessible to all. Nat Methods. 2022;19:679–82. 35637307 10.1038/s41592-022-01488-1 PMC9184281

[35] Johnson M, Hawkins P, Vanderplas J, et al. JAX: Autograd and XLA. 2020. https://github.com/google/jax. [Accessed June 29, 2022]

[36] Bisong E. Google Colaboratory. 2019. 10.1007/978-1-4842-4470-8_7.[Accessed April 24, 2023]

[37] Kaggle. Kaggle . 2020. https://www.kaggle.com. [Accessed June 29, 2022]

[38] Amazon SageMaker . Amazon SageMaker. 2017. https://aws.amazon.com/sagemaker/. [Accessed June 29, 2022]

[39] Moon KR, van Dijk D, Wang Z. Visualizing structure and transitions in high-dimensional biological data. Nat Biotechnol. 2019;37:1482–92. 31796933 10.1038/s41587-019-0336-3 PMC7073148

[40] Boileau P, Hejazi NS, Dudoit S. Exploring high-dimensional biological data with sparse contrastive principal component analysis. Bioinformatics. 2020;36(11):3422–30. 32176249 10.1093/bioinformatics/btaa176

[41] Nvidia/Docker. nvidia/cuda:11.8.0-cudnn8-runtime-ubuntu20.04. 2014. https://hub.docker.com/r/nvidia/cuda/tags?page=1&name=11.8.0-cudnn8-runtime-ubuntu20.04. [Accessed April 24, 2023]

[42] Batut B, Hiltemann S, Bagnacani A, et al. Community-driven data analysis training for biology. Cell Systems. 2018;6(6):752–8. 29953864 10.1016/j.cels.2018.05.012 PMC6296361

[43] Kumar A. A Docker-based interactive Jupyterlab powered by GPU for artificial intelligence in Galaxy (Galaxy Training Materials).. 2022. https://training.galaxyproject.org/training-material/topics/statistics/tutorials/gpu_jupyter_lab/tutorial.html. [Accessed January 23, 2023]

[44] Saeedizadeh N, Minaee S, Kafieh R, et al. COVID TV-Unet: segmenting COVID-19 chest CT images using connectivity imposed Unet. Comput Methods Programs Biomed Update. 2021;1:100007 34337587 10.1016/j.cmpbup.2021.100007 PMC8056883

[45] Jumper J, Evans R, Pritzel A et al. Highly accurate protein structure prediction with AlphaFold. Nature. 2021;596:583–9. 34265844 10.1038/s41586-021-03819-2 PMC8371605

[46] Ronneberger O, Fischer P, Brox T. U-Net: convolutional networks for biomedical image segmentation. Medical Image Computing and Computer-Assisted Intervention – MICCAI 2015. Springer, Cham 2015;9351. 234–-241. https://doi.org/10.1007/978-3-319-24574-4_28.

[47] Kumar A. Jupyterlab notebooks. GitHub. 2022. https://github.com/anuprulez/gpu_jupyterlab_ct_image_segmentation. [Accessed June 29, 2022]

[48] Kumar A. COVID Image segmentation datasets and trained model. Zenodo.. 2022. 10.5281/zenodo.6091361.[Accessed April 24, 2023]

[49] Saeedizadeh N, Minaee S, Kafieh R, et al. COVID TV-Unet: Segmenting COVID-19 chest CT images using connectivity imposed Unet. GitHub. 2021. https://github.com/narges-sa/COVID-CT-Segmentation/blob/main/main_TV_Unet_Split1.py. [Accessed June 30, 2022] 10.1016/j.cmpbup.2021.100007 PMC8056883 34337587

[50] Steinegger M, Söding J. MMseqs2 enables sensitive protein sequence searching for the analysis of massive data sets. Nat Biotechnol. 2017;35:1026–8. 29035372 10.1038/nbt.3988

[51] Chen L, Kenyon G, Curtin F, et al. 4 Oxalocrotonate tautomerase, an enzyme composed of 62 amino acid residues per monomer. J Biol Chem. 1992;267(25):17716–21. 1339435

[52] Mirdita M, Schütze K, Moriwaki Y et al. ColabFold: making protein folding accessible to all. GitHub. 2022. https://github.com/sokrypton/ColabFold/blob/main/AlphaFold2.ipynb. [Accessed June 30, 2022] 10.1038/s41592-022-01488-1 PMC9184281 35637307

[53] Kumar A. Run long-running jupyterlab script. Github.. 2022. https://github.com/bgruening/galaxytools/blob/master/tools/jupyter_job/run_jupyter_job.xml. [Accessed June 30, 2022]

[54] Kumar A. Custom jupyterlab notebook function to start model training job in Galaxy. Github. 2021. https://github.com/anuprulez/ml-jupyter-notebook/blob/master/galaxy_script_job.py#L43. [Accessed June 30, 2022]

[55] Kumar A. Remotely trained image segmentation model. Galaxy. 2022. https://usegalaxy.eu/u/kumara/h/image-segmentation-from-galaxy-jupyterlab. [Accessed August 23, 2022]

[56] Galaxy’s Interactive Environments. GitHub. 2016. https://github.com/bgruening/docker-jupyter-notebook/blob/master/default_notebook.ipynb.[Accessed April 24, 2023]

[57] Kubeflow . GitHub. 2017. https://github.com/kubeflow/kubeflow.[Accessed April 24, 2023]

[58] Apache Airflow . GitHub. 2019. https://github.com/apache/airflow-site.[Accessed April 24 2023]

[59] Kumar A, Cuccuru G, Gruening B, et al. Supporting data for “An Accessible Infrastructure for Artificial Intelligence Using a Docker-Based JupyterLab in Galaxy.” GigaScience Database. 2023. 10.5524/102381. PMC10132306 37099385

