# Supplementary material for: An accessible infrastructure for artificial intelligence using a Docker-based JupyterLab in Galaxy
Source: Gigascience. 2023 Apr 26;12:giad028. doi: 10.1093/gigascience/giad028 (PMC10132306; doi:10.1093/gigascience/giad028)
Supplement: giad028_GIGA-D-22-00220_Original_Submission [file giad028_giga-d-22-00220_original_submission.pdf]

GigaScience

An accessible infrastructure for artificial intelligence using a docker-based Jupyterlab in Galaxy

--Manuscript Draft--

**Manuscript Number:**	GIGA-D-22-00220
**Full Title:**	An accessible infrastructure for artificial intelligence using a docker-based Jupyterlab in Galaxy
**Article Type:**	Technical Note
**Funding Information:**	DFG (German Research Foundation) (CIBSS - EXC-2189 - Project ID 390939984) — Dr Rolf Backofen<br>BMBF grant (de.NBI) (031A538A) — Dr Björn Grüning
**Abstract:**	Background Artificial intelligence (AI) programs that train on a large amount of data require powerful compute infrastructure. Jupyterlab notebook provides an excellent framework for developing AI programs but it needs to be hosted on a powerful infrastructure to enable AI programs to train on large data.<br>Findings An open-source, docker-based, and GPU-enabled jupyterlab notebook infrastructure is developed that runs on the public compute infrastructure of Galaxy Europe to rapidly prototype and develop end-to-end AI projects. Using such a notebook, long-running AI model training programs can be executed remotely to create trained models, represented in open neural network exchange (ONNX) format, and other output datasets in Galaxy. Other features include GPU support for faster training, git integration for version control, the option of creating and executing pipelines of notebooks, and multiple dashboards for monitoring compute resources and packages for visualisation.<br>Conclusions These features make the jupyterlab notebook highly suitable for creating and managing AI projects. A recent scientific publication that predicts infected regions in COVID-19 CT scan images is reproduced using various features of this notebook. In addition, colabfold, a faster implementation of alphafold2, is accessed in this notebook to predict the 3D structure of protein sequences. Jupyterlab notebook is accessible in two ways - one as an interactive Galaxy tool and the other by running the underlying docker container. In both ways, long-running training can be executed on Galaxy's compute infrastructure. Scripts to create the docker container are available under MIT license at https://github.com/anuprulez/ml-jupyter-notebook .
**Corresponding Author:**	Anup Kumar<br>Albert-Ludwigs-Universitat Freiburg<br>Freiburg, GERMANY
**Corresponding Author Secondary Information:**	
**Corresponding Author's Institution:**	Albert-Ludwigs-Universitat Freiburg
**Corresponding Author's Secondary Institution:**	
**First Author:**	Anup Kumar
**First Author Secondary Information:**	
**Order of Authors:**	Anup Kumar<br>Gianmauro Cuccuru<br>Björn Grüning<br>Rolf Backofen
**Order of Authors Secondary Information:**	
**Additional Information:**	

Powered by Editorial Manager® and ProduXion Manager® from Aries Systems Corporation

Question	Response
Are you submitting this manuscript to a special series or article collection?	No
**Experimental design and statistics**<br><br>Full details of the experimental design and statistical methods used should be given in the Methods section, as detailed in our Minimum Standards Reporting Checklist. Information essential to interpreting the data presented should be made available in the figure legends.<br><br>Have you included all the information requested in your manuscript?	Yes
**Resources**<br><br>A description of all resources used, including antibodies, cell lines, animals and software tools, with enough information to allow them to be uniquely identified, should be included in the Methods section. Authors are strongly encouraged to cite Research Resource Identifiers (RRIDs) for antibodies, model organisms and tools, where possible.<br><br>Have you included the information requested as detailed in our Minimum Standards Reporting Checklist?	Yes
**Availability of data and materials**<br><br>All datasets and code on which the conclusions of the paper rely must be either included in your submission or deposited in publicly available repositories (where available and ethically appropriate), referencing such data using a unique identifier in the references and in the "Availability of Data and Materials" section of your manuscript.	Yes

Have you have met the above requirement as detailed in our Minimum Standards Reporting Checklist?	

Powered by Editorial Manager® and ProduXion Manager® from Aries Systems Corporation

Main text file Click here to access/download;LaTeX - Main Document (TeX file);main.tex

GigaScience, 0000, 1–7

doi: xx.xxxx/xxxx
Manuscript in Preparation
Technical Note

TECHNICAL NOTE

An accessible infrastructure for artificial intelligence using a docker-based Jupyterlab in Galaxy

Anup Kumar[1,*,†], Gianmauro Cuccuru[1, ‡, †], Björn Grüning[1, §, †] and Rolf Backofen[1,2, ¶, †]

[1]Bioinformatics Group, Department of Computer Science, University of Freiburg, Georges-Koehler-Allee 106, 79110 Freiburg, Germany and [2]Signalling Research Centres BIOSS and CIBSS, University of Freiburg, Schaenzlestr. 18, 79104 Freiburg, Germany

[*]kumara@informatik.uni-freiburg.de https://orcid.org/0000-0002-2068-4695
[‡]gmauro@gmail.com https://orcid.org/0000-0002-5335-545X
[§]gruening@informatik.uni-freiburg.de https://orcid.org/0000-0002-3079-6586
[¶]backofen@informatik.uni-freiburg.de https://orcid.org/0000-0001-8231-3323
[†]Contributions follow the order of the names of authors

Abstract

Background Artificial intelligence (AI) programs that train on a large amount of data require powerful compute infrastructure. Jupyterlab notebook provides an excellent framework for developing AI programs but it needs to be hosted on a powerful infrastructure to enable AI programs to train on large data. **Findings** An open-source, docker-based, and GPU-enabled jupyterlab notebook infrastructure is developed that runs on the public compute infrastructure of Galaxy Europe to rapidly prototype and develop end-to-end AI projects. Using such a notebook, long-running AI model training programs can be executed remotely to create trained models, represented in open neural network exchange (ONNX) format, and other output datasets in Galaxy. Other features include GPU support for faster training, git integration for version control, the option of creating and executing pipelines of notebooks, and multiple dashboards for monitoring compute resources and packages for visualisation. **Conclusions** These features make the jupyterlab notebook highly suitable for creating and managing AI projects. A recent scientific publication that predicts infected regions in COVID-19 CT scan images is reproduced using various features of this notebook. In addition, colabfold, a faster implementation of alphafold2, is accessed in this notebook to predict the 3D structure of protein sequences. Jupyterlab notebook is accessible in two ways - one as an interactive Galaxy tool and the other by running the underlying docker container. In both ways, long-running training can be executed on Galaxy's compute infrastructure. Scripts to create the docker container are available under MIT license at https://github.com/anuprulez/ml-jupyter-notebook.

Key words: Jupyterlab notebooks; Galaxy Europe; Artificial intelligence; Remote model training; ONNX; Elyra AI; GPU; CUDA;

Findings

Background

Bioinformatics comprises many sub-fields such as single-cell, medical imaging, sequencing, proteomics and many more that produce a huge amount of biological data in myriad formats.

Compiled on: August 24, 2022.
Draft manuscript prepared by the author.

For example, the single-cell field creates gene expression patterns for each cell that are represented as matrices of real numbers. The medical imaging field generates images of cells and tissues, radiography images such as chest x-rays and computerized tomography (CT) scans. Next-Generation sequencing generates deoxyribonucleic acid (DNA) sequences that are stored as fasta [1] files. Artificial intelligence (AI) approaches such as machine learning (ML) and deep learning (DL) have been vastly used with these datasets [2] for predictive tasks such as medical diagnosis, imputing missing features, augmenting datasets with artificially generated ones, and estimating gene expression patterns and many more. To be able to use ML and DL algorithms on such datasets, a robust and efficient compute infrastructure is needed that can serve multiple purposes. They include pre-processing raw datasets to transform them into suitable formats that are compatible with ML and DL algorithms, creating and executing their complex architectures on pre-processed datasets and making trained models and predicted datasets readily available for further analyses. To facilitate such tasks, a complete infrastructure is developed that combines jupyterlab [3] notebook, augmented with many useful features, running on public compute resources of Galaxy [4] Europe to perform end-to-end AI analyses on biological datasets. The infrastructure consists of three important components. First, a docker container [5] that encapsulates jupyterlab together with a large number of packages used for developing AI programs, data manipulation and visualisation. Section S2 in the supplementary file lists all the packages with their respective versions. Second, a Galaxy interactive tool [6, 7] that downloads this docker container to serve jupyterlab online on Galaxy Europe. Third, the compute infrastructure consisting of several CPUs and GPUs that acts as a backend on which the online jupyterlab runs.

Docker container

Docker [8] containers are popular for shipping packaged software as complete ecosystems enabling them to be reproducible in a platform-independent manner. Software executing inside a docker container is abstracted from the operating system (OS) as most of the requirements necessary for them to run successfully are already configured inside the container. A container runs as an isolated environment making the minimum number of interactions to the host OS and thereby, improving the security aspects of the running software. Using a docker container leverages the security benefits that are necessary to have for online program editor softwares executing arbitrary code. In addition to minimising security risks, docker containers provide performance benefits compared to running programs on a virtual machine [9]. Motivated by such benefits, a docker container is used in this project to encapsulate jupyterlab along with many useful packages such as git [10], elyra AI [11], tensorflow-GPU [12], scikit-learn [13], CUDA [14], ONNX [15], and many others. Docker container inherits many packages such as numpy [16], scipy [17] and a few more from its base container, jupyter/tensorflow-notebook [18], and augments them with many other packages suitable for ML and DL, data manipulation and visualisation. Docker container is decoupled from Galaxy and can independently be executed for serving jupyterlab with the same set of packages on a different compute infrastructure or any personal computer (PC) or laptop having approximately 25 gigabytes (GB) of disk space. Moreover, it can easily be extended by installing suitable packages only by adding their appropriate package names in its dockerfile [19]. The approach extending the docker container is discussed in Methods section in detail.

Jupyterlab

Jupyterlab is a web-based, robust editor used for varied purposes such as data science, scientific computing, machine learning and deep learning. It is a program editor that supports more than 40 programming languages, some of the popular ones are python, R, julia and scala. Python is one of the most popular languages used by researchers for performing numerous scientific and predictive analyses. Therefore, it is used as the programming language in Galaxy jupyterlab notebooks because many popular packages such as tensorflow, scikit-learn for ML and DL, data manipulation packages such as pandas, visualisation packages such as seaborn [20], matplotlib [21], bokeh [22] and many others are readily available as python packages. Moreover, the extensible architecture of jupyterlab makes it possible to add many external packages as jupyterlab plugins such as git, elyra AI, dashboards and many others that have a user interface (UI) as necessary components. Editors such as jupyterlab, integrated with several useful packages, provide a favourable platform for both, rapid prototyping and end-to-end development and management of AI projects. To harness the benefits of jupyterlab, it has been used as the editor for the Jupyterlab interactive tool in Galaxy.

Features of jupyterlab notebook infrastructure

Many features such as easy accessibility, support of a wide variety of programming languages on jupyterlab, and extensibility to install useful plugins make it a desirable editor for researchers for creating project prototypes rapidly. Many such features have been integrated into a jupyterlab notebook infrastructure that serves jupyterlab notebook online on Galaxy Europe running on large compute resources enabling researchers to create prototypes and end-to-end AI projects (Figure 1). A few important features are discussed here. To allow GPU computation from jupyterlab notebooks, tensorflow-GPU interacts with Nvidia GPU hardware using another software, CUDA, when the compute resource has GPU(s) for accelerating deep learning programs. Faster execution of deep learning programs is one of the significant features of jupyterlab hosted on Galaxy Europe. However, if the hosted machine on which a docker container runs does not have GPUs, then the program in a jupyterlab notebook relies on CPUs. Other useful features include ONNX for transforming trained tensorflow and scikit-learn models to ONNX models, open-CV [23] and scikit-image [24] for processing images, nibabel [25] for reading image files stored as ".nii", bioblend [26] for accessing Galaxy's datasets, histories and workflows in a jupyterlab notebook and visualisation packages such as bqplot [27] and bokeh for plotting interactive charts, voila [28] for displaying output cells of a jupyterlab notebook, dashboard such as nvdashboard [29] for monitoring GPU usage and performance. Support for the file extension such as H5 [30], efficient for storing matrices, enables machine learning researchers to save model weights and input datasets for AI algorithms. Other packages such as colabfold [31] together with JAX [32] are used for predicting 3D structures of proteins which are discussed in detail in the Results section. In addition, it is possible to create a long-running training job that runs remotely and stores trained models and output datasets permanently in a newly created Galaxy history. The trained model is saved as an ONNX file and tabular datasets in H5 file.

Related infrastructure

There are a few other infrastructures available, free and commercial, that offer jupyterlab or similar environments for de-

veloping data science and AI projects. A few popular ones are google colab [33], kaggle kernels [34] and amazon's sagemaker [35]. Google colab is partially free and offers an online editor similar to a jupyterlab notebook. The free version of colab offers dynamic compute resources. The disk space is around 70 GB and the memory (RAM) is around 12 GB. These resources are scarce for the AI projects that deal with high-dimensional biological data [36, 37]. In addition, these resources offered by colab are variable and depend on a user's past usage. More compute resources are assigned to those users that have used less in the past for a more equitable sharing of resources. Moreover, there is a limitation of only 12 hours of running time that may prove to be insufficient and non-ideal for training AI models on large datasets. However, colab pro and pro+ offer better compute resources but they come at a price, EUR 9.25 and EUR 42.25 per month, respectively. In contrast, kaggle kernels are free of charge but similar to colab, their computing resources are scarce. The total disk space is approximately 73 GB and RAM is 16 GB for a CPU-based kernel. For the GPU-based kernel, the disk space is of the same size as that of the CPU-based kernel but the RAM of the CPU decreases to 13 GB. An additional RAM of 15 GB is added through a GPU and computation time is limited to 30 hours a week. It also supports TPUs but the computation time is further limited to only 20 hours a week. Amazon's sagemaker is also a commercial software for developing AI algorithms that is free of charge but only for 2 months. Overall, these notebook infrastructures do not offer unrestricted compute resources free of charge. In addition, compute resources offered free of charge are insufficient for training AI models on high-dimensional biological datasets. To address the drawbacks of these notebook infrastructures and provide researchers and users large compute resources more reliably, Galaxy jupyterlab notebook infrastructure offers 1 terabyte (TB) of disk space and unlimited computation time on 1 GPU and 7 CPUs per session. RAM for GPU is around 15 GB and for CPUs is 20 GB (Table 1). The offered resources for the jupyterlab notebook running in Galaxy stay constant and are independent of the user's past usage. To make it more useful, jupyterlab opens a tab for each notebook that allows researchers to develop and execute several notebooks inside the same session of the allotted compute resource rather than having them connect to a different session for each notebook as in google's colab and kaggle kernels.

Implementation

Jupyterlab infrastructure has been developed in two stages. First, a docker container is created containing all the necessary packages such as jupyterlab itself, CUDA, tensorflow, scikit-learn, ONNX and many more. The docker container is inherited from a base container that is well-suited for serving a jupyterlab environment. Such docker containers are collectively known as jupyter docker stacks [38]. In addition to software packages such as numpy, scipy and tensorflow that are already wrapped around the base container, many packages are added with their compatible versions. Compatible packages for CUDA, CUDA DNN and tensorflow are necessary so that they together interact with the GPU on the host machine for accelerating deep learning programs. Other significant packages, integrated into the docker container, are ONNX, scikit-learn, elyra AI, bioblend, nibabel, scikit-image, open-CV, bqplot and voila. The integrated docker container contains all the necessary packages for developing data science, ML and DL projects. Second, the container can be downloaded to any powerful compute infrastructure and jupyterlab can be served in any internet browser via the URL that it generates. In addition, to run this container in Galaxy, an interactive tool is created that downloads this container on a remote compute infrastructure and generates a URL that is used to run jupyterlab in a browser. The architecture of jupyterlab infrastructure in Galaxy is shown in Figure 1. The running instance of jupyterlab in Galaxy contains a home page, a jupyterlab notebook, that summarises several of its features. Further, there are other notebooks available, each describing a feature of the jupyterlab with code examples such as how to create ONNX models for scikit-learn and tensorflow classifiers, how to connect to Galaxy using bioblend, how to create interactive plots using bqplots and how to create a pipeline of notebooks using elyra AI. To access the jupyterlab notebook in Galaxy Europe, a ready-to-use hands-on Galaxy training network (GTN) [39] tutorial [40] is developed that shows steps such as opening the notebook, using git to clone a code repository from github, and sending long-running training jobs to a remote Galaxy cluster. The approach of remote model training is explained in the Methods section. The two use-cases, explained in the Results section, are also discussed in the tutorial along with their respective jupyterlab notebooks.

Results

Jupyterlab notebook infrastructure in Galaxy Europe is used to reproduce the results of two recent scientific publications. They demonstrate its robustness and usefulness to develop deep learning models using COVID CT scan images [41] and predict the 3D structure of proteins using colabfold, a faster implementation of alphafold2 [42].

COVID-19 CT scan image segmentation

In [41], COVID-19 CT scan images have been used to develop and train a deep learning model architecture that predicts COVID-19 infected regions in those images with high accuracy. An open-source implementation of the work is available that trains a unet deep learning architecture [43] that distinguishes between normal and infected regions in CT scan images. Scripts of this implementation are adapted and executed on Galaxy's jupyterlab notebook infrastructure. Adaption only involves the transformation of all CT scan images, used in [41], into an H5 file so that they can directly be used as an input to the unet architecture defined in a jupyterlab notebook in [44]. A composite H5 file [45] is created using script [46] that contains multiple datasets inside and each dataset is a real-valued matrix corresponding to the training, test and validation sets as used in [41]. The entire analysis of [41] can be reproduced using multiple notebooks in [44]. They achieve similar values of precision and recall (approximately 0.98) metrics as mentioned in [41]. In [44], the first notebook (1_fetch_datasets.ipynb) downloads input dataset as an H5 file. Additionally, it also downloads the trained ONNX model. The second notebook (2_create_model_and_train.ipynb) creates and trains a unet model on the training dataset extracted from the H5 file. Training, accelerated by GPU, for 10 iterations over the entire training dataset finishes in a few minutes. The third notebook (3_predict_masks.ipynb) extracts the test dataset and predicts infected regions of the CT scan images in the test dataset using the trained model created by the second notebook. Figure 2 shows the comparison of ground truth infected regions in the second column and the predicted infected regions in the third column. A few original CT scan images from the test dataset are shown in the first column of Figure 2.

Predict 3D structure of proteins using ColabFold

Alphafold2 has made a breakthrough in predicting the 3D structure of proteins with outstanding accuracy. However, due to

Table 1. Comparison of Galaxy jupyterlab with other notebook infrastructures

Indicators/Infrastructures	Google Colab [33]	Kaggle Kernel [34]	Galaxy Jupyterlab
Memory/Disk space (GB)	12/70	16/73	**20/1000**
GPU/TPU	Yes/**Yes**	Yes/**Yes**	**Yes**/No
Max usage time (Hours)	12	12, 30 hrs of GPU/week, 20 hrs of TPU/week	**No time restriction** on GPU usage, notebook sessions and job execution
Dynamic compute resources	Yes	Yes	**Fixed and guaranteed**
Remote model training	No	No	**Yes**
Run multiple notebooks (as tabs) in one session	No	No	**Yes**

their large database size (a few TB), it is not easily accessible to researchers. Therefore, a few approaches have been developed that replace the time-consuming steps of alphafold2 with slightly different steps but predict the 3D structure of proteins with similar accuracy while consuming less memory and time. One such approach is colabfold which replaces a large database search in alphafold2 for finding homologous sequences by a significantly (40-60 times) faster MMseqs2 API [47] call to generate input features based on the query protein sequence. Colabfold's prediction of 3D structures in batches is approximately 90 times faster. It is integrated into the docker container [5] by adding two packages - colabfold and GPU-enabled JAX which is a just-in-time compiler for making mathematical transformations. "7_ColabFold_MMseq2.ipynb" notebook in [44] predicts the 3D structure of a protein sequence using colabfold by making use of the alphafold2 pre-trained weights. Figure 3 shows the 3D structure of 4Oxalocrotonate_Tautomerase [48], a protein sequence of length 62, along with its side chains. This 3D structure is extremely similar to the structure predicted by the jupyter notebook [49] from colabfold in [31].

Methods

Remote model training

For large datasets, model training may need several hours or even days. In such cases, it would be non-ideal to keep the jupyterlab notebook open in a browser's tab till the training finishes. Therefore, another Galaxy tool [50] is developed to enable researchers to send long-running training jobs to a remote Galaxy cluster. The tool can be executed from a jupyterlab notebook using a custom python function [51], part of each jupyterlab notebook, that takes input datasets and training script as input parameters. The input datasets to be used for training, testing and validation must be provided in H5 format. It allows standardisation of input data format for AI models that train on matrices in jupyterlab notebooks. Input data to an AI model can be in multiple formats such as images, genomic sequences or gene expression patterns. H5 files can be created using any of these data formats and fed to the AI model in jupyterlab notebooks. Long-running training happens in a remote Galaxy cluster as a regular Galaxy job. Upon completion of the job, the resulting datasets and the trained model become available in a newly created Galaxy history [52]. The trained model and other resulting datasets can be downloaded from the Galaxy history for further analysis. In [44], a few notebooks are available that showcase the approach of remote model training. Notebook "4_create_model_and_train_remote.ipynb" contains code for developing and training a unet architecture. Notebook "5_run_remote_training.ipynb" executes the previous notebook on a cluster remotely after creating a Galaxy history and then uploading the script extracted from "4_create_model_and_train_remote.ipynb" notebook and input datasets. Custom python function, "run_script_job", creates a Galaxy history using bioblend and then uploads the datasets to the same history. After the upload is finished, the python script from the specified notebook is executed dynamically. It trains a deep learning model on the uploaded datasets to create a model and saves it as an ONNX file in the Galaxy history. Using "6_predict_masks_remote_model.ipynb" notebook from [44], the trained model can be downloaded from the Galaxy history and used for predicting infected regions of the CT scan images of the test dataset. A significant advantage of training deep learning models remotely is that researchers don't have to keep the jupyterlab notebook session running as long as the model is being trained as model training becomes decoupled from jupyterlab notebook. Using such a feature, deep learning models that take several hours or even days to train can be conveniently trained.

Extend docker container

The customised docker container developed as shown in Figure 1 can be easily extended to have more or different packages. To update the container, a package or a list of new packages should be added to the dockerfile and then a new container should be built. Once this new container is pushed to docker hub [5], the next time when Galaxy interactive tool is accessed, it downloads the updated container. All the newly added packages are available in the new container. Similarly, versions of existing packages should be updated or existing packages should be removed if no longer needed. The simplified extension procedure of the entire infrastructure makes it incur low maintenance costs as any change to this entire infrastructure is reflected only in the container without updating Galaxy's codebase.

Collaborative notebooks

Notebooks created in Galaxy's jupyterlab infrastructure are instantly shared with other researchers and collaborators only by sharing the public URL of a notebook. Researchers and users that share a notebook can collaborate on the same notebook without having to store it anywhere as it is directly served from Galaxy Europe.

Workflow of notebooks

Using the elyra AI package, a workflow of notebooks can be created and executed as one unit of software in similar ways as Galaxy workflows are created using several tools. It is possible to execute such workflows of jupyterlab notebooks on the same compute resource on which the jupyterlab notebook runs. In addition, a few other services such as kubeflow [53] or apache airflow [54] can also be used to deploy, run and manage such workflows on cloud.

Summary

Jupyterlab notebook is integrated as an interactive tool in Galaxy Europe running on a public and powerful compute infrastructure comprising several CPUs and GPUs. It is configured in a docker container along with many different packages such as CUDA, tensorflow-GPU and scikit-learn to provide a robust architecture for the development and management of projects in ML, DL and data science. Remote model training makes it convenient to run multiple analyses in parallel as different Galaxy jobs by executing the same Galaxy tool and results become available in different histories. Features such as git integration are useful for managing entire code repositories on github and elyra AI for creating pipelines of notebooks working as one unit of software. All notebooks created by a user run on the same session of the jupyterlab in different tabs. The entire infrastructure of jupyterlab is readily accessible through Galaxy Europe. In contrast to commercial infrastructures that host editors similar to jupyterlab and offer powerful and reliable compute only through paid subscriptions, this infrastructure provides large compute resources invariant to usage and has an unlimited usage time while ensuring the same set of compute resources across multiple usages.

Availability of supporting source code and requirements

Project name: GPU-enabled docker container with Jupyterlab for artificial intelligence
Project home page: https://github.com/anuprulez/ml-jupyter-notebook
Galaxy interactive tool: https://github.com/usegalaxy-eu/galaxy/blob/release_22.01_europe/tools/interactive/interactivetool_ml_jupyter_notebook.xml
Operating system: Linux
Programming languages: Python, XML, Docker, Bash
Other requirements:
License: MIT License
RRID: SCR_022695
Biotools ID: gpu-enabled_docker_container_with_jupyterlab_for_ai

Additional files

Supplementary Material: Docker-based Jupyterlab in Galaxy.

List of abbreviations

AI: Artificial intelligence; CT: Computerised tomography; CUDA: Compute unified device architecture; DL: Deep learning; DNA: Deoxyribonucleic acid; EUR: Euro; GPU: Graphical processing unit; GB: Gigabyte; GTN: Galaxy training network; JAX: Accelerated linear algebra; ML: Machine learning; ONNX: Open neural network exchange; OS: PC: Personal computer; RAM: Random-access memory; TB: Terabyte; UI: User interface; URL: Uniform resource locator;

Competing Interests

The authors declare that they have no competing interests.

Ethics approval and consent to participate

Not applicable

Consent for publication

Not applicable

Funding

This work was supported by the German Research Foundation (DFG) under Germany's Excellence Strategy (CIBSS - EXC-2189 - Project ID 390939984) and German Federal Ministry of Education and Research (BMBF grant 031A538A de.NBI).

Authors' contributions

A.K. developed the project and wrote the manuscript. G.C. deployed the project on Galaxy Europe. B.G. devised the idea of the project. R.B. provided the necessary support for the entire project. All authors contributed to and approved the manuscript.

Acknowledgements

We thank Daniel Blankenberg for his suggestions to improve the docker container. In addition, we thank Galaxy Europe team for running and maintaining the project.

References

1. Pearson W, et al, The FASTA package - protein and DNA sequence similarity searching and alignment programs. GitHub; 2016. https://github.com/wrpearson/fasta36. 2016. Accessed 30 June 2022.
2. Kumar I, Singh SP, Shivam. Chapter 26 - Machine learning in bioinformatics. Academic Press 2022;p. 443–456. https://www.sciencedirect.com/science/article/pii/B9780323897754000201.
3. Kluyver T, Ragan-Kelley B, Pérez F, Granger B, Bussonnier M, Frederic J, et al.; IOS Press. Jupyter Notebooks—a publishing format for reproducible computational workflows 2016;p. 87.
4. Afgan E, Baker D, Batut B, et al. The Galaxy platform for accessible, reproducible and collaborative biomedical analyses: 2018 update. Nucleic Acids Research 2018;46(W1):W537–W544. doi:10.1093/nar/gky379.
5. Kumar A, Container for machine learning and deep learning in Jupyter notebook. Docker; 2021. https://hub.docker.com/r/anupkumar/docker-ml-jupyterlab. 2021. Accessed 29 June 2022.
6. Galaxy Europe, Live instance of the European Galaxy server. Galaxy Europe; 2019. https://live.usegalaxy.eu/. 2019. Accessed 30 June 2022.
7. Kumar A, et al, GPU enabled Interactive Jupyter Notebook for Machine Learning; 2021. https://github.com/

usegalaxy-eu/galaxy/blob/release_22.01_europe/tools/interactive/interactivetool_ml_jupyter_notebook.xml.
8. Merkel D. Docker: lightweight linux containers for consistent development and deployment. Linux journal 2014;2014(239):2.
9. Baset et al , Docker and Container Security White Paper; 2016. https://dominoweb.draco.res.ibm.com/reports/rc25625.pdf.
10. Collonval Fea, A JupyterLab extension for version control using Git.;. https://github.com/jupyterlab/jupyterlab-git. 2017. Accessed 29 June 2022.
11. Resende Lea, Elyra is a set of AI-centric extensions to JupyterLab Notebooks.;. https://github.com/elyra-ai/elyra. 2018. Accessed 29 June 2022.
12. Abadi M, Agarwal A, Barham P, Brevdo E, Chen Z, Citro C, et al., TensorFlow: Large-Scale Machine Learning on Heterogeneous Systems; 2015. https://www.tensorflow.org/, software available from tensorflow.org.
13. Pedregosa F, Varoquaux G, Gramfort A, Michel V, Thirion B, Grisel O, et al. Scikit-learn: Machine Learning in Python. Journal of Machine Learning Research 2011;12:2825–2830.
14. NVIDIA, Vingelmann P, Fitzek FHP, CUDA, release: 10.2.89; 2020. https://developer.nvidia.com/cuda-toolkit.2020.Accessed 29 June 2022.
15. Bai J, Lu F, Zhang K, et al, ONNX: Open Neural Network Exchange. GitHub; 2019. https://github.com/onnx/onnx. 2019. Accessed 29 June 2022.
16. Harris CR, Millman KJ, van der Walt SJ, Gommers R, Virtanen P, Cournapeau D, et al. Array programming with NumPy. Nature 2020 Sep;585(7825):357–362. https://doi.org/10.1038/s41586-020-2649-2.
17. Virtanen P, Gommers R, Oliphant TE, Haberland M, Reddy T, Cournapeau D, et al. SciPy 1.0: Fundamental Algorithms for Scientific Computing in Python. Nature Methods 2020;17:261–272.
18. Jupyter Project, Jupyter Notebook Scientific Python Stack w/ Tensorflow. Docker; 2014. https://hub.docker.com/r/jupyter/tensorflow-notebook. 2014. Accessed 29 June 2022.
19. Kumar A, Jupyter container used for Data Science and Tensorflow. GitHub; 2021. https://github.com/anuprulez/ml-jupyter-notebook/blob/master/Dockerfile. 2021. Accessed 29 June 2022.
20. Waskom ML. seaborn: statistical data visualization. Journal of Open Source Software 2021;6(60):3021. https://doi.org/10.21105/joss.03021.
21. Hunter JD, Matplotlib: A 2D graphics environment. IEEE COMPUTER SOC; 2007.
22. Bokeh Development Team, Bokeh: Python library for interactive visualization; 2018. https://bokeh.pydata.org/en/latest/.
23. Bradski G. The OpenCV Library. Dr Dobb's Journal of Software Tools 2000;.
24. Van der Walt S, Schönberger JL, Nunez-Iglesias J, Boulogne F, Warner JD, Yager N, et al. scikit-image: image processing in Python. PeerJ 2014;2:e453.
25. Brett M, Markiewicz CJ, Hanke M, Côté MA, Cipollini B, McCarthy P, et al., nipy/nibabel: 3.2.2. Zenodo; 2022. https://doi.org/10.5281/zenodo.6617121.
26. Sloggett C, Goonasekera N, Afgan E. BioBlend: automating pipeline analyses within Galaxy and CloudMan. Bioinformatics 2013;29(13):1685–1686. https://doi.org/10.1093/bioinformatics/btt199.
27. Corlay, S and et al , 2-D plotting library for Project Jupyter. GitHub; 2015. https://github.com/bqplot/bqplot. 2015. Accessed 29 June 2022.
28. Tuloup, J and et al , Rendering of live Jupyter notebooks with interactive widgets. GitHub; 2018. https://github.com/voila-dashboards/voila. 2018. Accessed 29 June 2022.
29. Tomlinson, J and et al , A JupyterLab extension for displaying GPU usage dashboards. GitHub; 2021. https://github.com/rapidsai/jupyterlab-nvdashboard. 2021. Accessed 29 June 2022.
30. The HDF Group, Hierarchical Data Format, version 5; 1997-2022. https://www.hdfgroup.org/HDF5/. 1997. Accessed 29 June 2022.
31. Mirdita M, Schütze K, Moriwaki Y, et al. ColabFold: making protein folding accessible to all. Nat Methods 2022;19:679–682 (2022). https://doi.org/10.1038/s41592-022-01488-1.
32. Johnson M, et al, JAX: Autograd and XLA; 2020. https://github.com/google/jax. 2020. Accessed 29 June 2022.
33. Bisong E. Google Colaboratory 2019;p. 59–64. https://doi.org/10.1007/978-1-4842-4470-8_7.
34. Kaggle, Kaggle; 2020. https://www.kaggle.com. 2010. Accessed 29 June 2022.
35. Amazon SageMaker, Amazon SageMaker; 2017. https://aws.amazon.com/sagemaker/. 2017. Accessed 29 June 2022.
36. Moon KR, van Dijk D, Wang Zea. Visualizing structure and transitions in high-dimensional biological data. Nat Biotechnol 2019;37:1482–1492 (2019). https://doi.org/10.1038/s41587-019-0336-3.
37. Boileau P, Hejazi NS, Dudoit S. Exploring high-dimensional biological data with sparse contrastive principal component analysis. Bioinformatics 2020;36(11):3422–3430. https://doi.org/10.1093/bioinformatics/btaa176.
38. Jupyter Docker Stacks. Github; 2015. https://jupyter-docker-stacks.readthedocs.io/en/latest/.
39. Batut B, Hiltemann S, Bagnacani A, Baker D, Bhardwaj V, Blank C, et al. Community-Driven Data Analysis Training for Biology. Cell Systems 2018 jun;6(6):752–758.e1. https://doi.org/10.1016/j.cels.2018.05.012.
40. Kumar A, A Docker-based interactive Jupyterlab powered by GPU for artificial intelligence in Galaxy (Galaxy Training Materials); 2022. https://training.galaxyproject.org/training-material/topics/statistics/tutorials/gpu_jupyter_lab/tutorial.html. 2022. Accessed 29 June 2022.
41. Saeedizadeh N, Minaee S, Kafieh R, Yazdani S, Sonka M. COVID TV-Unet: Segmenting COVID-19 chest CT images using connectivity imposed Unet. Computer Methods and Programs in Biomedicine Update 2021;1:100007. https://www.sciencedirect.com/science/article/pii/S2666990021000069.
42. Jumper J, Evans R, Pritzel A, et al. Highly accurate protein structure prediction with AlphaFold. Nature 2021;596:583–589 (2021). https://doi.org/10.1038/s41586-021-03819-2.
43. Ronneberger O, Fischer P, Brox T. U-Net: Convolutional Networks for Biomedical Image Segmentation. CoRR 2015;abs/1505.04597. http://arxiv.org/abs/1505.04597.
44. Kumar A, Jupyterlab notebooks. GitHub; 2022. https://github.com/anuprulez/gpu_jupyterlab_ct_image_segmentation. 2022. Accessed 29 June 2022.
45. Kumar A, COVID Image segmentation datasets and trained model. Zenodo; 2022. https://doi.org/10.5281/zenodo.6091361.
46. Saeedizadeh N, Minaee S, Kafieh R, Yazdani S, Sonka M, COVID TV-Unet: Segmenting COVID-19 chest CT images using connectivity imposed Unet. Github; 2021. https://github.com/narges-sa/COVID-CT-Segmentation/blob/main/main_TV_Unet_Split1.py. 2021. Accessed 30 June 2022.
47. Steinegger M, Söding J. MMseqs2 enables sensitive protein sequence searching for the analysis of massive data sets. Nat Biotechnol 2017;35:1026–1028 (2017). https://doi.org/10.1038/nbt.3988.

48. Chen L, Kenyon G, Curtin F, Harayama S, Bembenek M, Hajipour G, et al. 4-Oxalocrotonate tautomerase, an enzyme composed of 62 amino acid residues per monomer. J Biol Chem 1992;267(25):17716–21. https://pubmed.ncbi.nlm.nih.gov/1339435/.
49. Mirdita M, Schütze K, Moriwaki Y, et al, ColabFold: making protein folding accessible to all. Github; 2022. https://github.com/sokrypton/ColabFold/blob/main/AlphaFold2.ipynb. 2022. Accessed 30 June 2022.
50. Kumar A, Run long running jupyterlab script. Github; 2022. https://github.com/bgruening/galaxytools/blob/master/tools/jupyter_job/run_jupyter_job.xml. 2022. Accessed 30 June 2022.
51. Kumar A, Custom jupyterlab notebook function to start model training job in Galaxy. Github; 2021. https://github.com/anuprulez/ml-jupyter-notebook/blob/master/galaxy_script_job.py#L43. 2021. Accessed 30 June 2022.
52. Kumar A, Remotely trained image segmentation model. Galaxy; 2022. https://usegalaxy.eu/u/kumara/h/image-segmentation-from-galaxy-jupyterlab. 2022. Accessed 23 August 2022.
53. Kubeflow. Github; 2017. https://github.com/kubeflow/kubeflow.
54. Apache Airflow. Github; 2019. https://github.com/apache/airflow-site.

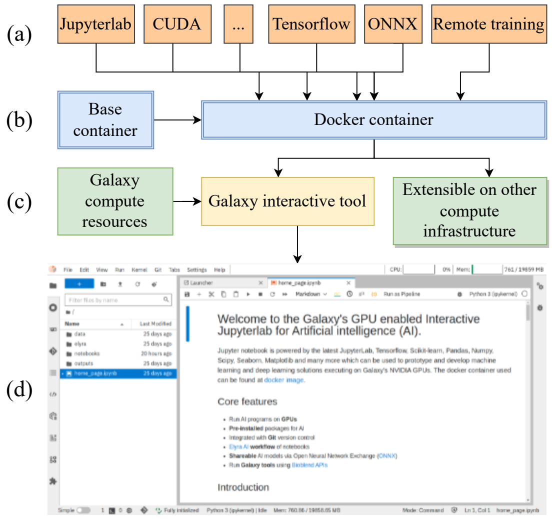

Figure 1. Architecture of Galaxy's jupyterlab. Part (a) shows packages and features wrapped inside a docker container. Part (b) shows a base docker container [18] from which the customised container [5] is derived. In part (c), Galaxy's interactive tool downloads the customised container. The customised docker container can also be hosted on a different compute infrastructure. Part (d) shows Galaxy's jupyterlab.

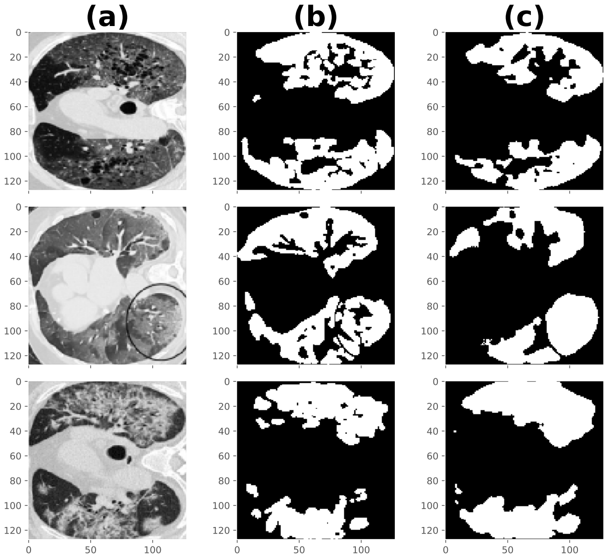

Figure 2. Figure shows original CT scan images in column (a), corresponding ground-truth masks of original CT scan images in column (b) and the predicted masks in column (c). Masks are COVID-19 infected regions in the corresponding CT scan images. The ground-truth and predicted masks show high similarity [41].

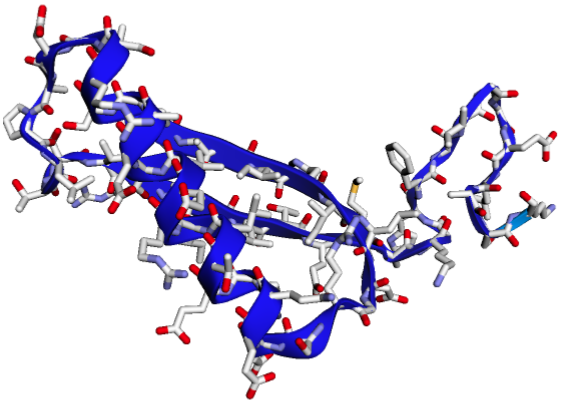

Figure 3. Figures shows a 3D structure of 4 Oxalocrotonate_Tautomerase enzyme (protein) [48] predicted by ColabFold

Reference PDF

Click here to access/download;Manuscript;GPU_Jupyterlab_Galaxy_images

GigaScience, 0000, 1–7

doi: xx.xxxx/xxxx
Manuscript in Preparation
Technical Note

TECHNICAL NOTE

An accessible infrastructure for artificial intelligence using a docker-based Jupyterlab in Galaxy

Anup Kumar[1,*,†], Gianmauro Cuccuru[1,‡,†], Björn Grüning[1,§,†] and Rolf Backofen[1,2,¶,†]

[1]Bioinformatics Group, Department of Computer Science, University of Freiburg, Georges-Koehler-Allee 106, 79110 Freiburg, Germany and [2]Signalling Research Centres BIOSS and CIBSS, University of Freiburg, Schaenzlestr. 18, 79104 Freiburg, Germany

[*]kumara@informatik.uni-freiburg.de https://orcid.org/0000-0002-2068-4695
[‡]gmauro@gmail.com https://orcid.org/0000-0002-5335-545X
[§]gruening@informatik.uni-freiburg.de https://orcid.org/0000-0002-3079-6586
[¶]backofen@informatik.uni-freiburg.de https://orcid.org/0000-0001-8231-3323
[†]Contributions follow the order of the names of authors

Abstract

Background Artificial intelligence (AI) programs that train on a large amount of data require powerful compute infrastructure. Jupyterlab notebook provides an excellent framework for developing AI programs but it needs to be hosted on a powerful infrastructure to enable AI programs to train on large data. **Findings** An open-source, docker-based, and GPU-enabled jupyterlab notebook infrastructure is developed that runs on the public compute infrastructure of Galaxy Europe to rapidly prototype and develop end-to-end AI projects. Using such a notebook, long-running AI model training programs can be executed remotely to create trained models, represented in open neural network exchange (ONNX) format, and other output datasets in Galaxy. Other features include GPU support for faster training, git integration for version control, the option of creating and executing pipelines of notebooks, and multiple dashboards for monitoring compute resources and packages for visualisation. **Conclusions** These features make the jupyterlab notebook highly suitable for creating and managing AI projects. A recent scientific publication that predicts infected regions in COVID-19 CT scan images is reproduced using various features of this notebook. In addition, colabfold, a faster implementation of alphafold2, is accessed in this notebook to predict the 3D structure of protein sequences. Jupyterlab notebook is accessible in two ways - one as an interactive Galaxy tool and the other by running the underlying docker container. In both ways, long-running training can be executed on Galaxy's compute infrastructure. Scripts to create the docker container are available under MIT license at https://github.com/anuprulez/ml-jupyter-notebook.

Key words: Jupyterlab notebooks; Galaxy Europe; Artificial intelligence; Remote model training; ONNX; Elyra AI; GPU; CUDA;

Findings

Background

Bioinformatics comprises many sub-fields such as single-cell, medical imaging, sequencing, proteomics and many more that produce a huge amount of biological data in myriad formats. For example, the single-cell field creates gene expression patterns for each cell that are represented as matrices of real numbers. The medical imaging field generates images of cells and tissues, radiography images such as chest x-rays and computerized tomography (CT) scans. Next-Generation sequencing generates deoxyribonucleic acid (DNA) sequences that are stored as fasta [1] files. Artificial intelligence (AI) approaches such as machine learning (ML) and deep

Compiled on: August 24, 2022.
Draft manuscript prepared by the author.

learning (DL) have been vastly used with these datasets [2] for predictive tasks such as medical diagnosis, imputing missing features, augmenting datasets with artificially generated ones, and estimating gene expression patterns and many more. To be able to use ML and DL algorithms on such datasets, a robust and efficient compute infrastructure is needed that can serve multiple purposes. They include pre-processing raw datasets to transform them into suitable formats that are compatible with ML and DL algorithms, creating and executing their complex architectures on pre-processed datasets and making trained models and predicted datasets readily available for further analyses. To facilitate such tasks, a complete infrastructure is developed that combines jupyterlab [3] notebook, augmented with many useful features, running on public compute resources of Galaxy [4] Europe to perform end-to-end AI analyses on biological datasets. The infrastructure consists of three important components. First, a docker container [5] that encapsulates jupyterlab together with a large number of packages used for developing AI programs, data manipulation and visualisation. Section S2 in the supplementary file lists all the packages with their respective versions. Second, a Galaxy interactive tool [6, 7] that downloads this docker container to serve jupyterlab online on Galaxy Europe. Third, the compute infrastructure consisting of several CPUs and GPUs that acts as a backend on which the online jupyterlab runs.

Docker container

Docker [8] containers are popular for shipping packaged software as complete ecosystems enabling them to be reproducible in a platform-independent manner. Software executing inside a docker container is abstracted from the operating system (OS) as most of the requirements necessary for them to run successfully are already configured inside the container. A container runs as an isolated environment making the minimum number of interactions to the host OS and thereby, improving the security aspects of the running software. Using a docker container leverages the security benefits that are necessary to have for online program editor softwares executing arbitrary code. In addition to minimising security risks, docker containers provide performance benefits compared to running programs on a virtual machine [9]. Motivated by such benefits, a docker container is used in this project to encapsulate jupyterlab along with many useful packages such as git [10], elyra AI [11], tensorflow-GPU [12], scikit-learn [13], CUDA [14], ONNX [15], and many others. Docker container inherits many packages such as numpy [16], scipy [17] and a few more from its base container, jupyter/tensorflow-notebook [18], and augments them with many other packages suitable for ML and DL, data manipulation and visualisation. Docker container is decoupled from Galaxy and can independently be executed for serving jupyterlab with the same set of packages on a different compute infrastructure or any personal computer (PC) or laptop having approximately 25 gigabytes (GB) of disk space. Moreover, it can easily be extended by installing suitable packages only by adding their appropriate package names in its dockerfile [19]. The approach extending the docker container is discussed in Methods section in detail.

Jupyterlab

Jupyterlab is a web-based, robust editor used for varied purposes such as data science, scientific computing, machine learning and deep learning. It is a program editor that supports more than 40 programming languages, some of the popular ones are python, R, julia and scala. Python is one of the most popular languages used by researchers for performing numerous scientific and predictive analyses. Therefore, it is used as the programming language in Galaxy jupyterlab notebooks because many popular packages such as tensorflow, scikit-learn for ML and DL, data manipulation packages such as pandas, visualisation packages such as seaborn [20], matplotlib [21], bokeh [22] and many others are readily available as python packages. Moreover, the extensible architecture of jupyterlab makes it possible to add many external packages as jupyterlab plugins such as git, elyra AI, dashboards and many others that have a user interface (UI) as necessary components. Editors such as jupyterlab, integrated with several useful packages, provide a favourable platform for both, rapid prototyping and end-to-end development and management of AI projects. To harness the benefits of jupyterlab, it has been used as the editor for the Jupyterlab interactive tool in Galaxy.

Features of jupyterlab notebook infrastructure

Many features such as easy accessibility, support of a wide variety of programming languages on jupyterlab, and extensibility to install useful plugins make it a desirable editor for researchers for creating project prototypes rapidly. Many such features have been integrated into a jupyterlab notebook infrastructure that serves jupyterlab notebook online on Galaxy Europe running on large compute resources enabling researchers to create prototypes and end-to-end AI projects (Figure 1). A few important features are discussed here. To allow GPU computation from jupyterlab notebooks, tensorflow-GPU interacts with Nvidia GPU hardware using another software, CUDA, when the compute resource has GPU(s) for accelerating deep learning programs. Faster execution of deep learning programs is one of the significant features of jupyterlab hosted on Galaxy Europe. However, if the hosted machine on which a docker container runs does not have GPUs, then the program in a jupyterlab notebook relies on CPUs. Other useful features include ONNX for transforming trained tensorflow and scikit-learn models to ONNX models, open-CV [23] and scikit-image [24] for processing images, nibabel [25] for reading image files stored as ".nii", bioblend [26] for accessing Galaxy's datasets, histories and workflows in a jupyterlab notebook and visualisation packages such as bqplot [27] and bokeh for plotting interactive charts, voila [28] for displaying output cells of a jupyterlab notebook, dashboard such as nvdashboard [29] for monitoring GPU usage and performance. Support for the file extension such as H5 [30], efficient for storing matrices, enables machine learning researchers to save model weights and input datasets for AI algorithms. Other packages such as colabfold [31] together with JAX [32] are used for predicting 3D structures of proteins which are discussed in detail in the Results section. In addition, it is possible to create a long-running training job that runs remotely and stores trained models and output datasets permanently in a newly created Galaxy history. The trained model is saved as an ONNX file and tabular datasets in H5 file.

Related infrastructure

There are a few other infrastructures available, free and commercial, that offer jupyterlab or similar environments for developing data science and AI projects. A few popular ones are google colab [33], kaggle kernels [34] and amazon's sagemaker [35]. Google colab is partially free and offers an online editor similar to a jupyterlab notebook. The free version of colab offers dynamic compute resources. The disk space is around 70 GB and the memory (RAM) is around 12 GB. These resources are scarce for the AI projects that deal with high-dimensional biological data [36, 37]. In addition, these resources offered by colab are variable and depend on a user's past usage. More compute resources are assigned to those users that have used less in the past for a more equitable sharing of resources. Moreover, there is a limitation of only 12 hours of running time that may prove to be insufficient and non-ideal for training AI models on large datasets. However, colab pro and pro+ offer better compute resources but they come at a price, EUR 9.25 and EUR 42.25 per month, respectively. In contrast, kaggle kernels are free of charge but similar to colab, their computing resources are scarce. The total

disk space is approximately 73 GB and RAM is 16 GB for a CPU-based kernel. For the GPU-based kernel, the disk space is of the same size as that of the CPU-based kernel but the RAM of the CPU decreases to 13 GB. An additional RAM of 15 GB is added through a GPU and computation time is limited to 30 hours a week. It also supports TPUs but the computation time is further limited to only 20 hours a week. Amazon's sagemaker is also a commercial software for developing AI algorithms that is free of charge but only for 2 months. Overall, these notebook infrastructures do not offer unrestricted compute resources free of charge. In addition, compute resources offered free of charge are insufficient for training AI models on high-dimensional biological datasets. To address the drawbacks of these notebook infrastructures and provide researchers and users large compute resources more reliably, Galaxy jupyterlab notebook infrastructure offers 1 terabyte (TB) of disk space and unlimited computation time on 1 GPU and 7 CPUs per session. RAM for GPU is around 15 GB and for CPUs is 20 GB (Table 1). The offered resources for the jupyterlab notebook running in Galaxy stay constant and are independent of the user's past usage. To make it more useful, jupyterlab opens a tab for each notebook that allows researchers to develop and execute several notebooks inside the same session of the allotted compute resource rather than having them connect to a different session for each notebook as in google's colab and kaggle kernels.

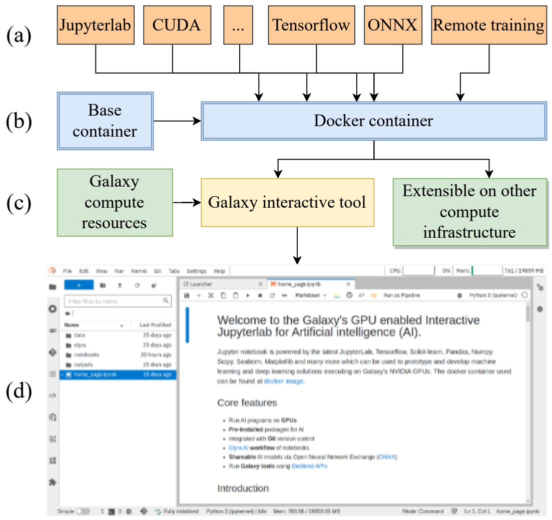

Figure 1. Architecture of Galaxy's jupyterlab. Part (a) shows packages and features wrapped inside a docker container. Part (b) shows a base docker container [18] from which the customised container [5] is derived. In part (c), Galaxy's interactive tool downloads the customised container. The customised docker container can also be hosted on a different compute infrastructure. Part (d) shows Galaxy's jupyterlab.

Implementation

Jupyterlab infrastructure has been developed in two stages. First, a docker container is created containing all the necessary packages such as jupyterlab itself, CUDA, tensorflow, scikit-learn, ONNX and many more. The docker container is inherited from a base container that is well-suited for serving a jupyterlab environment. Such docker containers are collectively known as jupyter docker stacks [38]. In addition to software packages such as numpy, scipy and tensorflow that are already wrapped around the base container, many packages are added with their compatible versions. Compatible packages for CUDA, CUDA DNN and tensorflow are necessary so that they together interact with the GPU on the host machine for accelerating deep learning programs. Other significant packages, integrated into the docker container, are ONNX, scikit-learn, elyra AI, bioblend, nibabel, scikit-image, open-CV, bqplot and voila. The integrated docker container contains all the necessary packages for developing data science, ML and DL projects. Second, the container can be downloaded to any powerful compute infrastructure and jupyterlab can be served in any internet browser via the URL that it generates. In addition, to run this container in Galaxy, an interactive tool is created that downloads this container on a remote compute infrastructure and generates a URL that is used to run jupyterlab in a browser. The architecture of jupyterlab infrastructure in Galaxy is shown in Figure 1. The running instance of jupyterlab in Galaxy contains a home page, a jupyterlab notebook, that summarises several of its features. Further, there are other notebooks available, each describing a feature of the jupyterlab with code examples such as how to create ONNX models for scikit-learn and tensorflow classifiers, how to connect to Galaxy using bioblend, how to create interactive plots using bqplots and how to create a pipeline of notebooks using elyra AI. To access the jupyterlab notebook in Galaxy Europe, a ready-to-use hands-on Galaxy training network (GTN) [39] tutorial [40] is developed that shows steps such as opening the notebook, using git to clone a code repository from github, and sending long-running training jobs to a remote Galaxy cluster. The approach of remote model training is explained in the Methods section. The two use-cases, explained in the Results section, are also discussed in the tutorial along with their respective jupyterlab notebooks.

Results

Jupyterlab notebook infrastructure in Galaxy Europe is used to reproduce the results of two recent scientific publications. They demonstrate its robustness and usefulness to develop deep learning models using COVID CT scan images [41] and predict the 3D structure of proteins using colabfold, a faster implementation of alphafold2 [42].

COVID-19 CT scan image segmentation

In [41], COVID-19 CT scan images have been used to develop and train a deep learning model architecture that predicts COVID-19 infected regions in those images with high accuracy. An open-source implementation of the work is available that trains a unet deep learning architecture [43] that distinguishes between normal and infected regions in CT scan images. Scripts of this implementation are adapted and executed on Galaxy's jupyterlab notebook infrastructure. Adaption only involves the transformation of all CT scan images, used in [41], into an H5 file so that they can directly be used as an input to the unet architecture defined in a jupyterlab notebook in [44]. A composite H5 file [45] is created using script [46] that contains multiple datasets inside and each dataset is a real-valued matrix corresponding to the training, test and validation sets as used in [41]. The entire analysis of [41] can be reproduced using multiple notebooks in [44]. They achieve similar values of precision and recall (approximately 0.98) metrics as mentioned in [41]. In [44], the first notebook (1_fetch_datasets.ipynb) downloads input dataset as an H5 file. Additionally, it also downloads the trained ONNX model. The second notebook (2_create_model_and_train.ipynb) creates and trains a unet model on the training dataset extracted from the H5 file. Training, accelerated by GPU, for 10 iterations over the entire training dataset finishes in a few minutes. The third notebook (3_predict_masks.ipynb) extracts the test dataset and predicts infected regions of the CT scan images in the test dataset using the trained model created by the second notebook. Figure 2 shows the comparison of ground truth infected regions in the second column and the predicted infected regions in the third column. A few original CT scan images from the test dataset are shown in the first column of Figure 2.

Table 1. Comparison of Galaxy jupyterlab with other notebook infrastructures

Indicators/Infrastructures	Google Colab [33]	Kaggle Kernel [34]	Galaxy Jupyterlab
Memory/Disk space (GB)	12/70	16/73	**20/1000**
GPU/TPU	Yes/**Yes**	Yes/**Yes**	**Yes**/No
Max usage time (Hours)	12	12, 30 hrs of GPU/week, 20 hrs of TPU/week	**No time restriction** on GPU usage, notebook sessions and job execution
Dynamic compute resources	Yes	Yes	**Fixed and guaranteed**
Remote model training	No	No	**Yes**
Run multiple notebooks (as tabs) in one session	No	No	**Yes**

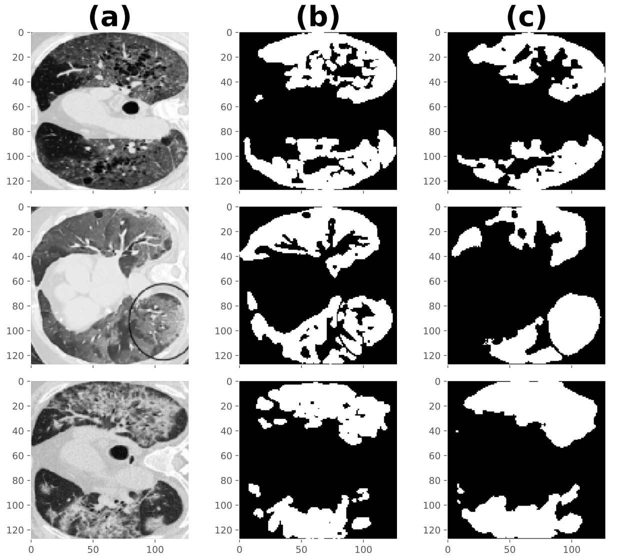

Figure 2. Figure shows original CT scan images in column (a), corresponding ground-truth masks of original CT scan images in column (b) and the predicted masks in column (c). Masks are COVID-19 infected regions in the corresponding CT scan images. The ground-truth and predicted masks show high similarity [41].

Predict 3D structure of proteins using ColabFold

Alphafold2 has made a breakthrough in predicting the 3D structure of proteins with outstanding accuracy. However, due to their large database size (a few TB), it is not easily accessible to researchers. Therefore, a few approaches have been developed that replace the time-consuming steps of alphafold2 with slightly different steps but predict the 3D structure of proteins with similar accuracy while consuming less memory and time. One such approach is colabfold which replaces a large database search in alphafold2 for finding homologous sequences by a significantly (40-60 times) faster MM-seqs2 API [47] call to generate input features based on the query protein sequence. Colabfold's prediction of 3D structures in batches is approximately 90 times faster. It is integrated into the docker container [5] by adding two packages - colabfold and GPU-enabled JAX which is a just-in-time compiler for making mathematical transformations. "7_ColabFold_MMseq2.ipynb" notebook in [44] predicts the 3D structure of a protein sequence using colabfold by making use of the alphafold2 pre-trained weights. Figure 3 shows the 3D structure of 4Oxalocrotonate_Tautomerase [48], a protein sequence of length 62, along with its side chains. This 3D structure is extremely similar to the structure predicted by the jupyter notebook [49] from colabfold in [31].

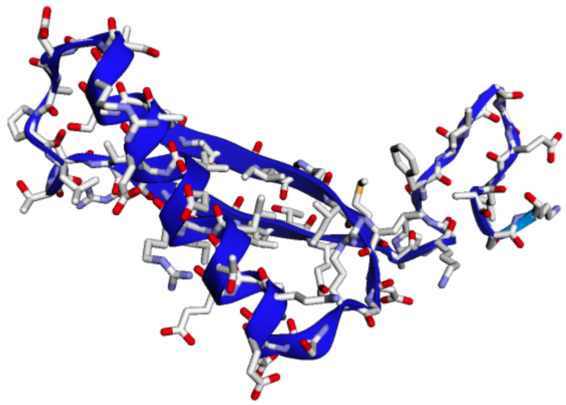

Figure 3. Figures shows a 3D structure of 4 Oxalocrotonate_Tautomerase enzyme (protein) [48] predicted by ColabFold

Methods

Remote model training

For large datasets, model training may need several hours or even days. In such cases, it would be non-ideal to keep the jupyterlab notebook open in a browser's tab till the training finishes. Therefore, another Galaxy tool [50] is developed to enable researchers to send long-running training jobs to a remote Galaxy cluster. The tool can be executed from a jupyterlab notebook using a custom python function [51], part of each jupyterlab notebook, that takes input datasets and training script as input parameters. The input datasets to be used for training, testing and validation must be provided in H5 format. It allows standardisation of input data format for AI models that train on matrices in jupyterlab notebooks. Input data to an AI model can be in multiple formats such as images, genomic sequences or gene expression patterns. H5 files can be created using any of these data formats and fed to the AI model in jupyterlab notebooks. Long-running training happens in a remote Galaxy cluster as a regular Galaxy job. Upon completion of the job, the resulting datasets and the trained model become available in a newly created Galaxy history [52]. The trained model and other resulting datasets can be downloaded from the Galaxy history for further analysis. In [44], a few notebooks are available that showcase the approach of remote model training. Notebook "4_create_model_and_train_remote.ipynb" contains code for developing and training a unet architecture. Notebook "5_run_remote_training.ipynb" executes the previous notebook on a cluster remotely after creating a Galaxy history and then uploading the script extracted from "4_create_model_and_train_remote.ipynb" notebook and input datasets.

Custom python function, "run_script_job", creates a Galaxy history using bioblend and then uploads the datasets to the same history. After the upload is finished, the python script from the specified notebook is executed dynamically. It trains a deep learning model on the uploaded datasets to create a model and saves it as an ONNX file in the Galaxy history. Using "6_predict_masks_remote_model.ipynb" notebook from [44], the trained model can be downloaded from the Galaxy history and used for predicting infected regions of the CT scan images of the test dataset. A significant advantage of training deep learning models remotely is that researchers don't have to keep the jupyterlab notebook session running as long as the model is being trained as model training becomes decoupled from jupyterlab notebook. Using such a feature, deep learning models that take several hours or even days to train can be conveniently trained.

Extend docker container

The customised docker container developed as shown in Figure 1 can be easily extended to have more or different packages. To update the container, a package or a list of new packages should be added to the dockerfile and then a new container should be built. Once this new container is pushed to docker hub [5], the next time when Galaxy interactive tool is accessed, it downloads the updated container. All the newly added packages are available in the new container. Similarly, versions of existing packages should be updated or existing packages should be removed if no longer needed. The simplified extension procedure of the entire infrastructure makes it incur low maintenance costs as any change to this entire infrastructure is reflected only in the container without updating Galaxy's codebase.

Collaborative notebooks

Notebooks created in Galaxy's jupyterlab infrastructure are instantly shared with other researchers and collaborators only by sharing the public URL of a notebook. Researchers and users that share a notebook can collaborate on the same notebook without having to store it anywhere as it is directly served from Galaxy Europe.

Workflow of notebooks

Using the elyra AI package, a workflow of notebooks can be created and executed as one unit of software in similar ways as Galaxy workflows are created using several tools. It is possible to execute such workflows of jupyterlab notebooks on the same compute resource on which the jupyterlab notebook runs. In addition, a few other services such as kubeflow [53] or apache airflow [54] can also be used to deploy, run and manage such workflows on cloud.

Summary

Jupyterlab notebook is integrated as an interactive tool in Galaxy Europe running on a public and powerful compute infrastructure comprising several CPUs and GPUs. It is configured in a docker container along with many different packages such as CUDA, tensorflow-GPU and scikit-learn to provide a robust architecture for the development and management of projects in ML, DL and data science. Remote model training makes it convenient to run multiple analyses in parallel as different Galaxy jobs by executing the same Galaxy tool and results become available in different histories. Features such as git integration are useful for managing entire code repositories on github and elyra AI for creating pipelines of notebooks working as one unit of software. All notebooks created by a user run on the same session of the jupyterlab in different tabs. The entire infrastructure of jupyterlab is readily accessible through Galaxy Europe. In contrast to commercial infrastructures that host editors similar to jupyterlab and offer powerful and reliable compute only through paid subscriptions, this infrastructure provides large compute resources invariant to usage and has an unlimited usage time while ensuring the same set of compute resources across multiple usages.

Availability of supporting source code and requirements

Project name: GPU-enabled docker container with Jupyterlab for artificial intelligence
Project home page: https://github.com/anuprulez/ml-jupyter-notebook
Galaxy interactive tool: https://github.com/usegalaxy-eu/galaxy/blob/release_22.01_europe/tools/interactive/interactivetool_ml_jupyter_notebook.xml
Operating system: Linux
Programming languages: Python, XML, Docker, Bash
Other requirements:
License: MIT License
RRID: SCR_022695
Biotools ID: gpu-enabled_docker_container_with_jupyterlab_for_ai

Additional files

Supplementary Material: Docker-based Jupyterlab in Galaxy.

List of abbreviations

AI: Artificial intelligence; CT: Computerised tomography; CUDA: Compute unified device architecture; DL: Deep learning; DNA: Deoxyribonucleic acid; EUR: Euro; GPU: Graphical processing unit; GB: Gigabyte; GTN: Galaxy training network; JAX: Accelerated linear algebra; ML: Machine learning; ONNX: Open neural network exchange; OS: PC: Personal computer; RAM: Random-access memory; TB: Terabyte; UI: User interface; URL: Uniform resource locator;

Competing Interests

The authors declare that they have no competing interests.

Ethics approval and consent to participate

Not applicable

Consent for publication

Not applicable

Funding

This work was supported by the German Research Foundation (DFG) under Germany's Excellence Strategy (CIBSS - EXC-2189 - Project ID 390939984) and German Federal Ministry of Education and Research (BMBF grant 031A538A de.NBI).

Authors' contributions

A.K. developed the project and wrote the manuscript. G.C. deployed the project on Galaxy Europe. B.G. devised the idea of the project. R.B. provided the necessary support for the entire project. All authors contributed to and approved the manuscript.

Acknowledgements

We thank Daniel Blankenberg for his suggestions to improve the docker container. In addition, we thank Galaxy Europe team for running and maintaining the project.

References

1. Pearson W, et al, The FASTA package - protein and DNA sequence similarity searching and alignment programs. GitHub; 2016. https://github.com/wrpearson/fasta36. 2016. Accessed 30 June 2022.
2. Kumar I, Singh SP, Shivam. Chapter 26 - Machine learning in bioinformatics. Academic Press 2022;p. 443–456. https://www.sciencedirect.com/science/article/pii/B9780323897754000201.
3. Kluyver T, Ragan-Kelley B, Pérez F, Granger B, Bussonnier M, Frederic J, et al.; IOS Press. Jupyter Notebooks—a publishing format for reproducible computational workflows 2016;p. 87.
4. Afgan E, Baker D, Batut B, et al. The Galaxy platform for accessible, reproducible and collaborative biomedical analyses: 2018 update. Nucleic Acids Research 2018;46(W1):W537–W544. doi:10.1093/nar/gky379.
5. Kumar A, Container for machine learning and deep learning in Jupyter notebook. Docker; 2021. https://hub.docker.com/r/anupkumar/docker-ml-jupyterlab. 2021. Accessed 29 June 2022.
6. Galaxy Europe, Live instance of the European Galaxy server. Galaxy Europe; 2019. https://live.usegalaxy.eu/. 2019. Accessed 30 June 2022.
7. Kumar A, et al, GPU enabled Interactive Jupyter Notebook for Machine Learning; 2021. https://github.com/usegalaxy-eu/galaxy/blob/release_22.01_europe/tools/interactive/interactivetool_ml_jupyter_notebook.xml.
8. Merkel D. Docker: lightweight linux containers for consistent development and deployment. Linux journal 2014;2014(239):2.
9. Baset et al , Docker and Container Security White Paper; 2016. https://dominoweb.draco.res.ibm.com/reports/rc25625.pdf.
10. Collonval Fea, A JupyterLab extension for version control using Git.;. https://github.com/jupyterlab/jupyterlab-git. 2017. Accessed 29 June 2022.
11. Resende Lea, Elyra is a set of AI-centric extensions to JupyterLab Notebooks.;. https://github.com/elyra-ai/elyra. 2018. Accessed 29 June 2022.
12. Abadi M, Agarwal A, Barham P, Brevdo E, Chen Z, Citro C, et al., TensorFlow: Large-Scale Machine Learning on Heterogeneous Systems; 2015. https://www.tensorflow.org/, software available from tensorflow.org.
13. Pedregosa F, Varoquaux G, Gramfort A, Michel V, Thirion B, Grisel O, et al. Scikit-learn: Machine Learning in Python. Journal of Machine Learning Research 2011;12:2825–2830.
14. NVIDIA, Vingelmann P, Fitzek FHP, CUDA, release: 10.2.89; 2020. https://developer.nvidia.com/cuda-toolkit.2020.Accessed 29 June 2022.
15. Bai J, Lu F, Zhang K, et al, ONNX: Open Neural Network Exchange. GitHub; 2019. https://github.com/onnx/onnx. 2019. Accessed 29 June 2022.
16. Harris CR, Millman KJ, van der Walt SJ, Gommers R, Virtanen P, Cournapeau D, et al. Array programming with NumPy. Nature 2020 Sep;585(7825):357–362. https://doi.org/10.1038/s41586-020-2649-2.
17. Virtanen P, Gommers R, Oliphant TE, Haberland M, Reddy T, Cournapeau D, et al. SciPy 1.0: Fundamental Algorithms for Scientific Computing in Python. Nature Methods 2020;17:261–272.
18. Jupyter Project, Jupyter Notebook Scientific Python Stack w/ Tensorflow. Docker; 2014. https://hub.docker.com/r/jupyter/tensorflow-notebook. 2014. Accessed 29 June 2022.
19. Kumar A, Jupyter container used for Data Science and Tensorflow. GitHub; 2021. https://github.com/anuprulez/ml-jupyter-notebook/blob/master/Dockerfile. 2021. Accessed 29 June 2022.
20. Waskom ML. seaborn: statistical data visualization. Journal of Open Source Software 2021;6(60):3021. https://doi.org/10.21105/joss.03021.
21. Hunter JD, Matplotlib: A 2D graphics environment. IEEE COMPUTER SOC; 2007.
22. Bokeh Development Team, Bokeh: Python library for interactive visualization; 2018. https://bokeh.pydata.org/en/latest/.
23. Bradski G. The OpenCV Library. Dr Dobb's Journal of Software Tools 2000;.
24. Van der Walt S, Schönberger JL, Nunez-Iglesias J, Boulogne F, Warner JD, Yager N, et al. scikit-image: image processing in Python. PeerJ 2014;2:e453.
25. Brett M, Markiewicz CJ, Hanke M, Côté MA, Cipollini B, McCarthy P, et al., nipy/nibabel: 3.2.2. Zenodo; 2022. https://doi.org/10.5281/zenodo.6617121.
26. Sloggett C, Goonasekera N, Afgan E. BioBlend: automating pipeline analyses within Galaxy and CloudMan. Bioinformatics 2013;29(13):1685–1686. https://doi.org/10.1093/bioinformatics/btt199.
27. Corlay, S and et al , 2-D plotting library for Project Jupyter. GitHub; 2015. https://github.com/bqplot/bqplot. 2015. Accessed 29 June 2022.
28. Tuloup, J and et al , Rendering of live Jupyter notebooks with interactive widgets. GitHub; 2018. https://github.com/voila-dashboards/voila. 2018. Accessed 29 June 2022.
29. Tomlinson, J and et al , A JupyterLab extension for displaying GPU usage dashboards. GitHub; 2021. https://github.com/rapidsai/jupyterlab-nvdashboard. 2021. Accessed 29 June 2022.
30. The HDF Group, Hierarchical Data Format, version 5; 1997-2022. https://www.hdfgroup.org/HDF5/. 1997. Accessed 29 June 2022.
31. Mirdita M, Schütze K, Moriwaki Y, et al. ColabFold: making protein folding accessible to all. Nat Methods 2022;19:679–682 (2022). https://doi.org/10.1038/s41592-022-01488-1.
32. Johnson M, et al, JAX: Autograd and XLA; 2020. https://github.com/google/jax. 2020. Accessed 29 June 2022.
33. Bisong E. Google Colaboratory 2019;p. 59–64. https://doi.org/10.1007/978-1-4842-4470-8_7.
34. Kaggle, Kaggle; 2020. https://www.kaggle.com. 2010. Accessed 29 June 2022.
35. Amazon SageMaker, Amazon SageMaker; 2017. https://aws.amazon.com/sagemaker/. 2017. Accessed 29 June 2022.
36. Moon KR, van Dijk D, Wang Zea. Visualizing structure and transitions in high-dimensional biological data. Nat Biotechnol 2019;37:1482–1492 (2019). https://doi.org/10.1038/s41587-019-0336-3.
37. Boileau P, Hejazi NS, Dudoit S. Exploring high-dimensional biological data with sparse contrastive principal component analysis. Bioinformatics 2020;36(11):3422–3430. https://doi.org/10.1093/bioinformatics/btaa176.
38. Jupyter Docker Stacks. Github; 2015. https://jupyter-docker-stacks.readthedocs.io/en/latest/.

39. Batut B, Hiltemann S, Bagnacani A, Baker D, Bhardwaj V, Blank C, et al. Community-Driven Data Analysis Training for Biology. Cell Systems 2018 jun;6(6):752–758.e1. https://doi.org/10.1016/j.cels.2018.05.012.
40. Kumar A, A Docker-based interactive Jupyterlab powered by GPU for artificial intelligence in Galaxy (Galaxy Training Materials); 2022. https://training.galaxyproject.org/training-material/topics/statistics/tutorials/gpu_jupyter_lab/tutorial.html. 2022. Accessed 29 June 2022.
41. Saeedizadeh N, Minaee S, Kafieh R, Yazdani S, Sonka M. COVID TV-Unet: Segmenting COVID-19 chest CT images using connectivity imposed Unet. Computer Methods and Programs in Biomedicine Update 2021;1:100007. https://www.sciencedirect.com/science/article/pii/S2666990021000069.
42. Jumper J, Evans R, Pritzel A, et al. Highly accurate protein structure prediction with AlphaFold. Nature 2021;596:583–589 (2021). https://doi.org/10.1038/s41586-021-03819-2.
43. Ronneberger O, Fischer P, Brox T. U-Net: Convolutional Networks for Biomedical Image Segmentation. CoRR 2015;abs/1505.04597. http://arxiv.org/abs/1505.04597.
44. Kumar A, Jupyterlab notebooks. GitHub; 2022. https://github.com/anuprulez/gpu_jupyterlab_ct_image_segmentation. 2022. Accessed 29 June 2022.
45. Kumar A, COVID Image segmentation datasets and trained model. Zenodo; 2022. https://doi.org/10.5281/zenodo.6091361.
46. Saeedizadeh N, Minaee S, Kafieh R, Yazdani S, Sonka M, COVID TV-Unet: Segmenting COVID-19 chest CT images using connectivity imposed Unet. Github; 2021. https://github.com/narges-sa/COVID-CT-Segmentation/blob/main/main_TV_Unet_Split1.py. 2021. Accessed 30 June 2022.
47. Steinegger M, Söding J. MMseqs2 enables sensitive protein sequence searching for the analysis of massive data sets. Nat Biotechnol 2017;35:1026–1028 (2017). https://doi.org/10.1038/nbt.3988.
48. Chen L, Kenyon G, Curtin F, Harayama S, Bembenek M, Hajipour G, et al. 4-Oxalocrotonate tautomerase, an enzyme composed of 62 amino acid residues per monomer. J Biol Chem 1992;267(25):17716–21. https://pubmed.ncbi.nlm.nih.gov/1339435/.
49. Mirdita M, Schütze K, Moriwaki Y, et al, ColabFold: making protein folding accessible to all. Github; 2022. https://github.com/sokrypton/ColabFold/blob/main/AlphaFold2.ipynb. 2022. Accessed 30 June 2022.
50. Kumar A, Run long running jupyterlab script. Github; 2022. https://github.com/bgruening/galaxytools/blob/master/tools/jupyter_job/run_jupyter_job.xml. 2022. Accessed 30 June 2022.
51. Kumar A, Custom jupyterlab notebook function to start model training job in Galaxy. Github; 2021. https://github.com/anuprulez/ml-jupyter-notebook/blob/master/galaxy_script_job.py#L43. 2021. Accessed 30 June 2022.
52. Kumar A, Remotely trained image segmentation model. Galaxy; 2022. https://usegalaxy.eu/u/kumara/h/image-segmentation-from-galaxy-jupyterlab. 2022. Accessed 23 August 2022.
53. Kubeflow. Github; 2017. https://github.com/kubeflow/kubeflow.
54. Apache Airflow. Github; 2019. https://github.com/apache/airflow-site.

Supplementary document

Click here to access/download
Supplementary Material
Supplementary Material Docker-based Jupyterlab in Galaxy.pdf

Cover letter

Click here to access/download;Personal Cover;Cover letter for Galaxy jupyterlab project.pdf

Dear Dr. Goodman,

We have developed a Jupyterlab notebook infrastructure in Galaxy Europe that is docker-based and GPU-enabled for developing machine learning, deep learning and data science projects. We would like to submit the associated paper titled "An accessible infrastructure for artificial intelligence using a docker-based Jupyterlab in Galaxy" to GigaScience as a technical note. The infrastructure allows artificial intelligence (AI) researchers to rapidly develop their project prototypes and end-to-end projects artificial intelligence projects. There are many interesting aspects of the work which bring it closer to GigaScience:

- **High-quality software**: The infrastructure supports high-quality softwares such as Tensorflow and Scikit-learn for writing AI models; Bokeh and Seaborn for interactive visualizations; Elyra AI for creating workflow of notebooks and Git for managing entire codebases; Bioblend to access Galaxy's tools, datasets and workflows and GPU computation for faster training of AI models.
- **Publicly available**: The infrastructure is available on Galaxy Europe for registered and authorised Galaxy users. The extra authorisation step is necessary to restrict the non-intended usage of public compute resources (especially GPUs) such as bitcoin mining as experienced by us in the past.
- **Shareability and reproducibility**: Jupyterlab notebooks created using this infrastructure promote sharing of analyses among researchers by making them shareable directly via a URL.
- **Extensibility**: Softwares included in the infrastructure are controlled by a docker file and are independent of Galaxy. To extend it to be used in other scientific fields, then only the new set of packages should be installed and the new container should be updated to the docker hub making the infrastructure extensible to different scientific fields beyond Bioinformatics.
- **Unlimited compute time and remote model training**: Researchers can train AI models for hours or even days without caring about when their programs would terminate. Other similar infrastructures such as Google Colab and Kaggle Kernels restrict users to have limited running time (~12 hours). Also, Galaxy's jupyterlab infrastructure provides an approach to train AI models remotely.
- **Example notebooks and tutorials**: Several example notebooks (1, 2) are available for users to learn different aspects of the entire infrastructure. Also, a tutorial is available on how to use this resource.

Potential reviewers of the manuscript:

- Joshua T. Vogelstein: jovo@jhu.edu
- Kelly D Goodwin: Kelly.Goodwin@noaa.gov
- Stephen R Piccolo: stephen_piccolo@byu.edu
- David Landsman: landsman@ncbi.nlm.nih.gov
- Alexej Abyzov: abyzov.alexej@mayo.edu

The authors agree that there are no competing interests and they have approved the manuscript to be submitted to GigaScience. Moreover, the manuscript has not been sent elsewhere. The first version of the manuscript was uploaded to bioRxiv (preprint server).

On behalf of all authors,

Yours sincerely,

Anup Kumar
