# Supplementary material for: An accessible infrastructure for artificial intelligence using a Docker-based JupyterLab in Galaxy
Source: Gigascience. 2023 Apr 26;12:giad028. doi: 10.1093/gigascience/giad028 (PMC10132306; doi:10.1093/gigascience/giad028)
Supplement: giad028_GIGA-D-22-00220_Revision_1 [file giad028_giga-d-22-00220_revision_1.pdf]

GigaScience

An accessible infrastructure for artificial intelligence using a Docker-based JupyterLab in Galaxy

--Manuscript Draft--

**Manuscript Number:**	GIGA-D-22-00220R1
**Full Title:**	An accessible infrastructure for artificial intelligence using a Docker-based JupyterLab in Galaxy
**Article Type:**	Technical Note
**Funding Information:**	DFG (German Research Foundation) (CIBSS - EXC-2189 - Project ID 390939984) — Dr Rolf Backofen<br>BMBF grant (de.NBI) (031A538A) — Dr Björn Grüning
**Abstract:**	Background Artificial intelligence (AI) programs that train on large datasets require powerful compute infrastructure consisting of several CPUs and GPUs. JupyterLab provides an excellent framework for developing AI programs but it needs to be hosted on such an infrastructure to enable faster training of AI programs using parallel computing.<br><br>Findings An open-source, docker-based, and GPU-enabled JupyterLab infrastructure is developed that runs on the public compute infrastructure of Galaxy Europe consisting of thousands of CPUs, a few GPUs and several petabytes (PB) of storage to rapidly prototype and develop end-to-end AI projects. Using a JupyterLab notebook, long-running AI model training programs can also be executed remotely to create trained models, represented in open neural network exchange (ONNX) format, and other output datasets in Galaxy. Other features include Git integration for version control, the option of creating and executing pipelines of notebooks, and multiple dashboards and packages for monitoring compute resources and visualisation, respectively.<br><br>Conclusions These features make JupyterLab in Galaxy Europe highly suitable for creating and managing AI projects. A recent scientific publication that predicts infected regions in COVID-19 CT scan images is reproduced using various features of the JupyterLab on Galaxy Europe. In addition, ColabFold, a faster implementation of AlphaFold2, is accessed in JupyterLab to predict the 3D structure of protein sequences. JupyterLab is accessible in two ways - one as an interactive Galaxy tool and the other by running the underlying Docker container. In both ways, long-running training can be executed on Galaxy's compute infrastructure. Scripts to create the Docker container are available under MIT license at https://github.com/anuprulez/ml-jupyter-notebook.
**Corresponding Author:**	Anup Kumar<br>Albert-Ludwigs-Universitat Freiburg<br>Freiburg, GERMANY
**Corresponding Author Secondary Information:**	
**Corresponding Author's Institution:**	Albert-Ludwigs-Universitat Freiburg
**Corresponding Author's Secondary Institution:**	
**First Author:**	Anup Kumar
**First Author Secondary Information:**	
**Order of Authors:**	Anup Kumar<br>Gianmauro Cuccuru<br>Björn Grüning

Powered by Editorial Manager® and ProduXion Manager® from Aries Systems Corporation

	Rolf Backofen
**Order of Authors Secondary Information:**	
**Response to Reviewers:**	GIGA-D-22-00220<br>An accessible infrastructure for artificial intelligence using a docker-based Jupyterlab in Galaxy<br>Anup Kumar; Gianmauro Cuccuru; Björn Grüning; Rolf Backofen<br>GigaScience<br><br>Dear Mr. Kumar,<br><br>Your manuscript "An accessible infrastructure for artificial intelligence using a docker-based Jupyterlab in Galaxy" (GIGA-D-22-00220) has been assessed by our reviewers. Although it is of interest, we are unable to consider it for publication in its current form. The reviewers have raised a number of points which we believe would improve the manuscript and may allow a revised version to be published in GigaScience.<br><br><br>Author response:<br>We thank the editor and reviewers for their encouragement, valuable time and useful reviews. We have fixed all the review comments and our responses are as follows.<br><br><br>They also mentioned this manuscript may need a professional editing service, please make sure the language is checked carefully before re-sub.<br>Note: The comments from reviewer1 was uploaded as an attachment.<br><br><br>Author response:<br>Thank you for your comment. We have improved the language of the manuscript.<br><br><br>Their reports, together with any other comments, are below. Please also take a moment to check our website at https://www.editorialmanager.com/giga/ for any additional comments that were saved as attachments.<br><br>In addition, please register any new software application in the bio.tools and SciCrunch.org databases to receive RRID (Research Resource Identification Initiative ID) and biotoolsID identifiers, and include these in your manuscript. Computational workflows should be registered in workflowhub.eu and the DOIs cited in the relevant places in the manuscript. These will facilitate tracking, reproducibility and re-use of your tool.<br><br><br>Author response:<br>We have added the resource to SciCrunch.org (with RRID: SCR_022695) and also to bio.tools (ID: gpu-enabled_docker_container_with_jupyterlab_for_ai, link: https://bio.tools/gpu-enabled_docker_container_with_jupyterlab_for_ai). These have also been mentioned in the manuscript.<br><br><br>If you are able to fully address these points, we would encourage you to submit a revised manuscript to GigaScience. Once you have made the necessary corrections, please submit online at:<br><br>https://www.editorialmanager.com/giga/<br><br>If you have forgotten your username or password please use the "Send Login Details" link to get your login information. For security reasons, your password will be reset.<br><br>Please include a point-by-point within the 'Response to Reviewers' box in the submission system. Please ensure you describe additional experiments that were carried out and include a detailed rebuttal of any criticisms or requested revisions that you disagreed with. Please also ensure that your revised manuscript conforms to the

journal style, which can be found in the Instructions for Authors on the journal homepage. If the data and code has been modified in the revision process please be sure to update the public versions of this too.

The due date for submitting the revised version of your article is 24 Jan 2023.

I look forward to receiving your revised manuscript soon.

Best wishes,

Hongling Zhou
GigaScience
www.gigasciencejournal.com

Reviewer reports:

Reviewer #1:

Review: “An accessible infrastructure for artificial intelligence using a docker-based Jupyterlab in Galaxy”

September 8, 2022

This manuscript introduces the new docker-based JupyterLab framework in Galaxy, describing its core components and demonstrating its use in the reproduction of two analyses. The proposed framework is also thoroughly compared to competitors, like Google’s Colab and Amazon’s SageMaker. This tool is bound to have an impact on the life sciences: it democratizes computational analyses and facilitates reproducibility. I thank the authors for their important work.

Author response:
Thank you for your encouragement and review comments.

However, I think that this technical note should be reviewed for grammatical errors and faulty punctuation. I’ve identified some such issues in the comments below but wasn’t able to address all of
them. Included in the comments are other remarks which, if addressed, could strengthen some key takeaways.

Author response:
We have updated the manuscript based on the review comments. Our responses are as follows.

- The first sentence of the abstract states that AI programs require “powerful compute infrastructure” when applied to large datasets. I think readers would like to know how you qualify
an infrastructure as “powerful”. A brief definition could be included in the second sentence
instead of repeating “. . . hosted on a powerful infrastructure . . . ”.

Author response:
We have removed the redundant "powerful" word and quantified the powerful infrastructure in the Abstract as well as at appropriate places in the manuscript.

- Is it “JupyterLab” or “jupyterlab notebook”? The Project Jupyter site seems to use the former. Based on the documentation, JupyterLab is a web-based user interface that can open
Jupyter notebooks (.ipynb files).

Powered by Editorial Manager® and ProduXion Manager® from Aries Systems Corporation

Author response:
We have used the official name of JupyterLab and have made it consistent in the manuscript.

- The statement “Artificial intelligence (AI) approaches such as machine learning (ML) and
deep learning (DL) . . . ” implies that ML and DL are distinct aspects of AI. This distinction
is insinuated throughout the rest of text. Isn’t DL a subset of ML? I suggest replacing “ML
and DL algorithms” by “ML algorithms” and specifying “DL algorithms” only as needed.

Author response: Yes, we agree that DL is a subset of ML. We have made the required changes in the manuscript and have mostly used the term ML and DL only at the required places.

- I believe there’s a missing comma between “ecosystems” and “enabling” in the first sentence of the Docker container section.

Author response:
We have made this change in the manuscript.

- Consider reformatting “A container runs . . . of the running software.” to “A container runs an isolated environment with minimal interactions between it and the host OS. Running software
in a container is more secure.”

Author response:
This has been fixed in the manuscript.

- Related to the suggestion above: Can you explain why this increased security is necessary? An example might help emphasize the importance of a secure container.

Author response:
Docker containers provide several security benefits for Galaxy's interactive tools as these interactive tools running on Galaxy's remote machines (part of compute infrastructure) let users run computer programs using various programming languages such as Python, R and so on. Executing arbitrary programs on host machines may induce security threats and maliciously written programs can claim the root of these host machines. To prevent such access, Docker containers are being used that maintain minimum interactions with the host operating system. Arbitrary code written inside for example JupyterLab notebook runs within the container instead of directly on the host machine. Moreover, the container runs with a non-root user which provides additional security benefits.

We have added a similar explanation to the manuscript in "Docker container" section.

- I think “Docker container inherits . . . ” should be “The Docker container inherits . . . ”. Same goes for “Docker container is decoupled . . . ”.

Author response:
This has been fixed by using "The Docker container" or "a Docker container"

appropriately at different places in the manuscript.

- Consider reformatting “Moreover, it can easily be extended by installing suitable packages only by adding their appropriate package names in its dockerfile.” to “Moreover, the Docker
container is easily extended: additional software packages can be installed by adding their names to the dockerfile.”

Author response:
This has been fixed in the manuscript.

- Consider replacing “some of the popular ones are” by “including”

Author response:
This has been fixed in the manuscript.

- I believe there’s an unneeded comma between “. . . platform for both” and “rapid prototyping. . . ”.

Author response:
This has been fixed in the manuscript.

- I believe that there’s missing a word in the last sentence of the Features of jupyterlab and notebook infrastructure section: “. . . an H5 file.”

Author response:
This has been fixed in the manuscript.

- “google” and “amazon” should be capitalized.

Author response:
The names of Google, Amazon and Kaggle have been capitalized in the manuscript.

- Consider removing “and non-ideal” from Related infrastructure section.

Author response:
Yes, we agree that this phrase is not suitable. The phrase "and non-ideal" has been removed from the manuscript.

-I believe the comma in “. . . but they come at a price, . . . ” should be replace by a colon.

Author response:
This has been fixed in the manuscript.

-I believe there’s missing a comma between “. . . free of charge” and “similar to colab . . . ”.

Author response:
This has been fixed in the manuscript.

- Why is sharing a sessions's resources across multiple notebooks more useful than operating each notebook in a separate session? Isn't the latter preferable when a notebook causes a
session to crash?

Author response:
This is a good comment. Being able to run multiple notebooks together can help in multiple ways. For example, if there is a notebook that needs the output of two different notebooks (let's assume these two can be executed in parallel), then it would be faster to execute them independently by sharing GPU/CPU resources and making all the intermediate datasets available for the third notebook. In another case, two notebooks can be executed one after another when the second notebook requires datasets produced by the first notebook. A workflow of notebooks can be readily created when multiple notebooks can execute in JupyterLab tabs under one session of a powerful computer. Being able to run notebooks in tabs enables users to run entire projects/codebases consisting of several notebooks and scripts. Moreover, each JupyterLab session on Galaxy Europe requires a GPU and several CPUs and if it is allowed that users run a notebook on this set of resources, it may lead to non-optimized use of valuable compute resources. When many notebooks are updated, they can be committed together to a remote GitHub repository if they can be opened in tabs. Because of such benefits, it is preferable to use several tabs and they all run under one JupyterLab session. When a crashed JupyterLab session is reconnected, all the notebooks in different tabs start working again.

- "deep learning" in the Implementation section should be replaced by "DL" for consistency.

Author response:
This has been fixed in the manuscript.

- I think that readers would find a link to your tool on Galaxy Europe useful: https://usegalaxy.eu/root?tool_id=interactive_tool_ml_jupyter_notebook. The same is true for your tutorial: I think readers would find a URL in the text more easily than in the references.

Author response:
This is a very useful comment, thank you. GPU JupyterLab tool's link has been added to the training material at https://training.galaxyproject.org/training-material/topics/statistics/tutorials/gpu_jupyter_lab/tutorial.html. On Galaxy Europe, the tool can be searched in the tool search using text query such as "gpu jupyterlab".

However, the tool failed to execute on usegalaxy.edu with the following error message: "This tool is restricted to authorized users". I was unable to follow the tutorial. Was this a one-off issue with the Galaxy servers?

Author response:

The tool is open only to authorised users in order to optimise the usage of GPUs on Galaxy Europe's cluster. A Google form (http://usegalaxy.eu/gpu-request) has been created which users can use to apply for using this resource. Once the request is approved by a Galaxy admin made via this online form, the resource will become available to the authorised users within a day or two. More details on how to access this resource are given in Supplementary section S1. Since we do not restrict the usage of GPUs and users can run programs for days and months on these GPUs, it was used by bitcoin miners in the past. To prevent such usage and promote these

resources only for research purposes, we have implemented such an approval method. The steps to apply for this resource:

Please follow these steps to access this infrastructure:

1. Create an account on Galaxy Europe (https://usegalaxy.eu/) using your official university
email id
2. Apply for accessing GPU Jupyterlab using this Google form: http://usegalaxy.eu/gpu-request.
3. Use your official university email id in the Google form. This resource is available only for
research purposes.
4. Wait up to 1-2 days to get the request approved.
5. Once approved, you will be able to run this resource on Galaxy Europe (https://usegalaxy.eu/).
6. If not authorised, then an error message will be shown.
7. Contact us at: contact@usegalaxy.eu if there are issues.

Reviewer #2: Kumar et al. present a Docker-based integration of Jupyter Notebooks in the Galaxy workflow system that can utilize GPUs. This notebook is also available in the Galaxy Europe instance.

I was able to create a Galaxy Europe account, find the newly introduced Galaxy tool and submit a job. However, it remained stuck with the message "This job is waiting to run" and the job info "Stopped" for multiple hours.

Author response:
The tool is open only to authorised users in order to optimise the usage of GPUs on Galaxy Europe's cluster. A Google form (http://usegalaxy.eu/gpu-request) has been created which users can use to apply for using this resource. Once the request is approved by a Galaxy admin made via this online form, the resource will become available to the authorised users within a day or two. More details on how to access this resource are given in Supplementary section S1. Since we do not restrict the usage of GPUs and users can run programs for days and months on these GPUs, it was used by bitcoin miners in the past. To prevent such usage and promote these resources only for research purposes, we have implemented such an approval method. The steps to apply for this resource:

Please follow these steps to access this infrastructure:
1. Create an account on Galaxy Europe (https://usegalaxy.eu/) using your official university
email id
2. Apply for accessing GPU Jupyterlab using this Google form: http://usegalaxy.eu/gpu-request.
3. Use your official university email id in the Google form. This resource is available only for
research purposes.
4. Wait up to 1-2 days to get the request approved.
5. Once approved, you will be able to run this resource on Galaxy Europe (https://usegalaxy.eu/).
6. If not authorised, then an error message will be shown.
7. Contact us at: contact@usegalaxy.eu if there are issues.

I was able to download the docker image and run it on a local server with multiple Nvidia GPUs. This resulted in a running Jupyter Lab, however running the GPU based examples resulted in driver mismatch errors/warnings (pynvml.nvml.NVMLError_LibRmVersionMismatch: RM has detected an NVML/RM version mismatch; kernel version 470.141.3 does not match DSO version 515.65.1 -- cannot find working devices in this configuration). Thus, the examples ran on CPU only. I did not try to resolve this issue and only repeated some examples.

Author response:
NVIDIA and CUDA-related packages are now preinstalled in the container by running nvidia/cuda:11.8.0-cudnn8-runtime-ubuntu20.04 official docker image. In the old version of the Docker container, CUDA packages were manually installed in the Docker which could have caused the "driver mismatch" errors. To improve that we are using official NVIDIA containers (nvidia/cuda) as the base container that has prepackaged CUDA (version 11.8) and CuDNN packages. These are general-purpose containers that recognize GPUs on a host machine when NVIDIA drivers are installed. For deep learning to work with GPUs and TensorFlow, we install only TensorFlow-GPU separately in the container. When the container runs, it automatically recognises NVIDIA GPUs on the host machine.

The authors show two use-cases for the GPU Jupyter Docker and provide a step-by-step tutorial for usage on Galaxy Europe. Shipping machine learning applications that utilize GPUs as Jupyter Notebooks has become popular recently and supporting these through well-known and freely accessible Galaxy servers, such as Galaxy Europe, would be of clear benefit to users. Additionally, it would be very valuable for method developers like me to easily deploy GPU-based methods to Galaxy servers.

Author response:
Thank you for your encouraging thoughts.

Major:
- As mentioned before, I had issues getting a running Jupyter Lab on the Galaxy Europe server. Is this due to a limited number of GPUs or was this due to an error?

Author response:
It is because this tool in the past was available to all users and got exposed to bitcoin miners and they could misuse GPUs for days. Since then, it has been restricted only to authorised users. Moving forward, a Google form (http://usegalaxy.eu/gpu-request) has been implemented for researchers/users who would like to use this resource. Users should fill out this form using their respective university email ID and then this resource is made available for them within a day or two. More details on how to access this resource are given in Supplementary section S1.

Please follow these steps to access this infrastructure:
1. Create an account on Galaxy Europe (https://usegalaxy.eu/) using your official university
email id
2. Apply for accessing GPU Jupyterlab using this Google form:
http://usegalaxy.eu/gpu-request.
3. Use your official university email id in the Google form. This resource is available only for
research purposes.
4. Wait up to 1-2 days to get the request approved.
5. Once approved, you will be able to run this resource on Galaxy Europe (https://usegalaxy.eu/).
6. If not authorised, then an error message will be shown.
7. Contact us at: contact@usegalaxy.eu if there are issues.

- Our ColabFold Multiple Sequence Alignment server currently processes about 10-20k MSAs per day. We do not know how many of these are running on Google Colab or on users' local machines. However, a substantial number of predictions are running inside Google Colab. The authors claim that Google Colab's and Kaggle's resources are scarce. However, generally, users (with either free or pro accounts) are given an instance nearly immediately on Colab. I recognize that it is extremely difficult to compete with these commercial platform providers. However, providing a long-term, freely available and securely funded, platform with ML accelerators would be extremely

beneficial for the whole community. I would like to see a discussion on what GPU resources are currently available to users of Galaxy Europe (and the whole Galaxy Project) and what plans exist to expand these in the future.

Author response:
Galaxy Europe has a large cluster of compute resources that not only comprises computers located in Freiburg, Germany but also at many other locations in Germany as well as other locations in Europe. Currently, only Galaxy's Freiburg cluster has over 8000 CPUs, 60 TB of memory, 5 PB of storage, and 16 GPUs. With the addition of the Pulsar network that connects Galaxy Europe to many different clusters across Europe, the compute power is expected to rise even further. For example, through the Pulsar network, Galaxy can connect to Science and Technology Facilities Council (STFC)'s approximately 30 additional GPUs.

- The size of the docker container (compressed ~10GB, uncompressed ~22GB) seems difficult to sustain.

Author response:
We have made the Docker container approximately 30% smaller (uncompressed size ~13 GB from ~22 GB and compressed size ~7 GB from ~10 GB) by using the official "nvidia/cuda:11.8.0-cudnn8-runtime-ubuntu20.04" as the base container instead of directly installing CUDA packages in the container.

Both keeping up an up-to-date Docker image and ensuring the availability of older images for reproducibility looks difficult to me, especially with such fast moving dependencies such as machine learning frameworks. How do the authors plan to deal with this issue?

Author response:
We have created a versioning system of Docker containers and its interactive Galaxy tool. The current starting version of the tool and the Docker container is at 0.2. As soon as the tools's or Docker container's version is updated in future, the versions of both the Docker container and the tool get updated. Moving forward, it will be possible to work with older versions of the tool and its associated docker container. Many other Galaxy tools maintain a list of all versions which users can choose.

Minor:
- Please highlight the tutorial (https://training.galaxyproject.org/training-material/topics/statistics/tutorials/gpu_jupyter_lab/tutorial.html) on GitHub and inside the container readme (home_page.ipynb). It is very easy to overlook. I also nearly overlooked the example notebook repository (https://github.com/anuprulez/gpu_jupyterlab_ct_image_segmentation). I found it confusing, that I could not find the two shown example use-cases inside the Docker container. I only later figured out that I have to clone the example repository into the running container.

Author response:
This is a good comment. We have added the link to the tutorial to the home page of the Docker container (home_page.ipynb) in the "How to use" section and also to the Docker container Github repository. The notebooks of the use-case repository have been made available in the Docker container.

- The manuscript highlights various workflow methods (elyra, kubeflow, airflow), however it needs clarification on how the Galaxy workflow integration works. I saw that it is possible to give input of another Galaxy output to the tool. I would appreciate a tutorial on how to make the GPU Jupyter Docker into part of a Galaxy workflow with

	multiple tools running. I think the above mentioned tutorials can be expanded to show how the output can be given to the next tool.<br><br>Author response:<br>In the Docker container, we have added a use-case of how to use Elyra AI to make a workflow of JupyterLab notebooks. In the "Elyra" folder there are a few notebooks and a pipeline file that runs these notebooks like a workflow. This pipeline file can be readily executed inside JupyterLab.<br><br>The feature of using the GPU JupyterLab as a tool in a Galaxy workflow has also been integrated. The tool takes an IPython script as input along with datasets produced by other Galaxy tools and produces output(s) as a collection/set of datasets that are created in the executed script. The tutorial shows a step-by-step approach to running this tool in a Galaxy workflow. Tutorial link: https://training.galaxyproject.org/training-material/topics/statistics/tutorials/gpu_jupyter_lab/tutorial.html (Please refer to section: GPU Juyterlab tool in a Galaxy workflow).<br><br>- Docker Hub has introduced many business-model changes such as deleting container images that are rarely used, which poses a challenge for reproducibility. I know that Dr Grüning is involved in the Biocontainers project. I would recommend investigating if it is possible to combine these efforts to make this GPU container and derived containers long term available.<br><br>Author response:<br>We have stored the Docker container in Quay.io (https://quay.io/repository/anupkumar/docker-ml-jupyterlab) as well. Quay.io is supported by RedHat and poses no time restriction on storage. In case Docker stops supporting our containers, we only need to change the container's URL in the JupyterLab tool's XML.<br><br>- The Docker container is explicitly running as a root user, while the manuscript highlights the security benefits of Docker. The cited report by Baset et al. highlights the security benefits and the many security challenges that Docker containers pose. I suggest checking what security best practices for Docker containers are possible to implement, while still allowing GPUs to be exposed to users.<br><br>Author response:<br>Towards making the GPU JupyterLab container more secure, the following measures have been taken. A custom user (non-root) has been created inside the container and this user is permitted to run this resource with no "root" permissions. All "ADD" commands have been replaced with "COPY" commands. To minimize running arbitrary commands inside a container, a trusted image from "nvidia/cuda" official list of images (by NVIDIA Corporation) has been used as the base image to extend this container. While allocating resources to Galaxy's jobs, Linux control groups (also known as CGROUPS) are used to isolate these resources from several running processes. We believe that such measures would greatly improve the security aspects of the GPU JupyterLab project.<br><br>- I recommend revising the manuscript for conciseness, with an additional focus on capitalization of words.<br><br>Author response:<br>We have revised the manuscript improving the text in terms of readability and capitalization wherever required.
**Additional Information:**	
**Question**	**Response**

Are you submitting this manuscript to a special series or article collection?	No
**Experimental design and statistics**<br><br>Full details of the experimental design and statistical methods used should be given in the Methods section, as detailed in our Minimum Standards Reporting Checklist. Information essential to interpreting the data presented should be made available in the figure legends.<br><br>Have you included all the information requested in your manuscript?	Yes
**Resources**<br><br>A description of all resources used, including antibodies, cell lines, animals and software tools, with enough information to allow them to be uniquely identified, should be included in the Methods section. Authors are strongly encouraged to cite Research Resource Identifiers (RRIDs) for antibodies, model organisms and tools, where possible.<br><br>Have you included the information requested as detailed in our Minimum Standards Reporting Checklist?	Yes
**Availability of data and materials**<br><br>All datasets and code on which the conclusions of the paper rely must be either included in your submission or deposited in publicly available repositories (where available and ethically appropriate), referencing such data using a unique identifier in the references and in the "Availability of Data and Materials" section of your manuscript.<br><br>Have you have met the above requirement as detailed in our Minimum	Yes

Standards Reporting Checklist?	

Powered by Editorial Manager® and ProduXion Manager® from Aries Systems Corporation

Main tex
Click here to access/download;LaTeX - Main Document (TeX file);main.tex

OXFORD
(GIGA)n SCIENCE

GigaScience, 0000, 1–7

doi: xx.xxxx/xxxx
Manuscript in Preparation
Technical Note

TECHNICAL NOTE

An accessible infrastructure for artificial intelligence using a Docker-based JupyterLab in Galaxy

Anup Kumar[1,*,†], Gianmauro Cuccuru[1, ‡, †], Björn Grüning[1, §, †] and Rolf Backofen[1,2, ¶, †]

[1]Bioinformatics Group, Department of Computer Science, University of Freiburg, Georges-Koehler-Allee 106, 79110 Freiburg, Germany and [2]Signalling Research Centres BIOSS and CIBSS, University of Freiburg, Schaenzlestr. 18, 79104 Freiburg, Germany

[*]kumara@informatik.uni-freiburg.de https://orcid.org/0000-0002-2068-4695
[‡]gmauro@gmail.com https://orcid.org/0000-0002-5335-545X
[§]gruening@informatik.uni-freiburg.de https://orcid.org/0000-0002-3079-6586
[¶]backofen@informatik.uni-freiburg.de https://orcid.org/0000-0001-8231-3323
[†]Contributions follow the order of the names of authors

Abstract

Background Artificial intelligence (AI) programs that train on large datasets require powerful compute infrastructure consisting of several CPUs and GPUs. JupyterLab provides an excellent framework for developing AI programs but it needs to be hosted on such an infrastructure to enable faster training of AI programs using parallel computing. **Findings** An open-source, docker-based, and GPU-enabled JupyterLab infrastructure is developed that runs on the public compute infrastructure of Galaxy Europe consisting of thousands of CPUs, a few GPUs and several petabytes (PB) of storage to rapidly prototype and develop end-to-end AI projects. Using a JupyterLab notebook, long-running AI model training programs can also be executed remotely to create trained models, represented in open neural network exchange (ONNX) format, and other output datasets in Galaxy. Other features include Git integration for version control, the option of creating and executing pipelines of notebooks, and multiple dashboards and packages for monitoring compute resources and visualisation, respectively. **Conclusions** These features make JupyterLab in Galaxy Europe highly suitable for creating and managing AI projects. A recent scientific publication that predicts infected regions in COVID-19 CT scan images is reproduced using various features of the JupyterLab on Galaxy Europe. In addition, ColabFold, a faster implementation of AlphaFold2, is accessed in JupyterLab to predict the 3D structure of protein sequences. JupyterLab is accessible in two ways - one as an interactive Galaxy tool and the other by running the underlying Docker container. In both ways, long-running training can be executed on Galaxy's compute infrastructure. Scripts to create the Docker container are available under MIT license at https://github.com/anuprulez/ml-jupyter-notebook.

Key words: JupyterLab; Galaxy Europe; Artificial intelligence; Remote model training; ONNX; Elyra AI; GPU; CUDA;

Findings

Background

Bioinformatics comprises many sub-fields such as single-cell, medical imaging, sequencing, proteomics and many more that produce a huge amount of biological data in myriad formats. For example, the single-cell field creates gene expression patterns for each cell that are represented as matrices of real numbers. The medical imaging field generates images of cells and tissues, radiography images such as chest x-rays and computerized tomography (CT) scans. Next-Generation sequencing generates deoxyribonucleic

Compiled on: January 23, 2023.
Draft manuscript prepared by the author.

acid (DNA) sequences that are stored as fasta [1] files. Machine learning (ML) approaches have been vastly used with these datasets [2] for predictive tasks such as medical diagnosis, imputing missing features, augmenting datasets with artificially generated ones, estimating gene expression patterns and many more. To be able to use ML algorithms on such datasets, a robust and efficient compute infrastructure is needed that can serve multiple purposes. They include pre-processing raw datasets to transform them into suitable formats that are compatible with ML algorithms, creating and executing their complex architectures on pre-processed datasets and making trained models and predicted datasets readily available for further analyses. To facilitate such tasks, a complete infrastructure is developed that combines JupyterLab [3], augmented with many useful features, running on the vast public compute infrastructure of Galaxy [4] Europe to perform end-to-end AI analyses on scientific datasets. The infrastructure consists of three major components. First, a Docker container [5] that encapsulates JupyterLab together with multiple packages and plugins used for developing AI programs, data manipulation and visualisation. Section S2 in the supplementary file lists all such packages and plugins with their respective versions. Second, a Galaxy interactive tool [6, 7] that downloads this Docker container to serve JupyterLab online on Galaxy Europe. Third, the compute infrastructure consisting of over 8000 CPUs and a few GPUs and has over 60 TB of memory and 5 PB of disk space. From this collection, each session of JupyterLab receives over 20 gigabytes (GB) of memory (RAM), in addition to 15 GB of memory provided by one GPU. The disk space is 1 TB and there are 7 CPUs.

Docker container

Docker [8] containers are popular for shipping packaged software as complete ecosystems, enabling them to be reproducible in a platform-independent manner. Software executing inside a Docker container is abstracted from the host operating system (OS) as most of the requirements necessary for them to run successfully are already configured inside its container. A container runs in an isolated environment having minimal interactions with the host OS. Therefore, running softwares in a container is more secure. Using a Docker container leverages the security benefits necessary for online program editing softwares executing arbitrary code. Arbitrary code may contain some malicious script posing security risks. Using Docker containers can minimise their consequences. Further, in our Docker container, a non-root user is created that can execute and manage projects inside the JupyterLab environment which further minimises security risks. In addition to minimising security risks, Docker containers provide performance benefits compared to running programs on a virtual machine [9]. Motivated by such benefits, a Docker container is used in this project to encapsulate JupyterLab along with many useful packages such as Git [10], Elyra AI [11], TensorFlow-GPU [12], Scikit-learn [13], ONNX [14], and many others. The Docker container inherits many packages such as CUDA [15], NumPy [16], SciPy [17] and a few more from its base container, nvidia/cuda [18], and augments them with many other packages suitable for ML, data manipulation and visualisation. The Docker container is decoupled from Galaxy and can independently be executed for serving JupyterLab with the same set of packages on a different compute infrastructure or any personal computer (PC) or laptop having approximately 25 GB of disk space. Moreover, the Docker container is easily extended by adding the names of packages to the dockerfile [19]. Adding new packages requires the container to be rebuilt and added to Docker hub [20]. The approach to extending the Docker container is discussed in the Methods section.

JupyterLab

JupyterLab is a web-based, robust editor used for varied purposes such as data science, scientific computing and ML. It is a program editor that supports more than 40 programming languages including Python, R, Julia and Scala. Python is one of the most popular languages used by researchers for performing numerous scientific and predictive analyses. Therefore, it is used as the programming language in Galaxy's JupyterLab because many popular packages such as Scikit-learn and TensorFlow for ML, data manipulation packages such as Pandas, visualisation packages such as Seaborn [21], Matplotlib [22], Bokeh [23] and many others are readily available as Python packages. Moreover, the extensible architecture of JupyterLab makes it possible to add many external packages as its plugins such as Git, Elyra AI, dashboards and many others that have a user interface (UI) as necessary components. Such editors, integrated with several useful packages, provide a favourable platform for rapid prototyping and end-to-end development and management of AI projects. To harness the benefits of JupyterLab, it has been used as the editor for the interactive tool in Galaxy.

Features of JupyterLab infrastructure

Many features such as easy accessibility, support of a wide variety of programming languages on JupyterLab, and extensibility to install useful plugins make it a desirable editor for researchers for creating project prototypes rapidly. Many such features have been integrated into our JupyterLab infrastructure that is served online on Galaxy Europe enabling researchers to create prototypes and end-to-end AI projects (Figure 1). A few important features are discussed here. To allow GPU computation from JupyterLab, TensorFlow-GPU interacts with Nvidia GPU hardware using another software, CUDA, when the compute resource has GPU(s) for accelerating ML programs. Faster execution of ML programs is one of the significant features of JupyterLab hosted on Galaxy Europe. However, if the hosted machine on which a Docker container runs does not have GPUs, then the program in JupyterLab relies on CPUs. Other useful features include ONNX for transforming trained TensorFlow and Scikit-learn models to ONNX models, Open-CV [24] and Scikit-image [25] for processing images, Nibabel [26] for reading image files stored as ".nii", Bioblend [27] for accessing Galaxy's datasets, histories and workflows in a JupyterLab notebook and visualisation packages such as Bqplot [28] and Bokeh for plotting interactive charts, Voilà [29] for displaying output cells of a JupyterLab notebook in a different tab, dashboard such as NVDashboard [30] for monitoring GPU usage and performance. Support for file extension such as H5 [31], efficient for storing matrices, enables ML researchers to save model weights and input datasets for AI algorithms. Other packages such as ColabFold [32] together with JAX [33] are used for predicting 3D structures of proteins which are discussed in the Results section. In addition, it is possible to create a long-running training job that runs remotely and stores trained models and output datasets permanently in a newly created Galaxy history. The trained model is saved as an ONNX file and tabular datasets are in an H5 file. It is discussed in the Methods section.

Related infrastructure

There are a few other infrastructures available, free and commercial, that offer JupyterLab or similar environments for developing data science and AI projects. A few popular ones are Google Colab [34], Kaggle Kernel [35] and Amazon Sagemaker [36]. Google Colab is partially free and offers an online editor similar to JupyterLab. The free version of Google Colab offers dynamic compute resources. The disk space is around 70 GB and the memory (RAM) is around 12 GB. These resources are scarce for AI projects that deal with high-dimensional scientific data [37, 38]. In addition, these resources

offered by Google Colab are variable and depend on a user's past usage. More compute resources are assigned to those users that have used less in the past for a more equitable sharing of resources. Moreover, there is a limitation of only 12 hours of running time that may prove to be insufficient for training AI models on large datasets. However, Google Colab pro and pro+ offer better compute resources but they come at a price; EUR 9.25 and EUR 42.25 per month, respectively. In contrast, Kaggle Kernel is free of charge, but similar to Colab, their computing resources are scarce. The total disk space is approximately 73 GB and RAM is 16 GB for a CPU-based kernel. For the GPU-based kernel, the disk space is of the same size as that of the CPU-based kernel but the RAM of the CPU decreases to 13 GB. An additional RAM of 15 GB is added through a GPU and computation time is limited to 30 hours a week. It also supports TPUs but the computation time is further limited to only 20 hours a week. Amazon Sagemaker is also a commercial software for developing AI algorithms that is free of charge but only for 2 months. Overall, these notebook infrastructures do not offer unrestricted compute resources free of charge. In addition, compute resources offered free of charge are insufficient for training AI models on high-dimensional scientific datasets. To address the drawbacks of these notebook infrastructures and provide researchers and users with large compute resources more reliably, Galaxy JupyterLab infrastructure offers 1 terabyte (TB) of disk space and unlimited computation time on 1 GPU and 7 CPUs per session. RAM for GPU is around 15 GB and for CPUs is 20 GB (Table 1). The offered resources for JupyterLab running in Galaxy stay constant and are independent of the user's past usage. To make it more useful, JupyterLab opens a tab for each notebook that allows researchers to develop and execute several notebooks inside the same session of the allotted compute resource rather than having them connect to a different session for each notebook as in Google Colab and Kaggle Kernel.

Implementation

JupyterLab infrastructure has been developed in two stages. First, a Docker container is created containing all the necessary packages such as JupyterLab itself, CUDA from the base Docker image [39], TensorFlow, Scikit-learn, ONNX and many more. The Docker container is inherited from a base container that has all the necessary CUDA packages installed for working with NVIDIA GPUs. Many packages are added to the Docker container with their compatible versions. Compatible packages for CUDA, CUDA DNN and TensorFlow are necessary so that they together interact with the GPU on the host machine for accelerating ML programs. The versions of all packages installed in the Docker container are listed in Supplementary section S5. Second, the container can be downloaded to any powerful compute infrastructure and JupyterLab can be served in an internet browser via the URL that it generates. In addition, to run this container in Galaxy, an interactive tool is created that downloads this container on a remote compute infrastructure and generates a URL used to run JupyterLab in an internet browser. The architecture of JupyterLab infrastructure in Galaxy is shown in Figure 1. The running instance of JupyterLab in Galaxy contains a default IPython notebook that summarises several of its features. Further, there are other notebooks available, each describing a feature of JupyterLab with code examples such as how to create ONNX models for Scikit-learn and TensorFlow classifiers, how to connect to Galaxy using Bioblend, how to create interactive plots using Bqplots and how to create a pipeline of notebooks using Elyra AI. In addition, the notebooks explaining the use-cases are also available in the Docker container. To access JupyterLab in Galaxy Europe, a ready-to-use hands-on Galaxy training network (GTN) [40] tutorial [41] is developed that shows all the steps such as opening the notebook, using Git to clone a code repository from GitHub, sending long-running training jobs to a remote Galaxy cluster, and how this notebook can be used as a tool in a Galaxy workflow. The approach of remote model training is explained in the Methods section. The two use-cases are also discussed in the tutorial along with their respective notebooks. The steps to access this resource on Galaxy Europe are elaborated in Supplementary section S1.

Results

JupyterLab infrastructure in Galaxy Europe is used to reproduce the results of two recent scientific publications. They demonstrate its robustness and usefulness to develop ML models using COVID CT scan images [42] and predict the 3D structure of proteins using ColabFold, a faster implementation of AlphaFold2 [43].

COVID-19 CT scan image segmentation

In [42], COVID-19 CT scan images have been used to develop and train an ML model architecture that predicts COVID-19 infected regions in those images with high accuracy. An open-source implementation of the work is available that trains a Unet DL architecture [44] that distinguishes between normal and infected regions in CT scan images. Scripts of this implementation are adapted and executed on Galaxy's JupyterLab infrastructure. Adaption only involves the transformation of all CT scan images, used in [42], into an H5 file so that they can directly be used as an input to the Unet architecture defined in a notebook in [45]. All the notebooks available in [45] are also available in the Docker container in the "usecases" directory. A composite H5 file [46] is created using a script [47] that contains multiple datasets inside and each dataset is a real-valued matrix corresponding to the training, test and validation sets as used in [42]. The entire analysis of [42] can be reproduced using multiple notebooks in [45]. They achieve similar precision and recall (approximately 0.98) metrics as mentioned in [42]. In [45], the first notebook (1_fetch_datasets.ipynb) downloads the input dataset as an H5 file. Additionally, it also downloads the trained ONNX model. The second notebook (2_create_model_and_train.ipynb) creates and trains a Unet model on the training dataset extracted from the H5 file. Training, accelerated by GPU, for 10 iterations over the entire training dataset finishes in a few minutes. The third notebook (3_predict_masks.ipynb) extracts the test dataset and predicts infected regions of the CT scan images in the test dataset using the trained model created by the second notebook. Figure 2 shows the comparison of ground truth infected regions in the second column and the predicted infected regions in the third column. A few original CT scan images from the test dataset are shown in the first column of Figure 2.

Predict 3D structure of proteins using ColabFold

AlphaFold2 has made a breakthrough in predicting the 3D structures of proteins with outstanding accuracy. However, due to their large database size (a few TB), it is not easily accessible to researchers. Therefore, a few approaches have been developed that replace the time-consuming steps of AlphaFold2 with slightly different steps but predict the 3D structure of proteins with similar accuracy while consuming less memory and time. One such approach is ColabFold which replaces a large database search in AlphaFold2 for finding homologous sequences by a significantly (40-60 times) faster MMseqs2 API [48] call to generate input features based on the query protein sequence. ColabFold's prediction of 3D structures in batches is approximately 90 times faster. It is integrated into the Docker container [5] by adding two packages - ColabFold and GPU-enabled JAX which is a just-in-time compiler for making mathematical transformations. "7_ColabFold_MMseq2.ipynb" notebook in [45] predicts the 3D structure of a protein sequence using ColabFold by making use of the AlphaFold2 pre-trained weights. Figure 3 shows the 3D structure of 4Oxalocrotonate_Tautomerase

Table 1. Comparison of Galaxy JupyterLab with other notebook infrastructures

Indicators/Infrastructures	Google Colab [34]	Kaggle Kernel [35]	Galaxy JupyterLab
Memory/Disk space (GB)	12/70	16/73	**20/1000**
GPU/TPU	Yes/**Yes**	Yes/**Yes**	**Yes**/No
Max usage time (Hours)	12	12, 30 hrs of GPU/week, 20 hrs of TPU/week	**No time restriction** on GPU usage, notebook sessions and job execution
Dynamic compute resources	Yes	Yes	**Fixed and guaranteed**
Remote model training	No	No	**Yes**
Run multiple notebooks (as tabs) in one session	No	No	**Yes**

[49], a protein sequence of length 62, along with its side chains. This 3D structure is extremely similar to the structure predicted by the Jupyter notebook [50] from ColabFold [32].

Methods

Remote model training

For large datasets, ML model training may need several hours or even days. In such cases, it would be cumbersome to keep JupyterLab open in a browser's tab till the training finishes. Therefore, another Galaxy tool [51] is developed to enable researchers to send long-running training jobs to a remote Galaxy cluster. The tool can be executed from JupyterLab using a custom Python function [52], part of each JupyterLab notebook, that takes input datasets and a training script as input parameters. The input datasets to be used for training, testing and validation must be provided in H5 format. It allows the standardisation of input data format for AI models that train on matrices in JupyterLab. Input data to an AI model can be in multiple formats such as images, genomic sequences or gene expression patterns. H5 files can be created using any of these data formats and fed to the AI model in JupyterLab. Long-running training happens in a remote Galaxy cluster as a regular Galaxy job. Upon completion of the job, the resulting datasets and the trained model become available in a newly created Galaxy history [53]. The trained model and other resulting datasets can either be downloaded to a local machine or imported from the Galaxy history for further analysis using "get" (for fetching datasets directly into a JupyterLab notebook from Galaxy history) and "put" (for saving datasets directly into a Galaxy history from JupyterLab notebook) methods into a JupyterLab notebook [54]. In [45], a few notebooks are available that showcase the approach of remote model training. Notebook "4_create_model_and_train_remote.ipynb" contains code for developing and training a Unet architecture. Notebook "5_run_remote_training.ipynb" executes the previous notebook on a Galaxy cluster remotely after creating a Galaxy history and then uploading the script extracted from "4_create_model_and_train_remote.ipynb" notebook and input datasets. Custom Python function, "run_script_job", creates a Galaxy history using Bioblend and then uploads the datasets to the same history. After the upload is finished, the Python script from the specified notebook is executed dynamically. It trains an ML model on the uploaded datasets to create a model and saves it as an ONNX file in the Galaxy history. Using "6_predict_masks_remote_model.ipynb" notebook from [45], the trained model can be downloaded from the Galaxy history and used for predicting infected regions of the CT scan images of the test dataset. A significant advantage of training ML models remotely is that researchers don't have to keep the JupyterLab session running as long as the model is being trained as model training becomes decoupled from JupyterLab. Using such a feature, ML models that take several hours or even days to train can be conveniently trained.

Extend Docker container

The customised Docker container developed as shown in Figure 1 can be easily extended to have more or different packages. To update the container, a package or a list of new packages should be added to the dockerfile and then the new container should be built and pushed to Docker hub [5]. After pushing the new container, when Galaxy's JupyterLab interactive tool is accessed on Galaxy Europe, it downloads the new container and all the newly added packages are available in JupyterLab. Similarly, versions of existing packages can be updated or existing packages can be removed if no longer needed. The simplified extension procedure of the entire infrastructure incurs low maintenance costs as any change to this entire infrastructure is reflected only in the container without updating Galaxy's codebase. In addition, packages can also be added or updated using "pip" in any JupyterLab notebook. But, such changes remain as long as the JupyterLab session runs as they don't update the underlying Docker container.

Collaborative notebooks

Notebooks created in Galaxy's JupyterLab infrastructure can instantly be shared with other researchers and collaborators only by sharing the public URL of a notebook. Researchers and users that share a notebook can collaborate on the same notebook without having to store it anywhere as it is directly served by Galaxy Europe.

Workflow of notebooks

Resembling many tools in Galaxy, JupyterLab can also be used in any Galaxy workflow where it can accept datasets from different tools and then executes an IPython notebook to process the input datasets. It outputs a collection of datasets which can further be used by other Galaxy tools [41]. In addition, using the Elyra AI package, a workflow of notebooks can be created using existing notebooks in a JupyterLab session and executed as one unit of software similarly as Galaxy workflows are created using several tools. It is possible to execute such workflows of notebooks on the same compute resource on which the JupyterLab session runs. In addition, a few other services such as Kubeflow [55] or Apache Airflow [56] can also be used to deploy, run and manage such workflows on a cloud but are not explored in our work.

Summary

JupyterLab is integrated as an interactive tool in Galaxy Europe running on a public and powerful compute infrastructure comprising several CPUs and GPUs having large memories and disk space. A Docker container is created that wraps JupyterLab along with packages such as TensorFlow-GPU, Scikit-learn, Pandas and many others to provide a robust architecture for the development and management of projects in ML and data science. Remote model training makes it convenient to run multiple analyses in parallel in different Galaxy jobs by executing the same Galaxy tool. The resulting datasets of each job become available in different Galaxy histories. Features such as Git integration are useful for managing entire code repositories on GitHub and Elyra AI for creating pipelines of notebooks working as one software unit. All notebooks created by a user run on the same session of JupyterLab in different tabs. The entire infrastructure of JupyterLab is readily accessible through Galaxy Europe. In contrast to commercial infrastructures that host editors similar to JupyterLab and offer powerful and reliable compute only through paid subscriptions, this infrastructure provides large compute resources free of cost which are invariant to usage and has an unlimited usage time while ensuring a constant amount of compute resources across successive usages.

Availability of supporting source code and requirements

Project name: GPU-enabled Docker container with JupyterLab for artificial intelligence
Project home page: https://github.com/anuprulez/ml-jupyter-notebook
Galaxy interactive tool: https://github.com/usegalaxy-eu/galaxy/blob/release_22.05_europe/tools/interactive/interactivetool_ml_jupyter_notebook.xml
Operating system: Linux
Programming languages: Python, XML, Docker, Bash
License: MIT License
RRID: SCR_022695
Biotools ID: gpu-enabled_docker_container_with_jupyterlab_for_ai

Additional files

Supplementary Material: An accessible infrastructure for artificial intelligence using a docker-based JupyterLab in Galaxy.

List of abbreviations

AI: Artificial intelligence; CT: Computerised tomography; CUDA: Compute unified device architecture; DL: Deep learning; DNA: Deoxyribonucleic acid; EUR: Euro; GPU: Graphical processing unit; GB: Gigabyte; GTN: Galaxy training network; JAX: Accelerated linear algebra; ML: Machine learning; ONNX: Open neural network exchange; OS: PC: Personal computer; RAM: Random-access memory; TB: Terabyte; PB: Petabyte; UI: User interface; URL: Uniform resource locator;

Competing Interests

The authors declare that they have no competing interests.

Ethics approval and consent to participate

Not applicable

Consent for publication

Not applicable

Funding

This work was supported by the German Research Foundation (DFG) under Germany's Excellence Strategy (CIBSS - EXC-2189 - Project ID 390939984) and German Federal Ministry of Education and Research (BMBF grant 031A538A de.NBI).

Authors' contributions

A.K. developed the project and wrote the manuscript. G.C. deployed the project on Galaxy Europe. B.G. devised the idea of the project and helped in creating the resource's access method. R.B. provided the necessary support for the entire project. All authors contributed to and approved the manuscript.

Acknowledgements

We thank Daniel Blankenberg for his suggestions to improve the Docker container. In addition, we thank Galaxy Europe team for running and maintaining the project.

References

1. Pearson W, et al, The FASTA package - protein and DNA sequence similarity searching and alignment programs. GitHub; 2016. https://github.com/wrpearson/fasta36. 2016. Accessed 30 June 2022.
2. Kumar I, Singh SP, Shivam. Chapter 26 - Machine learning in bioinformatics. Academic Press 2022;p. 443–456. https://www.sciencedirect.com/science/article/pii/B9780323897754000201.
3. Kluyver T, Ragan-Kelley B, Pérez F, Granger B, Bussonnier M, Frederic J, et al.; IOS Press. Jupyter Notebooks—a publishing format for reproducible computational workflows 2016;p. 87.
4. Afgan E, Baker D, Batut B, et al. The Galaxy platform for accessible, reproducible and collaborative biomedical analyses: 2018 update. Nucleic Acids Research 2018;46(W1):W537–W544. doi:10.1093/nar/gky379.
5. Kumar A, Container for machine learning and deep learning in Jupyter notebook. Docker; 2021. https://hub.docker.com/r/anupkumar/docker-ml-jupyterlab. 2021. Accessed 29 June 2022.
6. Galaxy Europe, Live instance of the European Galaxy server. Galaxy Europe; 2019. https://live.usegalaxy.eu/. 2019. Accessed 30 June 2022.
7. Kumar A, et al, GPU enabled Interactive Jupyter Notebook for Machine Learning; 2021. https://github.com/usegalaxy-eu/galaxy/blob/release_22.01_europe/tools/interactive/interactivetool_ml_jupyter_notebook.xml.
8. Merkel D. Docker: lightweight linux containers for consistent development and deployment. Linux journal 2014;2014(239):2.
9. Baset et al , Docker and Container Security White Paper; 2016. https://dominoweb.draco.res.ibm.com/reports/rc25625.pdf.
10. Collonval Fea, A JupyterLab extension for version control using Git.;. https://github.com/jupyterlab/jupyterlab-git. 2017. Accessed 29 June 2022.
11. Resende Lea, Elyra is a set of AI-centric extensions to JupyterLab Notebooks.;. https://github.com/elyra-ai/elyra. 2018. Accessed 29 June 2022.
12. Abadi M, Agarwal A, Barham P, Brevdo E, Chen Z, Citro C, et al.,

TensorFlow: Large-Scale Machine Learning on Heterogeneous Systems; 2015. https://www.tensorflow.org/, software available from tensorflow.org.
13. Pedregosa F, Varoquaux G, Gramfort A, Michel V, Thirion B, Grisel O, et al. Scikit-learn: Machine Learning in Python. Journal of Machine Learning Research 2011;12:2825–2830.
14. Bai J, Lu F, Zhang K, et al, ONNX: Open Neural Network Exchange. GitHub; 2019. https://github.com/onnx/onnx. 2019. Accessed 29 June 2022.
15. NVIDIA, Vingelmann P, Fitzek FHP, CUDA, release: 10.2.89; 2020. https://developer.nvidia.com/cuda-toolkit.2020.Accessed 29 June 2022.
16. Harris CR, Millman KJ, van der Walt SJ, Gommers R, Virtanen P, Cournapeau D, et al. Array programming with NumPy. Nature 2020 Sep;585(7825):357–362. https://doi.org/10.1038/s41586-020-2649-2.
17. Virtanen P, Gommers R, Oliphant TE, Haberland M, Reddy T, Cournapeau D, et al. SciPy 1.0: Fundamental Algorithms for Scientific Computing in Python. Nature Methods 2020;17:261–272.
18. NVIDIA Corporation, CUDA and cuDNN images from gitlab.com/nvidia/cuda. Docker; 2014. https://hub.docker.com/r/nvidia/cuda. 2014. Accessed 29 June 2022.
19. Kumar A, Jupyter container used for Data Science and Tensorflow. GitHub; 2021. https://github.com/anuprulez/ml-jupyter-notebook/blob/master/Dockerfile. 2021. Accessed 29 June 2022.
20. Docker Hub. Docker; 2013. https://hub.docker.com/.
21. Waskom ML. seaborn: statistical data visualization. Journal of Open Source Software 2021;6(60):3021. https://doi.org/10.21105/joss.03021.
22. Hunter JD, Matplotlib: A 2D graphics environment. IEEE COMPUTER SOC; 2007.
23. Bokeh Development Team, Bokeh: Python library for interactive visualization. GitHub; 2018. https://bokeh.pydata.org/en/latest/.
24. Bradski G. The OpenCV Library. Dr Dobb's Journal of Software Tools 2000;.
25. Van der Walt S, Schönberger JL, Nunez-Iglesias J, Boulogne F, Warner JD, Yager N, et al. scikit-image: image processing in Python. PeerJ 2014;2:e453.
26. Brett M, Markiewicz CJ, Hanke M, Côté MA, Cipollini B, McCarthy P, et al., nipy/nibabel: 3.2.2. Zenodo; 2022. https://doi.org/10.5281/zenodo.6617121.
27. Sloggett C, Goonasekera N, Afgan E. BioBlend: automating pipeline analyses within Galaxy and CloudMan. Bioinformatics 2013;29(13):1685–1686. https://doi.org/10.1093/bioinformatics/btt199.
28. Corlay, S and et al , 2-D plotting library for Project Jupyter. GitHub; 2015. https://github.com/bqplot/bqplot. 2015. Accessed 29 June 2022.
29. Tuloup, J and et al , Rendering of live Jupyter notebooks with interactive widgets. GitHub; 2018. https://github.com/voila-dashboards/voila. 2018. Accessed 29 June 2022.
30. Tomlinson, J and et al , A JupyterLab extension for displaying GPU usage dashboards. GitHub; 2021. https://github.com/rapidsai/jupyterlab-nvdashboard. 2021. Accessed 29 June 2022.
31. The HDF Group, Hierarchical Data Format, version 5; 1997-2022. https://www.hdfgroup.org/HDF5/. 1997. Accessed 29 June 2022.
32. Mirdita M, Schütze K, Moriwaki Y, et al. ColabFold: making protein folding accessible to all. Nat Methods 2022;19:679–682 (2022). https://doi.org/10.1038/s41592-022-01488-1.
33. Johnson M, et al, JAX: Autograd and XLA; 2020. https://github.com/google/jax. 2020. Accessed 29 June 2022.
34. Bisong E. Google Colaboratory 2019;p. 59–64. https://doi.org/10.1007/978-1-4842-4470-8_7.
35. Kaggle, Kaggle; 2020. https://www.kaggle.com. 2010. Accessed 29 June 2022.
36. Amazon SageMaker, Amazon SageMaker; 2017. https://aws.amazon.com/sagemaker/. 2017. Accessed 29 June 2022.
37. Moon KR, van Dijk D, Wang Zea. Visualizing structure and transitions in high-dimensional biological data. Nat Biotechnol 2019;37:1482–1492 (2019). https://doi.org/10.1038/s41587-019-0336-3.
38. Boileau P, Hejazi NS, Dudoit S. Exploring high-dimensional biological data with sparse contrastive principal component analysis. Bioinformatics 2020;36(11):3422–3430. https://doi.org/10.1093/bioinformatics/btaa176.
39. nvidia/cuda:11.8.0-cudnn8-runtime-ubuntu20.04. Nvidia/Docker; 2014. https://hub.docker.com/layers/nvidia/cuda/11.8.0-cudnn8-runtime-ubuntu20.04/images/sha256-74b166e2091bb705e9ada685dffe79930612c725669bc87e01125b5245d13f context=explore.
40. Batut B, Hiltemann S, Bagnacani A, Baker D, Bhardwaj V, Blank C, et al. Community-Driven Data Analysis Training for Biology. Cell Systems 2018 jun;6(6):752–758.e1. https://doi.org/10.1016/j.cels.2018.05.012.
41. Kumar A, A Docker-based interactive Jupyterlab powered by GPU for artificial intelligence in Galaxy (Galaxy Training Materials); 2022. https://training.galaxyproject.org/training-material/topics/statistics/tutorials/gpu_jupyter_lab/tutorial.html. 2022. Accessed 23 January 2023.
42. Saeedizadeh N, Minaee S, Kafieh R, Yazdani S, Sonka M. COVID TV-Unet: Segmenting COVID-19 chest CT images using connectivity imposed Unet. Computer Methods and Programs in Biomedicine Update 2021;1:100007. https://www.sciencedirect.com/science/article/pii/S2666990021000069.
43. Jumper J, Evans R, Pritzel A, et al. Highly accurate protein structure prediction with AlphaFold. Nature 2021;596:583–589 (2021). https://doi.org/10.1038/s41586-021-03819-2.
44. Ronneberger O, Fischer P, Brox T. U-Net: Convolutional Networks for Biomedical Image Segmentation. CoRR 2015;abs/1505.04597. http://arxiv.org/abs/1505.04597.
45. Kumar A, Jupyterlab notebooks. GitHub; 2022. https://github.com/anuprulez/gpu_jupyterlab_ct_image_segmentation. 2022. Accessed 29 June 2022.
46. Kumar A, COVID Image segmentation datasets and trained model. Zenodo; 2022. https://doi.org/10.5281/zenodo.6091361.
47. Saeedizadeh N, Minaee S, Kafieh R, Yazdani S, Sonka M, COVID TV-Unet: Segmenting COVID-19 chest CT images using connectivity imposed Unet. GitHub; 2021. https://github.com/narges-sa/COVID-CT-Segmentation/blob/main/main_TV_Unet_Split1.py. 2021. Accessed 30 June 2022.
48. Steinegger M, Söding J. MMseqs2 enables sensitive protein sequence searching for the analysis of massive data sets. Nat Biotechnol 2017;35:1026–1028 (2017). https://doi.org/10.1038/nbt.3988.
49. Chen L, Kenyon G, Curtin F, Harayama S, Bembenek M, Hajipour G, et al. 4-Oxalocrotonate tautomerase, an enzyme composed of 62 amino acid residues per monomer. J Biol Chem 1992;267(25):17716–21. https://pubmed.ncbi.nlm.nih.gov/1339435/.
50. Mirdita M, Schütze K, Moriwaki Y, et al, ColabFold: making protein folding accessible to all. GitHub; 2022. https://github.com/sokrypton/ColabFold/blob/main/AlphaFold2.ipynb. 2021. Accessed 30 June 2022.
51. Kumar A, Run long running jupyterlab script. Github; 2022. https://github.com/bgruening/galaxytools/blob/master/tools/jupyter_job/run_jupyter_job.xml. 2022. Accessed 30 June 2022.
52. Kumar A, Custom jupyterlab notebook function to

start model training job in Galaxy. Github; 2021. https://github.com/anuprulez/ml-jupyter-notebook/blob/master/galaxy_script_job.py#L43. 2021. Accessed 30 June 2022.
53. Kumar A, Remotely trained image segmentation model. Galaxy; 2022. https://usegalaxy.eu/u/kumara/h/image-segmentation-from-galaxy-jupyterlab. 2022. Accessed 23 August 2022.
54. Galaxy's Interactive Environments. GitHub; 2016. https://github.com/bgruening/docker-jupyter-notebook/blob/master/default_notebook.ipynb.
55. Kubeflow. Github; 2017. https://github.com/kubeflow/kubeflow.
56. Apache Airflow. Github; 2019. https://github.com/apache/airflow-site.

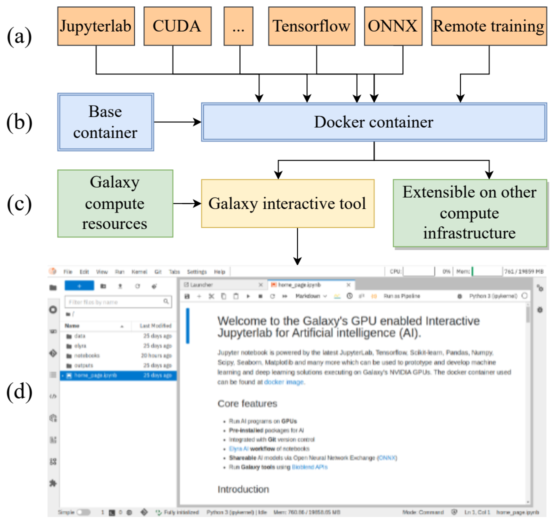

Figure 1. Architecture of Galaxy's JupyterLab. Part (a) shows packages and features wrapped inside a Docker container. Part (b) shows a base Docker container [39] from which the customised container [5] is derived. In part (c), Galaxy's interactive tool downloads the customised container. The customised Docker container can also be hosted on a different compute infrastructure. Part (d) shows Galaxy's JupyterLab.

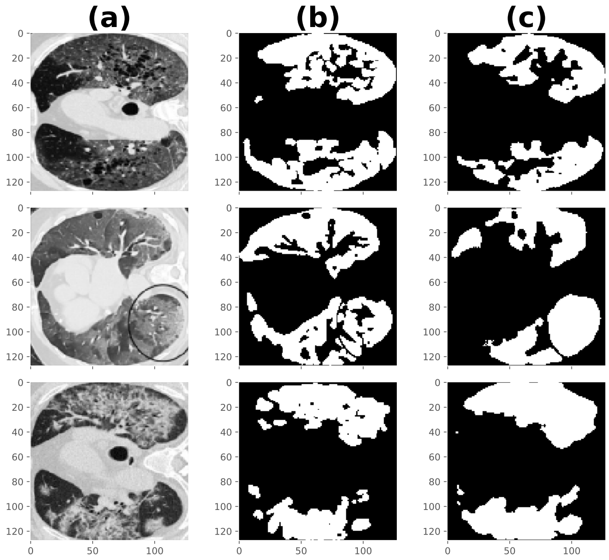

Figure 2. Figure shows original CT scan images in column (a), corresponding ground-truth masks of original CT scan images in column (b) and the predicted masks in column (c). Masks are COVID-19 infected regions in the corresponding CT scan images. The ground-truth and predicted masks show high similarity [42].

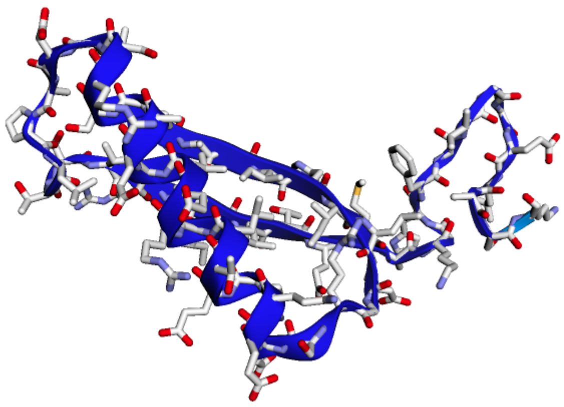

Figure 3. Figures shows a 3D structure of 4 Oxalocrotonate_Tautomerase enzyme (protein) [49] predicted by ColabFold

Manuscript Reference PDF
Click here to access/download;Manuscript;GPU_Jupyterlab_Galaxy_GigaSc

OXFORD (GIGA)n SCIENCE

GigaScience, 0000, 1–7

doi: xx.xxxx/xxxx
Manuscript in Preparation
Technical Note

TECHNICAL NOTE

An accessible infrastructure for artificial intelligence using a Docker-based JupyterLab in Galaxy

Anup Kumar[1,*,†], Gianmauro Cuccuru[1,‡,†], Björn Grüning[1,§,†] and Rolf Backofen[1,2,¶,†]

[1]Bioinformatics Group, Department of Computer Science, University of Freiburg, Georges-Koehler-Allee 106, 79110 Freiburg, Germany and [2]Signalling Research Centres BIOSS and CIBSS, University of Freiburg, Schaenzlestr. 18, 79104 Freiburg, Germany

[*]kumara@informatik.uni-freiburg.de https://orcid.org/0000-0002-2068-4695
[‡]gmauro@gmail.com https://orcid.org/0000-0002-5335-545X
[§]gruening@informatik.uni-freiburg.de https://orcid.org/0000-0002-3079-6586
[¶]backofen@informatik.uni-freiburg.de https://orcid.org/0000-0001-8231-3323
[†]Contributions follow the order of the names of authors

Abstract

Background Artificial intelligence (AI) programs that train on large datasets require powerful compute infrastructure consisting of several CPUs and GPUs. JupyterLab provides an excellent framework for developing AI programs but it needs to be hosted on such an infrastructure to enable faster training of AI programs using parallel computing. **Findings** An open-source, docker-based, and GPU-enabled JupyterLab infrastructure is developed that runs on the public compute infrastructure of Galaxy Europe consisting of thousands of CPUs, a few GPUs and several petabytes (PB) of storage to rapidly prototype and develop end-to-end AI projects. Using a JupyterLab notebook, long-running AI model training programs can also be executed remotely to create trained models, represented in open neural network exchange (ONNX) format, and other output datasets in Galaxy. Other features include Git integration for version control, the option of creating and executing pipelines of notebooks, and multiple dashboards and packages for monitoring compute resources and visualisation, respectively. **Conclusions** These features make JupyterLab in Galaxy Europe highly suitable for creating and managing AI projects. A recent scientific publication that predicts infected regions in COVID-19 CT scan images is reproduced using various features of the JupyterLab on Galaxy Europe. In addition, ColabFold, a faster implementation of AlphaFold2, is accessed in JupyterLab to predict the 3D structure of protein sequences. JupyterLab is accessible in two ways - one as an interactive Galaxy tool and the other by running the underlying Docker container. In both ways, long-running training can be executed on Galaxy's compute infrastructure. Scripts to create the Docker container are available under MIT license at https://github.com/anuprulez/ml-jupyter-notebook.

Key words: JupyterLab; Galaxy Europe; Artificial intelligence; Remote model training; ONNX; Elyra AI; GPU; CUDA;

Findings

Background

Bioinformatics comprises many sub-fields such as single-cell, medical imaging, sequencing, proteomics and many more that produce a huge amount of biological data in myriad formats. For example, the single-cell field creates gene expression patterns for each cell that are represented as matrices of real numbers. The medical imaging field generates images of cells and tissues, radiography images such as chest x-rays and computerized tomography (CT) scans. Next-Generation sequencing generates deoxyribonucleic

Compiled on: January 23, 2023.
Draft manuscript prepared by the author.

acid (DNA) sequences that are stored as fasta [1] files. Machine learning (ML) approaches have been vastly used with these datasets [2] for predictive tasks such as medical diagnosis, imputing missing features, augmenting datasets with artificially generated ones, estimating gene expression patterns and many more. To be able to use ML algorithms on such datasets, a robust and efficient compute infrastructure is needed that can serve multiple purposes. They include pre-processing raw datasets to transform them into suitable formats that are compatible with ML algorithms, creating and executing their complex architectures on pre-processed datasets and making trained models and predicted datasets readily available for further analyses. To facilitate such tasks, a complete infrastructure is developed that combines JupyterLab [3], augmented with many useful features, running on the vast public compute infrastructure of Galaxy [4] Europe to perform end-to-end AI analyses on scientific datasets. The infrastructure consists of three major components. First, a Docker container [5] that encapsulates JupyterLab together with multiple packages and plugins used for developing AI programs, data manipulation and visualisation. Section S2 in the supplementary file lists all such packages and plugins with their respective versions. Second, a Galaxy interactive tool [6, 7] that downloads this Docker container to serve JupyterLab online on Galaxy Europe. Third, the compute infrastructure consisting of over 8000 CPUs and a few GPUs and has over 60 TB of memory and 5 PB of disk space. From this collection, each session of JupyterLab receives over 20 gigabytes (GB) of memory (RAM), in addition to 15 GB of memory provided by one GPU. The disk space is 1 TB and there are 7 CPUs.

Docker container

Docker [8] containers are popular for shipping packaged software as complete ecosystems, enabling them to be reproducible in a platform-independent manner. Software executing inside a Docker container is abstracted from the host operating system (OS) as most of the requirements necessary for them to run successfully are already configured inside its container. A container runs in an isolated environment having minimal interactions with the host OS. Therefore, running softwares in a container is more secure. Using a Docker container leverages the security benefits necessary for online program editing softwares executing arbitrary code. Arbitrary code may contain some malicious script posing security risks. Using Docker containers can minimise their consequences. Further, in our Docker container, a non-root user is created that can execute and manage projects inside the JupyterLab environment which further minimises security risks. In addition to minimising security risks, Docker containers provide performance benefits compared to running programs on a virtual machine [9]. Motivated by such benefits, a Docker container is used in this project to encapsulate JupyterLab along with many useful packages such as Git [10], Elyra AI [11], TensorFlow-GPU [12], Scikit-learn [13], ONNX [14], and many others. The Docker container inherits many packages such as CUDA [15], NumPy [16], SciPy [17] and a few more from its base container, nvidia/cuda [18], and augments them with many other packages suitable for ML, data manipulation and visualisation. The Docker container is decoupled from Galaxy and can independently be executed for serving JupyterLab with the same set of packages on a different compute infrastructure or any personal computer (PC) or laptop having approximately 25 GB of disk space. Moreover, the Docker container is easily extended by adding the names of packages to the dockerfile [19]. Adding new packages requires the container to be rebuilt and added to Docker hub [20]. The approach to extending the Docker container is discussed in the Methods section.

JupyterLab

JupyterLab is a web-based, robust editor used for varied purposes such as data science, scientific computing and ML. It is a program editor that supports more than 40 programming languages including Python, R, Julia and Scala. Python is one of the most popular languages used by researchers for performing numerous scientific and predictive analyses. Therefore, it is used as the programming language in Galaxy's JupyterLab because many popular packages such as Scikit-learn and TensorFlow for ML, data manipulation packages such as Pandas, visualisation packages such as Seaborn [21], Matplotlib [22], Bokeh [23] and many others are readily available as Python packages. Moreover, the extensible architecture of JupyterLab makes it possible to add many external packages as its plugins such as Git, Elyra AI, dashboards and many others that have a user interface (UI) as necessary components. Such editors, integrated with several useful packages, provide a favourable platform for rapid prototyping and end-to-end development and management of AI projects. To harness the benefits of JupyterLab, it has been used as the editor for the interactive tool in Galaxy.

Features of JupyterLab infrastructure

Many features such as easy accessibility, support of a wide variety of programming languages on JupyterLab, and extensibility to install useful plugins make it a desirable editor for researchers for creating project prototypes rapidly. Many such features have been integrated into our JupyterLab infrastructure that is served online on Galaxy Europe enabling researchers to create prototypes and end-to-end AI projects (Figure 1). A few important features are discussed here. To allow GPU computation from JupyterLab, TensorFlow-GPU interacts with Nvidia GPU hardware using another software, CUDA, when the compute resource has GPU(s) for accelerating ML programs. Faster execution of ML programs is one of the significant features of JupyterLab hosted on Galaxy Europe. However, if the hosted machine on which a Docker container runs does not have GPUs, then the program in JupyterLab relies on CPUs. Other useful features include ONNX for transforming trained TensorFlow and Scikit-learn models to ONNX models, Open-CV [24] and Scikit-image [25] for processing images, Nibabel [26] for reading image files stored as ".nii", Bioblend [27] for accessing Galaxy's datasets, histories and workflows in a JupyterLab notebook and visualisation packages such as Bqplot [28] and Bokeh for plotting interactive charts, Voilà [29] for displaying output cells of a JupyterLab notebook in a different tab, dashboard such as NVDashboard [30] for monitoring GPU usage and performance. Support for file extension such as H5 [31], efficient for storing matrices, enables ML researchers to save model weights and input datasets for AI algorithms. Other packages such as ColabFold [32] together with JAX [33] are used for predicting 3D structures of proteins which are discussed in the Results section. In addition, it is possible to create a long-running training job that runs remotely and stores trained models and output datasets permanently in a newly created Galaxy history. The trained model is saved as an ONNX file and tabular datasets are in an H5 file. It is discussed in the Methods section.

Related infrastructure

There are a few other infrastructures available, free and commercial, that offer JupyterLab or similar environments for developing data science and AI projects. A few popular ones are Google Colab [34], Kaggle Kernel [35] and Amazon Sagemaker [36]. Google Colab is partially free and offers an online editor similar to JupyterLab. The free version of Google Colab offers dynamic compute resources. The disk space is around 70 GB and the memory (RAM) is around 12 GB. These resources are scarce for AI projects that deal with high-dimensional scientific data [37, 38]. In addition, these resources

offered by Google Colab are variable and depend on a user's past usage. More compute resources are assigned to those users that have used less in the past for a more equitable sharing of resources. Moreover, there is a limitation of only 12 hours of running time that may prove to be insufficient for training AI models on large datasets. However, Google Colab pro and pro+ offer better compute resources but they come at a price; EUR 9.25 and EUR 42.25 per month, respectively. In contrast, Kaggle Kernel is free of charge, but similar to Colab, their computing resources are scarce. The total disk space is approximately 73 GB and RAM is 16 GB for a CPU-based kernel. For the GPU-based kernel, the disk space is of the same size as that of the CPU-based kernel but the RAM of the CPU decreases to 13 GB. An additional RAM of 15 GB is added through a GPU and computation time is limited to 30 hours a week. It also supports TPUs but the computation time is further limited to only 20 hours a week. Amazon Sagemaker is also a commercial software for developing AI algorithms that is free of charge but only for 2 months. Overall, these notebook infrastructures do not offer unrestricted compute resources free of charge. In addition, compute resources offered free of charge are insufficient for training AI models on high-dimensional scientific datasets. To address the drawbacks of these notebook infrastructures and provide researchers and users with large compute resources more reliably, Galaxy JupyterLab infrastructure offers 1 terabyte (TB) of disk space and unlimited computation time on 1 GPU and 7 CPUs per session. RAM for GPU is around 15 GB and for CPUs is 20 GB (Table 1). The offered resources for JupyterLab running in Galaxy stay constant and are independent of the user's past usage. To make it more useful, JupyterLab opens a tab for each notebook that allows researchers to develop and execute several notebooks inside the same session of the allotted compute resource rather than having them connect to a different session for each notebook as in Google Colab and Kaggle Kernel.

Implementation

JupyterLab infrastructure has been developed in two stages. First, a Docker container is created containing all the necessary packages such as JupyterLab itself, CUDA from the base Docker image [39], TensorFlow, Scikit-learn, ONNX and many more. The Docker container is inherited from a base container that has all the necessary CUDA packages installed for working with NVIDIA GPUs. Many packages are added to the Docker container with their compatible versions. Compatible packages for CUDA, CUDA DNN and TensorFlow are necessary so that they together interact with the GPU on the host machine for accelerating ML programs. The versions of all packages installed in the Docker container are listed in Supplementary section S5. Second, the container can be downloaded to any powerful compute infrastructure and JupyterLab can be served in an internet browser via the URL that it generates. In addition, to run this container in Galaxy, an interactive tool is created that downloads this container on a remote compute infrastructure and generates a URL used to run JupyterLab in an internet browser. The architecture of JupyterLab infrastructure in Galaxy is shown in Figure 1. The running instance of JupyterLab in Galaxy contains a default IPython notebook that summarises several of its features. Further, there are other notebooks available, each describing a feature of JupyterLab with code examples such as how to create ONNX models for Scikit-learn and TensorFlow classifiers, how to connect to Galaxy using Bioblend, how to create interactive plots using Bqplots and how to create a pipeline of notebooks using Elyra AI. In addition, the notebooks explaining the use-cases are also available in the Docker container. To access JupyterLab in Galaxy Europe, a ready-to-use hands-on Galaxy training network (GTN) [40] tutorial [41] is developed that shows all the steps such as opening the notebook, using Git to clone a code repository from GitHub, sending long-running training jobs to a remote Galaxy cluster, and how this notebook can be used as a tool in a Galaxy workflow. The approach of remote model training is explained in the Methods section. The two use-cases are also discussed in the tutorial along with their respective notebooks. The steps to access this resource on Galaxy Europe are elaborated in Supplementary section S1.

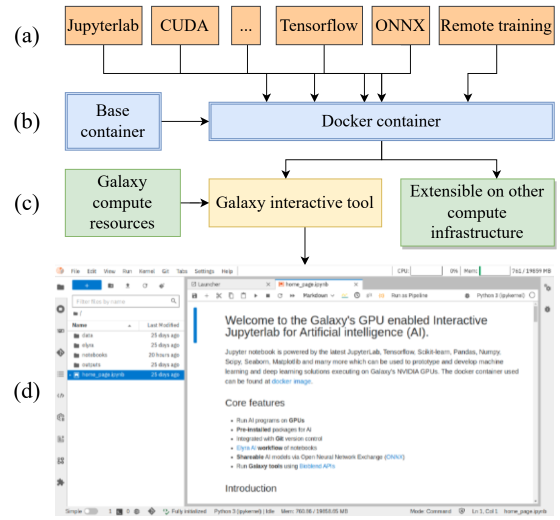

Figure 1. Architecture of Galaxy's JupyterLab. Part (a) shows packages and features wrapped inside a Docker container. Part (b) shows a base Docker container [39] from which the customised container [5] is derived. In part (c), Galaxy's interactive tool downloads the customised container. The customised Docker container can also be hosted on a different compute infrastructure. Part (d) shows Galaxy's JupyterLab.

Results

JupyterLab infrastructure in Galaxy Europe is used to reproduce the results of two recent scientific publications. They demonstrate its robustness and usefulness to develop ML models using COVID CT scan images [42] and predict the 3D structure of proteins using ColabFold, a faster implementation of AlphaFold2 [43].

COVID-19 CT scan image segmentation

In [42], COVID-19 CT scan images have been used to develop and train an ML model architecture that predicts COVID-19 infected regions in those images with high accuracy. An open-source implementation of the work is available that trains a Unet DL architecture [44] that distinguishes between normal and infected regions in CT scan images. Scripts of this implementation are adapted and executed on Galaxy's JupyterLab infrastructure. Adaption only involves the transformation of all CT scan images, used in [42], into an H5 file so that they can directly be used as an input to the Unet architecture defined in a notebook in [45]. All the notebooks available in [45] are also available in the Docker container in the "usecases" directory. A composite H5 file [46] is created using a script [47] that contains multiple datasets inside and each dataset is a real-valued matrix corresponding to the training, test and validation sets as used in [42]. The entire analysis of [42] can be reproduced using multiple notebooks in [45]. They achieve similar precision and recall (approximately 0.98) metrics as mentioned in [42]. In [45], the first notebook (1_fetch_datasets.ipynb) downloads the input dataset as an H5 file. Additionally, it also downloads the trained ONNX model. The second notebook (2_create_model_and_train.ipynb) creates and trains a Unet model on the training dataset extracted from the H5 file. Training, accelerated by GPU, for 10 iterations over the entire training dataset finishes in a few minutes. The third

Table 1. Comparison of Galaxy JupyterLab with other notebook infrastructures

Indicators/Infrastructures	Google Colab [34]	Kaggle Kernel [35]	Galaxy JupyterLab
Memory/Disk space (GB)	12/70	16/73	**20/1000**
GPU/TPU	Yes/**Yes**	Yes/**Yes**	**Yes**/No
Max usage time (Hours)	12	12, 30 hrs of GPU/week, 20 hrs of TPU/week	**No time restriction** on GPU usage, notebook sessions and job execution
Dynamic compute resources	Yes	Yes	**Fixed and guaranteed**
Remote model training	No	No	**Yes**
Run multiple notebooks (as tabs) in one session	No	No	**Yes**

notebook (3_predict_masks.ipynb) extracts the test dataset and predicts infected regions of the CT scan images in the test dataset using the trained model created by the second notebook. Figure 2 shows the comparison of ground truth infected regions in the second column and the predicted infected regions in the third column. A few original CT scan images from the test dataset are shown in the first column of Figure 2.

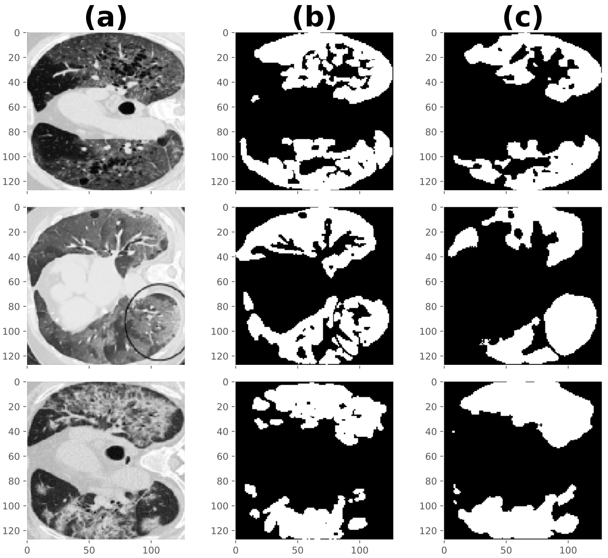

Figure 2. Figure shows original CT scan images in column (a), corresponding ground-truth masks of original CT scan images in column (b) and the predicted masks in column (c). Masks are COVID-19 infected regions in the corresponding CT scan images. The ground-truth and predicted masks show high similarity [42].

Predict 3D structure of proteins using ColabFold

AlphaFold2 has made a breakthrough in predicting the 3D structures of proteins with outstanding accuracy. However, due to their large database size (a few TB), it is not easily accessible to researchers. Therefore, a few approaches have been developed that replace the time-consuming steps of AlphaFold2 with slightly different steps but predict the 3D structure of proteins with similar accuracy while consuming less memory and time. One such approach is ColabFold which replaces a large database search in AlphaFold2 for finding homologous sequences by a significantly (40-60 times) faster MMseqs2 API [48] call to generate input features based on the query protein sequence. ColabFold's prediction of 3D structures in batches is approximately 90 times faster. It is integrated into the Docker container [5] by adding two packages - ColabFold and GPU-enabled JAX which is a just-in-time compiler for making mathematical transformations. "7_ColabFold_MMseq2.ipynb" notebook in [45] predicts the 3D structure of a protein sequence using ColabFold by making use of the AlphaFold2 pre-trained weights. Figure 3 shows the 3D structure of 4Oxalocrotonate_Tautomerase [49], a protein sequence of length 62, along with its side chains. This 3D structure is extremely similar to the structure predicted by the Jupyter notebook [50] from ColabFold [32].

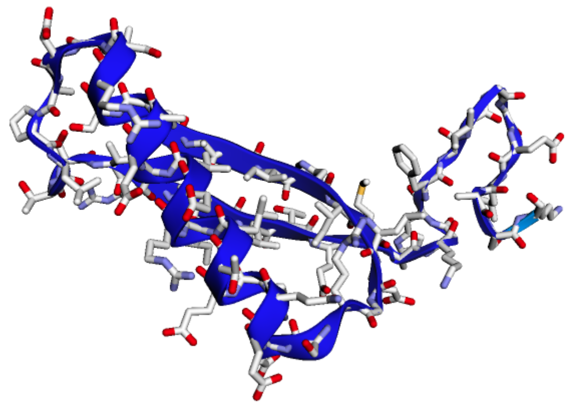

Figure 3. Figures shows a 3D structure of 4 Oxalocrotonate_Tautomerase enzyme (protein) [49] predicted by ColabFold

Methods

Remote model training

For large datasets, ML model training may need several hours or even days. In such cases, it would be cumbersome to keep JupyterLab open in a browser's tab till the training finishes. Therefore, another Galaxy tool [51] is developed to enable researchers to send long-running training jobs to a remote Galaxy cluster. The tool can be executed from JupyterLab using a custom Python function [52], part of each JupyterLab notebook, that takes input datasets and a training script as input parameters. The input datasets to be used for training, testing and validation must be provided in H5 format. It allows the standardisation of input data format for AI models that train on matrices in JupyterLab. Input data to an AI model can be in multiple formats such as images, genomic sequences or gene expression patterns. H5 files can be created using any of these data formats and fed to the AI model in JupyterLab. Long-running training happens in a remote Galaxy cluster as a regular Galaxy job. Upon completion of the job, the resulting datasets and the

trained model become available in a newly created Galaxy history [53]. The trained model and other resulting datasets can either be downloaded to a local machine or imported from the Galaxy history for further analysis using "get" (for fetching datasets directly into a JupyterLab notebook from Galaxy history) and "put" (for saving datasets directly into a Galaxy history from JupyterLab notebook) methods into a JupyterLab notebook [54]. In [45], a few notebooks are available that showcase the approach of remote model training. Notebook "4_create_model_and_train_remote.ipynb" contains code for developing and training a Unet architecture. Notebook "5_run_remote_training.ipynb" executes the previous notebook on a Galaxy cluster remotely after creating a Galaxy history and then uploading the script extracted from "4_create_model_and_train_remote.ipynb" notebook and input datasets. Custom Python function, "run_script_job", creates a Galaxy history using Bioblend and then uploads the datasets to the same history. After the upload is finished, the Python script from the specified notebook is executed dynamically. It trains an ML model on the uploaded datasets to create a model and saves it as an ONNX file in the Galaxy history. Using "6_predict_masks_remote_model.ipynb" notebook from [45], the trained model can be downloaded from the Galaxy history and used for predicting infected regions of the CT scan images of the test dataset. A significant advantage of training ML models remotely is that researchers don't have to keep the JupyterLab session running as long as the model is being trained as model training becomes decoupled from JupyterLab. Using such a feature, ML models that take several hours or even days to train can be conveniently trained.

Extend Docker container

The customised Docker container developed as shown in Figure 1 can be easily extended to have more or different packages. To update the container, a package or a list of new packages should be added to the dockerfile and then the new container should be built and pushed to Docker hub [5]. After pushing the new container, when Galaxy's JupyterLab interactive tool is accessed on Galaxy Europe, it downloads the new container and all the newly added packages are available in JupyterLab. Similarly, versions of existing packages can be updated or existing packages can be removed if no longer needed. The simplified extension procedure of the entire infrastructure incurs low maintenance costs as any change to this entire infrastructure is reflected only in the container without updating Galaxy's codebase. In addition, packages can also be added or updated using "pip" in any JupyterLab notebook. But, such changes remain as long as the JupyterLab session runs as they don't update the underlying Docker container.

Collaborative notebooks

Notebooks created in Galaxy's JupyterLab infrastructure can instantly be shared with other researchers and collaborators only by sharing the public URL of a notebook. Researchers and users that share a notebook can collaborate on the same notebook without having to store it anywhere as it is directly served by Galaxy Europe.

Workflow of notebooks

Resembling many tools in Galaxy, JupyterLab can also be used in any Galaxy workflow where it can accept datasets from different tools and then executes an IPython notebook to process the input datasets. It outputs a collection of datasets which can further be used by other Galaxy tools [41]. In addition, using the Elyra AI package, a workflow of notebooks can be created using existing notebooks in a JupyterLab session and executed as one unit of software similarly as Galaxy workflows are created using several tools. It is possible to execute such workflows of notebooks on the same compute resource on which the JupyterLab session runs. In addition, a few other services such as Kubeflow [55] or Apache Airflow [56] can also be used to deploy, run and manage such workflows on a cloud but are not explored in our work.

Summary

JupyterLab is integrated as an interactive tool in Galaxy Europe running on a public and powerful compute infrastructure comprising several CPUs and GPUs having large memories and disk space. A Docker container is created that wraps JupyterLab along with packages such as TensorFlow-GPU, Scikit-learn, Pandas and many others to provide a robust architecture for the development and management of projects in ML and data science. Remote model training makes it convenient to run multiple analyses in parallel in different Galaxy jobs by executing the same Galaxy tool. The resulting datasets of each job become available in different Galaxy histories. Features such as Git integration are useful for managing entire code repositories on GitHub and Elyra AI for creating pipelines of notebooks working as one software unit. All notebooks created by a user run on the same session of JupyterLab in different tabs. The entire infrastructure of JupyterLab is readily accessible through Galaxy Europe. In contrast to commercial infrastructures that host editors similar to JupyterLab and offer powerful and reliable compute only through paid subscriptions, this infrastructure provides large compute resources free of cost which are invariant to usage and has an unlimited usage time while ensuring a constant amount of compute resources across successive usages.

Availability of supporting source code and requirements

Project name: GPU-enabled Docker container with JupyterLab for artificial intelligence
Project home page: `https://github.com/anuprulez/ml-jupyter-notebook`
Galaxy interactive tool: `https://github.com/usegalaxy-eu/galaxy/blob/release_22.05_europe/tools/interactive/interactivetool_ml_jupyter_notebook.xml`
Operating system: Linux
Programming languages: Python, XML, Docker, Bash
License: MIT License
RRID: SCR_022695
Biotools ID: gpu-enabled_docker_container_with_jupyterlab_for_ai

Additional files

Supplementary Material: An accessible infrastructure for artificial intelligence using a docker-based JupyterLab in Galaxy.

List of abbreviations

AI: Artificial intelligence; CT: Computerised tomography; CUDA: Compute unified device architecture; DL: Deep learning; DNA: Deoxyribonucleic acid; EUR: Euro; GPU: Graphical processing unit; GB: Gigabyte; GTN: Galaxy training network; JAX: Accelerated linear algebra; ML: Machine learning; ONNX: Open neural network exchange; OS: PC: Personal computer; RAM: Random-access memory; TB: Terabyte; PB: Petabyte; UI: User interface; URL: Uniform resource locator;

Competing Interests

The authors declare that they have no competing interests.

Ethics approval and consent to participate

Not applicable

Consent for publication

Not applicable

Funding

This work was supported by the German Research Foundation (DFG) under Germany's Excellence Strategy (CIBSS - EXC-2189 - Project ID 390939984) and German Federal Ministry of Education and Research (BMBF grant 031A538A de.NBI).

Authors' contributions

A.K. developed the project and wrote the manuscript. G.C. deployed the project on Galaxy Europe. B.G. devised the idea of the project and helped in creating the resource's access method. R.B. provided the necessary support for the entire project. All authors contributed to and approved the manuscript.

Acknowledgements

We thank Daniel Blankenberg for his suggestions to improve the Docker container. In addition, we thank Galaxy Europe team for running and maintaining the project.

References

1. Pearson W, et al, The FASTA package - protein and DNA sequence similarity searching and alignment programs. GitHub; 2016. https://github.com/wrpearson/fasta36. 2016. Accessed 30 June 2022.
2. Kumar I, Singh SP, Shivam. Chapter 26 - Machine learning in bioinformatics. Academic Press 2022;p. 443–456. https://www.sciencedirect.com/science/article/pii/B9780323897754000201.
3. Kluyver T, Ragan-Kelley B, Pérez F, Granger B, Bussonnier M, Frederic J, et al.; IOS Press. Jupyter Notebooks—a publishing format for reproducible computational workflows 2016;p. 87.
4. Afgan E, Baker D, Batut B, et al. The Galaxy platform for accessible, reproducible and collaborative biomedical analyses: 2018 update. Nucleic Acids Research 2018;46(W1):W537–W544. doi:10.1093/nar/gky379.
5. Kumar A, Container for machine learning and deep learning in Jupyter notebook. Docker; 2021. https://hub.docker.com/r/anupkumar/docker-ml-jupyterlab. 2021. Accessed 29 June 2022.
6. Galaxy Europe, Live instance of the European Galaxy server. Galaxy Europe; 2019. https://live.usegalaxy.eu/. 2019. Accessed 30 June 2022.
7. Kumar A, et al, GPU enabled Interactive Jupyter Notebook for Machine Learning; 2021. https://github.com/usegalaxy-eu/galaxy/blob/release_22.01_europe/tools/interactive/interactivetool_ml_jupyter_notebook.xml.
8. Merkel D. Docker: lightweight linux containers for consistent development and deployment. Linux journal 2014;2014(239):2.
9. Baset et al , Docker and Container Security White Paper; 2016. https://dominoweb.draco.res.ibm.com/reports/rc25625.pdf.
10. Collonval Fea, A JupyterLab extension for version control using Git.;. https://github.com/jupyterlab/jupyterlab-git. 2017. Accessed 29 June 2022.
11. Resende Lea, Elyra is a set of AI-centric extensions to JupyterLab Notebooks.;. https://github.com/elyra-ai/elyra. 2018. Accessed 29 June 2022.
12. Abadi M, Agarwal A, Barham P, Brevdo E, Chen Z, Citro C, et al., TensorFlow: Large-Scale Machine Learning on Heterogeneous Systems; 2015. https://www.tensorflow.org/, software available from tensorflow.org.
13. Pedregosa F, Varoquaux G, Gramfort A, Michel V, Thirion B, Grisel O, et al. Scikit-learn: Machine Learning in Python. Journal of Machine Learning Research 2011;12:2825–2830.
14. Bai J, Lu F, Zhang K, et al, ONNX: Open Neural Network Exchange. GitHub; 2019. https://github.com/onnx/onnx. 2019. Accessed 29 June 2022.
15. NVIDIA, Vingelmann P, Fitzek FHP, CUDA, release: 10.2.89; 2020. https://developer.nvidia.com/cuda-toolkit.2020.Accessed 29 June 2022.
16. Harris CR, Millman KJ, van der Walt SJ, Gommers R, Virtanen P, Cournapeau D, et al. Array programming with NumPy. Nature 2020 Sep;585(7825):357–362. https://doi.org/10.1038/s41586-020-2649-2.
17. Virtanen P, Gommers R, Oliphant TE, Haberland M, Reddy T, Cournapeau D, et al. SciPy 1.0: Fundamental Algorithms for Scientific Computing in Python. Nature Methods 2020;17:261–272.
18. NVIDIA Corporation, CUDA and cuDNN images from gitlab.com/nvidia/cuda. Docker; 2014. https://hub.docker.com/r/nvidia/cuda. 2014. Accessed 29 June 2022.
19. Kumar A, Jupyter container used for Data Science and Tensorflow. GitHub; 2021. https://github.com/anuprulez/ml-jupyter-notebook/blob/master/Dockerfile. 2021. Accessed 29 June 2022.
20. Docker Hub. Docker; 2013. https://hub.docker.com/.
21. Waskom ML. seaborn: statistical data visualization. Journal of Open Source Software 2021;6(60):3021. https://doi.org/10.21105/joss.03021.
22. Hunter JD, Matplotlib: A 2D graphics environment. IEEE COMPUTER SOC; 2007.
23. Bokeh Development Team, Bokeh: Python library for interactive visualization. GitHub; 2018. https://bokeh.pydata.org/en/latest/.
24. Bradski G. The OpenCV Library. Dr Dobb's Journal of Software Tools 2000;.
25. Van der Walt S, Schönberger JL, Nunez-Iglesias J, Boulogne F, Warner JD, Yager N, et al. scikit-image: image processing in Python. PeerJ 2014;2:e453.
26. Brett M, Markiewicz CJ, Hanke M, Côté MA, Cipollini B, McCarthy P, et al., nipy/nibabel: 3.2.2. Zenodo; 2022. https://doi.org/10.5281/zenodo.6617121.
27. Sloggett C, Goonasekera N, Afgan E. BioBlend: automating pipeline analyses within Galaxy and CloudMan. Bioinformatics 2013;29(13):1685–1686. https://doi.org/10.1093/bioinformatics/btt199.
28. Corlay, S and et al , 2-D plotting library for Project Jupyter. GitHub; 2015. https://github.com/bqplot/bqplot. 2015. Accessed 29 June 2022.
29. Tuloup, J and et al , Rendering of live Jupyter notebooks with interactive widgets. GitHub; 2018. https://github.com/voila-dashboards/voila. 2018. Accessed 29 June 2022.
30. Tomlinson, J and et al , A JupyterLab extension for displaying GPU usage dashboards. GitHub; 2021. https://github.com/rapidsai/jupyterlab-nvdashboard. 2021. Accessed 29 June 2022.

31. The HDF Group, Hierarchical Data Format, version 5; 1997-2022. https://www.hdfgroup.org/HDF5/. 1997. Accessed 29 June 2022.
32. Mirdita M, Schütze K, Moriwaki Y, et al. ColabFold: making protein folding accessible to all. Nat Methods 2022;19:679–682 (2022). https://doi.org/10.1038/s41592-022-01488-1.
33. Johnson M, et al, JAX: Autograd and XLA; 2020. https://github.com/google/jax. 2020. Accessed 29 June 2022.
34. Bisong E. Google Colaboratory 2019;p. 59–64. https://doi.org/10.1007/978-1-4842-4470-8_7.
35. Kaggle, Kaggle; 2020. https://www.kaggle.com. 2010. Accessed 29 June 2022.
36. Amazon SageMaker, Amazon SageMaker; 2017. https://aws.amazon.com/sagemaker/. 2017. Accessed 29 June 2022.
37. Moon KR, van Dijk D, Wang Zea. Visualizing structure and transitions in high-dimensional biological data. Nat Biotechnol 2019;37:1482–1492 (2019). https://doi.org/10.1038/s41587-019-0336-3.
38. Boileau P, Hejazi NS, Dudoit S. Exploring high-dimensional biological data with sparse contrastive principal component analysis. Bioinformatics 2020;36(11):3422–3430. https://doi.org/10.1093/bioinformatics/btaa176.
39. nvidia/cuda:11.8.0-cudnn8-runtime-ubuntu20.04. Nvidia/Docker; 2014. https://hub.docker.com/layers/nvidia/cuda/11.8.0-cudnn8-runtime-ubuntu20.04/images/sha256-74b166e2091bb705e9ada685dffe79930612c725669bc87e01125b5245d13f97?context=explore.
40. Batut B, Hiltemann S, Bagnacani A, Baker D, Bhardwaj V, Blank C, et al. Community-Driven Data Analysis Training for Biology. Cell Systems 2018 jun;6(6):752–758.e1. https://doi.org/10.1016/j.cels.2018.05.012.
41. Kumar A, A Docker-based interactive Jupyterlab powered by GPU for artificial intelligence in Galaxy (Galaxy Training Materials); 2022. https://training.galaxyproject.org/training-material/topics/statistics/tutorials/gpu_jupyter_lab/tutorial.html. 2022. Accessed 23 January 2023.
42. Saeedizadeh N, Minaee S, Kafieh R, Yazdani S, Sonka M. COVID TV-Unet: Segmenting COVID-19 chest CT images using connectivity imposed Unet. Computer Methods and Programs in Biomedicine Update 2021;1:100007. https://www.sciencedirect.com/science/article/pii/S2666990021000069.
43. Jumper J, Evans R, Pritzel A, et al. Highly accurate protein structure prediction with AlphaFold. Nature 2021;596:583–589 (2021). https://doi.org/10.1038/s41586-021-03819-2.
44. Ronneberger O, Fischer P, Brox T. U-Net: Convolutional Networks for Biomedical Image Segmentation. CoRR 2015;abs/1505.04597. http://arxiv.org/abs/1505.04597.
45. Kumar A, Jupyterlab notebooks. GitHub; 2022. https://github.com/anuprulez/gpu_jupyterlab_ct_image_segmentation. 2022. Accessed 29 June 2022.
46. Kumar A, COVID Image segmentation datasets and trained model. Zenodo; 2022. https://doi.org/10.5281/zenodo.6091361.
47. Saeedizadeh N, Minaee S, Kafieh R, Yazdani S, Sonka M, COVID TV-Unet: Segmenting COVID-19 chest CT images using connectivity imposed Unet. GitHub; 2021. https://github.com/narges-sa/COVID-CT-Segmentation/blob/main/main_TV_Unet_Split1.py. 2021. Accessed 30 June 2022.
48. Steinegger M, Söding J. MMseqs2 enables sensitive protein sequence searching for the analysis of massive data sets. Nat Biotechnol 2017;35:1026–1028 (2017). https://doi.org/10.1038/nbt.3988.
49. Chen L, Kenyon G, Curtin F, Harayama S, Bembenek M, Hajipour G, et al. 4-Oxalocrotonate tautomerase, an enzyme composed of 62 amino acid residues per monomer. J Biol Chem 1992;267(25):17716–21. https://pubmed.ncbi.nlm.nih.gov/1339435/.
50. Mirdita M, Schütze K, Moriwaki Y, et al, ColabFold: making protein folding accessible to all. GitHub; 2022. https://github.com/sokrypton/ColabFold/blob/main/AlphaFold2.ipynb. 2021. Accessed 30 June 2022.
51. Kumar A, Run long running jupyterlab script. Github; 2022. https://github.com/bgruening/galaxytools/blob/master/tools/jupyter_job/run_jupyter_job.xml. 2022. Accessed 30 June 2022.
52. Kumar A, Custom jupyterlab notebook function to start model training job in Galaxy. Github; 2021. https://github.com/anuprulez/ml-jupyter-notebook/blob/master/galaxy_script_job.py#L43. 2021. Accessed 30 June 2022.
53. Kumar A, Remotely trained image segmentation model. Galaxy; 2022. https://usegalaxy.eu/u/kumara/h/image-segmentation-from-galaxy-jupyterlab. 2022. Accessed 23 August 2022.
54. Galaxy's Interactive Environments. GitHub; 2016. https://github.com/bgruening/docker-jupyter-notebook/blob/master/default_notebook.ipynb.
55. Kubeflow. Github; 2017. https://github.com/kubeflow/kubeflow.
56. Apache Airflow. Github; 2019. https://github.com/apache/airflow-site.

Supplementary document PDF

Click here to access/download

Supplementary Material

Supplementary Material_GPU_Jupyterlab.pdf

Cover letter

Click here to access/download;Personal Cover;Cover letter for Galaxy jupyterlab project.pdf

Dear Dr. Goodman,

We have developed a Jupyterlab notebook infrastructure in Galaxy Europe that is docker-based and GPU-enabled for developing machine learning, deep learning and data science projects. We would like to submit the associated paper titled "An accessible infrastructure for artificial intelligence using a docker-based Jupyterlab in Galaxy" to GigaScience as a technical note. The infrastructure allows artificial intelligence (AI) researchers to rapidly develop their project prototypes and end-to-end projects artificial intelligence projects. There are many interesting aspects of the work which bring it closer to GigaScience:

- **High-quality software**: The infrastructure supports high-quality softwares such as Tensorflow and Scikit-learn for writing AI models; Bokeh and Seaborn for interactive visualizations; Elyra AI for creating workflow of notebooks and Git for managing entire codebases; Bioblend to access Galaxy's tools, datasets and workflows and GPU computation for faster training of AI models.
- **Publicly available**: The infrastructure is available on Galaxy Europe for registered and authorised Galaxy users. The extra authorisation step is necessary to restrict the non-intended usage of public compute resources (especially GPUs) such as bitcoin mining as experienced by us in the past.
- **Shareability and reproducibility**: Jupyterlab notebooks created using this infrastructure promote sharing of analyses among researchers by making them shareable directly via a URL.
- **Extensibility**: Softwares included in the infrastructure are controlled by a docker file and are independent of Galaxy. To extend it to be used in other scientific fields, then only the new set of packages should be installed and the new container should be updated to the docker hub making the infrastructure extensible to different scientific fields beyond Bioinformatics.
- **Unlimited compute time and remote model training**: Researchers can train AI models for hours or even days without caring about when their programs would terminate. Other similar infrastructures such as Google Colab and Kaggle Kernels restrict users to have limited running time (~12 hours). Also, Galaxy's jupyterlab infrastructure provides an approach to train AI models remotely.
- **Example notebooks and tutorials**: Several example notebooks (1, 2) are available for users to learn different aspects of the entire infrastructure. Also, a tutorial is available on how to use this resource.

Potential reviewers of the manuscript:

- Joshua T. Vogelstein: jovo@jhu.edu
- Kelly D Goodwin: Kelly.Goodwin@noaa.gov
- Stephen R Piccolo: stephen_piccolo@byu.edu
- David Landsman: landsman@ncbi.nlm.nih.gov
- Alexej Abyzov: abyzov.alexej@mayo.edu

The authors agree that there are no competing interests and they have approved the manuscript to be submitted to GigaScience. Moreover, the manuscript has not been sent elsewhere. The first version of the manuscript was uploaded to bioRxiv (preprint server).

On behalf of all authors,

Yours sincerely,

Anup Kumar
