# Supplementary material for: An accessible infrastructure for artificial intelligence using a Docker-based JupyterLab in Galaxy
Source: Gigascience. 2023 Apr 26;12:giad028. doi: 10.1093/gigascience/giad028 (PMC10132306; doi:10.1093/gigascience/giad028)
Supplement: giad028_GIGA-D-22-00220_Revision_2 [file giad028_giga-d-22-00220_revision_2.pdf]

GigaScience

An accessible infrastructure for artificial intelligence using a Docker-based JupyterLab in Galaxy

--Manuscript Draft--

**Manuscript Number:**	GIGA-D-22-00220R2
**Full Title:**	An accessible infrastructure for artificial intelligence using a Docker-based JupyterLab in Galaxy
**Article Type:**	Technical Note
**Funding Information:**	DFG (German Research Foundation) (CIBSS - EXC-2189 - Project ID 390939984) — Dr Rolf Backofen<br>BMBF grant (de.NBI) (031A538A) — Dr Björn Grüning<br>European Commission (HORIZON-INFRA-2021-EOSC-01 - EOSC- EuroScienceGateway - 101057388) — Dr Björn Grüning
**Abstract:**	Background Artificial intelligence (AI) programs that train on large datasets require powerful compute infrastructure consisting of several CPU cores and GPUs. JupyterLab provides an excellent framework for developing AI programs but it needs to be hosted on such an infrastructure to enable faster training of AI programs using parallel computing.<br><br>Findings An open-source, docker-based, and GPU-enabled JupyterLab infrastructure is developed that runs on the public compute infrastructure of Galaxy Europe consisting of thousands of CPU cores, many GPUs and several petabytes (PB) of storage to rapidly prototype and develop end-to-end AI projects. Using a JupyterLab notebook, long-running AI model training programs can also be executed remotely to create trained models, represented in open neural network exchange (ONNX) format, and other output datasets in Galaxy. Other features include Git integration for version control, the option of creating and executing pipelines of notebooks, and multiple dashboards and packages for monitoring compute resources and visualisation, respectively.<br><br>Conclusions These features make JupyterLab in Galaxy Europe highly suitable for creating and managing AI projects. A recent scientific publication that predicts infected regions in COVID-19 CT scan images is reproduced using various features of JupyterLab on Galaxy Europe. In addition, ColabFold, a faster implementation of AlphaFold2, is accessed in JupyterLab to predict the 3D structure of protein sequences. JupyterLab is accessible in two ways - one as an interactive Galaxy tool and the other by running the underlying Docker container. In both ways, long-running training can be executed on Galaxy's compute infrastructure. Scripts to create the Docker container are available under MIT license at https://github.com/usegalaxy-eu/gpu-jupyterlab-docker.
**Corresponding Author:**	Anup Kumar<br>Albert-Ludwigs-Universitat Freiburg<br>Freiburg, GERMANY
**Corresponding Author Secondary Information:**	
**Corresponding Author's Institution:**	Albert-Ludwigs-Universitat Freiburg
**Corresponding Author's Secondary Institution:**	
**First Author:**	Anup Kumar
**First Author Secondary Information:**	
**Order of Authors:**	Anup Kumar

Powered by Editorial Manager® and ProduXion Manager® from Aries Systems Corporation

	Gianmauro Cuccuru
	Björn Grüning
	Rolf Backofen
**Order of Authors Secondary Information:**	

Response to Reviewers:

GIGA-D-22-00220R1
An accessible infrastructure for artificial intelligence using a Docker-based JupyterLab in Galaxy
Anup Kumar; Gianmauro Cuccuru; Björn Grüning; Rolf Backofen
GigaScience

Dear Mr. Kumar,

Your manuscript "An accessible infrastructure for artificial intelligence using a Docker-based JupyterLab in Galaxy" (GIGA-D-22-00220R1) has been assessed by our reviewers. Based on these reports, and my own assessment as Editor, I am pleased to inform you that it is potentially acceptable for publication in GigaScience, once you have carried out some minor revisions suggested by our reviewers.

Author response:
We would like to thank the reviewers and the editors.
--

Their reports, together with any other comments, are below. Please also take a moment to check our website at https://www.editorialmanager.com/giga/ for any additional comments that were saved as attachments.

In addition, please register any new software application in the bio.tools and SciCrunch.org databases to receive RRID (Research Resource Identification Initiative ID) and biotoolsID identifiers, and include these in your manuscript. Computational workflows should be registered in workflowhub.eu and the DOIs cited in the relevant places in the manuscript. These will facilitate tracking, reproducibility and re-use of your tool.

Author response:
Thank you. The manuscript mentions the following RRID and biotoolsID identifiers:
RRID: SCR_022695
Biotools ID: gpu-enabled_docker_container_with_jupyterlab_for_ai
--

Once you have made the necessary corrections, please submit a revised manuscript online at:

https://www.editorialmanager.com/giga/

If you have forgotten your username or password please use the "Send Login Details" link to get your login information. For security reasons, your password will be reset.

Please include a point-by-point within the 'Response to Reviewers' box in the submission system. Please ensure you describe additional experiments that were carried out and include a detailed rebuttal of any criticisms or requested revisions that you disagreed with. Please also ensure that your revised manuscript conforms to the journal style, which can be found in the Instructions for Authors on the journal homepage. If the data and code has been modified in the revision process please be sure to update the public versions of this too.

The due date for submitting the revised version of your article is 18 May 2023.

We look forward to receiving your revised manuscript soon.

Best wishes,

Hongling Zhou

Powered by Editorial Manager® and ProduXion Manager® from Aries Systems Corporation

GigaScience
www.gigasciencejournal.com

Reviewer reports:
Reviewer #1: Thank you for addressing my comments and suggestions. I look forward to seeing this article in print.

Author response:
Thank you.
--

Reviewer #2: Kumar et al. present the revised version of their manuscript "An accessible infrastructure for artificial intelligence using a docker-based Jupyterlab in Galaxy". The revised version is much improved compared to the initial version. I could now follow the authors' instructions to enable GPU support within Galaxy Europe and was able to execute the GPU-based ColabFold notebook and some of the other notebooks without any issues. The authors have resolved my previous points except as noted below. The remaining points should be resolvable during editing and should not require another review round.

Author response:
Thank you for your review comments.
--

Minor:
- In the authors' response it is mentioned that Galaxy Europe currently provides 16+30 GPUs. In the manuscript this is only referred to as "a few GPUs". I would recommend to state the currently available set of GPU resources in the manuscript.

Author response:
Thank you for your comment. The set of available GPUs within Galaxy Europe and through the Pulsar network are shared across many tools and processes. Only in the best scenario, all of them are available exclusively to the GPU-enabled JupyterLab in Galaxy. In future, the number of GPUs may increase too. In general, the resources might vary over time, not only for GPUs. Due to these reasons, we think it would be confusing for future readers if fixed numbers were stated in the manuscript as they are bound to change over time. To show this number, we have added a table at the Galaxy community site under (https://galaxyproject.org/news/2023-01-24-gpu-jupyterlab-galaxy/#current-resources-will-be-updated-regularly) that reports the availability of the current number of GPUs and other resources. This table is cited in the manuscript at appropriate places and the statistics of the table will be updated from time to time. The link is also referenced in the manuscript.
--

I also continue to recommend to not refer to GPU resources of commercial providers as "scarce". It's clear that its difficult or nearly impossible to compete with the big commercial providers, however, this doesn't detract from the usefulness of the provided service. My hope would be that clearly stating the current situation will also make the need clear to funders to sufficiently and sustainably fund computational resources in the future.

Author response:
Thank you for your comment. We have removed the word "scarce" and have added the "Sustaining and improving such an openly accessible infrastructure would highly benefit ML practitioners and researchers from various fields of science." sentence in the "Summary" section to emphasize the importance of this work. In addition, we would like to mention that Galaxy's unlimited computation time with a fixed set of resources provides a clear advantage over the commercial JupyterLab-like environments such as Google Colab in their free versions.
--

- There is still some odd phrasing in the manuscript. Two examples: In the beginning: ML [...] been [...] "vastly used" [...]. The authors referring to CPUs, when they mean

	CPU-cores.<br><br>Author response:<br>Thank you for your comment. We removed the odd phrasing from the manuscript and have accordingly updated it.<br>--<br><br>- One thing that could be improved within Galaxy is making it easier to open the "Active InteractiveTool". There is a "Display" button within the current history sidebar, however clicking that button just seems to return an error. It would be great if this button would directly go to the JupyterLab environment, instead of having to go through "User->Active InteractiveTools".<br><br>Milot Mirdita<br><br><br>Author response:<br>It's a very good comment, thank you. We have integrated this in the new Galaxy release. If the “eye/display” is used icon on your running JupyterLab notebook history entry, you will be directed to your Notebook. Similarly, a new icon (a group of few gear icons) appears on the Galaxy's topbar with quick access to "User->Active InteractiveTools" showing a collection of all accessed Interactive Tools in Galaxy. Further, we have plans for this year to integrate a new “Activity bar” and make Interactive Tools even more visible in Galaxy. Overall, all these UI/UX changes in Galaxy will benefit the GPU-enabled JupyterLab.<br><br>--
**Additional Information:**	
**Question**	**Response**
Are you submitting this manuscript to a special series or article collection?	No
**Experimental design and statistics**<br><br><br>Full details of the experimental design and statistical methods used should be given in the Methods section, as detailed in our Minimum Standards Reporting Checklist. Information essential to interpreting the data presented should be made available in the figure legends.<br><br><br>Have you included all the information requested in your manuscript?	Yes
**Resources**<br><br><br>A description of all resources used, including antibodies, cell lines, animals and software tools, with enough information to allow them to be uniquely identified, should be included in the Methods section. Authors are strongly	Yes

encouraged to cite Research Resource Identifiers (RRIDs) for antibodies, model organisms and tools, where possible.<br><br>Have you included the information requested as detailed in our Minimum Standards Reporting Checklist?	
**Availability of data and materials**<br><br>All datasets and code on which the conclusions of the paper rely must be either included in your submission or deposited in publicly available repositories (where available and ethically appropriate), referencing such data using a unique identifier in the references and in the "Availability of Data and Materials" section of your manuscript.<br><br>Have you have met the above requirement as detailed in our Minimum Standards Reporting Checklist?	Yes

LaTeX - Main Document (TeX file) Click here to access/download;LaTeX - Main Document (TeX file);main.tex

```
This is pdfTeX, Version 3.141592653-2.6-1.40.24 (TeX Live 2022)
(preloaded format=pdflatex 2023.2.8)  11 APR 2023 05:02
entering extended mode
 restricted \write18 enabled.
 %&-line parsing enabled.
**main.tex
(./main.tex
LaTeX2e <2022-11-01> patch level 1
L3 programming layer <2023-02-07> (./oup-contemporary.cls
Document Class: oup-contemporary 2017/06/28, v1.1
(c:/TeXLive/2022/texmf-dist/tex/latex/base/article.cls
Document Class: article 2022/07/02 v1.4n Standard LaTeX document class
(c:/TeXLive/2022/texmf-dist/tex/latex/base/size10.clo
File: size10.clo 2022/07/02 v1.4n Standard LaTeX file (size option)
)
\c@part=\count185
\c@section=\count186
\c@subsection=\count187
\c@subsubsection=\count188
\c@paragraph=\count189
\c@subparagraph=\count190
\c@figure=\count191
\c@table=\count192
\abovecaptionskip=\skip48
\belowcaptionskip=\skip49
\bibindent=\dimen140
) (c:/TeXLive/2022/texmf-dist/tex/latex/base/inputenc.sty
Package: inputenc 2021/02/14 v1.3d Input encoding file
\inpenc@prehook=\toks16
\inpenc@posthook=\toks17
) (c:/TeXLive/2022/texmf-dist/tex/latex/base/fontenc.sty
Package: fontenc 2021/04/29 v2.0v Standard LaTeX package
) (c:/TeXLive/2022/texmf-dist/tex/generic/iftex/ifpdf.sty
Package: ifpdf 2019/10/25 v3.4 ifpdf legacy package. Use iftex instead.
(c:/TeXLive/2022/texmf-dist/tex/generic/iftex/iftex.sty
Package: iftex 2022/02/03 v1.0f TeX engine tests
)) (c:/TeXLive/2022/texmf-dist/tex/latex/microtype/microtype.sty
Package: microtype 2023/03/13 v3.1a Micro-typographical refinements (RS)
(c:/TeXLive/2022/texmf-dist/tex/latex/graphics/keyval.sty
Package: keyval 2022/05/29 v1.15 key=value parser (DPC)
\KV@toks@=\toks18
) (c:/TeXLive/2022/texmf-dist/tex/latex/etoolbox/etoolbox.sty
Package: etoolbox 2020/10/05 v2.5k e-TeX tools for LaTeX (JAW)
\etb@tempcnta=\count193
)
\MT@toks=\toks19
\MT@tempbox=\box51
\MT@count=\count194
LaTeX Info: Redefining \noprotrusionifhmode on input line 1059.
LaTeX Info: Redefining \leftprotrusion on input line 1060.
\MT@prot@toks=\toks20
LaTeX Info: Redefining \rightprotrusion on input line 1078.
LaTeX Info: Redefining \textls on input line 1368.
\MT@outer@kern=\dimen141
```

```
LaTeX Info: Redefining \textmicrotypecontext on input line 1988.
\MT@listname@count=\count195
(c:/TeXLive/2022/texmf-dist/tex/latex/microtype/microtype-pdftex.def
File: microtype-pdftex.def 2023/03/13 v3.1a Definitions specific to
pdftex (RS)

LaTeX Info: Redefining \lsstyle on input line 902.
LaTeX Info: Redefining \lslig on input line 902.
\MT@outer@space=\skip50
)
Package microtype Info: Loading configuration file microtype.cfg.
(c:/TeXLive/2022/texmf-dist/tex/latex/microtype/microtype.cfg
File: microtype.cfg 2023/03/13 v3.1a microtype main configuration file
(RS)
)) (c:/TeXLive/2022/texmf-dist/tex/latex/euler/euler.sty
Package: euler 1995/03/05 v2.5
Package: `euler' v2.5 <1995/03/05> (FJ and FMi)
LaTeX Font Info:    Redeclaring symbol font `letters' on input line 35.
LaTeX Font Info:    Encoding `OML' has changed to `U' for symbol font
(Font)              `letters' in the math version `normal' on input line
35.
LaTeX Font Info:    Overwriting symbol font `letters' in version `normal'
(Font)                  OML/cmm/m/it --> U/eur/m/n on input line 35.
LaTeX Font Info:    Encoding `OML' has changed to `U' for symbol font
(Font)              `letters' in the math version `bold' on input line
35.
LaTeX Font Info:    Overwriting symbol font `letters' in version `bold'
(Font)                  OML/cmm/b/it --> U/eur/m/n on input line 35.
LaTeX Font Info:    Overwriting symbol font `letters' in version `bold'
(Font)                  U/eur/m/n --> U/eur/b/n on input line 36.
LaTeX Font Info:    Redeclaring math symbol \Gamma on input line 47.
LaTeX Font Info:    Redeclaring math symbol \Delta on input line 48.
LaTeX Font Info:    Redeclaring math symbol \Theta on input line 49.
LaTeX Font Info:    Redeclaring math symbol \Lambda on input line 50.
LaTeX Font Info:    Redeclaring math symbol \Xi on input line 51.
LaTeX Font Info:    Redeclaring math symbol \Pi on input line 52.
LaTeX Font Info:    Redeclaring math symbol \Sigma on input line 53.
LaTeX Font Info:    Redeclaring math symbol \Upsilon on input line 54.
LaTeX Font Info:    Redeclaring math symbol \Phi on input line 55.
LaTeX Font Info:    Redeclaring math symbol \Psi on input line 56.
LaTeX Font Info:    Redeclaring math symbol \Omega on input line 57.
\symEulerFraktur=\mathgroup4
LaTeX Font Info:    Overwriting symbol font `EulerFraktur' in version
`bold'
(Font)                  U/euf/m/n --> U/euf/b/n on input line 63.
LaTeX Info: Redefining \oldstylenums on input line 85.
\symEulerScript=\mathgroup5
LaTeX Font Info:    Overwriting symbol font `EulerScript' in version
`bold'
(Font)                  U/eus/m/n --> U/eus/b/n on input line 93.
LaTeX Font Info:    Redeclaring math symbol \aleph on input line 97.
LaTeX Font Info:    Redeclaring math symbol \Re on input line 98.
LaTeX Font Info:    Redeclaring math symbol \Im on input line 99.
LaTeX Font Info:    Redeclaring math delimiter \vert on input line 101.
```

```
LaTeX Font Info:    Redeclaring math delimiter \backslash on input line
103.
LaTeX Font Info:    Redeclaring math symbol \neg on input line 106.
LaTeX Font Info:    Redeclaring math symbol \wedge on input line 108.
LaTeX Font Info:    Redeclaring math symbol \vee on input line 110.
LaTeX Font Info:    Redeclaring math symbol \setminus on input line 112.
LaTeX Font Info:    Redeclaring math symbol \sim on input line 113.
LaTeX Font Info:    Redeclaring math symbol \mid on input line 114.
LaTeX Font Info:    Redeclaring math delimiter \arrowvert on input line
116.
LaTeX Font Info:    Redeclaring math symbol \mathsection on input line
117.
\symEulerExtension=\mathgroup6
LaTeX Font Info:    Redeclaring math symbol \coprod on input line 125.
LaTeX Font Info:    Redeclaring math symbol \prod on input line 125.
LaTeX Font Info:    Redeclaring math symbol \sum on input line 125.
LaTeX Font Info:    Redeclaring math symbol \intop on input line 130.
LaTeX Font Info:    Redeclaring math symbol \ointop on input line 131.
LaTeX Font Info:    Redeclaring math symbol \braceld on input line 132.
LaTeX Font Info:    Redeclaring math symbol \bracerd on input line 133.
LaTeX Font Info:    Redeclaring math symbol \bracelu on input line 134.
LaTeX Font Info:    Redeclaring math symbol \braceru on input line 135.
LaTeX Font Info:    Redeclaring math symbol \infty on input line 136.
LaTeX Font Info:    Redeclaring math symbol \nearrow on input line 153.
LaTeX Font Info:    Redeclaring math symbol \searrow on input line 154.
LaTeX Font Info:    Redeclaring math symbol \nwarrow on input line 155.
LaTeX Font Info:    Redeclaring math symbol \swarrow on input line 156.
LaTeX Font Info:    Redeclaring math symbol \Leftrightarrow on input line
157.
LaTeX Font Info:    Redeclaring math symbol \Leftarrow on input line 158.
LaTeX Font Info:    Redeclaring math symbol \Rightarrow on input line
159.
LaTeX Font Info:    Redeclaring math symbol \leftrightarrow on input line
160.
LaTeX Font Info:    Redeclaring math symbol \leftarrow on input line 161.
LaTeX Font Info:    Redeclaring math symbol \rightarrow on input line
163.
LaTeX Font Info:    Redeclaring math delimiter \uparrow on input line
166.
LaTeX Font Info:    Redeclaring math delimiter \downarrow on input line
168.
LaTeX Font Info:    Redeclaring math delimiter \updownarrow on input line
170.
LaTeX Font Info:    Redeclaring math delimiter \Uparrow on input line
172.
LaTeX Font Info:    Redeclaring math delimiter \Downarrow on input line
174.
LaTeX Font Info:    Redeclaring math delimiter \Updownarrow on input line
176.
LaTeX Font Info:    Redeclaring math symbol \leftharpoonup on input line
177.
LaTeX Font Info:    Redeclaring math symbol \leftharpoondown on input
line 178.
```

```
LaTeX Font Info:    Redeclaring math symbol \rightharpoonup on input line
179.
LaTeX Font Info:    Redeclaring math symbol \rightharpoondown on input
line 180
.
LaTeX Font Info:    Redeclaring math delimiter \lbrace on input line 182.
LaTeX Font Info:    Redeclaring math delimiter \rbrace on input line 184.
\symcmmigroup=\mathgroup7
LaTeX Font Info:    Overwriting symbol font `cmmigroup' in version `bold'
(Font)                  OML/cmm/m/it --> OML/cmm/b/it on input line 200.
LaTeX Font Info:    Redeclaring math accent \vec on input line 201.
LaTeX Font Info:    Redeclaring math symbol \triangleleft on input line
202.
LaTeX Font Info:    Redeclaring math symbol \triangleright on input line
203.
LaTeX Font Info:    Redeclaring math symbol \star on input line 204.
LaTeX Font Info:    Redeclaring math symbol \lhook on input line 205.
LaTeX Font Info:    Redeclaring math symbol \rhook on input line 206.
LaTeX Font Info:    Redeclaring math symbol \flat on input line 207.
LaTeX Font Info:    Redeclaring math symbol \natural on input line 208.
LaTeX Font Info:    Redeclaring math symbol \sharp on input line 209.
LaTeX Font Info:    Redeclaring math symbol \smile on input line 210.
LaTeX Font Info:    Redeclaring math symbol \frown on input line 211.
LaTeX Font Info:    Redeclaring math accent \grave on input line 245.
LaTeX Font Info:    Redeclaring math accent \acute on input line 246.
LaTeX Font Info:    Redeclaring math accent \tilde on input line 247.
LaTeX Font Info:    Redeclaring math accent \ddot on input line 248.
LaTeX Font Info:    Redeclaring math accent \check on input line 249.
LaTeX Font Info:    Redeclaring math accent \breve on input line 250.
LaTeX Font Info:    Redeclaring math accent \bar on input line 251.
LaTeX Font Info:    Redeclaring math accent \dot on input line 252.
LaTeX Font Info:    Redeclaring math accent \hat on input line 254.
) (c:/TeXLive/2022/texmf-dist/tex/latex/merriweather/merriweather.sty
Package: merriweather 2022/09/20 (Bob Tennent) Supports
Merriweather(Sans) font
s for all LaTeX engines.
(c:/TeXLive/2022/texmf-dist/tex/generic/iftex/ifxetex.sty
Package: ifxetex 2019/10/25 v0.7 ifxetex legacy package. Use iftex
instead.
) (c:/TeXLive/2022/texmf-dist/tex/generic/iftex/ifluatex.sty
Package: ifluatex 2019/10/25 v1.5 ifluatex legacy package. Use iftex
instead.
) (c:/TeXLive/2022/texmf-dist/tex/latex/base/textcomp.sty
Package: textcomp 2020/02/02 v2.0n Standard LaTeX package
) (c:/TeXLive/2022/texmf-dist/tex/latex/xkeyval/xkeyval.sty
Package: xkeyval 2022/06/16 v2.9 package option processing (HA)
(c:/TeXLive/2022/texmf-dist/tex/generic/xkeyval/xkeyval.tex
(c:/TeXLive/2022/te
xmf-dist/tex/generic/xkeyval/xkvutils.tex
\XKV@toks=\toks21
\XKV@tempa@toks=\toks22
)
\XKV@depth=\count196
File: xkeyval.tex 2014/12/03 v2.7a key=value parser (HA)
```

```
)) (c:/TeXLive/2022/texmf-dist/tex/latex/base/fontenc.sty
Package: fontenc 2021/04/29 v2.0v Standard LaTeX package
) (c:/TeXLive/2022/texmf-dist/tex/latex/fontaxes/fontaxes.sty
Package: fontaxes 2020/07/21 v1.0e Font selection axes
LaTeX Info: Redefining \upshape on input line 29.
LaTeX Info: Redefining \itshape on input line 31.
LaTeX Info: Redefining \slshape on input line 33.
LaTeX Info: Redefining \swshape on input line 35.
LaTeX Info: Redefining \scshape on input line 37.
LaTeX Info: Redefining \sscshape on input line 39.
LaTeX Info: Redefining \ulcshape on input line 41.
LaTeX Info: Redefining \textsw on input line 47.
LaTeX Info: Redefining \textssc on input line 48.
LaTeX Info: Redefining \textulc on input line 49.
)) (c:/TeXLive/2022/texmf-dist/tex/latex/mathastext/mathastext.sty
Package: mathastext 2022/11/04 v1.3y Use the text font in math mode (JFB)
\mst@exists@muskip=\muskip16
\mst@forall@muskip=\muskip17
\mst@prime@muskip=\muskip18
\mst@do@nonletters=\toks23
\mst@do@easynonletters=\toks24
\mst@do@az=\toks25
\mst@do@AZ=\toks26
\symmtoperatorfont=\mathgroup8
\symmtletterfont=\mathgroup9
** ! and ?
** punctuation: , . : ; and \colon
LaTeX Info: Redefining \relbar on input line 844.
LaTeX Info: Redefining \rightarrowfill on input line 847.
LaTeX Info: Redefining \leftarrowfill on input line 852.
** + and =
LaTeX Info: Redefining \Relbar on input line 943.
** adding = ; and + to \nfss@catcodes
** parentheses ( ) [ ] and slash /
** alldelims: < > \backslash \setminus | \vert \mid \{ and \}
LaTeX Font Info:    Redeclaring math delimiter \backslash on input line
989.
LaTeX Font Info:    Redeclaring math symbol \setminus on input line 1001.
LaTeX Info: Redefining \models on input line 1010.
** \# \mathdollar \% \&
** \imath and \jmath
LaTeX Font Info:    Overwriting math alphabet `\mathnormalbold' in
version `nor
mal'
(Font)                  T1/Merriwthr-OsF/b/it --> T1/Merriwthr-OsF/b/it
on inpu
t line 2370.
LaTeX Font Info:    Overwriting math alphabet `\mathnormalbold' in
version `bol
d'
(Font)                  T1/Merriwthr-OsF/b/it --> T1/Merriwthr-OsF/b/it
on inpu
t line 2370.
```

```
LaTeX Font Info:    Overwriting symbol font `mtletterfont' in version
`normal'
(Font)                  T1/Merriwthr-OsF/m/it --> T1/Merriwthr-OsF/m/it
on inpu
t line 2370.
LaTeX Font Info:    Overwriting symbol font `mtletterfont' in version
`bold'
(Font)                  T1/Merriwthr-OsF/m/it --> T1/Merriwthr-OsF/b/it
on inpu
t line 2370.
LaTeX Font Info:    Overwriting symbol font `mtoperatorfont' in version
`normal
'
(Font)                  T1/Merriwthr-OsF/m/n --> T1/Merriwthr-OsF/m/n on
input
line 2370.
LaTeX Font Info:    Overwriting symbol font `mtoperatorfont' in version
`bold'
(Font)                  T1/Merriwthr-OsF/m/n --> T1/Merriwthr-OsF/b/n on
input
line 2370.
LaTeX Font Info:    Overwriting math alphabet `\Mathbf' in version
`normal'
(Font)                  T1/Merriwthr-OsF/b/n --> T1/Merriwthr-OsF/b/n on
input
line 2370.
LaTeX Font Info:    Overwriting math alphabet `\Mathbf' in version `bold'
(Font)                  T1/Merriwthr-OsF/b/n --> T1/Merriwthr-OsF/b/n on
input
line 2370.
LaTeX Font Info:    Overwriting math alphabet `\Mathit' in version
`normal'
(Font)                  T1/Merriwthr-OsF/m/it --> T1/Merriwthr-OsF/m/it
on inpu
t line 2370.
LaTeX Font Info:    Overwriting math alphabet `\Mathit' in version `bold'
(Font)                  T1/Merriwthr-OsF/m/it --> T1/Merriwthr-OsF/b/it
on inpu
t line 2370.
LaTeX Font Info:    Overwriting math alphabet `\Mathsf' in version
`normal'
(Font)                  T1/MerriwthrSans-OsF/m/n --> T1/MerriwthrSans-
OsF/m/n o
n input line 2370.
LaTeX Font Info:    Overwriting math alphabet `\Mathsf' in version `bold'
(Font)                  T1/MerriwthrSans-OsF/m/n --> T1/MerriwthrSans-
OsF/b/n o
n input line 2370.
LaTeX Font Info:    Overwriting math alphabet `\Mathtt' in version
`normal'
(Font)                  T1/lmtt/m/n --> T1/lmtt/m/n on input line 2370.
LaTeX Font Info:    Overwriting math alphabet `\Mathtt' in version `bold'
(Font)                  T1/lmtt/m/n --> T1/lmtt/b/n on input line 2370.
** Latin letters in the `normal' (resp. `bold') math versions are now
```

```
** set up to use the fonts T1/Merriwthr-OsF/m(b)/it
** Other characters (digits, ...) and \log-like names will be
** typeset with the n shape.
** \hbar
** minus as endash
** \HUGE has been (re)-defined.
** mathastext has declared larger sizes for subscripts.
** To keep LaTeX defaults, use option `defaultmathsizes'.
) (c:/TeXLive/2022/texmf-dist/tex/latex/relsize/relsize.sty
Package: relsize 2013/03/29 ver 4.1
) (c:/TeXLive/2022/texmf-dist/tex/latex/ragged2e/ragged2e.sty
Package: ragged2e 2023/02/25 v3.4 ragged2e Package
\CenteringLeftskip=\skip51
\RaggedLeftLeftskip=\skip52
\RaggedRightLeftskip=\skip53
\CenteringRightskip=\skip54
\RaggedLeftRightskip=\skip55
\RaggedRightRightskip=\skip56
\CenteringParfillskip=\skip57
\RaggedLeftParfillskip=\skip58
\RaggedRightParfillskip=\skip59
\JustifyingParfillskip=\skip60
\CenteringParindent=\skip61
\RaggedLeftParindent=\skip62
\RaggedRightParindent=\skip63
\JustifyingParindent=\skip64
) (c:/TeXLive/2022/texmf-dist/tex/latex/xcolor/xcolor.sty
Package: xcolor 2022/06/12 v2.14 LaTeX color extensions (UK)
(c:/TeXLive/2022/texmf-dist/tex/latex/graphics-cfg/color.cfg
File: color.cfg 2016/01/02 v1.6 sample color configuration
)
Package xcolor Info: Driver file: pdftex.def on input line 227.
(c:/TeXLive/2022/texmf-dist/tex/latex/graphics-def/pdftex.def
File: pdftex.def 2022/09/22 v1.2b Graphics/color driver for pdftex
) (c:/TeXLive/2022/texmf-dist/tex/latex/graphics/mathcolor.ltx)
Package xcolor Info: Model `cmy' substituted by `cmy0' on input line
1353.
Package xcolor Info: Model `hsb' substituted by `rgb' on input line 1357.
Package xcolor Info: Model `RGB' extended on input line 1369.
Package xcolor Info: Model `HTML' substituted by `rgb' on input line
1371.
Package xcolor Info: Model `Hsb' substituted by `hsb' on input line 1372.
Package xcolor Info: Model `tHsb' substituted by `hsb' on input line
1373.
Package xcolor Info: Model `HSB' substituted by `hsb' on input line 1374.
Package xcolor Info: Model `Gray' substituted by `gray' on input line
1375.
Package xcolor Info: Model `wave' substituted by `hsb' on input line
1376.
) (c:/TeXLive/2022/texmf-dist/tex/latex/colortbl/colortbl.sty
Package: colortbl 2022/06/20 v1.0f Color table columns (DPC)
(c:/TeXLive/2022/texmf-dist/tex/latex/tools/array.sty
Package: array 2022/09/04 v2.5g Tabular extension package (FMi)
\col@sep=\dimen142
```

```
\ar@mcellbox=\box52
\extrarowheight=\dimen143
\NC@list=\toks27
\extratabsurround=\skip65
\backup@length=\skip66
\ar@cellbox=\box53
)
\everycr=\toks28
\minrowclearance=\skip67
\rownum=\count197
) (c:/TeXLive/2022/texmf-dist/tex/latex/graphics/graphicx.sty
Package: graphicx 2021/09/16 v1.2d Enhanced LaTeX Graphics (DPC,SPQR)
(c:/TeXLive/2022/texmf-dist/tex/latex/graphics/graphics.sty
Package: graphics 2022/03/10 v1.4e Standard LaTeX Graphics (DPC,SPQR)
(c:/TeXLive/2022/texmf-dist/tex/latex/graphics/trig.sty
Package: trig 2021/08/11 v1.11 sin cos tan (DPC)
) (c:/TeXLive/2022/texmf-dist/tex/latex/graphics-cfg/graphics.cfg
File: graphics.cfg 2016/06/04 v1.11 sample graphics configuration
)
Package graphics Info: Driver file: pdftex.def on input line 107.
)
\Gin@req@height=\dimen144
\Gin@req@width=\dimen145
) (c:/TeXLive/2022/texmf-dist/tex/latex/xpatch/xpatch.sty
(c:/TeXLive/2022/texm
f-dist/tex/latex/l3kernel/expl3.sty
Package: expl3 2023-02-22 L3 programming layer (loader)

! LaTeX Error: Mismatched LaTeX support files detected.
(LaTeX)        Loading 'expl3.sty' aborted!
(LaTeX)
(LaTeX)        The L3 programming layer in the LaTeX format
(LaTeX)        is dated 2023-02-07, but in your TeX tree the files
require
(LaTeX)        at least 2023-02-22.

For immediate help type H <return>.
 ...

l.77      \ExplLoaderFileDate{expl3.sty}}
                                          %

The most likely causes are:
 - A recent format generation failed;
 - A stray format file in the user tree which needs to be removed or
rebuilt;
 - You are running a manually installed version of expl3.sty
   which is incompatible with the version in LaTeX.

LaTeX will abort loading the incompatible support files but this may lead
to
later errors. Please ensure that your LaTeX format is correctly
regenerated.
```

```
)
Package: xpatch 2020/03/25 v0.3a Extending etoolbox patching commands
(c:/TeXLive/2022/texmf-dist/tex/latex/l3packages/xparse/xparse.sty
Package: xparse 2023-02-02 L3 Experimental document command parser
)) (c:/TeXLive/2022/texmf-dist/tex/latex/environ/environ.sty
Package: environ 2014/05/04 v0.3 A new way to define environments
(c:/TeXLive/2022/texmf-dist/tex/latex/trimspaces/trimspaces.sty
Package: trimspaces 2009/09/17 v1.1 Trim spaces around a token list
)
\@envbody=\toks29
) (c:/TeXLive/2022/texmf-dist/tex/latex/lastpage/lastpage.sty
Package: lastpage 2023/03/07 v2.0a lastpage: 2.09 or 2e? (HMM)
(c:/TeXLive/2022/texmf-dist/tex/latex/lastpage/lastpage2e.sty
Package: lastpage2e 2023/03/07 v2.0a Decide which 2e lastpage version to
use (H
MM)
(c:/TeXLive/2022/texmf-dist/tex/latex/lastpage/lastpagemodern.sty
Package: lastpagemodern 2023-03-07 v2.0a Refers to last page's name (HMM;
JPG)
)
)) (c:/TeXLive/2022/texmf-dist/tex/latex/graphics/rotating.sty
Package: rotating 2016/08/11 v2.16d rotated objects in LaTeX
(c:/TeXLive/2022/texmf-dist/tex/latex/base/ifthen.sty
Package: ifthen 2022/04/13 v1.1d Standard LaTeX ifthen package (DPC)
)
\c@r@tfl@t=\count198
\rotFPtop=\skip68
\rotFPbot=\skip69
\rot@float@box=\box54
\rot@mess@toks=\toks30
) (c:/TeXLive/2022/texmf-dist/tex/latex/graphics/lscape.sty
Package: lscape 2020/05/28 v3.02 Landscape Pages (DPC)
) (c:/TeXLive/2022/texmf-dist/tex/latex/tools/afterpage.sty
Package: afterpage 2014/10/28 v1.08 After-Page Package (DPC)
\AP@output=\toks31
\AP@partial=\box55
\AP@footins=\box56
) (c:/TeXLive/2022/texmf-dist/tex/latex/textpos/textpos.sty
Package: textpos 2022/07/23 v1.10.1
Package textpos Info: choosing support for LaTeX3 on input line 60.
\TP@textbox=\box57
\TP@holdbox=\box58
\TPHorizModule=\dimen146
\TPVertModule=\dimen147
\TP@margin=\dimen148
\TP@absmargin=\dimen149
Grid set 16 x 16 = 37.34424pt x 52.81541pt
\TPboxrulesize=\dimen150
\TP@ox=\dimen151
\TP@oy=\dimen152
\TP@tbargs=\toks32
TextBlockOrigin set to 0pt x 0pt
) (c:/TeXLive/2022/texmf-dist/tex/latex/url/url.sty
```

```
\Urlmuskip=\muskip19
Package: url 2013/09/16  ver 3.4  Verb mode for urls, etc.
) (c:/TeXLive/2022/texmf-dist/tex/latex/newfloat/newfloat.sty
Package: newfloat 2019/09/02 v1.1l Defining new floating environments
(AR)
Package newfloat Info: `rotating' package detected.
) (c:/TeXLive/2022/texmf-dist/tex/latex/mdframed/mdframed.sty
Package: mdframed 2013/07/01 1.9b: mdframed
(c:/TeXLive/2022/texmf-dist/tex/latex/kvoptions/kvoptions.sty
Package: kvoptions 2022-06-15 v3.15 Key value format for package options
(HO)
(c:/TeXLive/2022/texmf-dist/tex/generic/ltxcmds/ltxcmds.sty
Package: ltxcmds 2020-05-10 v1.25 LaTeX kernel commands for general use
(HO)
) (c:/TeXLive/2022/texmf-dist/tex/latex/kvsetkeys/kvsetkeys.sty
Package: kvsetkeys 2022-10-05 v1.19 Key value parser (HO)
)) (c:/TeXLive/2022/texmf-dist/tex/latex/zref/zref-abspage.sty
Package: zref-abspage 2022-04-07 v2.34 Module abspage for zref (HO)
(c:/TeXLive/2022/texmf-dist/tex/latex/zref/zref-base.sty
Package: zref-base 2022-04-07 v2.34 Module base for zref (HO)
(c:/TeXLive/2022/texmf-dist/tex/generic/infwarerr/infwarerr.sty
Package: infwarerr 2019/12/03 v1.5 Providing info/warning/error messages
(HO)
) (c:/TeXLive/2022/texmf-dist/tex/generic/kvdefinekeys/kvdefinekeys.sty
Package: kvdefinekeys 2019-12-19 v1.6 Define keys (HO)
) (c:/TeXLive/2022/texmf-dist/tex/generic/pdftexcmds/pdftexcmds.sty
Package: pdftexcmds 2020-06-27 v0.33 Utility functions of pdfTeX for
LuaTeX (HO
)
Package pdftexcmds Info: \pdf@primitive is available.
Package pdftexcmds Info: \pdf@ifprimitive is available.
Package pdftexcmds Info: \pdfdraftmode found.
) (c:/TeXLive/2022/texmf-dist/tex/generic/etexcmds/etexcmds.sty
Package: etexcmds 2019/12/15 v1.7 Avoid name clashes with e-TeX commands
(HO)
) (c:/TeXLive/2022/texmf-dist/tex/latex/auxhook/auxhook.sty
Package: auxhook 2019-12-17 v1.6 Hooks for auxiliary files (HO)
)
Package zref Info: New property list: main on input line 767.
Package zref Info: New property: default on input line 768.
Package zref Info: New property: page on input line 769.
) (c:/TeXLive/2022/texmf-dist/tex/latex/base/atbegshi-ltx.sty
Package: atbegshi-ltx 2021/01/10 v1.0c Emulation of the original atbegshi
package with kernel methods
)
\c@abspage=\count199
Package zref Info: New property: abspage on input line 65.
) (c:/TeXLive/2022/texmf-dist/tex/latex/needspace/needspace.sty
Package: needspace 2010/09/12 v1.3d reserve vertical space
)
\mdf@templength=\skip70
\c@mdf@globalstyle@cnt=\count266
\mdf@skipabove@length=\skip71
\mdf@skipbelow@length=\skip72
```

```
\mdf@leftmargin@length=\skip73
\mdf@rightmargin@length=\skip74
\mdf@innerleftmargin@length=\skip75
\mdf@innerrightmargin@length=\skip76
\mdf@innertopmargin@length=\skip77
\mdf@innerbottommargin@length=\skip78
\mdf@splittopskip@length=\skip79
\mdf@splitbottomskip@length=\skip80
\mdf@outermargin@length=\skip81
\mdf@innermargin@length=\skip82
\mdf@linewidth@length=\skip83
\mdf@innerlinewidth@length=\skip84
\mdf@middlelinewidth@length=\skip85
\mdf@outerlinewidth@length=\skip86
\mdf@roundcorner@length=\skip87
\mdf@footenotedistance@length=\skip88
\mdf@userdefinedwidth@length=\skip89
\mdf@needspace@length=\skip90
\mdf@frametitleaboveskip@length=\skip91
\mdf@frametitlebelowskip@length=\skip92
\mdf@frametitlerulewidth@length=\skip93
\mdf@frametitleleftmargin@length=\skip94
\mdf@frametitlerightmargin@length=\skip95
\mdf@shadowsize@length=\skip96
\mdf@extratopheight@length=\skip97
\mdf@subtitleabovelinewidth@length=\skip98
\mdf@subtitlebelowlinewidth@length=\skip99
\mdf@subtitleaboveskip@length=\skip100
\mdf@subtitlebelowskip@length=\skip101
\mdf@subtitleinneraboveskip@length=\skip102
\mdf@subtitleinnerbelowskip@length=\skip103
\mdf@subsubtitleabovelinewidth@length=\skip104
\mdf@subsubtitlebelowlinewidth@length=\skip105
\mdf@subsubtitleaboveskip@length=\skip106
\mdf@subsubtitlebelowskip@length=\skip107
\mdf@subsubtitleinneraboveskip@length=\skip108
\mdf@subsubtitleinnerbelowskip@length=\skip109
(c:/TeXLive/2022/texmf-dist/tex/latex/mdframed/md-frame-0.mdf
File: md-frame-0.mdf 2013/07/01\ 1.9b: md-frame-0
)
\mdf@frametitlebox=\box59
\mdf@footnotebox=\box60
\mdf@splitbox@one=\box61
\mdf@splitbox@two=\box62
\mdf@splitbox@save=\box63
\mdfsplitboxwidth=\skip110
\mdfsplitboxtotalwidth=\skip111
\mdfsplitboxheight=\skip112
\mdfsplitboxdepth=\skip113
\mdfsplitboxtotalheight=\skip114
\mdfframetitleboxwidth=\skip115
\mdfframetitleboxtotalwidth=\skip116
\mdfframetitleboxheight=\skip117
\mdfframetitleboxdepth=\skip118
```

```
\mdfframetitleboxtotalheight=\skip119
\mdffootnoteboxwidth=\skip120
\mdffootnoteboxtotalwidth=\skip121
\mdffootnoteboxheight=\skip122
\mdffootnoteboxdepth=\skip123
\mdffootnoteboxtotalheight=\skip124
\mdftotallinewidth=\skip125
\mdfboundingboxwidth=\skip126
\mdfboundingboxtotalwidth=\skip127
\mdfboundingboxheight=\skip128
\mdfboundingboxdepth=\skip129
\mdfboundingboxtotalheight=\skip130
\mdf@freevspace@length=\skip131
\mdf@horizontalwidthofbox@length=\skip132
\mdf@verticalmarginwhole@length=\skip133
\mdf@horizontalspaceofbox=\skip134
\mdfsubtitleheight=\skip135
\mdfsubsubtitleheight=\skip136
\c@mdfcountframes=\count267

****** mdframed patching \endmdf@trivlist

****** -- success******

\mdf@envdepth=\count268
\c@mdf@env@i=\count269
\c@mdf@env@ii=\count270
\c@mdf@zref@counter=\count271
Package zref Info: New property: mdf@pagevalue on input line 895.
) (c:/TeXLive/2022/texmf-dist/tex/latex/titlesec/titlesec.sty
Package: titlesec 2021/07/05 v2.14 Sectioning titles
\ttl@box=\box64
\beforetitleunit=\skip137
\aftertitleunit=\skip138
\ttl@plus=\dimen153
\ttl@minus=\dimen154
\ttl@toksa=\toks33
\titlewidth=\dimen155
\titlewidthlast=\dimen156
\titlewidthfirst=\dimen157
) (c:/TeXLive/2022/texmf-dist/tex/latex/koma-script/scrextend.sty
Package: scrextend 2022/10/12 v3.38 KOMA-Script package (extend other classes w
ith features of KOMA-Script classes)
(c:/TeXLive/2022/texmf-dist/tex/latex/koma-script/scrkbase.sty
Package: scrkbase 2022/10/12 v3.38 KOMA-Script package (KOMA-Script-dependent b
asics and keyval usage)
(c:/TeXLive/2022/texmf-dist/tex/latex/koma-script/scrbase.sty
Package: scrbase 2022/10/12 v3.38 KOMA-Script package (KOMA-Script-independent
basics and keyval usage)
(c:/TeXLive/2022/texmf-dist/tex/latex/koma-script/scrlfile.sty
Package: scrlfile 2022/10/12 v3.38 KOMA-Script package (file load hooks)
```

```
(c:/TeXLive/2022/texmf-dist/tex/latex/koma-script/scrlfile-hook.sty
Package: scrlfile-hook 2022/10/12 v3.38 KOMA-Script package (using LaTeX
hooks)

(c:/TeXLive/2022/texmf-dist/tex/latex/koma-script/scrlogo.sty
Package: scrlogo 2022/10/12 v3.38 KOMA-Script package (logo)
)))
Applying: [2021/05/01] Usage of raw or classic option list on input line
252.
Already applied: [0000/00/00] Usage of raw or classic option list on
input line
 368.
))
Package scrextend Info: unexpected definition of `\@makefnmark'.
(scrextend)             Trying to patch it on input line 1709.
Package scrextend Info: patch seems to be successfull on input line 1709.
)

LaTeX Font Warning: Font shape `T1/cmr/m/n' in size <7.5> not available
(Font)              size <7> substituted on input line 65.

(c:/TeXLive/2022/texmf-dist/tex/latex/tools/calc.sty
Package: calc 2017/05/25 v4.3 Infix arithmetic (KKT,FJ)
\calc@Acount=\count272
\calc@Bcount=\count273
\calc@Adimen=\dimen158
\calc@Bdimen=\dimen159
\calc@Askip=\skip139
\calc@Bskip=\skip140
LaTeX Info: Redefining \setlength on input line 80.
LaTeX Info: Redefining \addtolength on input line 81.
\calc@Ccount=\count274
\calc@Cskip=\skip141
) (c:/TeXLive/2022/texmf-dist/tex/latex/geometry/geometry.sty
Package: geometry 2020/01/02 v5.9 Page Geometry
(c:/TeXLive/2022/texmf-dist/tex/generic/iftex/ifvtex.sty
Package: ifvtex 2019/10/25 v1.7 ifvtex legacy package. Use iftex instead.
)
\Gm@cnth=\count275
\Gm@cntv=\count276
\c@Gm@tempcnt=\count277
\Gm@bindingoffset=\dimen160
\Gm@wd@mp=\dimen161
\Gm@odd@mp=\dimen162
\Gm@even@mp=\dimen163
\Gm@layoutwidth=\dimen164
\Gm@layoutheight=\dimen165
\Gm@layouthoffset=\dimen166
\Gm@layoutvoffset=\dimen167
\Gm@dimlist=\toks34
) (c:/TeXLive/2022/texmf-dist/tex/latex/hyperref/hyperref.sty
Package: hyperref 2023-02-07 v7.00v Hypertext links for LaTeX
(c:/TeXLive/2022/texmf-dist/tex/generic/pdfescape/pdfescape.sty
```

```
Package: pdfescape 2019/12/09 v1.15 Implements pdfTeX's escape features
(HO)
) (c:/TeXLive/2022/texmf-dist/tex/latex/hycolor/hycolor.sty
Package: hycolor 2020-01-27 v1.10 Color options for hyperref/bookmark
(HO)
) (c:/TeXLive/2022/texmf-dist/tex/latex/letltxmacro/letltxmacro.sty
Package: letltxmacro 2019/12/03 v1.6 Let assignment for LaTeX macros (HO)
) (c:/TeXLive/2022/texmf-dist/tex/latex/hyperref/nameref.sty
Package: nameref 2022-05-17 v2.50 Cross-referencing by name of section
(c:/TeXLive/2022/texmf-dist/tex/latex/refcount/refcount.sty
Package: refcount 2019/12/15 v3.6 Data extraction from label references
(HO)
) (c:/TeXLive/2022/texmf-
dist/tex/generic/gettitlestring/gettitlestring.sty
Package: gettitlestring 2019/12/15 v1.6 Cleanup title references (HO)
)
\c@section@level=\count278
)
\@linkdim=\dimen168
\Hy@linkcounter=\count279
\Hy@pagecounter=\count280
(c:/TeXLive/2022/texmf-dist/tex/latex/hyperref/pd1enc.def
File: pd1enc.def 2023-02-07 v7.00v Hyperref: PDFDocEncoding definition
(HO)
Now handling font encoding PD1 ...
... no UTF-8 mapping file for font encoding PD1
) (c:/TeXLive/2022/texmf-dist/tex/generic/intcalc/intcalc.sty
Package: intcalc 2019/12/15 v1.3 Expandable calculations with integers
(HO)
)
\Hy@SavedSpaceFactor=\count281
(c:/TeXLive/2022/texmf-dist/tex/latex/hyperref/puenc.def
File: puenc.def 2023-02-07 v7.00v Hyperref: PDF Unicode definition (HO)
Now handling font encoding PU ...
... no UTF-8 mapping file for font encoding PU
)
Package hyperref Info: Option `colorlinks' set `true' on input line 4060.
Package hyperref Info: Hyper figures OFF on input line 4177.
Package hyperref Info: Link nesting OFF on input line 4182.
Package hyperref Info: Hyper index ON on input line 4185.
Package hyperref Info: Plain pages OFF on input line 4192.
Package hyperref Info: Backreferencing OFF on input line 4197.
Package hyperref Info: Implicit mode ON; LaTeX internals redefined.
Package hyperref Info: Bookmarks ON on input line 4425.
\c@Hy@tempcnt=\count282
LaTeX Info: Redefining \url on input line 4763.
\XeTeXLinkMargin=\dimen169
(c:/TeXLive/2022/texmf-dist/tex/generic/bitset/bitset.sty
Package: bitset 2019/12/09 v1.3 Handle bit-vector datatype (HO)
(c:/TeXLive/2022/texmf-dist/tex/generic/bigintcalc/bigintcalc.sty
Package: bigintcalc 2019/12/15 v1.5 Expandable calculations on big
integers (HO
)
))
```

```
\Fld@menulength=\count283
\Field@Width=\dimen170
\Fld@charsize=\dimen171
Package hyperref Info: Hyper figures OFF on input line 6042.
Package hyperref Info: Link nesting OFF on input line 6047.
Package hyperref Info: Hyper index ON on input line 6050.
Package hyperref Info: backreferencing OFF on input line 6057.
Package hyperref Info: Link coloring ON on input line 6060.
Package hyperref Info: Link coloring with OCG OFF on input line 6067.
Package hyperref Info: PDF/A mode OFF on input line 6072.
\Hy@abspage=\count284
\c@Item=\count285
\c@Hfootnote=\count286
)
Package hyperref Info: Driver (autodetected): hpdftex.
(c:/TeXLive/2022/texmf-dist/tex/latex/hyperref/hpdftex.def
File: hpdftex.def 2023-02-07 v7.00v Hyperref driver for pdfTeX
(c:/TeXLive/2022/texmf-dist/tex/latex/base/atveryend-ltx.sty
Package: atveryend-ltx 2020/08/19 v1.0a Emulation of the original
atveryend pac
kage
with kernel methods
)
\HyAnn@Count=\count287
\Fld@listcount=\count288
\c@bookmark@seq@number=\count289
(c:/TeXLive/2022/texmf-dist/tex/latex/rerunfilecheck/rerunfilecheck.sty
Package: rerunfilecheck 2022-07-10 v1.10 Rerun checks for auxiliary files
(HO)
(c:/TeXLive/2022/texmf-dist/tex/generic/uniquecounter/uniquecounter.sty
Package: uniquecounter 2019/12/15 v1.4 Provide unlimited unique counter
(HO)
)
Package uniquecounter Info: New unique counter `rerunfilecheck' on input
line 2
85.
)
\Hy@SectionHShift=\skip142
) (c:/TeXLive/2022/texmf-dist/tex/latex/preprint/authblk.sty
Package: authblk 2001/02/27 1.3 (PWD)
\affilsep=\skip143
\@affilsep=\skip144
\c@Maxaffil=\count290
\c@authors=\count291
\c@affil=\count292
) (c:/TeXLive/2022/texmf-dist/tex/latex/footmisc/footmisc.sty
Package: footmisc 2022/03/08 v6.0d a miscellany of footnote facilities
\FN@temptoken=\toks35
\footnotemargin=\dimen172
\@outputbox@depth=\dimen173
Package footmisc Info: Declaring symbol style bringhurst on input line
695.
Package footmisc Info: Declaring symbol style chicago on input line 703.
Package footmisc Info: Declaring symbol style wiley on input line 712.
```

```
Package footmisc Info: Declaring symbol style lamport-robust on input
line 723.

Package footmisc Info: Declaring symbol style lamport* on input line 743.
Package footmisc Info: Declaring symbol style lamport*-robust on input
line 764
.
) (c:/TeXLive/2022/texmf-dist/tex/latex/fancyhdr/fancyhdr.sty
Package: fancyhdr 2022/11/09 v4.1 Extensive control of page headers and
footers

\f@nch@headwidth=\skip145
\f@nch@O@elh=\skip146
\f@nch@O@erh=\skip147
\f@nch@O@olh=\skip148
\f@nch@O@orh=\skip149
\f@nch@O@elf=\skip150
\f@nch@O@erf=\skip151
\f@nch@O@olf=\skip152
\f@nch@O@orf=\skip153
) (c:/TeXLive/2022/texmf-dist/tex/generic/alphalph/alphalph.sty
Package: alphalph 2019/12/09 v2.6 Convert numbers to letters (HO)
)
\c@authorfn=\count293
(c:/TeXLive/2022/texmf-dist/tex/latex/abstract/abstract.sty
Package: abstract 2009/06/08 v1.2a configurable abstracts
\abstitleskip=\skip154
\absleftindent=\skip155
\absrightindent=\skip156
\absparindent=\skip157
\absparsep=\skip158
)
Package newfloat Info: New float `keypoints' with options
`placement=t!,name=kp
t' on input line 286.
\c@keypoints=\count294
\newfloat@ftype=\count295
Package newfloat Info: float type `keypoints'=8 on input line 286.
(c:/TeXLive/2022/texmf-dist/tex/latex/enumitem/enumitem.sty
Package: enumitem 2019/06/20 v3.9 Customized lists
\labelindent=\skip159
\enit@outerparindent=\dimen174
\enit@toks=\toks36
\enit@inbox=\box65
\enit@count@id=\count296
\enitdp@description=\count297
) (c:/TeXLive/2022/texmf-dist/tex/latex/quoting/quoting.sty
Package: quoting 2014/01/28 v0.1c Consolidated environment for displayed
text
\quo@toppartop=\skip160
) (c:/TeXLive/2022/texmf-dist/tex/latex/sttools/stfloats.sty
Package: stfloats 2017/03/27 v3.3 Improve float mechanism and
baselineskip sett
ings
```

```
\@dblbotnum=\count298
\c@dblbotnumber=\count299
) (c:/TeXLive/2022/texmf-dist/tex/latex/booktabs/booktabs.sty
Package: booktabs 2020/01/12 v1.61803398 Publication quality tables
\heavyrulewidth=\dimen175
\lightrulewidth=\dimen176
\cmidrulewidth=\dimen177
\belowrulesep=\dimen178
\belowbottomsep=\dimen179
\aboverulesep=\dimen180
\abovetopsep=\dimen181
\cmidrulesep=\dimen182
\cmidrulekern=\dimen183
\defaultaddspace=\dimen184
\@cmidla=\count300
\@cmidlb=\count301
\@aboverulesep=\dimen185
\@belowrulesep=\dimen186
\@thisruleclass=\count302
\@lastruleclass=\count303
\@thisrulewidth=\dimen187
) (c:/TeXLive/2022/texmf-dist/tex/latex/tools/tabularx.sty
Package: tabularx 2020/01/15 v2.11c `tabularx' package (DPC)
\TX@col@width=\dimen188
\TX@old@table=\dimen189
\TX@old@col=\dimen190
\TX@target=\dimen191
\TX@delta=\dimen192
\TX@cols=\count304
\TX@ftn=\toks37
)
\enitdp@tablenotes=\count305
(c:/TeXLive/2022/texmf-dist/tex/latex/caption/caption.sty
Package: caption 2022/03/01 v3.6b Customizing captions (AR)
(c:/TeXLive/2022/texmf-dist/tex/latex/caption/caption3.sty
Package: caption3 2022/03/17 v2.3b caption3 kernel (AR)
\caption@tempdima=\dimen193
\captionmargin=\dimen194
\caption@leftmargin=\dimen195
\caption@rightmargin=\dimen196
\caption@width=\dimen197
\caption@indent=\dimen198
\caption@parindent=\dimen199
\caption@hangindent=\dimen256
Package caption Info: Standard document class detected.
)
\c@caption@flags=\count306
\c@continuedfloat=\count307
Package caption Info: hyperref package is loaded.
Package caption Info: rotating package is loaded.
) (c:/TeXLive/2022/texmf-dist/tex/latex/natbib/natbib.sty
Package: natbib 2010/09/13 8.31b (PWD, AO)
\bibhang=\skip161
\bibsep=\skip162
```

```
LaTeX Info: Redefining \cite on input line 694.
\c@NAT@ctr=\count308
)) (c:/TeXLive/2022/texmf-dist/tex/latex/siunitx/siunitx.sty
Package: siunitx 2023-03-04 v3.2.2 A comprehensive (SI) units package
\l__siunitx_angle_tmp_dim=\dimen257
\l__siunitx_angle_marker_box=\box66
\l__siunitx_angle_unit_box=\box67
\l__siunitx_compound_count_int=\count309
(c:/TeXLive/2022/texmf-dist/tex/latex/translations/translations.sty
Package: translations 2022/02/05 v1.12 internationalization of LaTeX2e
packages
 (CN)
)
\l__siunitx_number_exponent_fixed_int=\count310
\l__siunitx_number_min_decimal_int=\count311
\l__siunitx_number_min_integer_int=\count312
\l__siunitx_number_round_precision_int=\count313
\l__siunitx_number_lower_threshold_int=\count314
\l__siunitx_number_upper_threshold_int=\count315
\l__siunitx_number_group_first_int=\count316
\l__siunitx_number_group_size_int=\count317
\l__siunitx_number_group_minimum_int=\count318
(c:/TeXLive/2022/texmf-dist/tex/latex/amsmath/amstext.sty
Package: amstext 2021/08/26 v2.01 AMS text
(c:/TeXLive/2022/texmf-dist/tex/latex/amsmath/amsgen.sty
File: amsgen.sty 1999/11/30 v2.0 generic functions
\@emptytoks=\toks38
\ex@=\dimen258
))
\l__siunitx_table_tmp_box=\box68
\l__siunitx_table_tmp_dim=\dimen259
\l__siunitx_table_column_width_dim=\dimen260
\l__siunitx_table_integer_box=\box69
\l__siunitx_table_decimal_box=\box70
\l__siunitx_table_uncert_box=\box71
\l__siunitx_table_before_box=\box72
\l__siunitx_table_after_box=\box73
\l__siunitx_table_before_dim=\dimen261
\l__siunitx_table_carry_dim=\dimen262
\l__siunitx_unit_tmp_int=\count319
\l__siunitx_unit_position_int=\count320
\l__siunitx_unit_total_int=\count321
)
Package translations Info: No language package found. I am going to use
`englis
h' as default language. on input line 56.
LaTeX Font Info:    Trying to load font information for T1+Merriwthr-OsF
on inp
ut line 56.
(c:/TeXLive/2022/texmf-dist/tex/latex/merriweather/T1Merriwthr-OsF.fd
File: T1Merriwthr-OsF.fd 2020/08/30 (autoinst) Font definitions for
T1/Merriwth
r-OsF.
)
```

```
LaTeX Font Info:    Font shape `T1/Merriwthr-OsF/m/n' will be
(Font)              scaled to size 7.5pt on input line 56.
(c:/TeXLive/2022/texmf-dist/tex/latex/l3backend/l3backend-pdftex.def
File: l3backend-pdftex.def 2023-01-16 L3 backend support: PDF output
(pdfTeX)
\l__color_backend_stack_int=\count322
\l__pdf_internal_box=\box74
) (./main.aux)
\openout1 = `main.aux'.

LaTeX Font Info:    Checking defaults for OML/cmm/m/it on input line 56.
LaTeX Font Info:    ... okay on input line 56.
LaTeX Font Info:    Checking defaults for OMS/cmsy/m/n on input line 56.
LaTeX Font Info:    ... okay on input line 56.
LaTeX Font Info:    Checking defaults for OT1/cmr/m/n on input line 56.
LaTeX Font Info:    ... okay on input line 56.
LaTeX Font Info:    Checking defaults for T1/cmr/m/n on input line 56.
LaTeX Font Info:    ... okay on input line 56.
LaTeX Font Info:    Checking defaults for TS1/cmr/m/n on input line 56.
LaTeX Font Info:    ... okay on input line 56.
LaTeX Font Info:    Checking defaults for OMX/cmex/m/n on input line 56.
LaTeX Font Info:    ... okay on input line 56.
LaTeX Font Info:    Checking defaults for U/cmr/m/n on input line 56.
LaTeX Font Info:    ... okay on input line 56.
LaTeX Font Info:    Checking defaults for PD1/pdf/m/n on input line 56.
LaTeX Font Info:    ... okay on input line 56.
LaTeX Font Info:    Checking defaults for PU/pdf/m/n on input line 56.
LaTeX Font Info:    ... okay on input line 56.
LaTeX Info: Redefining \microtypecontext on input line 56.
Package microtype Info: Applying patch `item' on input line 56.
Package microtype Info: Applying patch `toc' on input line 56.
Package microtype Info: Applying patch `eqnum' on input line 56.

Package microtype Warning: Unable to apply patch `footnote' on input line
56.

Package microtype Info: Applying patch `verbatim' on input line 56.
Package microtype Info: Generating PDF output.
Package microtype Info: Character protrusion enabled (level 2).
Package microtype Info: Using default protrusion set `alltext'.
Package microtype Info: Automatic font expansion enabled (level 2),
(microtype)             stretch: 20, shrink: 20, step: 1, non-selected.
Package microtype Info: Using default expansion set `alltext-nott'.
LaTeX Info: Redefining \showhyphens on input line 56.
Package microtype Info: No adjustment of tracking.
Package microtype Info: No adjustment of interword spacing.
Package microtype Info: No adjustment of character kerning.
Package microtype Info: Loading generic protrusion settings for font
family
(microtype)             `Merriwthr-OsF' (encoding: T1).
(microtype)             For optimal results, create family-specific
settings.
(microtype)             See the microtype manual for details.
LaTeX Font Info:    Redeclaring symbol font `operators' on input line 56.
```

```
LaTeX Font Info:    Encoding `OT1' has changed to `T1' for symbol font
(Font)              `operators' in the math version `normal' on input
line 56.
LaTeX Font Info:    Overwriting symbol font `operators' in version
`normal'
(Font)                  OT1/cmr/m/n --> T1/Merriwthr-OsF/m/up on input
line 56.

LaTeX Font Info:    Encoding `OT1' has changed to `T1' for symbol font
(Font)              `operators' in the math version `bold' on input line
56.
LaTeX Font Info:    Overwriting symbol font `operators' in version `bold'
(Font)                  OT1/cmr/bx/n --> T1/Merriwthr-OsF/m/up on input
line 56
.
LaTeX Font Info:    Overwriting symbol font `operators' in version `bold'
(Font)                  T1/Merriwthr-OsF/m/up --> T1/Merriwthr-OsF/b/up
on inpu
t line 56.
LaTeX Font Info:    Redeclaring math alphabet \mathbf on input line 56.
LaTeX Font Info:    Overwriting math alphabet `\mathbf' in version
`normal'
(Font)                  OT1/cmr/bx/n --> T1/Merriwthr-OsF/b/up on input
line 56
.
LaTeX Font Info:    Overwriting math alphabet `\mathbf' in version `bold'
(Font)                  OT1/cmr/bx/n --> T1/Merriwthr-OsF/b/up on input
line 56
.
LaTeX Font Info:    Redeclaring math alphabet \mathsf on input line 56.
LaTeX Font Info:    Overwriting math alphabet `\mathsf' in version
`normal'
(Font)                  OT1/cmss/m/n --> T1/MerriwthrSans-OsF/m/up on
input lin
e 56.
LaTeX Font Info:    Overwriting math alphabet `\mathsf' in version `bold'
(Font)                  OT1/cmss/bx/n --> T1/MerriwthrSans-OsF/m/up on
input li
ne 56.
LaTeX Font Info:    Redeclaring math alphabet \mathit on input line 56.
LaTeX Font Info:    Overwriting math alphabet `\mathit' in version
`normal'
(Font)                  OT1/cmr/m/it --> T1/Merriwthr-OsF/m/it on input
line 56
.
LaTeX Font Info:    Overwriting math alphabet `\mathit' in version `bold'
(Font)                  OT1/cmr/bx/it --> T1/Merriwthr-OsF/m/it on input
line 5
6.
LaTeX Font Info:    Redeclaring math alphabet \mathtt on input line 56.
LaTeX Font Info:    Overwriting math alphabet `\mathtt' in version
`normal'
(Font)                  OT1/cmtt/m/n --> T1/lmtt/m/up on input line 56.
LaTeX Font Info:    Overwriting math alphabet `\mathtt' in version `bold'
```

```
(Font)                  OT1/cmtt/m/n --> T1/lmtt/m/up on input line 56.
LaTeX Font Info:    Overwriting math alphabet `\mathsf' in version `bold'
(Font)                  T1/MerriwthrSans-OsF/m/up --> T1/MerriwthrSans-
OsF/b/up
 on input line 56.
LaTeX Font Info:    Overwriting math alphabet `\mathit' in version `bold'
(Font)                  T1/Merriwthr-OsF/m/it --> T1/Merriwthr-OsF/b/it
on inpu
t line 56.
\c@mv@tabular=\count323
\c@mv@boldtabular=\count324
(c:/TeXLive/2022/texmf-dist/tex/context/base/mkii/supp-pdf.mkii
[Loading MPS to PDF converter (version 2006.09.02).]
\scratchcounter=\count325
\scratchdimen=\dimen263
\scratchbox=\box75
\nofMPsegments=\count326
\nofMParguments=\count327
\everyMPshowfont=\toks39
\MPscratchCnt=\count328
\MPscratchDim=\dimen264
\MPnumerator=\count329
\makeMPintoPDFobject=\count330
\everyMPtoPDFconversion=\toks40
) (c:/TeXLive/2022/texmf-dist/tex/latex/epstopdf-pkg/epstopdf-base.sty
Package: epstopdf-base 2020-01-24 v2.11 Base part for package epstopdf
Package epstopdf-base Info: Redefining graphics rule for `.eps' on input
line 4
85.
(c:/TeXLive/2022/texmf-dist/tex/latex/latexconfig/epstopdf-sys.cfg
File: epstopdf-sys.cfg 2010/07/13 v1.3 Configuration of (r)epstopdf for
TeX Liv
e
))
*geometry* driver: auto-detecting
*geometry* detected driver: pdftex
*geometry* verbose mode - [ preamble ] result:
* driver: pdftex
* paper: a4paper
* layout: <same size as paper>
* layoutoffset:(h,v)=(0.0pt,0.0pt)
* modes: includefoot twoside
* h-part:(L,W,R)=(54.64pt, 488.22787pt, 54.64pt)
* v-part:(T,H,B)=(66.0pt, 745.04684pt, 34.0pt)
* \paperwidth=597.50787pt
* \paperheight=845.04684pt
* \textwidth=488.22787pt
* \textheight=715.04684pt
* \oddsidemargin=-17.62999pt
* \evensidemargin=-17.62999pt
* \topmargin=-47.76999pt
* \headheight=17.5pt
* \headsep=24.0pt
* \topskip=10.0pt
```

```
* \footskip=30.0pt
* \marginparwidth=48.0pt
* \marginparsep=10.0pt
* \columnsep=18.0pt
* \skip\footins=22.0pt plus 2.0pt
* \hoffset=0.0pt
* \voffset=0.0pt
* \mag=1000
* \@twocolumntrue
* \@twosidetrue
* \@mparswitchtrue
* \@reversemarginfalse
* (1in=72.27pt=25.4mm, 1cm=28.453pt)

Package hyperref Info: Link coloring ON on input line 56.
(./main.out) (./main.out)
\@outlinefile=\write3
\openout3 = `main.out'.

\@gscitedetails=\box76
\@gscitedetailsheight=\skip163
\@gsheadbox=\box77
\@gsheadboxheight=\skip164
LaTeX Font Info:    Font shape `T1/Merriwthr-OsF/b/n' will be
(Font)              scaled to size 6.5pt on input line 56.
LaTeX Font Info:    Calculating math sizes for size <7.5> on input line
56.

LaTeX Font Warning: Font shape `T1/Merriwthr-OsF/m/up' undefined
(Font)              using `T1/Merriwthr-OsF/m/n' instead on input line
56.

LaTeX Font Info:    Font shape `T1/Merriwthr-OsF/m/up' will be
(Font)              scaled to size 6.24973pt on input line 56.
LaTeX Font Info:    Font shape `T1/Merriwthr-OsF/m/up' will be
(Font)              scaled to size 5.24997pt on input line 56.
LaTeX Font Info:    Trying to load font information for U+eur on input
line 56.

(c:/TeXLive/2022/texmf-dist/tex/latex/amsfonts/ueur.fd
File: ueur.fd 2013/01/14 v3.01 Euler Roman
) (c:/TeXLive/2022/texmf-dist/tex/latex/microtype/mt-eur.cfg
File: mt-eur.cfg 2006/07/31 v1.1 microtype config. file: AMS Euler Roman
(RS)
)

LaTeX Font Warning: Font shape `OMS/cmsy/m/n' in size <7.5> not available
(Font)              size <7> substituted on input line 56.

LaTeX Font Info:    External font `cmex10' loaded for size
(Font)              <7.5> on input line 56.
LaTeX Font Info:    External font `cmex10' loaded for size
(Font)              <6.24973> on input line 56.
LaTeX Font Info:    External font `cmex10' loaded for size
```

```
(Font)              <5.24997> on input line 56.
LaTeX Font Info:    Trying to load font information for U+euf on input
line 56.

(c:/TeXLive/2022/texmf-dist/tex/latex/amsfonts/ueuf.fd
File: ueuf.fd 2013/01/14 v3.01 Euler Fraktur
) (c:/TeXLive/2022/texmf-dist/tex/latex/microtype/mt-euf.cfg
File: mt-euf.cfg 2006/07/03 v1.1 microtype config. file: AMS Euler
Fraktur (RS)

)
LaTeX Font Info:    Trying to load font information for U+eus on input
line 56.

(c:/TeXLive/2022/texmf-dist/tex/latex/amsfonts/ueus.fd
File: ueus.fd 2013/01/14 v3.01 Euler Script
) (c:/TeXLive/2022/texmf-dist/tex/latex/microtype/mt-eus.cfg
File: mt-eus.cfg 2006/07/28 v1.2 microtype config. file: AMS Euler Script
(RS)
)
LaTeX Font Info:    Trying to load font information for U+euex on input
line 56
.
(c:/TeXLive/2022/texmf-dist/tex/latex/amsfonts/ueuex.fd
File: ueuex.fd 2013/01/14 v3.01 Euler extra symbols
)

LaTeX Font Warning: Font shape `OML/cmm/m/it' in size <7.5> not available
(Font)              size <7> substituted on input line 56.

LaTeX Font Info:    Font shape `T1/Merriwthr-OsF/m/n' will be
(Font)              scaled to size 6.24973pt on input line 56.
LaTeX Font Info:    Font shape `T1/Merriwthr-OsF/m/n' will be
(Font)              scaled to size 5.24997pt on input line 56.
LaTeX Font Info:    Font shape `T1/Merriwthr-OsF/m/it' will be
(Font)              scaled to size 7.5pt on input line 56.
LaTeX Font Info:    Font shape `T1/Merriwthr-OsF/m/it' will be
(Font)              scaled to size 6.24973pt on input line 56.
LaTeX Font Info:    Font shape `T1/Merriwthr-OsF/m/it' will be
(Font)              scaled to size 5.24997pt on input line 56.
LaTeX Font Info:    Font shape `T1/Merriwthr-OsF/m/n' will be
(Font)              scaled to size 8.0pt on input line 56.
LaTeX Font Info:    Font shape `T1/Merriwthr-OsF/m/it' will be
(Font)              scaled to size 8.0pt on input line 56.
LaTeX Font Info:    Font shape `T1/Merriwthr-OsF/b/it' will be
(Font)              scaled to size 8.0pt on input line 56.
Package caption Info: Begin \AtBeginDocument code.
Package caption Info: End \AtBeginDocument code.

(c:/TeXLive/2022/texmf-dist/tex/latex/translations/translations-basic-
dictionar
y-english.trsl
File: translations-basic-dictionary-english.trsl (english translation
file `tra
```

```
nslations-basic-dictionary')
)
Package translations Info: loading dictionary `translations-basic-dictionary' f
or `english'. on input line 56.
TextBlockOrigin set to 4pc+6.64pt x 4pc+6pt

Overfull \hbox (54.64pt too wide) in paragraph at lines 71--71
[][]
 []

LaTeX Font Info:    Font shape `T1/Merriwthr-OsF/m/n' will be
(Font)              scaled to size 14.0pt on input line 71.
LaTeX Font Info:    Font shape `T1/Merriwthr-OsF/m/n' will be
(Font)              scaled to size 8.99997pt on input line 71.
LaTeX Font Info:    Calculating math sizes for size <14> on input line
71.
LaTeX Font Info:    Font shape `T1/Merriwthr-OsF/m/up' will be
(Font)              scaled to size 14.0pt on input line 71.
LaTeX Font Info:    Font shape `T1/Merriwthr-OsF/m/up' will be
(Font)              scaled to size 11.66617pt on input line 71.
LaTeX Font Info:    Font shape `T1/Merriwthr-OsF/m/up' will be
(Font)              scaled to size 9.79996pt on input line 71.
LaTeX Font Info:    External font `cmex10' loaded for size
(Font)              <14> on input line 71.
LaTeX Font Info:    External font `cmex10' loaded for size
(Font)              <11.66617> on input line 71.
LaTeX Font Info:    External font `cmex10' loaded for size
(Font)              <9.79996> on input line 71.
LaTeX Font Info:    Font shape `T1/Merriwthr-OsF/m/n' will be
(Font)              scaled to size 11.66617pt on input line 71.
LaTeX Font Info:    Font shape `T1/Merriwthr-OsF/m/n' will be
(Font)              scaled to size 9.79996pt on input line 71.
LaTeX Font Info:    Font shape `T1/Merriwthr-OsF/m/it' will be
(Font)              scaled to size 14.0pt on input line 71.
LaTeX Font Info:    Font shape `T1/Merriwthr-OsF/m/it' will be
(Font)              scaled to size 11.66617pt on input line 71.
LaTeX Font Info:    Font shape `T1/Merriwthr-OsF/m/it' will be
(Font)              scaled to size 9.79996pt on input line 71.
LaTeX Font Info:    Font shape `T1/Merriwthr-OsF/b/n' will be
(Font)              scaled to size 18.0pt on input line 71.
LaTeX Font Info:    Font shape `T1/Merriwthr-OsF/m/n' will be
(Font)              scaled to size 13.0pt on input line 71.
LaTeX Font Info:    Calculating math sizes for size <13> on input line
71.
LaTeX Font Info:    Font shape `T1/Merriwthr-OsF/m/up' will be
(Font)              scaled to size 13.0pt on input line 71.
LaTeX Font Info:    Font shape `T1/Merriwthr-OsF/m/up' will be
(Font)              scaled to size 10.83287pt on input line 71.
LaTeX Font Info:    Font shape `T1/Merriwthr-OsF/m/up' will be
(Font)              scaled to size 9.09996pt on input line 71.

LaTeX Font Warning: Font shape `OMS/cmsy/m/n' in size <13> not available
(Font)              size <12> substituted on input line 71.
```

```
LaTeX Font Info:    External font `cmex10' loaded for size
(Font)              <13> on input line 71.
LaTeX Font Info:    External font `cmex10' loaded for size
(Font)              <10.83287> on input line 71.
LaTeX Font Info:    External font `cmex10' loaded for size
(Font)              <9.09996> on input line 71.

LaTeX Font Warning: Font shape `OML/cmm/m/it' in size <13> not available
(Font)              size <12> substituted on input line 71.

LaTeX Font Info:    Font shape `T1/Merriwthr-OsF/m/n' will be
(Font)              scaled to size 10.83287pt on input line 71.
LaTeX Font Info:    Font shape `T1/Merriwthr-OsF/m/n' will be
(Font)              scaled to size 9.09996pt on input line 71.
LaTeX Font Info:    Font shape `T1/Merriwthr-OsF/m/it' will be
(Font)              scaled to size 13.0pt on input line 71.
LaTeX Font Info:    Font shape `T1/Merriwthr-OsF/m/it' will be
(Font)              scaled to size 10.83287pt on input line 71.
LaTeX Font Info:    Font shape `T1/Merriwthr-OsF/m/it' will be
(Font)              scaled to size 9.09996pt on input line 71.
LaTeX Font Info:    Trying to load font information for TS1+Merriwthr-OsF
on in
put line 71.
(c:/TeXLive/2022/texmf-dist/tex/latex/merriweather/TS1Merriwthr-OsF.fd
File: TS1Merriwthr-OsF.fd 2020/08/30 (autoinst) Font definitions for
TS1/Merriw
thr-OsF.
)
LaTeX Font Info:    Font shape `TS1/Merriwthr-OsF/m/n' will be
(Font)              scaled to size 10.83287pt on input line 71.
Package microtype Info: Loading generic protrusion settings for font
family
(microtype)             `Merriwthr-OsF' (encoding: TS1).
(microtype)             For optimal results, create family-specific
settings.
(microtype)             See the microtype manual for details.
LaTeX Font Info:    Font shape `T1/Merriwthr-OsF/m/n' will be
(Font)              scaled to size 9.0pt on input line 71.
LaTeX Font Info:    Font shape `T1/Merriwthr-OsF/m/up' will be
(Font)              scaled to size 9.0pt on input line 71.
LaTeX Font Info:    Font shape `T1/Merriwthr-OsF/m/up' will be
(Font)              scaled to size 7.0pt on input line 71.
LaTeX Font Info:    Font shape `T1/Merriwthr-OsF/m/up' will be
(Font)              scaled to size 5.0pt on input line 71.
LaTeX Font Info:    External font `cmex10' loaded for size
(Font)              <9> on input line 71.
LaTeX Font Info:    External font `cmex10' loaded for size
(Font)              <7> on input line 71.
LaTeX Font Info:    External font `cmex10' loaded for size
(Font)              <5> on input line 71.
LaTeX Font Info:    Font shape `T1/Merriwthr-OsF/m/n' will be
(Font)              scaled to size 7.0pt on input line 71.
LaTeX Font Info:    Font shape `T1/Merriwthr-OsF/m/n' will be
```

```
(Font)              scaled to size 5.0pt on input line 71.
LaTeX Font Info:    Font shape `T1/Merriwthr-OsF/m/it' will be
(Font)              scaled to size 9.0pt on input line 71.
LaTeX Font Info:    Font shape `T1/Merriwthr-OsF/m/it' will be
(Font)              scaled to size 7.0pt on input line 71.
LaTeX Font Info:    Font shape `T1/Merriwthr-OsF/m/it' will be
(Font)              scaled to size 5.0pt on input line 71.
LaTeX Font Info:    Font shape `T1/Merriwthr-OsF/m/n' will be
(Font)              scaled to size 6.5pt on input line 71.
LaTeX Font Info:    Calculating math sizes for size <6.5> on input line
71.
LaTeX Font Info:    Font shape `T1/Merriwthr-OsF/m/up' will be
(Font)              scaled to size 6.5pt on input line 71.
LaTeX Font Info:    Font shape `T1/Merriwthr-OsF/m/up' will be
(Font)              scaled to size 5.41643pt on input line 71.
LaTeX Font Info:    Font shape `T1/Merriwthr-OsF/m/up' will be
(Font)              scaled to size 4.54997pt on input line 71.

LaTeX Font Warning: Font shape `OMS/cmsy/m/n' in size <6.5> not available
(Font)              size <6> substituted on input line 71.


LaTeX Font Warning: Font shape `OMS/cmsy/m/n' in size <5.41643> not
available
(Font)              size <5> substituted on input line 71.


LaTeX Font Warning: Font shape `OMS/cmsy/m/n' in size <4.54997> not
available
(Font)              size <5> substituted on input line 71.

LaTeX Font Info:    External font `cmex10' loaded for size
(Font)              <6.5> on input line 71.
LaTeX Font Info:    External font `cmex10' loaded for size
(Font)              <5.41643> on input line 71.
LaTeX Font Info:    External font `cmex10' loaded for size
(Font)              <4.54997> on input line 71.

LaTeX Font Warning: Font shape `OML/cmm/m/it' in size <6.5> not available
(Font)              size <6> substituted on input line 71.


LaTeX Font Warning: Font shape `OML/cmm/m/it' in size <5.41643> not
available
(Font)              size <5> substituted on input line 71.


LaTeX Font Warning: Font shape `OML/cmm/m/it' in size <4.54997> not
available
(Font)              size <5> substituted on input line 71.

LaTeX Font Info:    Font shape `T1/Merriwthr-OsF/m/n' will be
(Font)              scaled to size 5.41643pt on input line 71.
LaTeX Font Info:    Font shape `T1/Merriwthr-OsF/m/n' will be
```

```
(Font)               scaled to size 4.54997pt on input line 71.
LaTeX Font Info:    Font shape `T1/Merriwthr-OsF/m/it' will be
(Font)               scaled to size 6.5pt on input line 71.
LaTeX Font Info:    Font shape `T1/Merriwthr-OsF/m/it' will be
(Font)               scaled to size 5.41643pt on input line 71.
LaTeX Font Info:    Font shape `T1/Merriwthr-OsF/m/it' will be
(Font)               scaled to size 4.54997pt on input line 71.
LaTeX Font Info:    Font shape `TS1/Merriwthr-OsF/m/n' will be
(Font)               scaled to size 5.41643pt on input line 71.
LaTeX Font Info:    Trying to load font information for T1+lmtt on input
line 7
1.
(c:/TeXLive/2022/texmf-dist/tex/latex/lm/t1lmtt.fd
File: t1lmtt.fd 2015/05/01 v1.6.1 Font defs for Latin Modern
)
Package microtype Info: Loading generic protrusion settings for font
family
(microtype)             `lmtt' (encoding: T1).
(microtype)             For optimal results, create family-specific
settings.
(microtype)             See the microtype manual for details.

Overfull \hbox (54.64pt too wide) in paragraph at lines 71--71
[][][]
 []

LaTeX Font Info:    Font shape `T1/Merriwthr-OsF/b/n' will be
(Font)               scaled to size 10.0pt on input line 71.
LaTeX Font Info:    Font shape `T1/Merriwthr-OsF/b/n' will be
(Font)               scaled to size 8.0pt on input line 71.
LaTeX Font Info:    Font shape `T1/Merriwthr-OsF/m/up' will be
(Font)               scaled to size 8.0pt on input line 71.
LaTeX Font Info:    Font shape `T1/Merriwthr-OsF/m/up' will be
(Font)               scaled to size 6.0pt on input line 71.
LaTeX Font Info:    External font `cmex10' loaded for size
(Font)               <8> on input line 71.
LaTeX Font Info:    External font `cmex10' loaded for size
(Font)               <6> on input line 71.
LaTeX Font Info:    Font shape `T1/Merriwthr-OsF/m/n' will be
(Font)               scaled to size 6.0pt on input line 71.
LaTeX Font Info:    Font shape `T1/Merriwthr-OsF/m/it' will be
(Font)               scaled to size 6.0pt on input line 71.

Overfull \hbox (54.64pt too wide) in paragraph at lines 71--71
[][][]
 []

LaTeX Font Info:    Font shape `T1/Merriwthr-OsF/b/n' will be
(Font)               scaled to size 8.5pt on input line 74.
LaTeX Font Info:    Font shape `T1/Merriwthr-OsF/b/n' will be
(Font)               scaled to size 7.5pt on input line 75.

Package natbib Warning: Citation `fasta-format' on page 1 undefined on
input li
```

```
ne 75.


Package natbib Warning: Citation `KUMAR2022443' on page 1 undefined on 
input li
ne 75.


Package natbib Warning: Citation `kluyver2016jupyter' on page 1 undefined 
on in
put line 75.


Package natbib Warning: Citation `galaxy-nar-2022' on page 1 undefined on 
input
 line 75.


Package natbib Warning: Citation `dockercontainer-galaxy' on page 1 
undefined o
n input line 75.


Package natbib Warning: Citation `interactive-tool' on page 1 undefined 
on inpu
t line 75.


Package natbib Warning: Citation `galaxy-ml-interactive-tool' on page 1 
undefin
ed on input line 75.


Package natbib Warning: Citation `galaxy-resources' on page 1 undefined 
on inpu
t line 75.


Package natbib Warning: Citation `galaxy-denbi' on page 1 undefined on 
input li
ne 75.


Underfull \vbox (badness 10000) has occurred while \output is active []


Underfull \vbox (badness 10000) has occurred while \output is active []

LaTeX Font Info:    Font shape `T1/Merriwthr-OsF/m/n' will be
(Font)              scaled to size 7.8pt on input line 76.
LaTeX Font Info:    Font shape `T1/Merriwthr-OsF/b/n' will be
(Font)              scaled to size 7.8pt on input line 76.
[1{c:/TeXLive/2022/texmf-var/fonts/map/pdftex/updmap/pdftex.map}
```

```
]

Package natbib Warning: Citation `merkel2014docker' on page 2 undefined
on inpu
t line 78.


Package natbib Warning: Citation `docker-security-ibm' on page 2
undefined on i
nput line 78.


Package natbib Warning: Citation `jupyterlab-git' on page 2 undefined on
input
line 78.


Package natbib Warning: Citation `elyra-ai' on page 2 undefined on input
line 7
8.


Package natbib Warning: Citation `tensorflow2015-whitepaper' on page 2
undefine
d on input line 78.


Package natbib Warning: Citation `scikit-learn' on page 2 undefined on
input li
ne 78.


Package natbib Warning: Citation `onnx-bai2019' on page 2 undefined on
input li
ne 78.


Package natbib Warning: Citation `cuda2020' on page 2 undefined on input
line 7
8.


Package natbib Warning: Citation `harris2020array' on page 2 undefined on
input
 line 78.


Package natbib Warning: Citation `2020SciPy-NMeth' on page 2 undefined on
input
 line 78.
```

```
Package natbib Warning: Citation `nvidia-cuda' on page 2 undefined on
input lin
e 78.


Package natbib Warning: Citation `dockerfile' on page 2 undefined on
input line
 79.


Package natbib Warning: Citation `docker-hub' on page 2 undefined on
input line
 79.


Package natbib Warning: Citation `seaborn-plots' on page 2 undefined on
input l
ine 82.


Package natbib Warning: Citation `matplotlib-plots' on page 2 undefined
on inpu
t line 82.


Package natbib Warning: Citation `bokeh-plots' on page 2 undefined on
input lin
e 82.


Package natbib Warning: Citation `opencv_library' on page 2 undefined on
input
line 85.


Package natbib Warning: Citation `van2014scikit' on page 2 undefined on
input l
ine 85.


Package natbib Warning: Citation `nibabel-brett-matthew-2022-6617121' on
page 2
 undefined on input line 85.


Package natbib Warning: Citation `bioblend-10.1093/bioinformatics/btt199'
on pa
ge 2 undefined on input line 85.


Package natbib Warning: Citation `bqplot-jupyter' on page 2 undefined on
input
line 85.
```

```
Package natbib Warning: Citation `voila-jupyter' on page 2 undefined on
input l
ine 85.


Package natbib Warning: Citation `jupyterlab-nvdashboard' on page 2
undefined o
n input line 85.


Package natbib Warning: Citation `hdf5' on page 2 undefined on input line
85.


Package natbib Warning: Citation `colabfold-protein-prediction' on page 2
undef
ined on input line 85.


Package natbib Warning: Citation `jax-google' on page 2 undefined on
input line
 85.


Package natbib Warning: Citation `google-colab-Bisong2019' on page 2
undefined
on input line 88.


Package natbib Warning: Citation `kaggle-kernels' on page 2 undefined on
input
line 88.


Package natbib Warning: Citation `amazon-sagemaker' on page 2 undefined
on inpu
t line 88.


Package natbib Warning: Citation `high_dim_data_1' on page 2 undefined on
input
 line 88.


Package natbib Warning: Citation `high_dim_data_2' on page 2 undefined on
input
 line 88.


Package natbib Warning: Citation `galaxy-resources' on page 2 undefined
on inpu
t line 88.
```

```
LaTeX Font Info:    Font shape `T1/Merriwthr-OsF/m/it' will be
(Font)              scaled to size 7.8pt on input line 89.
[2]

Package natbib Warning: Citation `base-docker-image' on page 3 undefined
on inp
ut line 91.


Package natbib Warning: Citation `Batut_2018' on page 3 undefined on
input line
 91.


Package natbib Warning: Citation `statistics-gpu_jupyter_lab' on page 3
undefin
ed on input line 91.



pdfTeX warning: pdflatex.exe (file ./1_gpu_jupyter_architecture.pdf): PDF
inclu
sion: found PDF version <1.7>, but at most version <1.5> allowed
<1_gpu_jupyter_architecture.pdf, id=131, 418.4433pt x 388.33081pt>
File: 1_gpu_jupyter_architecture.pdf Graphic file (type pdf)
<use 1_gpu_jupyter_architecture.pdf>
Package pdftex.def Info: 1_gpu_jupyter_architecture.pdf  used on input
line 98.

(pdftex.def)             Requested size: 209.22113pt x 194.16492pt.
LaTeX Font Info:    Font shape `T1/Merriwthr-OsF/b/n' will be
(Font)              scaled to size 6.0pt on input line 99.

Package natbib Warning: Citation `base-docker-image' on page 3 undefined
on inp
ut line 99.


Package natbib Warning: Citation `dockercontainer-galaxy' on page 3
undefined o
n input line 99.


Package natbib Warning: Citation `covid-ct-scans-unet' on page 3
undefined on i
nput line 105.


Package natbib Warning: Citation `alphafold2' on page 3 undefined on
input line
 105.

LaTeX Font Info:    Font shape `T1/Merriwthr-OsF/b/n' will be
(Font)              scaled to size 7.0pt on input line 108.
```

Package natbib Warning: Citation `google-colab-Bisong2019' on page 3
undefined
on input line 134.

Package natbib Warning: Citation `kaggle-kernels' on page 3 undefined on
input
line 134.

Package natbib Warning: Citation `covid-ct-scans-unet' on page 3
undefined on i
nput line 138.

Package natbib Warning: Citation `unet-ronneberger' on page 3 undefined
on inpu
t line 138.

Package natbib Warning: Citation `covid-ct-scans-unet' on page 3
undefined on i
nput line 138.

Package natbib Warning: Citation `gpu_jupyterlab_ct_image_segmentation'
on page
3 undefined on input line 138.

Package natbib Warning: Citation `gpu_jupyterlab_ct_image_segmentation'
on page
3 undefined on input line 138.

Package natbib Warning: Citation `covid-ct-scans-h5' on page 3 undefined
on inp
ut line 138.

Package natbib Warning: Citation `tv-unet-split' on page 3 undefined on
input l
ine 138.

Package natbib Warning: Citation `covid-ct-scans-unet' on page 3
undefined on i
nput line 138.

Package natbib Warning: Citation `covid-ct-scans-unet' on page 3
undefined on i
nput line 138.

```
Package natbib Warning: Citation `gpu_jupyterlab_ct_image_segmentation'
on page
 3 undefined on input line 138.


Package natbib Warning: Citation `covid-ct-scans-unet' on page 3
undefined on i
nput line 138.


Package natbib Warning: Citation `gpu_jupyterlab_ct_image_segmentation'
on page
 3 undefined on input line 138.

<2_image_segmentation.pdf, id=132, 659.21594pt x 605.73175pt>
File: 2_image_segmentation.pdf Graphic file (type pdf)
<use 2_image_segmentation.pdf>
Package pdftex.def Info: 2_image_segmentation.pdf  used on input line
145.
(pdftex.def)             Requested size: 230.72903pt x 212.00928pt.

Package natbib Warning: Citation `covid-ct-scans-unet' on page 3
undefined on i
nput line 146.

LaTeX Font Info:    Font shape `T1/Merriwthr-OsF/b/sl' in size <7.5> not
availa
ble
(Font)              Font shape `T1/Merriwthr-OsF/b/it' tried instead on
input l
ine 151.
LaTeX Font Info:    Font shape `T1/Merriwthr-OsF/b/it' will be
(Font)              scaled to size 7.5pt on input line 151.

Package natbib Warning: Citation `mmseqs2' on page 3 undefined on input
line 15
2.


Package natbib Warning: Citation `dockercontainer-galaxy' on page 3
undefined o
n input line 152.


Package natbib Warning: Citation `gpu_jupyterlab_ct_image_segmentation'
on page
 3 undefined on input line 152.


Package natbib Warning: Citation `4Oxalocrotonate-Tautomerase' on page 3
undefi
ned on input line 152.
```

```
Package natbib Warning: Citation `colabfold-alphafold-2' on page 3
undefined on
 input line 152.


Package natbib Warning: Citation `colabfold-protein-prediction' on page 3
undef
ined on input line 152.


Underfull \vbox (badness 10000) has occurred while \output is active []

[3 <./1_gpu_jupyter_architecture.pdf>]

pdfTeX warning: pdflatex.exe (file ./3_3D_structure.pdf): PDF inclusion:
found
PDF version <1.7>, but at most version <1.5> allowed
<3_3D_structure.pdf, id=150, 420.06938pt x 309.40594pt>
File: 3_3D_structure.pdf Graphic file (type pdf)
<use 3_3D_structure.pdf>
Package pdftex.def Info: 3_3D_structure.pdf  used on input line 159.
(pdftex.def)             Requested size: 210.03418pt x 154.70259pt.

Package natbib Warning: Citation `4Oxalocrotonate-Tautomerase' on page 4
undefi
ned on input line 160.


Underfull \vbox (badness 10000) has occurred while \output is active []


Package natbib Warning: Citation `run-jupyterjob-tool' on page 4
undefined on i
nput line 167.


Package natbib Warning: Citation `run-script-job-function' on page 4
undefined
on input line 167.


Package natbib Warning: Citation `remotely-trained-galaxy-history' on
page 4 un
defined on input line 167.


Package natbib Warning: Citation `galaxy-ie-helpers' on page 4 undefined
on inp
ut line 167.
```

```
Package natbib Warning: Citation `gpu_jupyterlab_ct_image_segmentation'
on page
 4 undefined on input line 167.


Package natbib Warning: Citation `gpu_jupyterlab_ct_image_segmentation'
on page
 4 undefined on input line 167.


Underfull \vbox (badness 7468) has occurred while \output is active []

[4 <./2_image_segmentation.pdf> <./3_3D_structure.pdf>]

Package natbib Warning: Citation `dockercontainer-galaxy' on page 5
undefined o
n input line 170.


Package natbib Warning: Citation `statistics-gpu_jupyter_lab' on page 5
undefin
ed on input line 177.


Package natbib Warning: Citation `kubeflow' on page 5 undefined on input
line 1
77.


Package natbib Warning: Citation `apache-airflow' on page 5 undefined on
input
line 177.


Underfull \vbox (badness 10000) has occurred while \output is active []

LaTeX Font Info:    Font shape `T1/Merriwthr-OsF/m/up' will be
(Font)              scaled to size 7.5pt on input line 185.

Underfull \hbox (badness 10000) in paragraph at lines 184--193
\T1/Merriwthr-OsF/m/up/7.5 (+20) Project home page: [][]$\T1/lmtt/m/n/7.5
https
 : / / github . com / usegalaxy-[]eu /
 []


Underfull \hbox (badness 10000) in paragraph at lines 184--193
\T1/Merriwthr-OsF/m/up/7.5 (+20) Galaxy in-ter-ac-tive tool:
[][]$\T1/lmtt/m/n/
7.5 https : / / github . com / usegalaxy-[]eu /
 []


Underfull \hbox (badness 10000) in paragraph at lines 184--193
```

```
\T1/lmtt/m/n/7.5 galaxy / blob / release _ 22 . 05 _ europe / tools /
interacti
ve /
 []


Package natbib Warning: Citation `giga-db' on page 5 undefined on input
line 19
5.

[5]
No file main.bbl.

Package natbib Warning: There were undefined citations.

[6

]
enddocument/afterlastpage: lastpage setting LastPage.
(./main.aux)

LaTeX Font Warning: Size substitutions with differences
(Font)              up to 1.0pt have occurred.


LaTeX Font Warning: Some font shapes were not available, defaults
substituted.

Package rerunfilecheck Info: File `main.out' has not changed.
(rerunfilecheck)             Checksum:
B800D43DF2657029A53B8ED9E04B1FA9;4193.
 )
Here is how much of TeX's memory you used:
 23826 strings out of 476029
 464872 string characters out of 5794165
 1902340 words of memory out of 5000000
 43303 multiletter control sequences out of 15000+600000
 1804167 words of font info for 507 fonts, out of 8000000 for 9000
 1141 hyphenation exceptions out of 8191
 123i,12n,131p,3086b,936s stack positions out of
10000i,1000n,20000p,200000b,200000s
{c:/TeXLive/2022/texmf-
dist/fonts/enc/dvips/merriweather/merriwthr_posqbl.enc
}{c:/TeXLive/2022/texmf-dist/fonts/enc/dvips/lm/lm-
ec.enc}{c:/TeXLive/2022/texm
f-
dist/fonts/enc/dvips/merriweather/merriwthr_owzwzj.enc}<c:/TeXLive/2022/t
exmf
-dist/fonts/type1/sorkin/merriweather/Merriwthr-
Bold.pfb><c:/TeXLive/2022/texmf
-dist/fonts/type1/sorkin/merriweather/Merriwthr-
BoldItalic.pfb><c:/TeXLive/2022
/texmf-dist/fonts/type1/sorkin/merriweather/Merriwthr-
Italic.pfb><c:/TeXLive/20
```

```
22/texmf-dist/fonts/type1/sorkin/merriweather/Merriwthr-
Regular.pfb><c:/TeXLive
/2022/texmf-dist/fonts/type1/public/lm/lmtt8.pfb>
Output written on main.pdf (6 pages, 610449 bytes).
PDF statistics:
 254 PDF objects out of 1000 (max. 8388607)
 203 compressed objects within 3 object streams
 38 named destinations out of 1000 (max. 500000)
 194272 words of extra memory for PDF output out of 221844 (max.
10000000)
```

Click here to access/download;Manuscript;REF.pdf

OXFORD (GIGA)^n SCIENCE

GigaScience, 0000, 1–7

doi: xx.xxxx/xxxx
Manuscript in Preparation
Technical Note

TECHNICAL NOTE

An accessible infrastructure for artificial intelligence using a Docker-based JupyterLab in Galaxy

Anup Kumar[1,*,†], Gianmauro Cuccuru[1,‡,†], Björn Grüning[1,§,†] and Rolf Backofen[1,2,¶,†]

[1]Bioinformatics Group, Department of Computer Science, University of Freiburg, Georges-Koehler-Allee 106, 79110 Freiburg, Germany and [2]Signalling Research Centres BIOSS and CIBSS, University of Freiburg, Schaenzlestr. 18, 79104 Freiburg, Germany

[*]kumara@informatik.uni-freiburg.de https://orcid.org/0000-0002-2068-4695
[‡]gmauro@gmail.com https://orcid.org/0000-0002-5335-545X
[§]gruening@informatik.uni-freiburg.de https://orcid.org/0000-0002-3079-6586
[¶]backofen@informatik.uni-freiburg.de https://orcid.org/0000-0001-8231-3323
[†]Contributions follow the order of the names of authors

Abstract

Background Artificial intelligence (AI) programs that train on large datasets require powerful compute infrastructure consisting of several CPU cores and GPUs. JupyterLab provides an excellent framework for developing AI programs but it needs to be hosted on such an infrastructure to enable faster training of AI programs using parallel computing. **Findings** An open-source, docker-based, and GPU-enabled JupyterLab infrastructure is developed that runs on the public compute infrastructure of Galaxy Europe consisting of thousands of CPU cores, many GPUs and several petabytes (PB) of storage to rapidly prototype and develop end-to-end AI projects. Using a JupyterLab notebook, long-running AI model training programs can also be executed remotely to create trained models, represented in open neural network exchange (ONNX) format, and other output datasets in Galaxy. Other features include Git integration for version control, the option of creating and executing pipelines of notebooks, and multiple dashboards and packages for monitoring compute resources and visualisation, respectively. **Conclusions** These features make JupyterLab in Galaxy Europe highly suitable for creating and managing AI projects. A recent scientific publication that predicts infected regions in COVID-19 CT scan images is reproduced using various features of JupyterLab on Galaxy Europe. In addition, ColabFold, a faster implementation of AlphaFold2, is accessed in JupyterLab to predict the 3D structure of protein sequences. JupyterLab is accessible in two ways - one as an interactive Galaxy tool and the other by running the underlying Docker container. In both ways, long-running training can be executed on Galaxy's compute infrastructure. Scripts to create the Docker container are available under MIT license at https://github.com/usegalaxy-eu/gpu-jupyterlab-docker.

Key words: JupyterLab; Galaxy Europe; Artificial intelligence; Remote model training; ONNX; Elyra AI; GPU; CUDA;

Findings

Background

Bioinformatics comprises many sub-fields such as single-cell, medical imaging, sequencing, proteomics and many more that produce a huge amount of biological data in myriad formats. For example, the single-cell field creates gene expression patterns for each cell that are represented as matrices of real numbers. The medical imaging field generates images of cells and tissues, radiography images such as chest x-rays and computerized tomography (CT) scans. Next-Generation sequencing generates deoxyribonu-

Compiled on: March 22, 2023.
Draft manuscript prepared by the author.

cleic acid (DNA) sequences that are stored as FASTA and FASTQ [1] files. Machine learning (ML) approaches are being increasingly used with these datasets [2] for predictive tasks such as medical diagnosis, imputing missing features, augmenting datasets with artificially generated ones, estimating gene expression patterns and many more. To be able to use ML algorithms on such datasets, a robust and efficient compute infrastructure is needed that can serve multiple purposes. They include pre-processing raw datasets to transform them into suitable formats that are compatible with ML algorithms, creating and executing their complex architectures on pre-processed datasets and making trained models and predicted datasets readily available for further analyses. To facilitate such tasks, a complete infrastructure is developed that combines JupyterLab [3], augmented with many useful features, running on the public compute infrastructure of Galaxy [4] Europe to perform end-to-end AI analyses on scientific datasets. The infrastructure consists of three major components. First, a Docker container [5] that encapsulates JupyterLab together with multiple packages and plugins used for developing AI programs, data manipulation and visualisation. Section S2 in the supplementary file lists all such packages and plugins with their respective versions. Second, a Galaxy interactive tool [6, 7] that downloads this Docker container to serve JupyterLab on Galaxy Europe. Third, the compute infrastructure [8] of Galaxy Europe and the de.NBI cloud [9].

Docker container

Docker [10] containers are popular for shipping packaged software as complete ecosystems, enabling them to be reproducible in a platform-independent manner. Software executing inside a Docker container is abstracted from the host operating system (OS) as most of the requirements necessary for them to run successfully are already configured inside its container. A container runs in an isolated environment having minimal interactions with the host OS. Therefore, running softwares in a container is more secure. Using a Docker container leverages the security benefits necessary for online program editing softwares executing arbitrary code. Arbitrary code may contain some malicious script posing security risks. Using Docker containers can minimise their consequences. Further, in our Docker container, a non-root user is created that can execute and manage projects inside the JupyterLab environment which further minimises security risks. In addition to minimising security risks, Docker containers provide performance benefits compared to running programs on a virtual machine [11]. Motivated by such benefits, a Docker container is used in this project to encapsulate JupyterLab along with many useful packages such as Git [12], Elyra AI [13], TensorFlow-GPU [14], Scikit-learn [15], ONNX [16], and many others. The Docker container inherits many packages such as CUDA [17], NumPy [18], SciPy [19] and a few more from its base container, nvidia/cuda [20], and augments them with many other packages suitable for ML, data manipulation and visualisation. The Docker container is decoupled from Galaxy and can independently be executed for serving JupyterLab with the same set of packages on a different compute infrastructure or any personal computer (PC) or laptop having approximately 25 GB of disk space. Moreover, the Docker container is easily extended by adding the names of packages to the dockerfile [21]. Adding new packages requires the container to be rebuilt and added to Docker hub [22]. The approach to extending the Docker container is discussed in the Methods section.

JupyterLab

JupyterLab is a web-based, robust editor used for varied purposes such as data science, scientific computing and ML. It is a program editor that supports more than 40 programming languages including Python, R, Julia and Scala. Python is one of the most popular languages used by researchers for performing numerous scientific and predictive analyses. Therefore, it is used as the programming language in Galaxy's JupyterLab because many popular packages such as Scikit-learn and TensorFlow for ML, data manipulation packages such as Pandas, visualisation packages such as Seaborn [23], Matplotlib [24], Bokeh [25] and many others are readily available as Python packages. Moreover, the extensible architecture of JupyterLab makes it possible to add many external packages as its plugins such as Git, Elyra AI, dashboards and many others that have a user interface (UI) as necessary components. Such editors, integrated with several useful packages, provide a favourable platform for rapid prototyping and end-to-end development and management of AI projects. To harness the benefits of JupyterLab, it is used as the editor for the interactive tool in Galaxy.

Features of JupyterLab infrastructure

Many features such as easy accessibility, support of a wide variety of programming languages on JupyterLab, and extensibility to install useful plugins make it a desirable editor for researchers for creating project prototypes rapidly. Many such features have been integrated into our JupyterLab infrastructure that is served online on Galaxy Europe enabling researchers to create prototypes and end-to-end AI projects (Figure 1). A few important features are discussed here. To allow GPU computation from JupyterLab, TensorFlow-GPU interacts with Nvidia GPU hardware using another software, CUDA, when the compute resource has GPU(s) for accelerating ML programs. Faster execution of ML programs is one of the significant features of JupyterLab hosted on Galaxy Europe. However, if the hosted machine on which a Docker container runs does not have GPUs, then the program in JupyterLab relies on CPU cores. Other useful features include ONNX for transforming trained TensorFlow and Scikit-learn models to ONNX models, Open-CV [26] and Scikit-image [27] for processing images, Nibabel [28] for reading image files stored as ".nii", Bioblend [29] for accessing Galaxy's datasets, histories and workflows in a JupyterLab notebook and visualisation packages such as Bqplot [30] and Bokeh for plotting interactive charts, Voilà [31] for displaying output cells of a JupyterLab notebook in a different tab, dashboard such as NVDashboard [32] for monitoring GPU usage and performance. Support for file extension such as H5 [33], efficient for storing matrices, enables ML researchers to save model weights and input datasets for AI algorithms. Other packages such as ColabFold [34] together with JAX [35] are used for predicting 3D structures of proteins which are discussed in the Results section. In addition, it is possible to create a long-running training job that runs remotely and stores trained models and output datasets permanently in a newly created Galaxy history. The trained model is saved as an ONNX file and tabular datasets are in an H5 file. It is discussed in the Methods section.

Related infrastructure

There are a few other infrastructures available, free and commercial, that offer JupyterLab or similar environments for developing data science and AI projects. A few popular ones are Google Colab [36], Kaggle Kernel [37] and Amazon Sagemaker [38]. Google Colab is partially free and offers an online editor similar to JupyterLab. The free version of Google Colab offers dynamic compute resources. The disk space is around 70 GB and the memory (RAM) is around 12 GB. AI projects that deal with high-dimensional scientific data [39, 40] may require more resources. In addition, these resources offered by Google Colab are variable and depend on a user's past usage. More compute resources are assigned to those users that have used less in the past for a more equitable sharing of resources. Moreover, there is a limitation of only 12 hours of running time which may be inadequate for training AI models on large datasets needing longer running time. However, Google Colab pro and pro+

offer better compute resources but they come at a price; EUR 9.25 and EUR 42.25 per month, respectively. In contrast, Kaggle Kernel is free of charge but its computing resources are comparable to Colab. The total disk space is approximately 73 GB and RAM is 16 GB for a CPU-based kernel. For the GPU-based kernel, the disk space is of the same size as that of the CPU-based kernel but the RAM of the CPU decreases to 13 GB. An additional RAM of 15 GB is added through a GPU and computation time is limited to 30 hours a week. It also supports TPUs but the computation time is further limited to only 20 hours a week. Amazon Sagemaker is also a commercial software for developing AI algorithms that is free of charge but only for 2 months. Overall, these notebook infrastructures do not offer unrestricted compute resources free of charge. In addition, compute resources offered free of charge can be insufficient for training AI models on high-dimensional scientific datasets. To address the drawbacks of these notebook infrastructures and provide researchers and users with large compute resources more reliably, Galaxy JupyterLab infrastructure [8] offers an unlimited computation time on GPU and many CPU cores for each session as shown in Table 1. The offered resources for JupyterLab running in Galaxy stay constant and are independent of the user's past usage. To make it more useful, JupyterLab opens a tab for each notebook that allows researchers to develop and execute several notebooks inside the same session of the allotted compute resource rather than having them connect to a different session for each notebook as in Google Colab and Kaggle Kernel.

Implementation

JupyterLab infrastructure is developed in two stages. First, a Docker container is created containing all the necessary packages such as JupyterLab itself, CUDA from the base Docker image [41], TensorFlow, Scikit-learn, ONNX and many more. The Docker container is inherited from a base container that has all the necessary CUDA packages installed for working with NVIDIA GPUs. Many packages are added to the Docker container with their compatible versions. Compatible packages for CUDA, CUDA DNN and TensorFlow are necessary so that they together interact with the GPU on the host machine for accelerating ML programs. The versions of all packages installed in the Docker container are listed in Supplementary section S5. Second, the container can be downloaded to any powerful compute infrastructure and JupyterLab can be served in an internet browser via the URL that it generates. In addition, to run this container in Galaxy, an interactive tool is created that downloads this container on a remote compute infrastructure and generates a URL used to run JupyterLab in an internet browser. The architecture of JupyterLab infrastructure in Galaxy is shown in Figure 1. The running instance of JupyterLab in Galaxy contains a default IPython notebook that summarises several of its features. Further, there are other notebooks available, each describing a feature of JupyterLab with code examples such as how to create ONNX models for Scikit-learn and TensorFlow classifiers, how to connect to Galaxy using Bioblend, how to create interactive plots using Bqplots and how to create a pipeline of notebooks using Elyra AI. In addition, the notebooks explaining the use-cases are also available in the Docker container. To access JupyterLab in Galaxy Europe, a ready-to-use hands-on Galaxy training network (GTN) [42] tutorial [43] is developed that shows all the steps such as opening the notebook, using Git to clone a code repository from GitHub, sending long-running training jobs to a remote Galaxy cluster, and how this notebook can be used as a tool in a Galaxy workflow. The approach of remote model training is explained in the Methods section. The two use-cases are also discussed in the tutorial along with their respective notebooks. The steps to access this resource on Galaxy Europe are elaborated in Supplementary section S1.

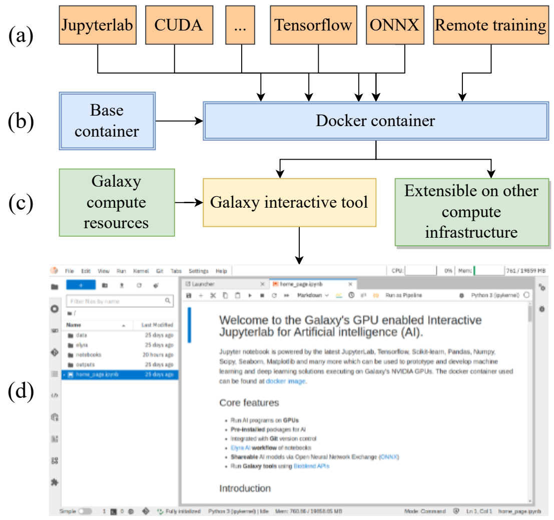

Figure 1. Architecture of Galaxy's JupyterLab. Part (a) shows packages and features wrapped inside a Docker container. Part (b) shows a base Docker container [41] from which the customised container [5] is derived. In part (c), Galaxy's interactive tool downloads the customised container. The customised Docker container can also be hosted on a different compute infrastructure. Part (d) shows Galaxy's JupyterLab.

Results

JupyterLab infrastructure in Galaxy Europe is used to reproduce the results of two recent scientific publications. They demonstrate its robustness and usefulness to develop ML models using COVID CT scan images [44] and predict the 3D structure of proteins using ColabFold, a faster implementation of AlphaFold2 [45].

COVID-19 CT scan image segmentation

In [44], COVID-19 CT scan images have been used to develop and train an ML model architecture that predicts COVID-19 infected regions in those images with high accuracy. An open-source implementation of the work is available that trains a Unet DL architecture [46] distinguishing between normal and infected regions in CT scan images. Scripts of this implementation are adapted and executed on Galaxy's JupyterLab infrastructure. Adaption only involves the transformation of all CT scan images, used in [44], into an H5 file so that they can directly be used as an input to the Unet architecture defined in a notebook available in [47]. All the notebooks available in [47] are also available in the Docker container in the "usecases" directory. A composite H5 file [48] is created using a script [49] that contains multiple datasets inside and each dataset is a real-valued matrix corresponding to the training, test and validation sets as used in [44]. The entire analysis of [44] can be reproduced using multiple notebooks in [47]. They achieve similar precision and recall (approximately 0.98) metrics as mentioned in [44]. In [47], the first notebook (1_fetch_datasets.ipynb) downloads the input dataset as an H5 file. Additionally, it also downloads the trained ONNX model. The second notebook (2_create_model_and_train.ipynb) creates and trains a Unet model on the training dataset extracted from the H5 file. Training, accelerated by GPU, for 10 iterations over the entire training dataset finishes in a few minutes. The third notebook (3_predict_masks.ipynb) extracts the test dataset and predicts infected regions of the CT scan images in the test dataset using the trained model created by the second notebook. Figure 2 shows the comparison of ground truth infected regions in the second column and the predicted infected regions in the third column. A few original CT scan images from the test dataset are shown in the first column of Figure 2.

Table 1. Comparison of Galaxy JupyterLab with other notebook infrastructures

Indicators/Infrastructures	Google Colab [36]	Kaggle Kernel [37]	Galaxy JupyterLab
Memory/Disk space (GB)	12/70	16/73	**20/250**
GPU/TPU	Yes/**Yes**	Yes/**Yes**	**Yes**/No
Max usage time (Hours)	12	12, 30 hrs of GPU/week, 20 hrs of TPU/week	**No time restriction** on GPU and CPU cores usage, notebook sessions and job execution
Dynamic compute resources	Yes	Yes	**Fixed and guaranteed**
Remote model training	No	No	**Yes**
Run multiple notebooks (as tabs) in one session	No	No	**Yes**

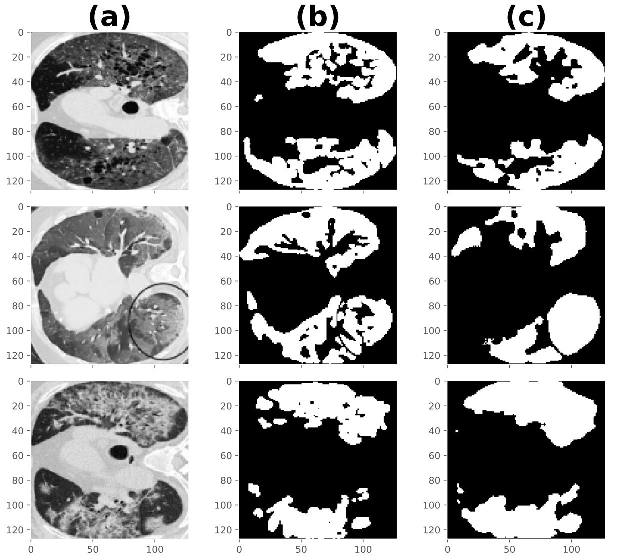

Figure 2. Figure shows original CT scan images in column (a), corresponding ground-truth masks of original CT scan images in column (b) and the predicted masks in column (c). Masks are COVID-19 infected regions in the corresponding CT scan images. The ground-truth and predicted masks show high similarity [44].

Predict 3D structure of proteins using ColabFold

AlphaFold2 has made a breakthrough in predicting the 3D structures of proteins with outstanding accuracy. However, due to their large database size (a few TBs), it is not easily accessible to researchers. Therefore, a few approaches have been developed to replace the time-consuming steps of AlphaFold2 with slightly different steps. They predict 3D structures of proteins with similar accuracy while consuming less memory and time. One such approach is ColabFold which replaces the large database search in AlphaFold2 for finding homologous sequences by a significantly faster (40-60 times) MMseqs2 API [50] call to generate input features based on the query protein sequence. ColabFold's prediction of 3D structures in batches is approximately 90 times faster. It is integrated into the Docker container [5] by adding two packages - ColabFold and GPU-enabled JAX which is a just-in-time compiler for making mathematical transformations. Notebook "7_ColabFold_MMseq2.ipynb" in [47] predicts the 3D structure of a protein sequence using ColabFold by making use of the pre-trained weights of AlphaFold2. Figure 3 shows the 3D structure of 4 Oxalocrotonate Tautomerase [51], a protein sequence of length 62, along with its side chains. This 3D structure is extremely similar to the structure predicted by the JupyterLab notebook [52] from ColabFold [34].

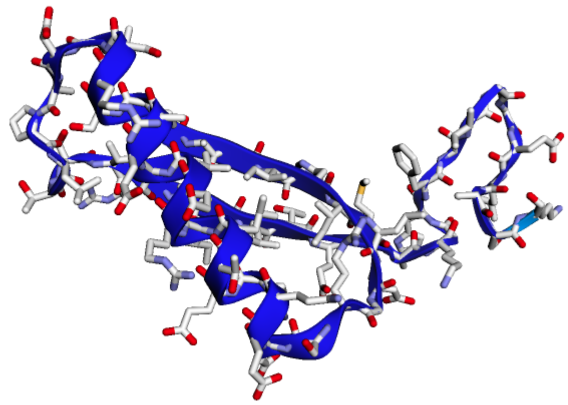

Figure 3. Figures shows a 3D structure of 4 Oxalocrotonate Tautomerase enzyme (protein) [51] predicted by ColabFold

Methods

Remote model training

For large datasets, ML model training may need several hours or even days. In such cases, it would be cumbersome to keep JupyterLab open in a browser's tab till the training finishes. Therefore, another Galaxy tool [53] is developed to enable researchers to send long-running training jobs to a remote Galaxy cluster. The tool can be executed from JupyterLab using a custom Python function [54], part of each JupyterLab notebook, that takes input datasets and a training script as input parameters. The input datasets to be used for training, testing and validation must be provided in H5 format. It allows the standardisation of input data format for AI models that train on matrices in JupyterLab. Input data to an AI model can be in multiple formats such as images, genomic sequences or gene expression patterns. H5 files can be created using any of these data formats and fed to the AI model in JupyterLab. Long-running training happens in a remote Galaxy cluster as a regular Galaxy job. Upon completion of the job, the resulting datasets and the trained model become available in a newly created Galaxy history [55]. The trained model and other resulting datasets can either be downloaded to a local machine or imported from the Galaxy history for further analysis using "get" (for fetching datasets directly into a JupyterLab notebook from Galaxy history) and "put" (for saving datasets directly into a Galaxy history from JupyterLab notebook) methods into a JupyterLab notebook [56]. In [47], a few notebooks are available that showcase the approach of remote model training. Notebook "4_create_model_and_train_remote.ipynb" contains code for developing and training a Unet architecture.

Notebook "5_run_remote_training.ipynb" executes the previous notebook on a Galaxy cluster remotely after creating a Galaxy history and then uploading the script extracted from "4_create_model_and_train_remote.ipynb" notebook and input datasets. Custom Python function, "run_script_job", creates a Galaxy history using Bioblend and then uploads the datasets to the same history. After the upload is finished, the Python script from the specified notebook is executed dynamically. It trains an ML architecture on the uploaded datasets to create a model and saves it as an ONNX file in the Galaxy history. Using "6_predict_masks_remote_model.ipynb" notebook from [47], the trained model can be downloaded from the Galaxy history and used for predicting infected regions of the CT scan images from the test dataset. A significant advantage of training ML models remotely is that researchers don't have to keep the JupyterLab session running as long as the model is being trained as the model training becomes decoupled from JupyterLab. Using such a feature, ML models that take several hours or even days to train can be conveniently trained.

Extend Docker container

The customised Docker container developed as shown in Figure 1 can be easily extended to have more or different packages. To update the container, a package or a list of new packages should be added to the dockerfile and then the new container should be built and pushed to Docker hub [5]. After pushing the new container, when Galaxy's JupyterLab interactive tool is accessed on Galaxy Europe, it downloads the new container and all the newly added packages are available in JupyterLab. Similarly, versions of existing packages can be updated or existing packages can be removed if no longer needed. The simplified extension procedure of the entire infrastructure incurs low maintenance costs as any change to this entire infrastructure is reflected only in the container without updating Galaxy's codebase. In addition, packages can also be added or updated using "pip" in any JupyterLab notebook. But, such changes remain as long as the JupyterLab session runs as they don't update the underlying Docker container.

Collaborative notebooks

Notebooks created in Galaxy's JupyterLab infrastructure can instantly be shared with other researchers and collaborators only by sharing the public URL of a notebook. Researchers and users that share a notebook can collaborate on the same notebook without having to store it anywhere as it is directly served by Galaxy Europe.

Workflow of notebooks

Resembling many tools in Galaxy, JupyterLab can also be used in any Galaxy workflow where it can accept datasets from different tools and then executes an IPython notebook to process the input datasets. It outputs a collection of datasets which can further be used by other Galaxy tools [43]. In addition, using the Elyra AI package, a workflow of notebooks can be created using existing notebooks in a JupyterLab session and executed as one unit of software similarly as Galaxy workflows are created using several tools. It is possible to execute such workflows of notebooks on the same compute resource on which the JupyterLab session runs. In addition, a few other services such as Kubeflow [57] or Apache Airflow [58] can also be used to deploy, run and manage such workflows on a cloud but are not explored in our work.

Summary

JupyterLab is integrated as an interactive tool in Galaxy Europe running on a public and powerful compute infrastructure comprising several CPU cores and GPUs having large memory and disk space. A Docker container is created that wraps JupyterLab along with packages such as TensorFlow-GPU, Scikit-learn, Pandas and many others to provide a robust architecture for the development and management of projects in ML and data science. Remote model training makes it convenient to run multiple analyses in parallel in different Galaxy jobs by executing the same Galaxy tool. The resulting datasets of each job become available in different Galaxy histories. Features such as Git integration are useful for managing entire code repositories on GitHub and Elyra AI for creating pipelines of notebooks working as one software unit. All notebooks created by a user run on the same session of JupyterLab in different tabs. The entire infrastructure of JupyterLab is readily accessible through Galaxy Europe. In contrast to commercial infrastructures that host editors similar to JupyterLab and offer powerful and reliable compute only through paid subscriptions, this infrastructure provides large compute resources free of cost which are invariant to usage and has an unlimited usage time while ensuring a constant amount of compute resources across successive usages. Sustaining and improving such an openly accessible infrastructure would highly benefit ML practitioners and researchers from various fields of science.

Availability of supporting source code and requirements

Project name: GPU-enabled Docker container with JupyterLab for artificial intelligence
Project home page: https://github.com/usegalaxy-eu/gpu-jupyterlab-docker
Galaxy interactive tool: https://github.com/usegalaxy-eu/galaxy/blob/release_22.05_europe/tools/interactive/interactivetool_ml_jupyter_notebook.xml
Operating system: Linux
Programming languages: Python, XML, Docker, Bash
License: MIT License
RRID: SCR_022695
Biotools ID: gpu-enabled_docker_container_with_jupyterlab_for_ai

Additional files

Supplementary Material: An accessible infrastructure for artificial intelligence using a docker-based JupyterLab in Galaxy.

List of abbreviations

AI: Artificial intelligence; CT: Computerised tomography; CUDA: Compute unified device architecture; DL: Deep learning; DNA: Deoxyribonucleic acid; EUR: Euro; GPU: Graphical processing unit; GB: Gigabyte; GTN: Galaxy training network; JAX: Accelerated linear algebra; ML: Machine learning; ONNX: Open neural network exchange; OS: PC: Personal computer; RAM: Random-access memory; TB: Terabyte; PB: Petabyte; UI: User interface; URL: Uniform resource locator;

Competing Interests

The authors declare that they have no competing interests.

Ethics approval and consent to participate

Not applicable

Consent for publication

Not applicable

Funding

This work was supported by the German Research Foundation (DFG) under Germany's Excellence Strategy (CIBSS - EXC-2189 - Project ID 390939984), German Federal Ministry of Education and Research (BMBF grant 031A538A de.NBI) and the European Commission (HORIZON - INFRA - 2021 - EOSC - 01 - EOSC - Euro-ScienceGateway - 101057388).

Authors' contributions

A.K. developed the project and wrote the manuscript. G.C. deployed the project on Galaxy Europe. B.G. devised the idea of the project and helped in creating the resource's access method. R.B. provided the necessary support for the entire project. All authors contributed to and approved the manuscript. R.B. and B.G. provided funding for the project.

Acknowledgements

We thank Daniel Blankenberg for his suggestions to improve the Docker container. In addition, we thank Galaxy Europe team for running and maintaining the project.

References

1. Pearson W, et al, The FASTA package - protein and DNA sequence similarity searching and alignment programs. GitHub; 2016. https://github.com/wrpearson/fasta36. 2016. Accessed 30 June 2022.
2. Kumar I, Singh SP, Shivam. Chapter 26 - Machine learning in bioinformatics. Academic Press 2022;p. 443–456. https://www.sciencedirect.com/science/article/pii/B9780323897754000201.
3. Kluyver T, Ragan-Kelley B, Pérez F, Granger B, Bussonnier M, Frederic J, et al.; IOS Press. Jupyter Notebooks—a publishing format for reproducible computational workflows 2016;p. 87.
4. The Galaxy Community. The Galaxy platform for accessible, reproducible and collaborative biomedical analyses: 2022 update. Nucleic Acids Research 2022 04;50(W1):W345–W351. https://doi.org/10.1093/nar/gkac247.
5. Kumar A, Container for machine learning and deep learning in Jupyter notebook. Docker; 2021. https://hub.docker.com/r/anupkumar/docker-ml-jupyterlab. 2021. Accessed 29 June 2022.
6. Galaxy Europe, Live instance of the European Galaxy server. Galaxy Europe; 2019. https://live.usegalaxy.eu/. 2019. Accessed 30 June 2022.
7. Kumar A, et al, GPU enabled Interactive Jupyter Notebook for Machine Learning; 2021. https://github.com/usegalaxy-eu/galaxy/blob/release_22.05_europe/tools/interactive/interactivetool_ml_jupyter_notebook.xml.
8. Compute resources in Galaxy Europe. GitHub; 2023. https://galaxyproject.org/news/2023-01-24-gpu-jupyterlab-galaxy/#current-resources-will-be-updated-regularly.
9. German Network for Bioinformatics Infrastructure. de.NBI; 2015. https://www.denbi.de/cloud.
10. Merkel D. Docker: lightweight linux containers for consistent development and deployment. Linux journal 2014;2014(239):2.
11. Baset et al , Docker and Container Security White Paper; 2016. https://dominoweb.draco.res.ibm.com/reports/rc25625.pdf.
12. Collonval F, et al (2017), A JupyterLab extension for version control using Git.;. https://github.com/jupyterlab/jupyterlab-git. 2017. Accessed 29 June 2022.
13. Resende L, et al (2018), Elyra is a set of AI-centric extensions to JupyterLab Notebooks.;. https://github.com/elyra-ai/elyra. 2018. Accessed 29 June 2022.
14. Abadi M, et al, TensorFlow: Large-Scale Machine Learning on Heterogeneous Systems; 2015. https://www.tensorflow.org/, software available from tensorflow.org.
15. Pedregosa F, Varoquaux G, Gramfort A, Michel V, Thirion B, Grisel O, et al. Scikit-learn: Machine Learning in Python. Journal of Machine Learning Research 2011;12:2825–2830.
16. Bai J, Lu F, Zhang K, et al, ONNX: Open Neural Network Exchange. GitHub; 2019. https://github.com/onnx/onnx. 2019. Accessed 29 June 2022.
17. NVIDIA, Vingelmann P, Fitzek FHP, CUDA, release: 10.2.89; 2020. https://developer.nvidia.com/cuda-toolkit. 2020. Accessed 29 June 2022.
18. Harris CR, et al. Array programming with NumPy. Nature 2020 September;585(7825):357–362. https://doi.org/10.1038/s41586-020-2649-2.
19. Virtanen P, SciPy 1 0 Contributors. SciPy 1.0: Fundamental Algorithms for Scientific Computing in Python. Nature Methods 2020;17:261–272.
20. NVIDIA Corporation, CUDA and cuDNN images from gitlab.com/nvidia/cuda. Docker; 2014. https://hub.docker.com/r/nvidia/cuda. 2014. Accessed 29 June 2022.
21. Kumar A, Jupyter container used for Data Science and Tensorflow. GitHub; 2021. https://github.com/anuprulez/ml-jupyter-notebook/blob/master/Dockerfile. 2021. Accessed 29 June 2022.
22. Docker Hub. Docker; 2013. https://hub.docker.com/.
23. Waskom ML. seaborn: statistical data visualization. Journal of Open Source Software 2021;6(60):3021. https://doi.org/10.21105/joss.03021.
24. Hunter JD, Matplotlib: A 2D graphics environment. IEEE COMPUTER SOC; 2007.
25. Bokeh Development Team, Bokeh: Python library for interactive visualization. GitHub; 2018. https://bokeh.pydata.org/en/latest/.
26. Bradski G. The OpenCV Library. Dr Dobb's Journal of Software Tools 2000;.
27. Van der Walt S, Schönberger JL, Nunez-Iglesias J, Boulogne F, Warner JD, Yager N, et al. scikit-image: image processing in Python. PeerJ 2014;2:e453.
28. Brett M, et al, nipy/nibabel: 3.2.2. Zenodo; 2022. https://doi.org/10.5281/zenodo.6617121.
29. Sloggett C, Goonasekera N, Afgan E. BioBlend: automating pipeline analyses within Galaxy and CloudMan. Bioinformatics 2013;29(13):1685–1686. https://doi.org/10.1093/bioinformatics/btt199.
30. Corlay, S and et al , 2-D plotting library for Project Jupyter. GitHub; 2015. https://github.com/bqplot/bqplot. 2015. Accessed 29 June 2022.
31. Tuloup, J and et al , Rendering of live Jupyter notebooks with interactive widgets. GitHub; 2018. https://github.com/voila-dashboards/voila. 2018. Accessed 29 June 2022.
32. Tomlinson, J and et al , A JupyterLab extension for displaying GPU usage dashboards. GitHub; 2021. https://github.com/rapidsai/jupyterlab-nvdashboard. 2021. Accessed 29 June 2022.

33. The HDF Group, Hierarchical Data Format, version 5; 1997-2022. https://www.hdfgroup.org/HDF5/. 1997. Accessed 29 June 2022.
34. Mirdita M, Schütze K, Moriwaki Y, et al. ColabFold: making protein folding accessible to all. Nat Methods 2022;19:679–682 (2022). https://doi.org/10.1038/s41592-022-01488-1.
35. Johnson M, et al, JAX: Autograd and XLA; 2020. https://github.com/google/jax. 2020. Accessed 29 June 2022.
36. Bisong E. Google Colaboratory 2019;p. 59–64. https://doi.org/10.1007/978-1-4842-4470-8_7.
37. Kaggle, Kaggle; 2020. https://www.kaggle.com. 2010. Accessed 29 June 2022.
38. Amazon SageMaker, Amazon SageMaker; 2017. https://aws.amazon.com/sagemaker/. 2017. Accessed 29 June 2022.
39. Moon KR, van Dijk D, Wang Zea. Visualizing structure and transitions in high-dimensional biological data. Nat Biotechnol 2019;37:1482–1492 (2019). https://doi.org/10.1038/s41587-019-0336-3.
40. Boileau P, Hejazi NS, Dudoit S. Exploring high-dimensional biological data with sparse contrastive principal component analysis. Bioinformatics 2020;36(11):3422–3430. https://doi.org/10.1093/bioinformatics/btaa176.
41. nvidia/cuda:11.8.0-cudnn8-runtime-ubuntu20.04. Nvidia/Docker; 2014. https://hub.docker.com/layers/nvidia/cuda/11.8.0-cudnn8-runtime-ubuntu20.04/images/sha256-74b166e2091bb705e9ada685dffe79930612c725669bc87e01125b5245d13f97?context=explore.
42. Batut B, et al. Community-Driven Data Analysis Training for Biology. Cell Systems 2018 jun;6(6):752–758.e1. https://doi.org/10.1016/j.cels.2018.05.012.
43. Kumar A, A Docker-based interactive Jupyterlab powered by GPU for artificial intelligence in Galaxy (Galaxy Training Materials); 2022. https://training.galaxyproject.org/training-material/topics/statistics/tutorials/gpu_jupyter_lab/tutorial.html. 2022. Accessed 23 January 2023.
44. Saeedizadeh N, Minaee S, Kafieh R, Yazdani S, Sonka M. COVID TV-Unet: Segmenting COVID-19 chest CT images using connectivity imposed Unet. Computer Methods and Programs in Biomedicine Update 2021;1:100007. https://www.sciencedirect.com/science/article/pii/S2666990021000069.
45. Jumper J, Evans R, Pritzel A, et al. Highly accurate protein structure prediction with AlphaFold. Nature 2021;596:583–589 (2021). https://doi.org/10.1038/s41586-021-03819-2.
46. Ronneberger O, Fischer P, Brox T. U-Net: Convolutional Networks for Biomedical Image Segmentation. CoRR 2015;abs/1505.04597. http://arxiv.org/abs/1505.04597.
47. Kumar A, Jupyterlab notebooks. GitHub; 2022. https://github.com/anuprulez/gpu_jupyterlab_ct_image_segmentation. 2022. Accessed 29 June 2022.
48. Kumar A, COVID Image segmentation datasets and trained model. Zenodo; 2022. https://doi.org/10.5281/zenodo.6091361.
49. Saeedizadeh N, Minaee S, Kafieh R, Yazdani S, Sonka M, COVID TV-Unet: Segmenting COVID-19 chest CT images using connectivity imposed Unet. GitHub; 2021. https://github.com/narges-sa/COVID-CT-Segmentation/blob/main/main_TV_Unet_Split1.py. 2021. Accessed 30 June 2022.
50. Steinegger M, Söding J. MMseqs2 enables sensitive protein sequence searching for the analysis of massive data sets. Nat Biotechnol 2017;35:1026–1028 (2017). https://doi.org/10.1038/nbt.3988.
51. Chen L, Kenyon G, Curtin F, Harayama S, Bembenek M, Hajipour G, et al. 4 Oxalocrotonate tautomerase, an enzyme composed of 62 amino acid residues per monomer. J Biol Chem 1992;267(25):17716–21. https://pubmed.ncbi.nlm.nih.gov/1339435/.
52. Mirdita M, Schütze K, Moriwaki Y, et al, ColabFold: making protein folding accessible to all. GitHub; 2022. https://github.com/sokrypton/ColabFold/blob/main/AlphaFold2.ipynb. 2021. Accessed 30 June 2022.
53. Kumar A, Run long-running jupyterlab script. Github; 2022. https://github.com/bgruening/galaxytools/blob/master/tools/jupyter_job/run_jupyter_job.xml. 2022. Accessed 30 June 2022.
54. Kumar A, Custom jupyterlab notebook function to start model training job in Galaxy. Github; 2021. https://github.com/anuprulez/ml-jupyter-notebook/blob/master/galaxy_script_job.py#L43. 2021. Accessed 30 June 2022.
55. Kumar A, Remotely trained image segmentation model. Galaxy; 2022. https://usegalaxy.eu/u/kumara/h/image-segmentation-from-galaxy-jupyterlab. 2022. Accessed 23 August 2022.
56. Galaxy's Interactive Environments. GitHub; 2016. https://github.com/bgruening/docker-jupyter-notebook/blob/master/default_notebook.ipynb.
57. Kubeflow. Github; 2017. https://github.com/kubeflow/kubeflow.
58. Apache Airflow. Github; 2019. https://github.com/apache/airflow-site.

Click here to access/download
Supplementary Material
Supplementary Material_GPU_Jupyterlab.pdf

Click here to access/download;Personal Cover;Cover letter for Galaxy jupyterlab project.pdf

Dear Dr. Goodman,

We have developed a Jupyterlab notebook infrastructure in Galaxy Europe that is docker-based and GPU-enabled for developing machine learning, deep learning and data science projects. We would like to submit the associated paper titled "An accessible infrastructure for artificial intelligence using a docker-based Jupyterlab in Galaxy" to GigaScience as a technical note. The infrastructure allows artificial intelligence (AI) researchers to rapidly develop their project prototypes and end-to-end projects artificial intelligence projects. There are many interesting aspects of the work which bring it closer to GigaScience:

- **High-quality software**: The infrastructure supports high-quality softwares such as Tensorflow and Scikit-learn for writing AI models; Bokeh and Seaborn for interactive visualizations; Elyra AI for creating workflow of notebooks and Git for managing entire codebases; Bioblend to access Galaxy's tools, datasets and workflows and GPU computation for faster training of AI models.
- **Publicly available**: The infrastructure is available on Galaxy Europe for registered and authorised Galaxy users. The extra authorisation step is necessary to restrict the non-intended usage of public compute resources (especially GPUs) such as bitcoin mining as experienced by us in the past.
- **Shareability and reproducibility**: Jupyterlab notebooks created using this infrastructure promote sharing of analyses among researchers by making them shareable directly via a URL.
- **Extensibility**: Softwares included in the infrastructure are controlled by a docker file and are independent of Galaxy. To extend it to be used in other scientific fields, then only the new set of packages should be installed and the new container should be updated to the docker hub making the infrastructure extensible to different scientific fields beyond Bioinformatics.
- **Unlimited compute time and remote model training**: Researchers can train AI models for hours or even days without caring about when their programs would terminate. Other similar infrastructures such as Google Colab and Kaggle Kernels restrict users to have limited running time (~12 hours). Also, Galaxy's jupyterlab infrastructure provides an approach to train AI models remotely.
- **Example notebooks and tutorials**: Several example notebooks (1, 2) are available for users to learn different aspects of the entire infrastructure. Also, a tutorial is available on how to use this resource.

Potential reviewers of the manuscript:

- Joshua T. Vogelstein: jovo@jhu.edu
- Kelly D Goodwin: Kelly.Goodwin@noaa.gov
- Stephen R Piccolo: stephen_piccolo@byu.edu
- David Landsman: landsman@ncbi.nlm.nih.gov
- Alexej Abyzov: abyzov.alexej@mayo.edu

The authors agree that there are no competing interests and they have approved the manuscript to be submitted to GigaScience. Moreover, the manuscript has not been sent elsewhere. The first version of the manuscript was uploaded to bioRxiv (preprint server).

On behalf of all authors,

Yours sincerely,

Anup Kumar
