# Supplementary material for: An accessible infrastructure for artificial intelligence using a Docker-based JupyterLab in Galaxy
Source: Gigascience. 2023 Apr 26;12:giad028. doi: 10.1093/gigascience/giad028 (PMC10132306; doi:10.1093/gigascience/giad028)
Supplement: giad028_Supplemental_File [file giad028_supplemental_file.pdf]

Supplementary Material: An accessible infrastructure for artificial intelligence using a Docker-based JupyterLab in Galaxy

Anup Kumar [1], Gianmauro Cuccuru [1], Bjoern Gruening [1], and Rolf Backofen [1,2]

[1]Bioinformatics Group, Department of Computer Science, University of Freiburg, Georges-Koehler-Allee 106, 79110 Freiburg, Germany and [2] Signalling Research Centres BIOSS and CIBSS, University of Freiburg, Schaenzlestr. 18, 79104 Freiburg, Germany

Correspondence address: Anup Kumar, Bioinformatics Group, Department of Computer Science, University of Freiburg, Georges-Koehler-Allee 106, 79110 Freiburg, Germany.
E-mail: kumara@informatik.uni-freiburg.de. ORCID: http://orcid.org/0000-0002-2068-4695

S1: Please follow these steps to access this infrastructure

1. Create an account on Galaxy Europe (https://usegalaxy.eu/) using your official university email id
2. Apply for accessing GPU Jupyterlab using this Google form: http://usegalaxy.eu/gpu-request.
3. Use your official university email id in the Google form. This resource is available only for research purposes.
4. Wait up to 1-2 days to get the request approved.
5. Once approved, you will be able to run this resource on Galaxy Europe (https://usegalaxy.eu/).
6. If not authorised, then an error message will be shown.
7. Contact us at: contact@usegalaxy.eu if there are issues.

S2: Galaxy history for remote model training:
https://usegalaxy.eu/u/kumara/h/image-segmentation-from-galaxy-jupyterlab

S3: GPU Jupyterlab tool in a Galaxy workflow:
https://usegalaxy.eu/u/kumara/w/gpujupytool-imported-from-uploaded-file

S4: Galaxy training network tutorial:
https://training.galaxyproject.org/training-material/topics/statistics/tutorials/gpu_jupyter_lab/tutorial.html

S5. List of all supported Python packages in the container with their respective versions (Docker: anupkumar/docker-ml-jupyterlab:galaxy-integration-0.2):

Package	Version
absl-py	0.13.0
aiohttp	3.8.3
aiosignal	1.3.1
alphafold-colabfold	2.1.16
ansiwrap	0.8.4
anyio	3.6.2
appdirs	1.4.4
aquirdturtle-collapsible-headings	3.1.0
argon2-cffi	21.3.0
argon2-cffi-bindings	21.2.0
astroid	2.13.3
asttokens	2.2.1
astunparse	1.6.3
async-timeout	4.0.2
attrs	22.2.0
autopep8	1.6.0
Babel	2.11.0
backcall	0.2.0
beautifulsoup4	4.11.1
bioblend	1.0.0
biopython	1.79
black	22.12.0
bleach	5.0.1
bokeh	2.4.0
bqplot	0.12.36
brotlipy	0.7.0
cachetools	5.2.1

certifi	2022.12.7
cffi	1.14.4
charset-normalizer	2.0.4
chex	0.1.5
click	8.1.3
cloudpickle	2.2.1
colabfold	1.3.0
colorama	0.4.6
coloredlogs	15.0.1
comm	0.1.2
conda	22.11.1
conda-content-trust	0.1.3
conda-package-handling	2.0.2
conda_package_streaming	0.7.0
contextlib2	21.6.0
cryptography	39.0.0
cycler	0.11.0
debugpy	1.6.5
decorator	5.1.1
defusedxml	0.7.1
Deprecated	1.2.13
deprecation	2.1.0
dill	0.3.6
dm-haiku	0.0.9
dm-tree	0.1.8
docker	6.0.1
docstring-parser	0.15
docstring-to-markdown	0.11
elyra	3.14.1

entrypoints	0.4
executing	1.2.0
fastjsonschema	2.16.2
fire	0.5.0
flake8	5.0.4
flatbuffers	2.0.7
frozenlist	1.3.3
future	0.18.3
galaxy-ie-helpers	0.2.7
gast	0.4.0
gitdb	4.0.10
GitPython	3.1.30
google-api-core	2.11.0
google-api-python-client	1.12.11
google-auth	2.16.0
google-auth-httplib2	0.1.0
google-auth-oauthlib	0.4.6
google-cloud-core	2.3.2
google-cloud-storage	2.7.0
google-crc32c	1.5.0
google-pasta	0.2.0
google-resumable-media	2.4.1
googleapis-common-protos	1.58.0
grpcio	1.51.1
h5py	3.7.0
httplib2	0.21.0
humanfriendly	10
idna	3.4
imageio	2.24.0

immutabledict	2.2.3
importlib-metadata	4.13.0
ipykernel	6.20.2
ipython	8.8.0
ipython-genutils	0.2.0
ipywidgets	8.0.4
isort	5.11.4
jax	0.3.25
jaxlib	0.3.25+cuda11.cudnn82
jedi	0.18.2
Jinja2	3.1.2
jmp	0.0.3
joblib	1.2.0
json5	0.9.11
jsonschema	3.2.0
jupyter_client	7.4.9
jupyter_core	4.12.0
jupyter-events	0.4.0
jupyter-lsp	1.5.1
jupyter_packaging	0.12.3
jupyter-resource-usage	0.7.0
jupyter-server	1.16.0
jupyter-server-mathjax	0.2.6
jupyter-server-proxy	3.2.2
jupyterlab	3.4.6
jupyterlab-execute-time	2.3.0
jupyterlab-git	0.39.3
jupyterlab-kernelspy	3.1.0
jupyterlab-lsp	3.10.2

jupyterlab-nvdashboard	0.7.0
jupyterlab-pygments	0.2.2
jupyterlab_server	2.16.3
jupyterlab-system-monitor	0.8.0
jupyterlab-topbar	0.6.1
jupyterlab-widgets	3.0.5
jupytext	1.14.1
keras	2.7.0
Keras-Preprocessing	1.1.2
kfp	1.8.18
kfp-pipeline-spec	0.1.16
kfp-server-api	1.8.5
kiwisolver	1.4.4
kubernetes	19.15.0
lazy-object-proxy	1.9.0
libclang	15.0.6.1
libmambapy	1.2.0
llvmlite	0.39.1
lxml	4.9.2
mamba	1.2.0
Markdown	3.4.1
markdown-it-py	2.1.0
MarkupSafe	2.1.2
matplotlib	3.1.3
matplotlib-inline	0.1.6
mccabe	0.7.0
mdit-py-plugins	0.3.3
mdurl	0.1.2
minio	7.1.13

mistune	0.8.4
ml-collections	0.1.1
mpmath	1.2.1
multidict	6.0.4
mypy-extensions	0.4.3
nbclassic	0.4.8
nbclient	0.5.13
nbconvert	6.5.4
nbdime	3.1.1
nbformat	5.7.3
nest-asyncio	1.5.6
networkx	3
nibabel	4.0.2
notebook	6.5.2
notebook_shim	0.2.2
numba	0.56.4
numpy	1.23.5
oauthlib	3.2.2
onnx	1.12.0
onnx-tf	1.10.0
onnxconverter-common	1.13.0
onnxruntime	1.13.1
opencv-python	4.6.0.66
opt-einsum	3.3.0
packaging	23
pandas	1.5.3
pandocfilters	1.5.0
papermill	2.4.0
parso	0.8.3

pathspec	0.10.3
pexpect	4.8.0
pickleshare	0.7.5
Pillow	9.4.0
pip	22.3.1
platformdirs	2.6.2
pluggy	1.0.0
prometheus-client	0.15.0
prompt-toolkit	3.0.36
protobuf	3.19.6
psutil	5.9.4
ptyprocess	0.7.0
pure-eval	0.2.2
py3Dmol	2.0.0.post2
pyasn1	0.4.8
pyasn1-modules	0.2.8
pycodestyle	2.9.1
pycosat	0.6.4
pycparser	2.21
pydantic	1.10.4
pydocstyle	6.2.3
pyflakes	2.5.0
PyGithub	1.57
Pygments	2.14.0
PyJWT	2.6.0
pylint	2.15.10
PyNaCl	1.5.0
pynvml	11.4.1
pyOpenSSL	22.0.0

pyparsing	3.0.9
pyrsistent	0.19.3
PySocks	1.7.1
python-dateutil	2.8.2
python-json-logger	2.0.4
python-lsp-jsonrpc	1.0.0
python-lsp-server	1.7.1
pytoolconfig	1.2.4
pytz	2022.7.1
PyWavelets	1.4.1
PyYAML	5.4.1
pyzmq	25.0.0
requests	2.28.2
requests-oauthlib	1.3.1
requests-toolbelt	0.10.1
rfc3986-validator	0.1.1
rope	1.7.0
rsa	4.9
ruamel.yaml	0.17.21
ruamel.yaml.clib	0.2.7
scikit-image	0.19.3
scikit-learn	1.1.1
scipy	1.10.0
seaborn	0.12.1
Send2Trash	1.8.0
setuptools	66.0.0
simpervisor	0.4
six	1.16.0
skl2onnx	1.13

smmap	5.0.0
sniffio	1.3.0
snowballstemmer	2.2.0
soupsieve	2.3.2.post1
stack-data	0.6.2
strip-hints	0.1.10
sympy	1.11.1
tabulate	0.9.0
tenacity	8.1.0
tensorboard	2.11.2
tensorboard-data-server	0.6.1
tensorboard-plugin-wit	1.8.1
tensorflow-addons	0.19.0
tensorflow-cpu	2.7.4
tensorflow-estimator	2.7.0
tensorflow-gpu	2.7.0
tensorflow-io-gcs-filesystem	0.30.0
tensorflow-probability	0.15.0
termcolor	2.2.0
terminado	0.17.1
textwrap3	0.9.2
tf2onnx	1.13.0
threadpoolctl	3.1.0
tifffile	2022.10.10
tinycss2	1.2.1
tinydb	4.7.1
toml	0.10.2
tomli	2.0.1
tomlkit	0.11.6

toolz	0.12.0
tornado	6.2
tqdm	4.64.1
traitlets	5.8.1
traittypes	0.2.1
tuspy	1.0.0
typeguard	2.13.3
typer	0.7.0
typing_extensions	4.4.0
ujson	5.7.0
uritemplate	3.0.1
urllib3	1.26.14
voila	0.3.5
watchdog	2.2.1
wcwidth	0.2.6
webencodings	0.5.1
websocket-client	1.4.2
websockets	10.4
Werkzeug	2.2.2
whatthepatch	1.0.3
wheel	0.37.1
widgetsnbextension	4.0.5
wrapt	1.14.1
yapf	0.32.0
yarl	1.8.2
yaspin	2.3.0
zipp	3.11.0
zstandard	0.19.0
