# Supplementary material for: An accessible infrastructure for artificial intelligence using a Docker-based JupyterLab in Galaxy
Source: Gigascience. 2023 Apr 26;12:giad028. doi: 10.1093/gigascience/giad028 (PMC10132306; doi:10.1093/gigascience/giad028)
Supplement: giad028_Response_to_Reviewer_Comments_Original_Submission [file giad028_response_to_reviewer_comments_original_submission.pdf]

Author's Response To Reviewer Comments

Close

GIGA-D-22-00220
An accessible infrastructure for artificial intelligence using a docker-based Jupyterlab in Galaxy
Anup Kumar; Gianmauro Cuccuru; Björn Grüning; Rolf Backofen
GigaScience

Dear Mr. Kumar,

Your manuscript "An accessible infrastructure for artificial intelligence using a docker-based Jupyterlab in Galaxy" (GIGA-D-22-00220) has been assessed by our reviewers. Although it is of interest, we are unable to consider it for publication in its current form. The reviewers have raised a number of points which we believe would improve the manuscript and may allow a revised version to be published in GigaScience.

Author response:
We thank the editor and reviewers for their encouragement, valuable time and useful reviews. We have fixed all the review comments and our responses are as follows.

They also mentioned this manuscript may need a professional editing service, please make sure the language is checked carefully before re-sub.
Note: The comments from reviewer1 was uploaded as an attachment.

Author response:
Thank you for your comment. We have improved the language of the manuscript.

Their reports, together with any other comments, are below. Please also take a moment to check our website at https://www.editorialmanager.com/giga/ for any additional comments that were saved as attachments.

In addition, please register any new software application in the bio.tools and SciCrunch.org databases to receive RRID (Research Resource Identification Initiative ID) and biotoolsID identifiers, and include these in your manuscript. Computational workflows should be registered in workflowhub.eu and the DOIs cited in the relevant places in the manuscript. These will facilitate tracking, reproducibility and re-use of your tool.

Author response:
We have added the resource to SciCrunch.org (with RRID: SCR_022695) and also to bio.tools (ID: gpu-enabled_docker_container_with_jupyterlab_for_ai, link: https://bio.tools/gpu-enabled_docker_container_with_jupyterlab_for_ai). These have also been mentioned in the manuscript.

If you are able to fully address these points, we would encourage you to submit a revised manuscript to GigaScience. Once you have made the necessary corrections, please submit online at:

https://www.editorialmanager.com/giga/

If you have forgotten your username or password please use the "Send Login Details" link to get your login

information. For security reasons, your password will be reset.

Please include a point-by-point within the 'Response to Reviewers' box in the submission system. Please ensure you describe additional experiments that were carried out and include a detailed rebuttal of any criticisms or requested revisions that you disagreed with. Please also ensure that your revised manuscript conforms to the journal style, which can be found in the Instructions for Authors on the journal homepage. If the data and code has been modified in the revision process please be sure to update the public versions of this too.

The due date for submitting the revised version of your article is 24 Jan 2023.

I look forward to receiving your revised manuscript soon.

Best wishes,

Hongling Zhou
GigaScience
www.gigasciencejournal.com

Reviewer reports:

Reviewer #1:

Review: “An accessible infrastructure for artificial intelligence using a docker-based Jupyterlab in Galaxy”

September 8, 2022

This manuscript introduces the new docker-based JupyterLab framework in Galaxy, describing its core components and demonstrating its use in the reproduction of two analyses. The proposed framework is also thoroughly compared to competitors, like Google’s Colab and Amazon’s SageMaker. This tool is bound to have an impact on the life sciences: it democratizes computational analyses and facilitates reproducibility. I thank the authors for their important work.

Author response:
Thank you for your encouragement and review comments.

However, I think that this technical note should be reviewed for grammatical errors and faulty punctuation. I’ve identified some such issues in the comments below but wasn’t able to address all of them. Included in the comments are other remarks which, if addressed, could strengthen some key takeaways.

Author response:
We have updated the manuscript based on the review comments. Our responses are as follows.

- The first sentence of the abstract states that AI programs require “powerful compute infrastructure” when applied to large datasets. I think readers would like to know how you qualify an infrastructure as “powerful”. A brief definition could be included in the second sentence instead of repeating “. . . hosted on a powerful infrastructure . . . ”.

Author response:
We have removed the redundant "powerful" word and quantified the powerful infrastructure in the Abstract as well as at appropriate places in the manuscript.

- Is it “JupyterLab” or “jupyterlab notebook”? The Project Jupyter site seems to use the former. Based on the documentation, JupyterLab is a web-based user interface that can open Jupyter notebooks (.ipynb files).

Author response:
We have used the official name of JupyterLab and have made it consistent in the manuscript.

- The statement “Artificial intelligence (AI) approaches such as machine learning (ML) and deep learning (DL) . . . ” implies that ML and DL are distinct aspects of AI. This distinction is insinuated throughout the rest of text. Isn’t DL a subset of ML? I suggest replacing “ML and DL algorithms” by “ML algorithms” and specifying “DL algorithms” only as needed.

Author response: Yes, we agree that DL is a subset of ML. We have made the required changes in the manuscript and have mostly used the term ML and DL only at the required places.

- I believe there’s a missing comma between “ecosystems” and “enabling” in the first sentence of the Docker container section.

Author response:
We have made this change in the manuscript.

- Consider reformatting “A container runs . . . of the running software.” to “A container runs an isolated environment with minimal interactions between it and the host OS. Running software in a container is more secure.”

Author response:
This has been fixed in the manuscript.

- Related to the suggestion above: Can you explain why this increased security is necessary? An example might help emphasize the importance of a secure container.

Author response:
Docker containers provide several security benefits for Galaxy's interactive tools as these interactive tools running on Galaxy's remote machines (part of compute infrastructure) let users run computer programs using various programming languages such as Python, R and so on. Executing arbitrary programs on host machines may induce security threats and maliciously written programs can claim the root of these host machines. To prevent such access, Docker containers are being used that maintain minimum interactions with the host operating system. Arbitrary code written inside for example JupyterLab notebook runs within the container instead of directly on the host machine. Moreover, the container runs with a non-root user which provides additional security benefits.

We have added a similar explanation to the manuscript in "Docker container" section.

- I think “Docker container inherits . . . ” should be “The Docker container inherits . . . ”. Same goes for “Docker container is decoupled . . . ”.

Author response:
This has been fixed by using "The Docker container" or "a Docker container" appropriately at different places in the manuscript.

- Consider reformatting “Moreover, it can easily be extended by installing suitable packages only by adding their appropriate package names in its dockerfile.” to “Moreover, the Docker
container is easily extended: additional software packages can be installed by adding their names to the dockerfile.”

Author response:
This has been fixed in the manuscript.

- Consider replacing “some of the popular ones are” by “including”

Author response:
This has been fixed in the manuscript.

- I believe there’s an unneeded comma between “. . . platform for both” and “rapid prototyping. . . ”.

Author response:
This has been fixed in the manuscript.

- I believe that there’s missing a word in the last sentence of the Features of jupyterlab and notebook infrastructure section: “. . . an H5 file.”

Author response:
This has been fixed in the manuscript.

- “google” and “amazon” should be capitalized.

Author response:
The names of Google, Amazon and Kaggle have been capitalized in the manuscript.

- Consider removing “and non-ideal” from Related infrastructure section.

Author response:
Yes, we agree that this phrase is not suitable. The phrase "and non-ideal" has been removed from the manuscript.

-I believe the comma in “. . . but they come at a price, . . . ” should be replace by a colon.

Author response:
This has been fixed in the manuscript.

-I believe there's missing a comma between ". . . free of charge" and "similar to colab . . . ".

Author response:
This has been fixed in the manuscript.

- Why is sharing a sessions's resources across multiple notebooks more useful than operating each notebook in a separate session? Isn't the latter preferable when a notebook causes a session to crash?

Author response:
This is a good comment. Being able to run multiple notebooks together can help in multiple ways. For example, if there is a notebook that needs the output of two different notebooks (let's assume these two can be executed in parallel), then it would be faster to execute them independently by sharing GPU/CPU resources and making all the intermediate datasets available for the third notebook. In another case, two notebooks can be executed one after another when the second notebook requires datasets produced by the first notebook. A workflow of notebooks can be readily created when multiple notebooks can execute in JupyterLab tabs under one session of a powerful computer. Being able to run notebooks in tabs enables users to run entire projects/codebases consisting of several notebooks and scripts. Moreover, each JupyterLab session on Galaxy Europe requires a GPU and several CPUs and if it is allowed that users run a notebook on this set of resources, it may lead to non-optimized use of valuable compute resources. When many notebooks are updated, they can be committed together to a remote GitHub repository if they can be opened in tabs. Because of such benefits, it is preferable to use several tabs and they all run under one JupyterLab session. When a crashed JupyterLab session is reconnected, all the notebooks in different tabs start working again.

- "deep learning" in the Implementation section should be replaced by "DL" for consistency.

Author response:
This has been fixed in the manuscript.

- I think that readers would find a link to your tool on Galaxy Europe useful: https://usegalaxy.eu/root?tool_id=interactive_tool_ml_jupyter_notebook. The same is true for your tutorial: I think readers would find a URL in the text more easily than in the references.

Author response:
This is a very useful comment, thank you. GPU JupyterLab tool's link has been added to the training material at https://training.galaxyproject.org/training-material/topics/statistics/tutorials/gpu_jupyter_lab/tutorial.html. On Galaxy Europe, the tool can be searched in the tool search using text query such as "gpu jupyterlab".

However, the tool failed to execute on usegalaxy.edu with the following error message: "This tool is restricted to authorized users". I was unable to follow the tutorial. Was this a one-off issue with the Galaxy servers?

Author response:

The tool is open only to authorised users in order to optimise the usage of GPUs on Galaxy Europe's cluster. A Google form (http://usegalaxy.eu/gpu-request) has been created which users can use to apply for using this resource. Once the request is approved by a Galaxy admin made via this online form, the resource will become available to the authorised users within a day or two. More details on how to access this resource are given in Supplementary section S1. Since we do not restrict the usage of GPUs and users can run programs for days and months on these GPUs, it was used by bitcoin miners in the past. To prevent such usage and promote these resources only for research purposes, we have implemented such an approval method. The steps to apply for this resource:

Please follow these steps to access this infrastructure:

1. Create an account on Galaxy Europe (https://usegalaxy.eu/) using your official university email id
2. Apply for accessing GPU Jupyterlab using this Google form: http://usegalaxy.eu/gpu-request.
3. Use your official university email id in the Google form. This resource is available only for research purposes.
4. Wait up to 1-2 days to get the request approved.
5. Once approved, you will be able to run this resource on Galaxy Europe (https://usegalaxy.eu/).
6. If not authorised, then an error message will be shown.
7. Contact us at: contact@usegalaxy.eu if there are issues.

Reviewer #2: Kumar et al. present a Docker-based integration of Jupyter Notebooks in the Galaxy workflow system that can utilize GPUs. This notebook is also available in the Galaxy Europe instance.

I was able to create a Galaxy Europe account, find the newly introduced Galaxy tool and submit a job. However, it remained stuck with the message "This job is waiting to run" and the job info "Stopped" for multiple hours.

Author response:
The tool is open only to authorised users in order to optimise the usage of GPUs on Galaxy Europe's cluster. A Google form (http://usegalaxy.eu/gpu-request) has been created which users can use to apply for using this resource. Once the request is approved by a Galaxy admin made via this online form, the resource will become available to the authorised users within a day or two. More details on how to access this resource are given in Supplementary section S1. Since we do not restrict the usage of GPUs and users can run programs for days and months on these GPUs, it was used by bitcoin miners in the past. To prevent such usage and promote these resources only for research purposes, we have implemented such an approval method. The steps to apply for this resource:

Please follow these steps to access this infrastructure:
1. Create an account on Galaxy Europe (https://usegalaxy.eu/) using your official university email id
2. Apply for accessing GPU Jupyterlab using this Google form: http://usegalaxy.eu/gpu-request.
3. Use your official university email id in the Google form. This resource is available only for research purposes.
4. Wait up to 1-2 days to get the request approved.
5. Once approved, you will be able to run this resource on Galaxy Europe (https://usegalaxy.eu/).
6. If not authorised, then an error message will be shown.
7. Contact us at: contact@usegalaxy.eu if there are issues.

I was able to download the docker image and run it on a local server with multiple Nvidia GPUs. This resulted in a running Jupyter Lab, however running the GPU based examples resulted in driver mismatch errors/warnings (pynvml.nvml.NVMLError_LibRmVersionMismatch: RM has detected an NVML/RM version mismatch; kernel version 470.141.3 does not match DSO version 515.65.1 -- cannot find working devices in this configuration). Thus, the examples ran on CPU only. I did not try to resolve this issue and only

repeated some examples.

Author response:
NVIDIA and CUDA-related packages are now preinstalled in the container by running nvidia/cuda:11.8.0-cudnn8-runtime-ubuntu20.04 official docker image. In the old version of the Docker container, CUDA packages were manually installed in the Docker which could have caused the "driver mismatch" errors. To improve that we are using official NVIDIA containers (nvidia/cuda) as the base container that has prepackaged CUDA (version 11.8) and CuDNN packages. These are general-purpose containers that recognize GPUs on a host machine when NVIDIA drivers are installed. For deep learning to work with GPUs and TensorFlow, we install only TensorFlow-GPU separately in the container. When the container runs, it automatically recognises NVIDIA GPUs on the host machine.

The authors show two use-cases for the GPU Jupyter Docker and provide a step-by-step tutorial for usage on Galaxy Europe. Shipping machine learning applications that utilize GPUs as Jupyter Notebooks has become popular recently and supporting these through well-known and freely accessible Galaxy servers, such as Galaxy Europe, would be of clear benefit to users. Additionally, it would be very valuable for method developers like me to easily deploy GPU-based methods to Galaxy servers.

Author response:
Thank you for your encouraging thoughts.

Major:
- As mentioned before, I had issues getting a running Jupyter Lab on the Galaxy Europe server. Is this due to a limited number of GPUs or was this due to an error?

Author response:
It is because this tool in the past was available to all users and got exposed to bitcoin miners and they could misuse GPUs for days. Since then, it has been restricted only to authorised users. Moving forward, a Google form (http://usegalaxy.eu/gpu-request) has been implemented for researchers/users who would like to use this resource. Users should fill out this form using their respective university email ID and then this resource is made available for them within a day or two. More details on how to access this resource are given in Supplementary section S1.

Please follow these steps to access this infrastructure:
1. Create an account on Galaxy Europe (https://usegalaxy.eu/) using your official university email id
2. Apply for accessing GPU Jupyterlab using this Google form: http://usegalaxy.eu/gpu-request.
3. Use your official university email id in the Google form. This resource is available only for research purposes.
4. Wait up to 1-2 days to get the request approved.
5. Once approved, you will be able to run this resource on Galaxy Europe (https://usegalaxy.eu/).
6. If not authorised, then an error message will be shown.
7. Contact us at: contact@usegalaxy.eu if there are issues.

- Our ColabFold Multiple Sequence Alignment server currently processes about 10-20k MSAs per day. We do not know how many of these are running on Google Colab or on users' local machines. However, a substantial number of predictions are running inside Google Colab. The authors claim that Google Colab's and Kaggle's resources are scarce. However, generally, users (with either free or pro accounts) are given an instance nearly immediately on Colab. I recognize that it is extremely difficult to compete with these commercial platform providers. However, providing a long-term, freely available and securely funded, platform with ML accelerators would be extremely beneficial for the whole community. I would like to see a discussion on what GPU resources are currently available to users of Galaxy Europe (and the whole Galaxy

Project) and what plans exist to expand these in the future.

Author response:
Galaxy Europe has a large cluster of compute resources that not only comprises computers located in Freiburg, Germany but also at many other locations in Germany as well as other locations in Europe. Currently, only Galaxy's Freiburg cluster has over 8000 CPUs, 60 TB of memory, 5 PB of storage, and 16 GPUs. With the addition of the Pulsar network that connects Galaxy Europe to many different clusters across Europe, the compute power is expected to rise even further. For example, through the Pulsar network, Galaxy can connect to Science and Technology Facilities Council (STFC)'s approximately 30 additional GPUs.

- The size of the docker container (compressed ~10GB, uncompressed ~22GB) seems difficult to sustain.

Author response:
We have made the Docker container approximately 30% smaller (uncompressed size ~13 GB from ~22 GB and compressed size ~7 GB from ~10 GB) by using the official "nvidia/cuda:11.8.0-cudnn8-runtime-ubuntu20.04" as the base container instead of directly installing CUDA packages in the container.

Both keeping up an up-to-date Docker image and ensuring the availability of older images for reproducibility looks difficult to me, especially with such fast moving dependencies such as machine learning frameworks. How do the authors plan to deal with this issue?

Author response:
We have created a versioning system of Docker containers and its interactive Galaxy tool. The current starting version of the tool and the Docker container is at 0.2. As soon as the tools's or Docker container's version is updated in future, the versions of both the Docker container and the tool get updated. Moving forward, it will be possible to work with older versions of the tool and its associated docker container. Many other Galaxy tools maintain a list of all versions which users can choose.

Minor:
- Please highlight the tutorial (https://training.galaxyproject.org/training-material/topics/statistics/tutorials/gpu_jupyter_lab/tutorial.html) on GitHub and inside the container readme (home_page.ipynb). It is very easy to overlook. I also nearly overlooked the example notebook repository (https://github.com/anuprulez/gpu_jupyterlab_ct_image_segmentation). I found it confusing, that I could not find the two shown example use-cases inside the Docker container. I only later figured out that I have to clone the example repository into the running container.

Author response:
This is a good comment. We have added the link to the tutorial to the home page of the Docker container (home_page.ipynb) in the "How to use" section and also to the Docker container Github repository. The notebooks of the use-case repository have been made available in the Docker container.

- The manuscript highlights various workflow methods (elyra, kubeflow, airflow), however it needs clarification on how the Galaxy workflow integration works. I saw that it is possible to give input of another Galaxy output to the tool. I would appreciate a tutorial on how to make the GPU Jupyter Docker into part of a Galaxy workflow with multiple tools running. I think the above mentioned tutorials can be expanded to show how the output can be given to the next tool.

Author response:
In the Docker container, we have added a use-case of how to use Elyra AI to make a workflow of JupyterLab notebooks. In the "Elyra" folder there are a few notebooks and a pipeline file that runs these notebooks like a workflow. This pipeline file can be readily executed inside JupyterLab.

The feature of using the GPU JupyterLab as a tool in a Galaxy workflow has also been integrated. The tool takes an IPython script as input along with datasets produced by other Galaxy tools and produces output(s) as a collection/set of datasets that are created in the executed script. The tutorial shows a step-by-step approach to running this tool in a Galaxy workflow. Tutorial link:
https://training.galaxyproject.org/training-material/topics/statistics/tutorials/gpu_jupyter_lab/tutorial.html
(Please refer to section: GPU Juyterlab tool in a Galaxy workflow).

- Docker Hub has introduced many business-model changes such as deleting container images that are rarely used, which poses a challenge for reproducibility. I know that Dr Grüning is involved in the Biocontainers project. I would recommend investigating if it is possible to combine these efforts to make this GPU container and derived containers long term available.

Author response:
We have stored the Docker container in Quay.io (https://quay.io/repository/anupkumar/docker-ml-jupyterlab) as well. Quay.io is supported by RedHat and poses no time restriction on storage. In case Docker stops supporting our containers, we only need to change the container's URL in the JupyterLab tool's XML.

- The Docker container is explicitly running as a root user, while the manuscript highlights the security benefits of Docker. The cited report by Baset et al. highlights the security benefits and the many security challenges that Docker containers pose. I suggest checking what security best practices for Docker containers are possible to implement, while still allowing GPUs to be exposed to users.

Author response:
Towards making the GPU JupyterLab container more secure, the following measures have been taken. A custom user (non-root) has been created inside the container and this user is permitted to run this resource with no "root" permissions. All "ADD" commands have been replaced with "COPY" commands. To minimize running arbitrary commands inside a container, a trusted image from "nvidia/cuda" official list of images (by NVIDIA Corporation) has been used as the base image to extend this container. While allocating resources to Galaxy's jobs, Linux control groups (also known as CGROUPS) are used to isolate these resources from several running processes. We believe that such measures would greatly improve the security aspects of the GPU JupyterLab project.

- I recommend revising the manuscript for conciseness, with an additional focus on capitalization of words.

Author response:
We have revised the manuscript improving the text in terms of readability and capitalization wherever required.

Close
