# Supplementary material for: An accessible infrastructure for artificial intelligence using a Docker-based JupyterLab in Galaxy
Source: Gigascience. 2023 Apr 26;12:giad028. doi: 10.1093/gigascience/giad028 (PMC10132306; doi:10.1093/gigascience/giad028)
Supplement: giad028_Response_to_Reviewer_Comments_Revision_1 [file giad028_response_to_reviewer_comments_revision_1.pdf]

Author's Response To Reviewer Comments

Close

GIGA-D-22-00220R1
An accessible infrastructure for artificial intelligence using a Docker-based JupyterLab in Galaxy
Anup Kumar; Gianmauro Cuccuru; Björn Grüning; Rolf Backofen
GigaScience

Dear Mr. Kumar,

Your manuscript "An accessible infrastructure for artificial intelligence using a Docker-based JupyterLab in Galaxy" (GIGA-D-22-00220R1) has been assessed by our reviewers. Based on these reports, and my own assessment as Editor, I am pleased to inform you that it is potentially acceptable for publication in GigaScience, once you have carried out some minor revisions suggested by our reviewers.

Author response:
We would like to thank the reviewers and the editors.
--

Their reports, together with any other comments, are below. Please also take a moment to check our website at https://www.editorialmanager.com/giga/ for any additional comments that were saved as attachments.

In addition, please register any new software application in the bio.tools and SciCrunch.org databases to receive RRID (Research Resource Identification Initiative ID) and biotoolsID identifiers, and include these in your manuscript. Computational workflows should be registered in workflowhub.eu and the DOIs cited in the relevant places in the manuscript. These will facilitate tracking, reproducibility and re-use of your tool.

Author response:
Thank you. The manuscript mentions the following RRID and biotoolsID identifiers:
RRID: SCR_022695
Biotools ID: gpu-enabled_docker_container_with_jupyterlab_for_ai
--

Once you have made the necessary corrections, please submit a revised manuscript online at:

https://www.editorialmanager.com/giga/

If you have forgotten your username or password please use the "Send Login Details" link to get your login information. For security reasons, your password will be reset.

Please include a point-by-point within the 'Response to Reviewers' box in the submission system. Please ensure you describe additional experiments that were carried out and include a detailed rebuttal of any criticisms or requested revisions that you disagreed with. Please also ensure that your revised manuscript conforms to the journal style, which can be found in the Instructions for Authors on the journal homepage. If the data and code has been modified in the revision process please be sure to update the public versions of this too.

The due date for submitting the revised version of your article is 18 May 2023.

We look forward to receiving your revised manuscript soon.

Best wishes,

Hongling Zhou
GigaScience
www.gigasciencejournal.com

Reviewer reports:
Reviewer #1: Thank you for addressing my comments and suggestions. I look forward to seeing this article in print.

Author response:
Thank you.
--

Reviewer #2: Kumar et al. present the revised version of their manuscript "An accessible infrastructure for artificial intelligence using a docker-based Jupyterlab in Galaxy". The revised version is much improved compared to the initial version. I could now follow the authors' instructions to enable GPU support within Galaxy Europe and was able to execute the GPU-based ColabFold notebook and some of the other notebooks without any issues. The authors have resolved my previous points except as noted below. The remaining points should be resolvable during editing and should not require another review round.

Author response:
Thank you for your review comments.
--

Minor:
- In the authors' response it is mentioned that Galaxy Europe currently provides 16+30 GPUs. In the manuscript this is only referred to as "a few GPUs". I would recommend to state the currently available set of GPU resources in the manuscript.

Author response:
Thank you for your comment. The set of available GPUs within Galaxy Europe and through the Pulsar network are shared across many tools and processes. Only in the best scenario, all of them are available exclusively to the GPU-enabled JupyterLab in Galaxy. In future, the number of GPUs may increase too. In general, the resources might vary over time, not only for GPUs. Due to these reasons, we think it would be confusing for future readers if fixed numbers were stated in the manuscript as they are bound to change over time. To show this number, we have added a table at the Galaxy community site under (https://galaxyproject.org/news/2023-01-24-gpu-jupyterlab-galaxy/#current-resources-will-be-updated-regularly) that reports the availability of the current number of GPUs and other resources. This table is cited in the manuscript at appropriate places and the statistics of the table will be updated from time to time. The link is also referenced in the manuscript.
--

I also continue to recommend to not refer to GPU resources of commercial providers as "scarce". It's clear that its difficult or nearly impossible to compete with the big commercial providers, however, this doesn't detract from the usefulness of the provided service. My hope would be that clearly stating the current situation will also make the need clear to funders to sufficiently and sustainably fund computational resources in the future.

Author response:
Thank you for your comment. We have removed the word "scarce" and have added the "Sustaining and improving such an openly accessible infrastructure would highly benefit ML practitioners and researchers from various fields of science." sentence in the "Summary" section to emphasize the importance of this work. In addition, we would like to mention that Galaxy's unlimited computation time with a fixed set of resources provides a clear advantage over the commercial JupyterLab-like environments such as Google Colab in their free versions.

--

- There is still some odd phrasing in the manuscript. Two examples: In the beginning: ML [...] been [...] "vastly used" [...]. The authors referring to CPUs, when they mean CPU-cores.

Author response:
Thank you for your comment. We removed the odd phrasing from the manuscript and have accordingly updated it.
--

- One thing that could be improved within Galaxy is making it easier to open the "Active InteractiveTool". There is a "Display" button within the current history sidebar, however clicking that button just seems to return an error. It would be great if this button would directly go to the JupyterLab environment, instead of having to go through "User->Active InteractiveTools".

Milot Mirdita

Author response:
It's a very good comment, thank you. We have integrated this in the new Galaxy release. If the “eye/display” is used icon on your running JupyterLab notebook history entry, you will be directed to your Notebook. Similarly, a new icon (a group of few gear icons) appears on the Galaxy's topbar with quick access to "User->Active InteractiveTools" showing a collection of all accessed Interactive Tools in Galaxy. Further, we have plans for this year to integrate a new “Activity bar” and make Interactive Tools even more visible in Galaxy. Overall, all these UI/UX changes in Galaxy will benefit the GPU-enabled JupyterLab.

--

Close
