# Supplementary material for: An accessible infrastructure for artificial intelligence using a Docker-based JupyterLab in Galaxy
Source: Gigascience. 2023 Apr 26;12:giad028. doi: 10.1093/gigascience/giad028 (PMC10132306; doi:10.1093/gigascience/giad028)
Supplement: giad028_Reviewer_2_Report_Original_Submission — Milot Mirdita -- 10/26/2022 Reviewed [file giad028_reviewer_2_report_original_submission.pdf]

Reviewer Report

Title: An accessible infrastructure for artificial intelligence using a Docker-based JupyterLab in Galaxy

Version: Original Submission **Date:** 10/26/2022

Reviewer name: Milot Mirdita

Reviewer Comments to Author:

Kumar et al. present a Docker-based integration of Jupyter Notebooks in the Galaxy workflow system that can utilize GPUs. This notebook is also available in the Galaxy Europe instance.I was able to create a Galaxy Europe account, find the newly introduced Galaxy tool and submit a job. However, it remained stuck with the message "This job is waiting to run" and the job info "Stopped" for multiple hours. I was able to download the docker image and run it on a local server with multiple Nvidia GPUs. This resulted in a running Jupyter Lab, however running the GPU based examples resulted in driver mismatch errors/warnings (pynvml.nvml.NVMLError_LibRmVersionMismatch: RM has detected an NVML/RM version mismatch; kernel version 470.141.3 does not match DSO version 515.65.1 -- cannot find working devices in this configuration). Thus, the examples ran on CPU only. I did not try to resolve this issue and only repeated some examples.The authors show two use-cases for the GPU Jupyter Docker and provide a step-by-step tutorial for usage on Galaxy Europe. Shipping machine learning applications that utilize GPUs as Jupyter Notebooks has become popular recently and supporting these through well-known and freely accessible Galaxy servers, such as Galaxy Europe, would be of clear benefit to users. Additionally, it would be very valuable for method developers like me to easily deploy GPU-based methods to Galaxy servers.Major:- As mentioned before, I had issues getting a running Jupyter Lab on the Galaxy Europe server. Is this due to a limited number of GPUs or was this due to an error?- Our ColabFold Multiple Sequence Alignment server currently processes about 10-20k MSAs per day. We do not know how many of these are running on Google Colab or on users' local machines. However, a substantial number of predictions are running inside Google Colab. The authors claim that Google Colab's and Kaggle's resources are scarce. However, generally, users (with either free or pro accounts) are given an instance nearly immediately on Colab. I recognize that it is extremely difficult to compete with these commercial platform providers. However, providing a long-term, freely available and securely funded, platform with ML accelerators would be extremely beneficial for the whole community. I would like to see a discussion on what GPU resources are currently available to users of Galaxy Europe (and the whole Galaxy Project) and what plans exist to expand these in the future.- The size of the docker container (compressed ~10GB, uncompressed ~22GB) seems difficult to sustain. Both keeping up an up-to-date Docker image and ensuring the availability of older images for reproducibility looks difficult to me, especially with such fast moving dependencies such as machine learning frameworks. How do the authors plan to deal with this issue?Minor:- Please highlight the tutorial (https://training.galaxyproject.org/training-material/topics/statistics/tutorials/gpu_jupyter_lab/tutorial.html) on GitHub and inside the container readme (home_page.ipynb). It is very easy to overlook. I also nearly overlooked the example notebook repository (https://github.com/anuprulez/gpu_jupyterlab_ct_image_segmentation). I found it confusing, that I could not find the two shown example use-cases inside the Docker container. I only

later figured out that I have to clone the example repository into the running container.- The manuscript highlights various workflow methods (elyra, kubeflow, airflow), however it needs clarification on how the Galaxy workflow integration works. I saw that it is possible to give input of another Galaxy output to the tool. I would appreciate a tutorial on how to make the GPU Jupyter Docker into part of a Galaxy workflow with multiple tools running. I think the above mentioned tutorials can be expanded to show how the output can be given to the next tool.- Docker Hub has introduced many business-model changes such as deleting container images that are rarely used, which poses a challenge for reproducibility. I know that Dr GrÃ¼ning is involved in the Biocontainers project. I would recommend investigating if it is possible to combine these efforts to make this GPU container and derived containers long term available.- The Docker container is explicitly running as a root user, while the manuscript highlights the security benefits of Docker. The cited report by Baset et al. highlights the security benefits and the many security challenges that Docker containers pose. I suggest checking what security best practices for Docker containers are possible to implement, while still allowing GPUs to be exposed to users.- I recommend revising the manuscript for conciseness, with an additional focus on capitalization of words.Best regards,Milot Mirdita

Level of Interest

Please indicate how interesting you found the manuscript: Choose an item.

Quality of Written English

Please indicate the quality of language in the manuscript: Choose an item.

Declaration of Competing Interests

Please complete a declaration of competing interests, considering the following questions:

- Have you in the past five years received reimbursements, fees, funding, or salary from an organisation that may in any way gain or lose financially from the publication of this manuscript, either now or in the future?
- Do you hold any stocks or shares in an organisation that may in any way gain or lose financially from the publication of this manuscript, either now or in the future?
- Do you hold or are you currently applying for any patents relating to the content of the manuscript?
- Have you received reimbursements, fees, funding, or salary from an organization that holds or has applied for patents relating to the content of the manuscript?
- Do you have any other financial competing interests?
- Do you have any non-financial competing interests in relation to this paper?

If you can answer no to all of the above, write 'I declare that I have no competing interests' below. If your reply is yes to any, please give details below.

I declare that I have no competing interests

I agree to the open peer review policy of the journal. I understand that my name will be included on my report to the authors and, if the manuscript is accepted for publication, my named report including any attachments I upload will be posted on the website along with the authors' responses. I agree for my report to be made available under an Open Access Creative Commons CC-BY license (http://creativecommons.org/licenses/by/4.0/). I understand that any comments which I do not wish to be included in my named report can be included as confidential comments to the editors, which will not be published.

Choose an item.

To further support our reviewers, we have joined with Publons, where you can gain additional credit to further highlight your hard work (see: https://publons.com/journal/530/gigascience). On publication of this paper, your review will be automatically added to Publons, you can then choose whether or not to claim your Publons credit. I understand this statement.

Yes Choose an item.
