# Supplementary material for: An accessible infrastructure for artificial intelligence using a Docker-based JupyterLab in Galaxy
Source: Gigascience. 2023 Apr 26;12:giad028. doi: 10.1093/gigascience/giad028 (PMC10132306; doi:10.1093/gigascience/giad028)
Supplement: giad028_Reviewer_2_Report_Revision_1 — Milot Mirdita -- 2/16/2023 Reviewed [file giad028_reviewer_2_report_revision_1.pdf]

Reviewer Report

Title: An accessible infrastructure for artificial intelligence using a Docker-based JupyterLab in Galaxy

Version: Revision 1 **Date:** 2/16/2023

Reviewer name: Milot Mirdita

Reviewer Comments to Author:

Kumar et al. present the revised version of their manuscript "An accessible infrastructure for artificial intelligence using a docker-based Jupyterlab in Galaxy". The revised version is much improved compared to the initial version. I could now follow the authors' instructions to enable GPU support within Galaxy Europe and was able to execute the GPU-based ColabFold notebook and some of the other notebooks without any issues. The authors have resolved my previous points except as noted below. The remaining points should be resolvable during editing and should not require another review round.Minor:- In the authors' response it is mentioned that Galaxy Europe currently provides 16+30 GPUs. In the manuscript this is only referred to as "a few GPUs". I would recommend to state the currently available set of GPU resources in the manuscript. I also continue to recommend to not refer to GPU resources of commercial providers as "scarce". It's clear that its difficult or nearly impossible to compete with the big commercial providers, however, this doesn't detract from the usefulness of the provided service. My hope would be that clearly stating the current situation will also make the need clear to funders to sufficiently and sustainably fund computational resources in the future.- There is still some odd phrasing in the manuscript. Two examples: In the beginning: ML [...] been [...] "vastly used" [...]. The authors referring to CPUs, when they mean CPU-cores.- One thing that could be improved within Galaxy is making it easier to open the "Active InteractiveTool". There is a "Display" button within the current history sidebar, however clicking that button just seems to return an error. It would be great if this button would directly go to the JupyterLab environment, instead of having to go through "User->Active InteractiveTools".Milot Mirdita

Level of Interest

Please indicate how interesting you found the manuscript: Choose an item.

Quality of Written English

Please indicate the quality of language in the manuscript: Choose an item.

Declaration of Competing Interests

Please complete a declaration of competing interests, considering the following questions:

- Have you in the past five years received reimbursements, fees, funding, or salary from an organisation that may in any way gain or lose financially from the publication of this manuscript, either now or in the future?
- Do you hold any stocks or shares in an organisation that may in any way gain or lose financially from the publication of this manuscript, either now or in the future?
- Do you hold or are you currently applying for any patents relating to the content of the manuscript?
- Have you received reimbursements, fees, funding, or salary from an organization that holds or has applied for patents relating to the content of the manuscript?
- Do you have any other financial competing interests?
- Do you have any non-financial competing interests in relation to this paper?

If you can answer no to all of the above, write 'I declare that I have no competing interests' below. If your reply is yes to any, please give details below.

I declare that I have no competing interests

I agree to the open peer review policy of the journal. I understand that my name will be included on my report to the authors and, if the manuscript is accepted for publication, my named report including any attachments I upload will be posted on the website along with the authors' responses. I agree for my report to be made available under an Open Access Creative Commons CC-BY license (http://creativecommons.org/licenses/by/4.0/). I understand that any comments which I do not wish to be included in my named report can be included as confidential comments to the editors, which will not be published.

Choose an item.

To further support our reviewers, we have joined with Publons, where you can gain additional credit to further highlight your hard work (see: https://publons.com/journal/530/gigascience). On publication of this paper, your review will be automatically added to Publons, you can then choose whether or not to claim your Publons credit. I understand this statement.

Yes Choose an item.
